# Transition Metal‐Free Heteroarene Insertion Into C─C Bonds of Benzocyclobutenones

**DOI:** 10.1002/anie.6371423

**Published:** 2026-06-20

**Authors:** Cole J. Wagner, Guangbin Dong

**Affiliations:** ^1^ Department of Chemistry University of Chicago Chicago Illinois United States

**Keywords:** benzocyclobutenones, C–C activation, fused rings, heteroaryne, transition‐metal free

## Abstract

The insertion of five‐membered heteroarenes into the C─C bonds of benzocyclobutenones (BCBs) has been achieved. This process proceeds under mild conditions and does not require the use of a transition metal catalyst. A variety of fused polycyclic compounds based on indoles, 7‐azaindoles, benzothiophenes, benzofurans, and thiophenes relevant to bioactive scaffolds are efficiently accessed through this approach. The reaction is scalable, and the products are suitable for various late‐stage derivatizations. Preliminary mechanistic studies reveal a catalytic role of base in facilitating C─C bond cleavage to generate a reactive intermediate which likely engages in subsequent dearomative cycloaddition with heteroarenes.

## Graphical abstract

A transition metal‐free insertion of five‐membered heteroarenes into the C─C bonds of benzocyclobutenones has been achieved under mild and operationally simple conditions. This method provides an efficient and modular approach to access fused partially saturated hetero‐polycyclic scaffolds that exist in many bioactive molecules.

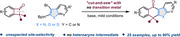

## 1 Introduction

**SCHEME 1:**
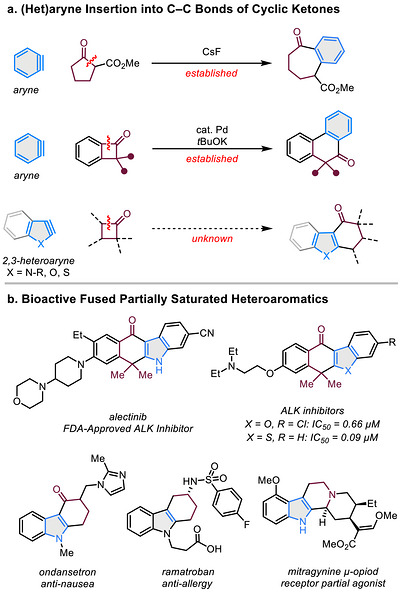
(a) (Hetero)aryne insertion into C–C bonds of cyclic ketones. (b) Bioactive compounds potentially accessible via heteroaryne insertion into C–C bonds. ALK, anaplastic lymphoma kinase; this kinase is implicated in various cancers.

Arene‐ and heteroarene‐derived partially saturated rings are privileged scaffolds in bioactive compounds [1]. From the synthetic efficiency standpoint, the direct insertion of arynes into C─C bonds of more saturated rings, for example, cyclic ketones, provides a straightforward strategy to construct benzo‐fused skeletons (Scheme 1a) [2, 3, 4, 5, 6]. Such a process can be achieved either via a formal [2+2] cycloaddition between arynes and ketone enolates followed by elimination [7, 8, 9, 10, 11] or capitalizing on transition metal‐catalyzed C─C bond activation [12, 13]. However, the analogous insertion of heteroarynes into C─C bonds remains elusive [14] despite the prevalence of heteroarene‐fused semi‐saturated ring systems in pharmaceutically relevant molecules (Scheme 1b) [15, 16, 17, 18, 19].

**SCHEME 2:**
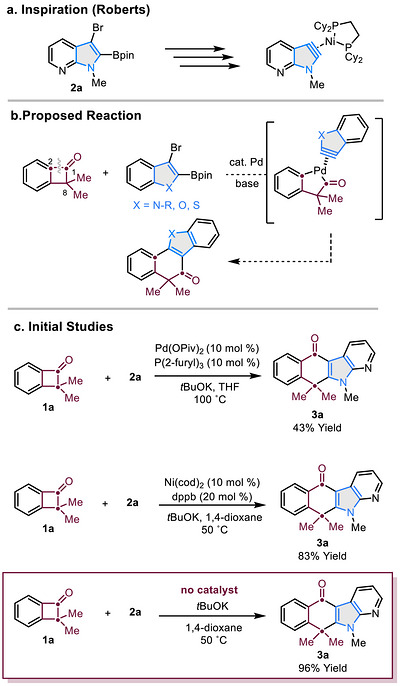
(a) Prior work on forming 2,3‐indolynes of a Ni complex. (b) Proposed “cut‐and‐sew” insertion of 2,3‐heteroarynes into BCBs. (c) Initial studies toward proposed formal 2,3‐indolyne insertion into BCBs.

Unlike arynes, generation of 2,3‐heteroarynes is much more challenging owing to the substantial strain incurred by the presence of a triple bond within a 5‐membered ring [20]. Pioneering work by Roberts and coworkers demonstrated for the first time that 2,3‐indolyne complexes of nickel can be synthesized, isolated, and applied to a handful of transformations (Scheme 2a) [21, 22, 23]. A similar strategy has been used to access 2,3‐(benzo)thiophynes, although these are less strained due to the elongated C─S bonds [24]. While these organometallic species hold great potential as valuable synthons, their application to C─C bond insertion reactions has yet to be demonstrated.

Given our ongoing interest in the development of “cut‐and‐sew” transformations [25, 26, 27, 28] for accessing complex molecular skeletons, we proposed a strategy to insert 2,3‐heteroarynes into C─C bonds of benzocyclobutenones (BCBs) (Scheme 2b) [29, 30, 31, 32, 33, 34]. Based on our prior success of the Pd‐catalyzed aryne insertion into C–C bonds of BCBs [13], it was expected that the generated metallo‐heteroaryne intermediate, according to Roberts’ work [21], should insert into the C1–C2 bond of BCBs, which should lead to 6‐6‐5‐6 heteroarene‐fused tetracycles.

## 2 Results and Discussion

We commenced this study by reacting BCB **1a** and 2,3‐indolyne precursor **2a** (Scheme 2c). Under both the palladium and nickel catalysis conditions, the formal 2,3‐indolyne insertion product **3a** was obtained as a single regioisomer. Intriguingly, the insertion took place at the bulkier C1–C8 bond in contrast to the typical C1–C2 reactivity observed in “cut‐and‐sew” reactions of BCBs, especially those with C8 substitution [35, 36]. Further control experiments show that the reaction can surprisingly take place in the absence of any transition metal to deliver tetracycle **3a** in high yield, and only the base is essential for the reactivity. After further optimization, product **3a** can be formed in 96% yield using *t*BuOK as the base in 1,4‐dioxane at 50°C (Table 1, entry 1). Typically, any **1a** that did not productively react with **2a** underwent decomposition with poor mass balance, whereas the mass balance of **2a** was generally high with protodeborylation being the most prominent side reaction. The product is reasonably stable under the reaction conditions, as prolonged reaction time to 36 h did not much reduce the yield (entry 2). While 1,4‐dioxane was found to be ideal, other solvents worked similarly well (entries 3 and 4). In the absence of base, no conversion of neither **1a** nor **2a** was detected (entry 5). Among various *tert*‐butoxide bases, the potassium one was most effective, though decent yields can still be obtained with *t*BuOLi and *t*BuONa (entries 6 and 7). The reduced efficiency with *t*BuOLi and *t*BuONa is possibly due to their slightly worse dissociation of *tert‐*butoxide ion compared to *t*BuOK. Stronger bases such as KHMDS mainly led to decomposition (entry 8), while less hindered NaOMe resulted in low yield of **3a** and a substantial conversion of **2a** to the corresponding protodeborylation product (entry 9). Of note, this reaction proceeds in good efficiency even at room temperature (entry 10).

**TABLE 1:** Selected optimization. Standard conditions (entry 1): 0.1 mmol **1a**, 0.15 mmol **2a**, 0.15 mmol *t*BuOK, and 0.5 mL 1,4‐dioxane for 24 h at 50°C.

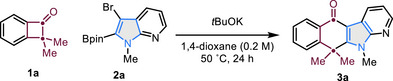				
Entries	Base	Solvents	Equiv. 2a/Base	Yield 3a^a^
1	*t*BuOK	1,4‐Dioxane	1.5	96%
2^b^	*t*BuOK	1,4‐Dioxane	1.5	94%
3	*t*BuOK	THF	1.5	93%
4	*t*BuOK	Toluene	1.5	95%
5	None	1,4‐Dioxane	—	n.d.
6	*t*BuOLi	1,4‐Dioxane	2	57%
7	*t*BuONa	1,4‐Dioxane	2	63%
8	KHMDS	1,4‐Dioxane	2	n.d.
9	NaOMe	1,4‐Dioxane	2	2%
10^c^	*t*BuOK	1,4‐Dioxane	2	84%

^a^ Yields determined using CH_2_Br_2_ as the internal standard.

^b^ 36 h Reaction time.

^c^ Reaction ran at room temperature.

With the optimized conditions in hand, we then sought to explore the scope of this cut‐and‐sew reaction between BCBs and 2,3‐heteroaryne synthons (Table 2). First, the scope of BCBs was investigated using *N*‐methyl‐substituted 7‐azaindole derivative **2a** as the coupling partner. Electron‐rich BCBs smoothly afforded the corresponding [4+2] adducts (**3b** and **3c**). The C4‐methyl‐substituted product (**3d**) was also obtained in good yield from BCB **3d**. In addition, BCBs with fluorine substitutions at all aryl positions (**1e–1h**) can work, though the yield dropped as the fluorine substituent was moved closer to the carbonyl group of the BCB. Moreover, disubstitution at the BCB C8 position was found to be necessary for the insertion to occur (see Supporting Information for details). The substituents at the α‐carbon could be altered, and bulkier *n*‐propyl‐substituted BCB **1i** successfully reacted with **2a** to give tetracycle **3i** in high yield. The unsymmetrically α‐disubstituted BCB **1j** could also deliver the corresponding product **3j** effectively. Spirocyclic substitution at the α‐carbon could also be tolerated where pentacycles **3k** and **3l** could be obtained from the reaction of **2a** with **1k** and **1l**, respectively. Notably, *N*‐benzyl‐protected 7‐azaindole derivative **2b** could also give the C–C insertion product (**3m**) in high yield.

**TABLE 2:** Substrate scope of the “cut‐and‐sew” reaction between BCBs and 2,3‐difunctionalized heteroarenes.

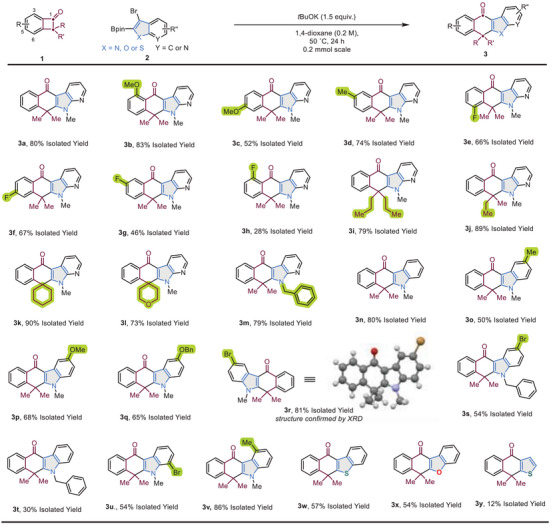

Regarding the heteroarene part, besides 7‐azaindoles, indole derivatives are also competent coupling partners. Simple indole (**3n**) and C5‐bromo‐indole (**3r**) both afforded the desired products in high yield. Owing to the transition metal‐free feature, aryl bromide moieties can be well tolerated, which could serve as a handle to introduce additional functional groups through late‐stage derivatizations (vide infra, Scheme 6b). Gratifyingly, the structure of **3r** was unambiguously confirmed via x‐ray crystallography, confirming the regioselectivity and site‐selectivity of this reaction [37]. The more electron‐rich methyl‐ and alkoxy‐substituted indoles gave somewhat lower yield (**3o–3q**). Similarly, *N*‐benzyl substituted indoles are suitable coupling partners, albeit with reduced yield, likely due to the increased sterics around the reaction site. **3u**, bearing a 7‐bromoindole moiety, could also be accessed. The 4‐methylindole‐based product **3v** was obtained in high yield, despite the steric encumbrance between the 4‐methyl and the carbonyl groups. Notably, C4 substituents were found incompatible when forming metallo‐indolynes [23]. Electron‐poor indoles were generally unproductive and resulted predominantly in protodeborylation products (see Supporting Information for challenging substrates). Gratifyingly, this transformation is not limited to indole and azaindole scaffolds. Both benzofuran and benzothiophene‐derived tetracycle products (**3w** and **3x**) can be synthesized in good yield via the same manner. Moreover, simple thiophene, as a non‐benzofused heteroarene, can also be inserted to give tricyclic product **3y** [38], albeit in low yield at this stage.

**SCHEME 3:**
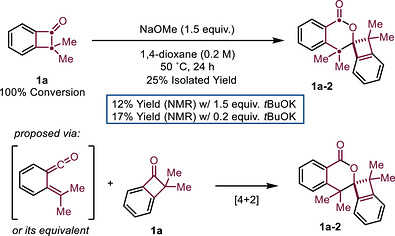
Reaction of BCBs in the absence of the heteroarene coupling partner.

To gain some insights into the mechanism of the transition metal‐free “cut‐and‐sew” transformation, a number of control experiments were carried out. First, we examined the reactivity of BCB **1a** under the basic conditions in the absence of the heteroarene coupling partner (Scheme 3). It was found that BCB **1a** was fully consumed and, while the mass balance was generally poor with formation of unidentifiable side‐products, an interesting dimerization product **1a‐2** was isolated. The lactone moiety in **1a‐2** could potentially be formed via a formal [4+2] reaction between a vinyl ketene (or its equivalent) derived from **1a** and the carbonyl from another **1a** molecule [39]. Notably, even if only 20 mol% *t*BuOK was used, full consumption of BCB **1a** was observed, indicating that a catalytic amount of base is sufficient to trigger C–C bond cleavage. Efforts to trap the vinyl ketene intermediate with electron‐rich alkenes and alkynes remain unfruitful. In the absence of base, no dimer **1a‐2** was observed with most of BCB **1a** recovered, suggesting a critical role of base in activating the BCB C–C bond.

**SCHEME 4:**
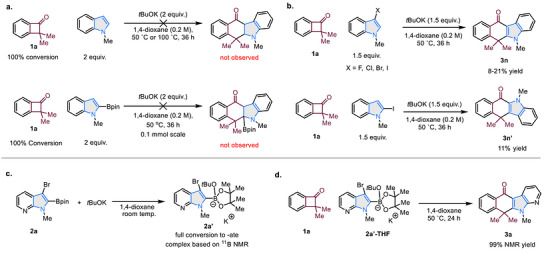
Mechanistic study. (a) Role of the C2‐boryl substitution on the indole coupling partner. (b) Role of the halogen substitution on indole coupling partner. (c) Analysis of ‐*ate* complex formation. (d) Stoichiometric reaction using the ‐*ate* complex.

Next, control experiments on the indole coupling partner were conducted (Scheme 4). The questions we asked were: 1) whether both of the C2 and C3 substituents on the indole are necessary for the reactivity and 2) whether 2,3‐heteroaryne is an actual intermediate in this reaction. It was found that simple *N*‐methyl indole and 2‐boryl‐*N*‐methylindole did not undergo any sort of [4+2] reaction with **1a** (even at elevated temperature), with only decomposition of **1a** under basic conditions observed (Scheme 4a). Intriguingly, in the absence of the boryl group, all 3‐halo‐indoles can afford the desired cut‐and‐sew product (**3n**) under the otherwise standard conditions (Scheme 4b), suggesting that the boryl group is not essential to the reactivity. In addition, the use of isomeric 2‐iodo‐*N*‐methylindole showed a flip in the regioselectivity, delivering product **3n’**. Although the reason why the halogen substitution plays such a crucial role and how it affects regiochemical outcome of the insertion is still under investigation, these results together can rule out the involvement of a 2,3‐indolyne intermediate in this reaction.

Subsequently, we attempted to determine the kinetic orders of each reactant using initial rates. BCB **1a** was found to be roughly first order, implicating its involvement in the rate‐determining step (RDS) (see Figure S8). With regard to azaindole **2a**, when the initial concentration of **2a** was decreased, rate enhancement was observed. However, when the initial concentration of **2a** was increased such that the ratio of **2a** to *t*BuOK was greater than 1, we saw complete inhibition of the reaction. While this phenomenon prevented us from obtaining an accurate numerical value for the kinetic order of **2a**, it nevertheless indicated that there is a strong inhibitory effect of **2a** on the RDS. In fact, after mixing a 1:1 ratio of **2a** and *t*BuOK for only a few minutes and analyzing by ^11^B NMR, all of **2a** was converted to the corresponding *‐ate* complex **2a’** through binding with *t*BuOK, and almost no signal corresponding to the uncoordinated boron could be detected (Scheme 4c).

This suggests a strong equilibrium between free *t*BuOK and boron‐bound base. To better understand this effect, we prepared and isolated a THF‐bound boronate (**2a’‐THF**). When this complex was subjected to reaction with BCB **1a**, nearly quantitative yield of **3a** was obtained (Scheme 4d). This improved efficiency may also explain why the 2‐boryl substitution on the indole affords much higher yield in comparison to the 3‐haloindoles. In the case of the latter, a low yield of product was obtained accompanied by full consumption of BCB **1a** and leftover 3‐haloindole, likely because a high concentration of free base can rapidly consume BCBs, leading to unproductive side‐reactions. By comparison, in the reaction of BCB **1a** and indole **2a** with *t*BuOK, the Bpin group of **2a** may serve to essentially buffer the reaction mixture and control the concentration of free base such that the reaction of BCB **1a** with *t*BuOK is tapered to enable a more selective reaction in which **1a** is not quickly decomposed in the presence of too high of a concentration of free base. Additionally, the existence of this equilibrium may explain why less bulky alkoxides and electron poor indoles do not work well as the stabilized *‐ate* complexes formed from either of these components may not as readily release the alkoxide that is needed to react with the BCB. Instead, protodeborylation of the indole occurs most predominantly in these cases.

**SCHEME 5:**
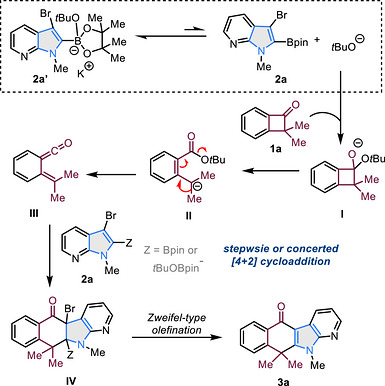
Plausible reaction mechanism.

While the exact mechanism of this transformation remains a topic of ongoing investigation [40], a plausible reaction pathway is tentatively proposed (Scheme 5). First, *t*BuOK reacts with boronate **2a** to form *‐ate* complex **2a’**, which exists in an equilibrium to slowly release the alkoxide. The free alkoxide can attack the carbonyl of BCB **1a** to form a tetrahedral intermediate **I**. Strain release then drives the cleavage of the C1–C8 bond to form a benzylic anion **II**. The subsequent elimination of *tert*‐butoxide leads to the formation of vinyl ketene **III**. The regenerated *tert*‐butoxide serves as a nucleophile catalyst to continue isomerizing BCB **1a** to vinyl ketene **III** [41, 42]. This vinyl ketene could possibly then undergo either stepwise or concerted dearomative [4+2] addition with indole **2a** to form indoline **IV** [31]. Finally, the elimination of boryl bromide via Zweifel‐type olefination furnishes the tetracyclic product (**3a**). It is noteworthy that an anion‐triggered halogen transfer mechanism cannot be excluded [43].

**SCHEME 6:**
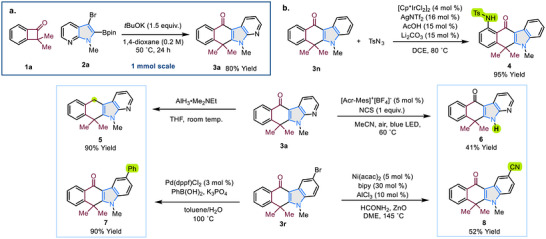
Synthetic utilities. (a) 1 mmol Scale reaction. (b) Derivatizations of the tetracyclic products.

The synthetic utilities of this reaction have also been explored (Scheme 6). First, the reaction could be scaled up (1 mmol) with no decrease in yield (Scheme 6a). In addition, the carbonyl group of **3n** could be utilized as a directing group in Chang's Ir‐catalyzed C–H amination to afford sulfonamide **4** in high yield [44]. Moreover, the carbonyl group could also be fully reduced in high yield using alane to give product **5**. Importantly, the free azaindole **6** could be accessed via a photochemical demethylation [45]. Finally, the bromo‐substituted tetracycle (**3r**) can undergo smooth cross couplings to introduce a phenyl group via the Pd‐catalyzed Suzuki–Miyaura reaction or a nitrile moiety via the Ni‐catalyzed cyanation (Scheme 6b) [46].

## 3 Conclusion

In conclusion, we have developed a transition metal‐free “cut‐and‐sew” reaction via inserting five‐membered heteroarenes into the C–C bonds of BCBs. Compared to the previous approaches, this annulation method exhibits complementary site‐selectivity for cleaving the C1–C8 bond of BCBs and complete regioselectivity for the heteroarene insertion. It offers modular access to fused hetero‐polycyclic frameworks that are not trivial to prepare otherwise. The unusual mechanistic pathway uncovered in this study could have broad implications on developing other heteroarene‐involved cycloaddition reactions. Efforts on extending the coupling partners to different dienophiles for accessing diverse ring systems are ongoing.

## Conflicts of Interest

The authors declare no conflicts of interest.

## Supporting information




**Supporting File 1**: The authors have cited additional references within the Supporting Information [47–66].

**Supporting File 2**: anie73250‐sup‐0002‐Data.zip.

## Data Availability

The data that supports the findings of this study are available in the Supporting Information of this article.
