# Supplementary material for: Transition Metal‐Free Heteroarene Insertion Into C─C Bonds of Benzocyclobutenones
Source: Angew Chem Int Ed Engl. 2026 Jun 20;65(32):e6371423. doi: 10.1002/anie.6371423 (PMC13427194; doi:10.1002/anie.6371423)

Table of Contents

General Information 2

Unsuccessful Substrates 3

Synthesis of Starting Materials 4

Insertion of 2,3-Difunctionalized Heteroarenes Into Benzocyclobutenones 17

Product Derivatizations 37

Mechanistic Experiments 43

References 69

X-Ray Crystallography Data 71

NMR Spectra 75

General Information

Unless otherwise noted, all solvents were dried by filtration using a Pure-Solv MD-5 Solvent Purification System (Innovative Technology), all reactions were performed under a nitrogen atmosphere, all reaction vessels were flame-dried prior to use, and all commercially available compounds were used without further purification. All solvents used in the 2,3-difunctionalized heteroarene [4+2] reaction were further dried *via* distillation from sodium/benzophenone. All 2,3-difunctionalized heteroarene [4+2] reactions were carried out in 4 mL vials sealed with PTFE-lined caps purchased from Qorpak. *t*BuOK used in the 2,3-difunctionalized heteroarene [4+2] reaction was stored in an inert atmosphere glovebox prior to use. All nuclear magnetic resonance (NMR) data (^{1}H NMR, ^{13}C NMR, ^{11}B NMR, ^{19}F NMR, ^{31}P NMR, ^{13}C DEPT-135 NMR, ^{1}H-^{1}H COSY, ^{1}H-^{13}C HSQC, and ^{1}H-^{13}C HMBC) were recorded with spectrometers from Bruker (^{1}H: 400, 500, or 600 MHz; ^{13}C: 101, 126, or 151 MHz; ^{11}B: 128, 160, or 193 MHz; ^{19}F: 376, 470, or 565 MHz; ^{31}P: 243 MHz) unless otherwise noted. Chemical shifts for ^{1}H NMR spectra were reported in ppm using tetramethylsilane at 0.00 ppm, $CDCl_3$ at 7.26 ppm, acetone-*d*6 at 2.05 ppm, DMSO-*d*6 at 2.50 ppm, or C_6D_6 at 7.16 ppm as standards. Chemical shifts for ^{13}C NMR spectra were reported in ppm with the center line of the triplet signal for $CDCl_3$ at 77.0 ppm, C_6D_6 at 128.06 ppm, acetone-*d*6 at 29.84 ppm, or DMSO-*d*6 at 39.52 ppm. Coupling constants (*J*) were reported in Hertz. All crude NMR yields were calculated using CH_2Br_2 as an internal standard. High-resolution mass spectra (HMRS) were obtained using an Agilent 6224 ToF-MS spectrometer using multimode (MM) ionization. Data were reported for the molecular ion $[M]^+$, $[M+H]^+$, $[M+Na]^+$, or $[M+NH_4]^+$. IR spectra were recorded on a Nicolet iS5 FT-IR spectrometer using sample solutions in $CHCl_3$ on a KBr salt plate and signals are reported in inverse centimeters (cm^{-1}).Thin-layer chromatography (TLC) analysis was run on silica gel plates purchased from EMD Chemical (silica gel 60, F254) and spots were visualized using ultraviolet light (254 nm), $KMnO_4$ stains, and ceric ammonium molybdenite stains. All transition metal-free control experiments were run using brand new magnetic stir bars and brand new, single-use 4 mL reaction vials. Additionally, none of the substrates or reagents used in the control experiments were prepared using any transition metal-catalyzed reactions.

Unsuccessful Substrates

The benzocyclobutenones **I-V** and cyclobutenone **VI** shown below underwent unproductive decomposition and did not deliver any C–C insertion product. Similarly, the indoles **VII-XII** shown below did not undergo C–C insertion. In general, electron poor indoles and indoles with bulky *N*-substituents were unproductive and predominantly underwent protodeborylation.

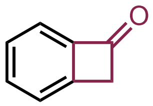

I

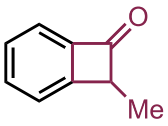

II

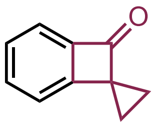

III

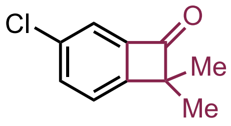

IV

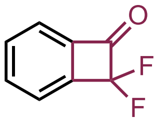

V

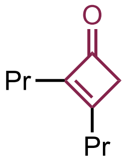

VI

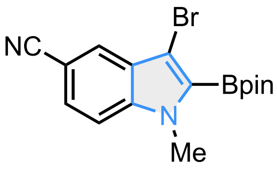

VII

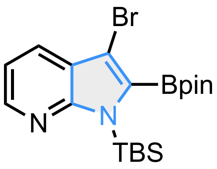

VIII

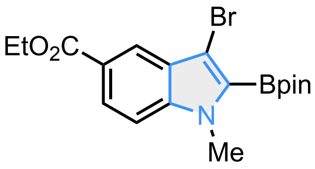

IX

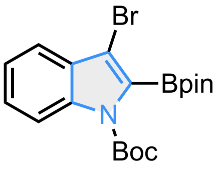

X

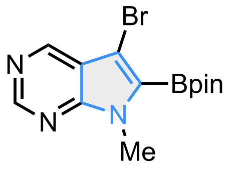

XI

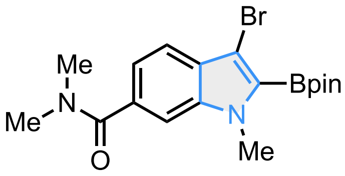

XII

Synthesis of Starting Materials

Benzocyclobutenones **1a** – **1h** and **1j** – **1l**[47] and **1i**[48] are known compounds and were prepared following literature procedures, and their characterization data matched their reported data.

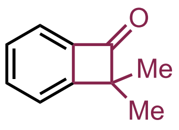
1a

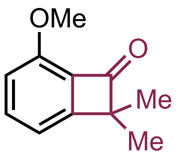
1b

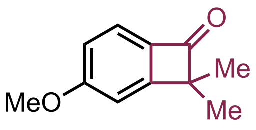
1c

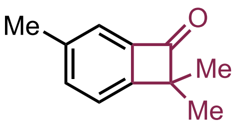
1d

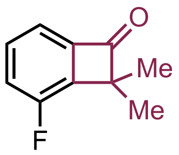
1e

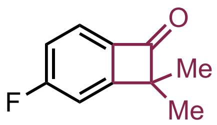
1f

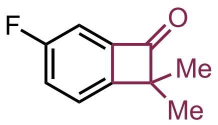
1g

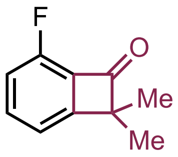
1h

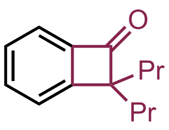
1i

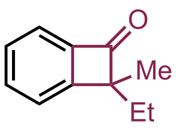
1j

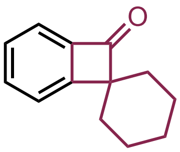
1k

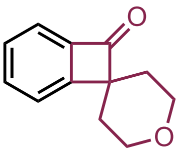
1l

Indoles **2a** and **2b**[49], **2c** and **2d**[50], benzothiophene **2m**[51], and thiophene **2n**[52] are known compounds and were prepared following literature procedures, and their characterization data matched their reported data.

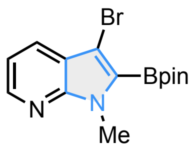
2a

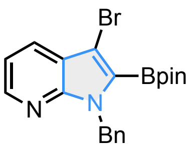
2b

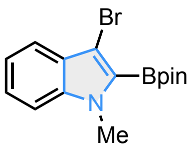
2c

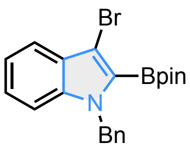
2i

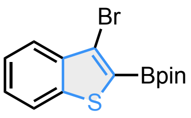
2m

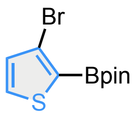
2n

Intermediate compounds **S1**,[53] **S3**,[54] **S4**,[55] **S6**,[56] **S8**,[57] **S9**,[58] **S11**[59], and **S13**[60] are known compounds and were prepared following literature procedures, and their characterization data matched their reported data.

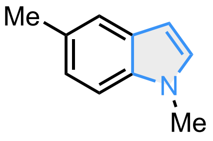

S1

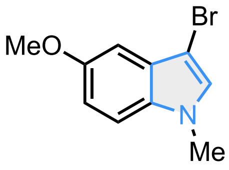

S3

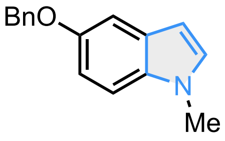

S4

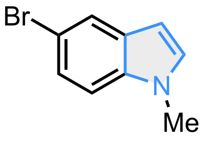

S6

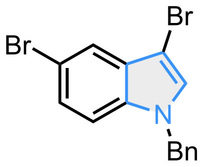

S8

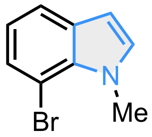

S9

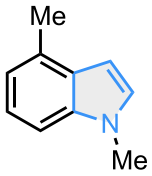

S11

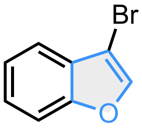

S13

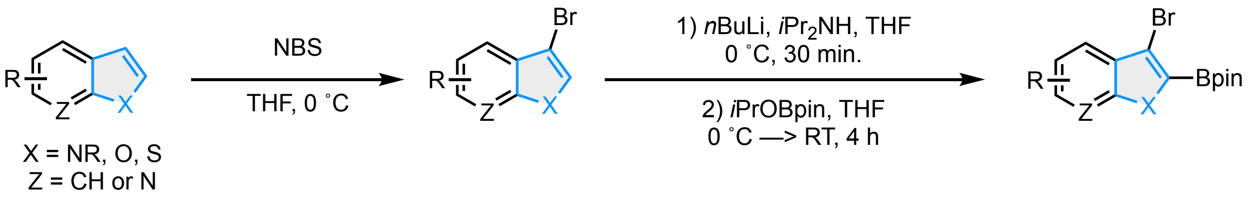

General Procedure **A** for C3 Bromination of *N*-methylindoles

NBS (1 equiv.) was added to a solution of *N*-methylindole (1 equiv.) in THF (0.15 M) at 0 ˚C. The reaction was monitored by TLC and upon completion, it was quenched by the addition of sat. aq. $NaHCO_3$ solution and EtOAc. The aqueous phase was extracted 3 times with EtOAc. The combined organic phases were washed with brine, dried with $MgSO_4$, and concentrated under reduced pressure. Purification by flash chromatography (Hexanes / EtOAc) gave the 3-bromo-*N*-methylindole product.

General Procedure **B** for C2 borylation of 3-bromo-*N*-methylindoles

Based on a known procedure,[21] *n*BuLi (2.5 M in hexanes, 1.2 equiv.) was added to a solution of diisopropylamine (1.2 equiv.) in THF (0.3 M) at 0 ˚C. After stirring at 0 ˚C for 30 minutes, a solution of 3-bromo-*N*-methylindole (1 equiv.) and *i*PrOBpin (1.35 equiv.) in THF (0.6 M) was added dropwise *via* a syringe. The reaction mixture was warmed to room temperature and stirred for 4 hours. Upon completion, the reaction was quenched with sat. aq. NH_4Cl solution and EtOAc. The aqueous phase was extracted three times with EtOAc. The combined organic phases were washed with water, brine, dried with $MgSO_4$, and concentrated under reduced pressure.

Purification by flash chromatography (Hexanes / EtOAc) gave the functionalized heteroarene product.

Synthesis of **S2**

Following General Procedure **A**, NBS (1.6324 g, 9.2 mmol, 1 equiv.) was added to a solution of **S1** (1.3392 g, 9.2 mmol, 1 equiv.) in THF (60 mL) at 0 ˚C. The reaction was monitored by TLC and upon completion, it was quenched by the addition of sat. aq. $NaHCO_3$ solution and EtOAc. The aqueous phase was extracted 3 times with EtOAc. The combined organic phases were washed with brine, dried with $MgSO_4$, and concentrated under reduced pressure. Purification by flash chromatography (20:1 Hexanes / EtOAc) gave 0.5720 g of **S2** as a pale yellow liquid in 28% yield.

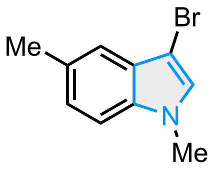

S2

^{1}H NMR (600 MHz, $CDCl_3$) δ 7.35 (s, 1H), 7.20 (d, *J* = 8.4 Hz, 1H), 7.10 (dd, *J* = 8.4, 1.8 Hz, 1H), 7.03 (s, 1H), 3.75 (s, 3H), 2.49 (s, 3H).

^{13}C NMR (151 MHz, $CDCl_3$) δ 134.73, 129.56, 127.67, 127.43, 124.32, 118.80, 109.20, 88.67, 33.08, 21.40.

IR (KBr Plate, cm^{-1}): 3117.4, 3020.2, 2916.6, 2822.3, 1572.2, 1516.4, 1490.9, 1444.1, 1423.1, 1359.2, 1334.3, 1293.0, 1240.7, 1194.4, 1152.8, 1112.2, 1039.6, 962.0, 867.1, 844.0, 784.0, 599.1, 534.0

R_f: 0.55 (10:1 Hexanes / EtOAc)

HRMS: Calc. $[M+H]^+$ for $C_{10}H_{10}BrN$ = 224.0075 ; found = 224.0078

Synthesis of **2d**

Following General Procedure **B**, *n*BuLi (2.5 M in hexanes, 1.22 mL, 3.06 mmol, 1.2 equiv.) was added to a solution of diisopropylamine (0.44 mL, 3.06 mmol, 1.2 equiv.) in THF (10 mL) at 0 ˚C. After stirring at 0 ˚C for 30 minutes, a solution of **S2** (0.5720 g, 2.55 mmol, 1 equiv.) and *i*PrOBpin (0.71 mL, 3.45 mmol, 1.35 equiv.) in THF (5 mL) was added dropwise *via* a syringe.

The reaction mixture was warmed to room temperature and stirred for 4 hours. Upon completion, the reaction was quenched with sat. aq. NH_4Cl solution and EtOAc. The aqueous phase was extracted 3 times with EtOAc. The combined organic phases were washed with water, brine, dried with $MgSO_4$, and concentrated under reduced pressure. Purification by flash chromatography (20:1 Hexanes / EtOAc) gave 0.6533 g of **2d** as a white solid in 73% yield.

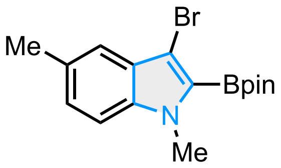

2d

^{1}H NMR (500 MHz, CDCl$_3$) δ 7.39 – 7.36 (m, 1H), 7.21 (d, J = 8.5 Hz, 1H), 7.12 (dd, J = 8.5, 1.6 Hz, 1H), 3.92 (s, 3H), 2.46 (s, 3H), 1.39 (s, 13H).

^{13}C NMR (126 MHz, CDCl$_3$) δ 137.80, 129.47, 127.81, 126.13, 119.57, 109.50, 102.04, 83.91, 32.81, 24.86, 21.35.

^{11}B NMR (160 MHz, CDCl$_3$) δ 28.83.

IR (KBr Plate, cm^{-1}): 3018.9, 2977.1, 2931.2, 2860.6, 1514.6, 1481.8, 1446.5, 1381.3, 1311.4, 1281.1, 1264.8, 1233.2, 1166.7, 1142.6, 1081.4, 963.1, 853.0, 791.1, 692.5, 670.1

R$_f$: 0.65 (4:1 Hexane / EtOAc)

HRMS: Calc. $[M+H]^+$ for $C_{16}H_{21}BBrNO_2$ = 350.0927 ; found = 350.0929

MP: 85.1 – 86.3 ˚C

Synthesis of **2e**

Following General Procedure **B**, *n*BuLi (2.5 M in hexanes, 1.37 mL, 3.44 mmol, 1.2 equiv.) was added to a solution of diisopropylamine (0.49 mL, 3.44 mmol, 1.2 equiv.) in THF (12 mL) at 0 ˚C. After stirring at 0 ˚C for 30 minutes, a solution of **S3** (0.6873 g, 2.86 mmol, 1 equiv.) and *i*PrOBpin (0.79 mL, 3.86 mmol, 1.35 equiv.) in THF (5 mL) was added dropwise *via* a syringe. The reaction mixture was warmed to room temperature and stirred for 4 hours. Upon completion, the reaction was quenched with sat. aq. NH_4Cl solution and EtOAc. The aqueous phase was extracted 3 times with EtOAc. The combined organic phases were washed with water, brine, dried with $MgSO_4$, and concentrated under reduced pressure. Purification by flash chromatography (10:1 Hexanes / EtOAc) gave 0.6290 g of **2e** as a white solid in 60% yield.

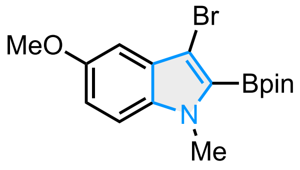

2e

^{1}H NMR (500 MHz, CDCl$_3$) δ 7.21 (d, J = 8.8 Hz, 1H), 6.99 (d, J = 2.0 Hz, 1H), 6.96 (dd, J = 8.9, 2.4 Hz, 1H), 3.93 (s, 3H), 3.89 (s, 3H), 1.40 (s, 12H).

^{13}C NMR (126 MHz, CDCl$_3$) δ 154.63, 134.72, 127.85, 115.81, 110.82, 101.77, 100.26, 83.92, 55.74, 32.93, 24.86.

^{11}B NMR (160 MHz, CDCl$_3$) δ 28.57.

IR (KBr plate, cm^{-1}): 3067.7, 2977.7, 2937.4, 2832.3, 1621.4, 1573.4, 1511.1, 1453.6, 1380.4, 1316.9, 1264.2, 1211.8, 1170.6, 1143.1, 1110.6, 1085.0, 1032.6, 963.9, 853.7, 832.4, 797.9, 713.2, 692.8, 670.2, 624.2, 578.8

R$_f$: 0.57 (4:1 Hexane / EtOAc)

HRMS: Calc. $[M+H]^+$ for $C_{16}H_{21}BBrNO_3$ = 366.0876 ; found = 366.0876

MP: 84.7 – 85.3 ˚C

Synthesis of **S5**

Following General Procedure **A**, NBS (0.5312 g, 3 mmol, 1 equiv.) was added to a solution of **S4** (0.7173 g, 3 mmol, 1 equiv.) in THF (20 mL) at 0 ˚C. The reaction was monitored by TLC and upon completion, it was quenched by the addition of sat. aq. $NaHCO_3$ solution and EtOAc. The aqueous phase was extracted 3 times with EtOAc. The combined organic phases were washed with brine, dried with $MgSO_4$, and concentrated under reduced pressure. Purification by flash chromatography (20:1 Hexanes / EtOAc) gave 0.4872 g of **S5** as oily tan wax in 51% yield.

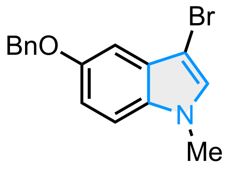

S5

^{1}H NMR (500 MHz, $CDCl_3$) δ 7.50 (d, *J* = 7.5 Hz, 2H), 7.40 (t, *J* = 7.4 Hz, 2H), 7.33 (t, *J* = 7.4 Hz, 1H), 7.21 (d, *J* = 8.8 Hz, 1H), 7.09 (d, *J* = 2.3 Hz, 1H), 7.05 (s, 1H), 7.01 (dd, *J* = 8.9, 2.4 Hz, 1H), 5.14 (s, 2H), 3.75 (s, 3H).

^{13}C NMR (126 MHz, $CDCl_3$) δ 153.89, 137.42, 131.70, 128.55, 128.13, 127.88, 127.69, 127.59, 114.02, 110.46, 101.95, 88.66, 70.83, 33.23.

IR (KBr Plate, cm^{-1}): 3116.7, 3062.7, 3030.9, 2904.4, 1621.0, 1572.6, 1511.7, 1488.7, 1452.7, 1423.1, 1384.3, 1284.1, 1238.4, 1217.6, 1201.1, 1158.6, 1133.2, 1109.1, 1025.0, 875.4, 831.4, 786.1, 738.3, 704.2, 622.8, 606.1

R_f: 0.33 (9:1 Hexane / EtOAc)

HRMS: Calc. $[M+H]^+$ for $C_{16}H_{14}BrNO$ = 318.0317 ; found = 318.0319

Synthesis of **2f**

Following General Procedure **B**, *n*BuLi (2.5 M in hexanes, 0.74 mL, 1.85 mmol, 1.2 equiv.) was added to a solution of diisopropylamine (0.26 mL, 1.85 mmol, 1.2 equiv.) in THF (6 mL) at 0 ˚C. After stirring at 0 ˚C for 30 minutes, a solution of **S5** (0.4872 g, 1.54 mmol, 1 equiv.) and *i*PrOBpin (0.43 mL, 2.1 mmol, 1.35 equiv.) in THF (3 mL) was added dropwise *via* a syringe. The reaction mixture was warmed to room temperature and stirred for 4 hours. Upon completion, the reaction was quenched with sat. aq. NH_4Cl solution and EtOAc. The aqueous phase was extracted 3 times with EtOAc. The combined organic phases were washed with water, brine, dried with $MgSO_4$, and concentrated under reduced pressure. Purification by flash chromatography (20:1 Hexanes / EtOAc) gave 0.3940 g of **2f** as a white solid in 57% yield.

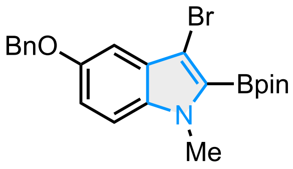

2f

^{1}H NMR (500 MHz, $CDCl_3$) δ 7.51 – 7.47 (m, 2H), 7.42 – 7.37 (m, 2H), 7.35 – 7.30 (m, 1H), 7.23 (d, *J* = 9.0 Hz, 1H), 7.10 (d, *J* = 2.4 Hz, 1H), 7.05 (dd, *J* = 8.9, 2.4 Hz, 1H), 5.13 (s, 2H), 3.93 (s, 3H), 1.40 (s, 12H).

^{13}C NMR (126 MHz, $CDCl_3$) δ 153.81, 137.37, 134.88, 128.53, 127.87, 127.85, 127.75, 116.31, 110.83, 101.88, 101.82, 83.93, 70.69, 32.93, 24.86.

^{11}B NMR (160 MHz, $CDCl_3$) δ 29.14.

IR (KBr Plate, cm^{-1}): 3063.8, 3031.9, 2977.0, 2932.1, 1619.3, 1509.1, 1453.8, 1379.8, 1314.5, 1263.2, 1231.7, 1213.4, 1186.6, 1142.0, 1110.4, 1084.5, 1025.2, 963.3, 895.8, 855.4, 830.7, 797.4, 725.8, 695.1, 622.8

R_f: 0.42 (8:1 Hexane / EtOAc)

HRMS: Calc. $[M+H]^+$ for $C_{22}H_{25}BBrNO_3$ = 444.1169 ; found = 444.1166

MP: 153.4 – 153.8 ˚C

Synthesis of **S7**

Following General Procedure **A**, NBS (1.77 g, 10 mmol, 1 equiv.) was added to a solution of **S6** (2.1007 g, 10 mmol, 1 equiv.) in THF (60mL) at 0 ˚C. The reaction was monitored by TLC and upon completion, it was quenched by the addition of sat. aq. $NaHCO_3$ solution and EtOAc. The aqueous phase was extracted 3 times with EtOAc. The combined organic phases were washed with brine, dried with $MgSO_4$, and concentrated under reduced pressure. Purification by flash chromatography (10:1 Hexanes / EtOAc) gave 1.8379 g of **S7** as a brown solid in 64% yield.

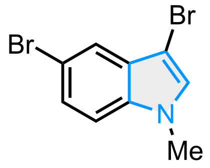

S7

^{1}H NMR (500 MHz, $CDCl_3$) δ 7.69 (d, *J* = 1.8 Hz, 1H), 7.34 (dd, *J* = 8.7, 2.0 Hz, 1H), 7.17 (d, *J* = 8.7 Hz, 1H), 7.06 (s, 1H), 3.75 (s, 3H).

^{13}C NMR (126 MHz, $CDCl_3$) δ 135.00, 128.89, 128.85, 125.59, 121.92, 113.57, 111.03, 88.57, 33.25.

IR (KBr plate, cm^{-1}): 3113.2, 2941.7, 2818.4, 1728.1, 1563.7, 1513.5, 1470.7, 1420.8, 1354.1, 1332.4, 1266.4, 1236.0, 1172.0, 1110.7, 1054.1, 1037.3, 953.9, 864.0, 782.2, 592.9, 533.4

R_f: 0.33 (4:1 Hexane / EtOAc)

HRMS: Calc. $[M+H]^+$ for $C_9H_7Br_2N$ = 289.9003 ; found = 289.8995

MP: 55.2 – 55.8 ˚C

Synthesis of **2g**

Following General Procedure **B**, *n*BuLi (2.5 M in hexanes, 3.05 mL, 7.63 mmol, 1.2 equiv.) was added to a solution of diisopropylamine (1.08 mL, 7.63 mmol, 1.2 equiv.) in THF (25 mL) at 0 ˚C. After stirring at 0 ˚C for 30 minutes, a solution of **S7** (1.8379 g, 6.36 mmol, 1 equiv.) and *i*PrOBpin (1.75 mL, 8.59 mmol, 1.35 equiv.) in THF (10 mL) was added dropwise *via* a syringe. The reaction mixture was warmed to room temperature and stirred for 4 hours. Upon completion, the reaction was quenched with sat. aq. NH_4Cl solution and EtOAc. The aqueous phase was extracted 3 times with EtOAc. The combined organic phases were washed with water, brine, dried with $MgSO_4$, and concentrated under reduced pressure. Purification by flash chromatography (10:1 Hexanes / EtOAc) gave 1.5443 g of **2g** as a white solid in 58% yield.

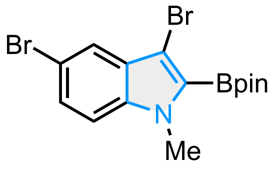

2g

^{1}H NMR (500 MHz, $CDCl_3$) δ 7.74 (d, J = 2.0 Hz, 1H), 7.35 (dd, J = 8.8, 2.0 Hz, 1H), 7.18 (d, J = 9.3 Hz, 1H), 3.92 (s, 3H), 1.40 (s, 12H).

^{13}C NMR (126 MHz, $CDCl_3$) δ 137.86, 129.24, 127.12, 122.77, 113.48, 111.33, 101.60, 84.19, 32.99, 24.86.

^{11}B NMR (160 MHz, $CDCl_3$) δ 28.52.

IR (KBr plate, cm^{-1}): 3060.8, 2978.3, 2933.8, 1508.9, 1469.2, 1445.6, 1379.3, 1317.4, 1286.1, 1256.3, 1229.5, 1142.5, 1110.7, 1088.1, 963.5, 952.1, 848.7, 790.6, 734.1, 696.6, 599.9

R_f: 0.63 (4:1 Hexane / EtOAc)

HRMS: Calc. $[M+H]^+$ for $C_{15}H_{18}BBr_2NO_2$ = 415.9855 ; found = 415.9855

MP: 86.4 – 87.1 ˚C

Synthesis of **2h**

Following General Procedure **B**, *n*BuLi (2.5 M in hexanes, 2.95 mL, 7.38 mmol, 1.2 equiv.) was added to a solution of diisopropylamine (1.04 mL, 7.38 mmol, 1.2 equiv.) in THF (25 mL) at 0 ˚C. After stirring at 0 ˚C for 30 minutes, a solution of **S8** (2.2451 g, 6.15 mmol, 1 equiv.) and *i*PrOBpin (1.69 mL, 8.30 mmol, 1.35 equiv.) in THF (10 mL) was added dropwise *via* a syringe. The reaction mixture was warmed to room temperature and stirred for 4 hours. Upon completion, the reaction was quenched with sat. aq. NH_4Cl solution and EtOAc. The aqueous phase was extracted 3 times with EtOAc. The combined organic phases were washed with water, brine, dried with $MgSO_4$, and concentrated under reduced pressure. Purification by flash chromatography (10:1 Hexanes / EtOAc) gave 1.9457 g of **2h** as a white solid in 64% yield.

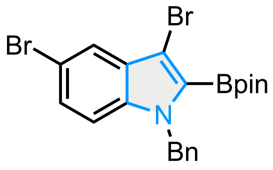

2h

^{1}H NMR (500 MHz, $CDCl_3$) δ 7.78 (d, *J* = 1.9 Hz, 1H), 7.30 (dd, *J* = 8.8, 1.9 Hz, 1H), 7.25 – 7.18 (m, 3H), 7.15 (d, *J* = 8.8 Hz, 1H), 7.00 – 6.95 (m, 2H), 5.63 (s, 2H), 1.27 (s, 12H).

^{13}C NMR (126 MHz, $CDCl_3$) δ 138.30, 137.61, 129.55, 128.56, 127.42, 127.27, 126.14, 122.94, 113.74, 111.84, 102.64, 84.22, 49.44, 24.68.

^{11}B NMR (160 MHz, $CDCl_3$) δ 28.59.

IR (KBr plate, cm^{-1}): 3064.0, 3030.6, 2978.2, 2931.8, 1738.4, 1605.3, 1509.5, 1452.9, 1380.5, 1345.4, 1318.2, 1292.1, 1259.4, 1181.2, 1167.3, 1138.4, 1100.8, 950.0, 848.6, 792.7, 732.4, 695.7, 431.8, 420.8

R_f: 0.67 (4:1 Hexane / EtOAc)

HRMS: Calc. $[M+H]^+$ for $C_{21}H_{22}BBr_2NO_2$ = 492.0168 ; found = 492.0158

MP: 130.6 – 131.8 ˚C

Synthesis of **S10**

Following General Procedure **A**, NBS (3.68 g, 16 mmol, 1.3 equiv.) was added to a solution of **S9** (3.3672 g, 16 mmol, 1 equiv.) in THF (100 mL) at 0 ˚C. The reaction was monitored by TLC and upon completion, it was quenched by the addition of sat. aq. $NaHCO_3$ solution and EtOAc. The aqueous phase was extracted 3 times with EtOAc. The combined organic phases were washed with brine, dried with $MgSO_4$, and concentrated under reduced pressure. Purification by flash chromatography (Hexanes) gave 1.7836 g of **S10** as a white solid in 38% yield.

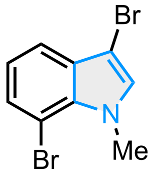

S10

^{1}H NMR (500 MHz, $CDCl_3$) δ 7.49 (dd, *J* = 7.9, 1.1 Hz, 1H), 7.39 (dd, *J* = 7.6, 1.0 Hz, 1H), 7.02 (s, 1H), 7.00 (t, *J* = 7.8 Hz, 1H), 4.14 (s, 3H).

^{13}C NMR (126 MHz, $CDCl_3$) δ 132.68, 130.61, 130.20, 127.67, 121.21, 118.95, 103.85, 89.27, 37.01.

IR (KBr plate, cm^{-1}): 3111.4, 3067.7, 2998.8, 2947.0, 2912.4, 1607.5, 1551.4, 1518.0, 1485.1, 1466.2, 1445.0, 1403.1, 1330.8, 1319.7, 1303.5, 1205.5, 1108.7, 1042.5, 966.6, 819.1, 806.8, 773.9, 728.3, 583.4

R_f: 0.74 (4:1 Hexane / EtOAc)

HRMS: Calc. $[M+H]^+$ for $C_9H_7Br_2N$ = 289.9003 ; found = 289.9009

MP: 69.4 – 70.5 ˚C

Synthesis of **2j**

Following General Procedure **B**, *n*BuLi (2.5 M in hexanes, 1.66 mL, 4.15 mmol, 1.2 equiv.) was added to a solution of diisopropylamine (0.59 mL, 4.15 mmol, 1.2 equiv.) in THF (14 mL) at 0 ˚C. After stirring at 0 ˚C for 30 minutes, a solution of **S10** (1.00 g, 3.46 mmol, 1 equiv.) and *i*PrOBpin (0.95 mL, 4.67 mmol, 1.35 equiv.) in THF (6 mL) was added dropwise *via* a syringe.

The reaction mixture was warmed to room temperature and stirred for 4 hours. Upon completion, the reaction was quenched with sat. aq. NH_4Cl solution and EtOAc. The aqueous phase was extracted 3 times with EtOAc. The combined organic phases were washed with water, brine, dried with $MgSO_4$, and concentrated under reduced pressure. Purification by flash chromatography (8:1 Hexanes / EtOAc) gave 1.1736 g of **2j** as a white solid in 58% yield.

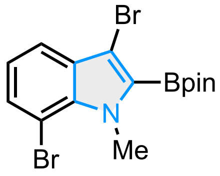

2j

^{1}H NMR (500 MHz, $CDCl_3$) δ 7.54 (dd, *J* = 8.0, 1.1 Hz, 1H), 7.44 (dd, *J* = 7.5, 1.1 Hz, 1H), 6.96 (t, *J* = 7.7 Hz, 1H), 4.34 (s, 3H), 1.40 (s, 13H).

^{13}C NMR (126 MHz, $CDCl_3$) δ 135.43, 130.63, 129.56, 121.05, 119.99, 103.90, 102.67, 84.32, 36.17, 24.82.

^{11}B NMR (160 MHz, $CDCl_3$) δ 28.67.

IR (KBr plate, cm^{-1}): 3061.8, 2977.5, 1517.6, 1379.1, 1331.5, 1261.5, 1200.0, 1142.2, 1110.5, 1085.5, 962.5, 851.3, 775.9, 729.8,

R_f: 0.78 (4:1 Hexane / EtOAc)

HRMS: Calc. $[M+H]^+$ for $C_{15}H_{18}BBr_2NO_2$ = 415.9855 ; found = 415.9862

MP: 121.7 – 122.1 ˚C

Synthesis of **S12**

Following General Procedure **A**, NBS (1.2213 g, 6.9 mmol, 1.0 equiv.) was added to a solution of **S11** (1.00 g, 6.9 mmol, 1 equiv.) in THF (50 mL) at 0 ˚C. The reaction was monitored by TLC and upon completion, it was quenched by the addition of sat. aq. $NaHCO_3$ solution and Et_2O. The aqueous phase was extracted 3 times with Et_2O. The combined organic phases were washed with brine, dried with $MgSO_4$, and concentrated under reduced pressure. Purification by flash chromatography (20:1 Pentane / Et_2O) gave 0.5619 g of **S12** as a white solid in 37% yield. This compound was found to be extremely prone to decomposition to form a dark orange/brown solid and should be used for the next step quickly or stored under nitrogen in a freezer. All rotovap

operations were carried out without heating of the water bath. NMR spectra were not collected as **S12** rapidly decomposed upon solvation in various NMR solvents, but the presence of **S12** was confirmed by low-resolution GC/MS using a sample dissolved in EtOAc, which appeared to be a suitable solvent for it. The product was thus carried on to the synthesis of **2k** and was used quickly after synthesis.

Synthesis of **2k**

Following General Procedure **B**, *n*BuLi (2.5 M in hexanes, 0.61 mL, 1.52 mmol, 1.2 equiv.) was added to a solution of diisopropylamine (0.22 mL, 1.52 mmol, 1.2 equiv.) in THF (5 mL) at 0 ˚C. After stirring at 0 ˚C for 30 minutes, a solution of **S12** (284.5 mg, 1.27 mmol, 1 equiv.) and *i*PrOBpin (0.35 mL, 1.71 mmol, 1.35 equiv.) in THF (2.5 mL) was added dropwise *via* a syringe. The reaction mixture was warmed to room temperature and stirred for 4 hours. Upon completion, the reaction was quenched with sat. aq. NH_4Cl solution and Et_2O. The aqueous phase was extracted 3 times with Et_2O. The combined organic phases were washed with water, brine, dried with $MgSO_4$, and concentrated under reduced pressure. Purification by flash chromatography (20:1 Pentane / Et_2O) gave 195.0 mg of **2k** as a white solid in 44% yield.

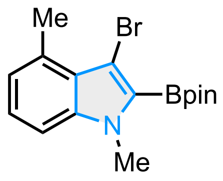

2k

^{1}H NMR (600 MHz, $CDCl_3$) δ 7.18 (d, *J* = 8.4 Hz, 1H), 7.14 (dd, *J* = 8.4, 6.8 Hz, 1H), 6.84 (dt, *J* = 6.8, 1.0 Hz, 1H), 3.90 (s, 3H), 2.86 (s, 3H), 1.40 (s, 12H).

^{13}C NMR (151 MHz, $CDCl_3$) δ 139.60, 132.19, 124.99, 123.95, 121.73, 107.93, 101.44, 83.96, 32.82, 24.84, 20.01.

^{11}B NMR (193 MHz, $CDCl_3$) δ 28.83.

IR (KBr plate, cm^{-1}): 3047.2, 2977.1, 2927.0, 1574.3, 1509.0, 1379.3, 1314.8, 1290.7, 1261.7, 1240.2, 1213.7, 1142.2, 1085.5, 1031.8, 962.4, 858.6, 771.7, 740.7, 694.8, 667.7

R_f: 0.46 (9:1 Hexanes / Et_2O)

HRMS: Calc. $[M+H]^+$ for $C_{16}H_{21}BBrNO_2$ = 350.0927 ; found = 350.0931

MP: 99.8 – 100.5 ˚C

Synthesis of **2l**

Following General Procedure **B**, *n*BuLi (2.5 M in hexanes, 1.98 mL, 4.96 mmol, 1.2 equiv.) was added to a solution of diisopropylamine (0.70 mL, 4.96 mmol, 1.2 equiv.) in THF (16 mL) at 0 ˚C. After stirring at 0 ˚C for 30 minutes, a solution of **S13** (0.8144 g, 4.1 mmol, 1 equiv.) and *i*PrOBpin (1.13 mL, 5.54 mmol, 1.35 equiv.) in THF (7 mL) was added dropwise *via* a syringe. The reaction mixture was warmed to room temperature and stirred for 4 hours. Upon completion, the reaction was quenched with sat. aq. NH_4Cl solution and EtOAc. The aqueous phase was extracted 3 times with EtOAc. The combined organic phases were washed with water, brine, dried with $MgSO_4$, and concentrated under reduced pressure. Purification by flash chromatography (4:1 Hexanes / EtOAc) gave 0.3464 g of **2l** as a brown solid in 26% yield.

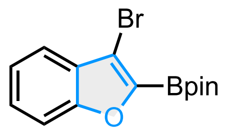

2l

^{1}H NMR (500 MHz, CDCl$_3$) δ 7.59 (d, *J* = 7.8 Hz, 1H), 7.55 (d, *J* = 8.3 Hz, 1H), 7.40 (ddd, *J* = 8.4, 7.1, 1.3 Hz, 1H), 7.33 – 7.29 (m, 1H), 1.41 (s, 12H).

^{13}C NMR (126 MHz, CDCl$_3$) δ 156.56, 127.91, 127.10, 123.37, 120.65, 112.21, 111.38, 84.87, 24.83.

^{11}B NMR (160 MHz, CDCl$_3$) δ 27.65.

IR (KBr plate, cm^{-1}): 3064.8, 2978.5, 2930.5, 1612.3, 1561.1, 1476.3, 1447.9, 1380.1, 1362.6, 1339.8, 1323.7, 1260.0, 1142.4, 1119.4, 1096.5, 995.1, 835.9, 748.1, 693.6

R$_f$: 0.20 (4:1 Hexane / EtOAc)

HRMS: Calc. $[M+NH_4]^+$ for $C_{14}H_{16}BBrO_3$ = 342.0699 ; found = 342.0715

MP: 105.6 -106.1 ˚C

Insertion of 2,3-Difunctionalized Heteroarenes Into Benzocyclobutenones

General Procedure **C** for the 2,3-Difunctionalized Heteroarene Insertion into C–C Bonds

Benzocyclobutenone **1** (0.2 mmol, 1 equiv.) and heteroarene **2** (0.3 mmol, 1.5 equiv.) were added to a flame-dried 4 mL vial. The vial was transferred into an inert atmosphere glovebox. *t*BuOK (33.7 mg, 0.3 mmol, 1.5 equiv.) and 1,4-dioxane (1.0 mL, 0.2 M) were added. The vial was sealed, removed from the glovebox, and stirred at 50 ˚C for 24 hours. Upon completion, the reaction mixture was diluted with EtOAc. The organic phase was washed 3 times with sat. aq. $NaHCO_3$ solution, dried with $MgSO_4$, and concentrated under reduced pressure. Purification by flash chromatography (Hexanes / EtOAc or Hexanes / Acetone) gave product **3**.

Synthesis of **3a**

Following general procedure **C** and reacting BCB **1a** with indole **2a**, purification by flash chromatography (5:1 Hexanes / Acetone) gave 44.4 mg of **3a** as an off-white solid in 80% yield.

Following general procedure **C** and scaling up to 1.0 mmol scale, BCB **1a** (146.2 mg, 1.0 mmol, 1 equiv.) and indole **2a** (505.5 mg, 1.5 mmol, 1.5 equiv.) were added to a flame-dried 40 mL vial equipped with a magnetic stir bar. The vial was transferred into an inert atmosphere glovebox. *t*BuOK (168.3 mg, 1.5 mmol, 1.5 equiv.) and 1,4-dioxane (5 mL) were added. The vial was sealed, removed from the glovebox, and stirred at 50 ˚C for 24 hours. Upon completion, the reaction mixture was diluted with EtOAc. The organic phase was washed 3 times with sat. aq. $NaHCO_3$ solution, dried with $MgSO_4$, and concentrated under reduced pressure. Purification by flash chromatography (5:1 Hexane / EtOAc) gave 222.2 mg of **3a** as an off-white solid in 80% yield.

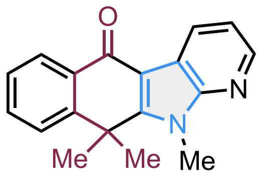

3a

^{1}H NMR (600 MHz, CDCl$_3$) δ 8.74 (d, *J* = 7.8 Hz, 1H), 8.42 (d, *J* = 5.0 Hz, 1H), 8.38 (d, *J* = 7.9 Hz, 1H), 7.67 (d, *J* = 7.9 Hz, 1H), 7.62 (t, *J* = 7.6 Hz, 1H), 7.46 (t, *J* = 7.4 Hz, 1H), 7.30 – 7.27 (m, 1H), 4.21 (s, 3H), 1.92 (s, 6H).

^{13}C NMR (151 MHz, CDCl$_3$) δ 180.53, 155.21, 149.76, 149.21, 144.21, 132.35, 130.98, 130.38, 126.93, 126.29, 126.14, 118.77, 117.20, 109.36, 37.38, 31.22, 28.11.

IR (KBr plate, cm^{-1}): 3060.2, 2984.1, 2938.2, 1643.2, 1600.1, 1579.4, 1524.1, 1485.8, 1459.3, 1432.4, 1402.4, 1256.8, 1231.7, 1087.6, 886.4, 791.7, 763.9, 692.9, 660.8, 552.0

R$_f$: 0.41 (2:1 Hexane / EtOAc)

HRMS: Calc. $[M+H]^+$ for $C_{18}H_{16}N_2O$ = 277.1341 ; found = 277.1344

MP: 142.8-143.5 ˚C

Synthesis of **3b**

Following general procedure **C** and reacting BCB **1b** with indole **2a**, purification by flash chromatography (5:1 Hexanes / Acetone) gave 51.2 mg of **3b** as yellow solid in 83% yield.

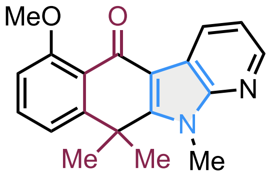

3b

^{1}H NMR (500 MHz, CDCl$_3$) δ 8.80 (d, *J* = 7.7 Hz, 1H), 8.38 (d, *J* = 4.8 Hz, 1H), 7.54 (t, *J* = 8.2 Hz, 1H), 7.28 – 7.19 (m, 2H), 6.97 (d, *J* = 8.3 Hz, 1H), 4.15 (s, 3H), 4.01 (s, 3H), 1.88 (s, 6H).

^{13}C NMR (126 MHz, CDCl$_3$) δ 181.18, 160.69, 152.79, 152.78, 149.69, 143.89, 132.95, 130.54, 120.29, 118.73, 118.54, 117.59, 110.59, 110.32, 56.29, 37.60, 31.02, 28.71.

IR (KBr plate, cm^{-1}): 3056.6, 2992.8, 2936.8, 2835.8, 1636.8, 1594.1, 1575.2, 1476.1, 1440.3, 1402.6, 1296.8, 1267.1, 1250.5, 1198.4, 1154.5, 1105.8, 1048.2, 908.8, 858.2, 819.9, 782.0, 741.1, 697.5, 550.3

R$_f$: 0.17 (1:1 Hexane / EtOAc)

HRMS: Calc. [M+H]$^+$ for $C_{19}H_{18}N_2O_2$ = 307.1447 ; found = 307.1452

MP: 174.3 – 175.0 ˚C

Synthesis of **3c**

Following general procedure **C** and reacting BCB **1c** with indole **2a**, purification by flash chromatography (4:1 Hexanes / EtOAc) gave 31.9 mg of **3c** an orange solid in 52% yield.

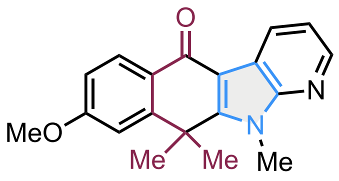

3c

^{1}H NMR (500 MHz, CDCl$_3$) δ 8.74 (dd, J = 7.7, 1.6 Hz, 1H), 8.41 (dd, J = 4.8, 1.7 Hz, 1H), 8.36 (d, J = 8.7 Hz, 1H), 7.30 – 7.25 (m, 1H), 7.12 (d, J = 2.4 Hz, 1H), 7.00 (dd, J = 8.7, 2.4 Hz, 1H), 4.20 (s, 3H), 3.93 (s, 3H), 1.91 (s, 6H).

^{13}C NMR (126 MHz, CDCl$_3$) δ 180.06, 162.93, 154.78, 151.49, 149.68, 144.05, 130.32, 128.57, 124.68, 118.64, 117.22, 112.14, 111.95, 109.23, 55.52, 37.51, 31.18, 28.33.

IR (KBr plate, cm^{-1}): 3059.8, 2982.6, 2938.4, 2836.1, 1639.5, 1602.6, 1452.7, 1405.0, 1368.4, 1303.9, 1269.0, 1238.4, 1198.3, 1131.0, 1077.6, 1036.4, 912.8, 885.7, 803.9, 774.8, 730.9, 694.7, 548.0

R$_f$: 0.32 (1:1 Hexane / EtOAc)

HRMS: Calc. [M+H]$^+$ for $C_{19}H_{18}N_2O_2$ = 307.1447 ; found = 307.1443

MP: 195.0 – 195.3 ˚C

Synthesis of **3d**

Following general procedure **C** and reacting BCB **1d** with indole **2a**, purification by flash chromatography (5:1 Hexanes / EtOAc) gave 43.0 mg of **3d** as a white solid in 74% yield.

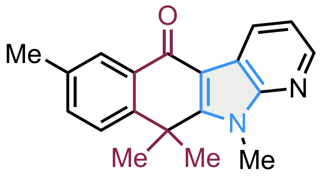

3d

^{1}H NMR (500 MHz, $CDCl_3$) δ 8.74 (dd, J = 7.7, 1.7 Hz, 1H), 8.41 (dd, J = 4.8, 1.7 Hz, 1H), 8.18 (s, 1H), 7.55 (d, J = 8.2 Hz, 1H), 7.44 (dd, J = 8.1, 2.4 Hz, 1H), 7.28 (dd, J = 7.7, 4.8 Hz, 1H), 4.20 (s, 3H), 2.45 (s, 3H), 1.89 (s, 6H).

^{13}C NMR (126 MHz, $CDCl_3$) δ 180.75, 155.32, 149.74, 146.46, 144.13, 136.65, 133.32, 130.78, 130.38, 126.37, 126.08, 118.70, 117.23, 109.45, 37.13, 31.19, 28.11, 20.96.

IR (KBr plate, cm^{-1}): 3054.7, 2980.5, 2927.9, 1645.6, 1606.9, 1579.2, 1523.1, 1451.4, 1403.2, 1386.0, 1368.0, 1304.7, 1258.8, 1238.0, 1161.4, 1132.9, 1085.1, 955.1, 928.2, 871.1, 832.6, 810.3, 777.5, 751.4, 685.1, 661.5, 581.6, 562.3, 547.8

R_f: 0.35 (1:1 Hexane / EtOAc)

HRMS: Calc. $[M+H]^+$ for $C_{19}H_{18}N_2O$ = 291.1497 ; found = 291.1496

MP: 219.0 – 219.7 ˚C

Synthesis of **3e**

Following general procedure **C** and reacting BCB **1e** with indole **2a**, purification by flash chromatography (5:1 Hexanes / EtOAc) gave 38.9 mg of **3e** as an off-white solid in 66% yield.

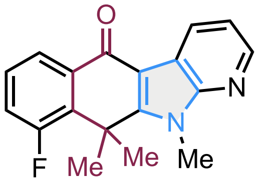

3e

^{1}H NMR (500 MHz, CDCl$_3$) δ 8.72 (dd, *J* = 7.7, 1.7 Hz, 1H), 8.43 (dd, *J* = 4.8, 1.7 Hz, 1H), 8.24 (d, *J* = 9.3 Hz, 1H), 7.46 (td, *J* = 7.9, 4.9 Hz, 1H), 7.35 – 7.27 (m, 2H), 4.20 (s, 3H), 2.01 (s, 6H).

^{13}C NMR (126 MHz, CDCl$_3$) δ 179.20, 179.18, 161.55, 159.57, 155.91, 149.82, 144.40, 135.65, 135.58, 133.23, 133.21, 130.35, 128.45, 128.38, 122.49, 122.47, 120.49, 120.29, 118.89, 117.00, 108.60, 36.58, 36.55, 31.41, 24.75, 24.70.

^{19}F NMR (470 MHz, CDCl$_3$) δ -108.43 (dd, *J* = 12.8, 4.7 Hz).

IR (KBr plate, cm^{-1}): 3010.1, 2993.4, 2945.9, 2849.8, 1636.3, 1604.0, 1581.7, 1477.1, 1434.3, 1405.1, 1368.1, 1305.0, 1248.3, 1225.0, 1198.2, 1124.3, 1097.8, 1061.7, 978.1, 919.0, 869.2, 850.3, 806.5, 755.0, 706.9, 557.7

R$_f$: 0.56 (1:1 Hexane / EtOAc)

HRMS: Calc. $[M+H]^+$ for $C_{18}H_{15}FN_2O$ = 295.1247 ; found = 295.1245

MP: 184.5 – 185.2 ˚C

Synthesis of **3f**

Following general procedure **C** and reacting BCB **1f** with indole **2a**, purification by flash chromatography (4:1 Hexanes / EtOAc) gave 39.3 mg of **3f** as an off-white solid in 67% yield.

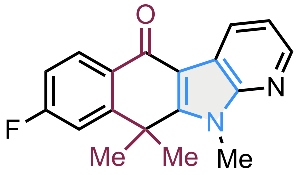

3f

^{1}H NMR (500 MHz, $CDCl_3$) δ 8.71 (d, *J* = 6.0 Hz, 1H), 8.43 – 8.35 (m, 2H), 7.34 – 7.25 (m, 2H), 7.14 (td, *J* = 8.3, 2.4 Hz, 1H), 4.19 (s, 3H), 1.90 (s, 6H).

^{13}C NMR (126 MHz, $CDCl_3$) δ 179.30, 166.46, 164.46, 154.73, 152.09, 152.02, 149.70, 144.34, 130.35, 129.21, 129.14, 127.60, 127.58, 118.84, 117.07, 114.76, 114.59, 112.99, 112.80, 109.17, 37.68, 37.66, 31.20, 28.14.

^{19}F NMR (470 MHz, $CDCl_3$) δ -106.36 – -106.43 (m).

IR (KBr plate, cm^{-1}): 3070.5, 3047.9, 3027.4, 2995.4, 2942.2, 2854.3, 1735.3, 1648.2, 1604.1, 1580.3, 1528.5, 1475.4, 1455.5, 1407.7, 1370.9, 1303.4, 1263.3, 1231.2, 1186.1, 1119.7, 1073.3, 922.5, 884.2, 802.0, 774.7, 690.1, 644.7, 554.2

R_f: 0.26 (2:1 Hexane / EtOAc)

HRMS: Calc. $[M+H]^+$ for $C_{18}H_{15}FN_2O$ = 295.1247 ; found = 295.1247

MP: 202.7 – 203.3 ˚C

Synthesis of **3g**

Following general procedure **C** and reacting BCB **1g** with indole **2a**, purification by flash chromatography (10:1 Hexanes / Acetone) gave 15.3 mg of **3g** as a white solid in 26% yield with ~87% purity.

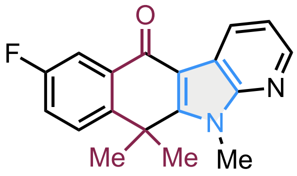

3g

^{1}H NMR (500 MHz, $CDCl_3$) δ 8.73 (d, *J* = 6.2 Hz, 1H), 8.44 (d, *J* = 4.8 Hz, 1H), 8.03 (dd, *J* = 9.2, 3.0 Hz, 1H), 7.66 (dd, *J* = 8.9, 4.8 Hz, 1H), 7.35 – 7.28 (m, 2H), 4.22 (s, 3H), 1.92 (s, 6H).

^{13}C NMR (126 MHz, $CDCl_3$) δ 179.22, 162.63, 160.67, 155.31, 149.79, 144.44, 130.41, 128.27, 128.21, 119.74, 119.56, 118.95, 118.66, 117.04, 112.22, 112.05, 109.30, 37.28, 31.27, 28.12.

^{19}F NMR (470 MHz, $CDCl_3$) δ -114.87 (td, *J* = 8.5, 4.9 Hz).

IR (KBr plate, cm^{-1}): 3059.7, 2978.2, 2925.0, 2850.1, 1706.0, 1645.7, 1610.9, 1585.5, 1489.7, 1453.1, 1404.2, 1369.1, 1257.4, 1215.7, 1149.2, 873.8, 832.8, 754.8, 666.1

R_f: 0.36 (1:1 Hexane / EtOAc)

HRMS: Calc. $[M+H]^+$ for $C_{19}H_{16}FN_2O$ = 295.1247 ; found = 295.1245

MP: 191.1 – 191.7 ˚C

Synthesis of **3h**

Following general procedure **C** and reacting BCB **1h** with indole **2a**, purification by flash chromatography (3:1 Hexanes / EtOAc) gave 16.3 mg of **3f** as an off-white solid in 28% yield.

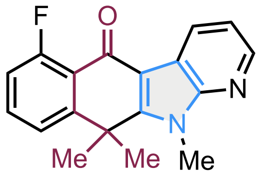

3h

^{1}H NMR (600 MHz, CDCl$_3$) δ 8.76 (dd, J = 7.7, 1.7 Hz, 1H), 8.42 (dd, J = 4.7, 1.6 Hz, 1H), 7.58 (td, J = 8.1, 5.1 Hz, 1H), 7.48 (d, J = 8.1 Hz, 1H), 7.29 (dd, J = 7.6, 4.7 Hz, 1H), 7.13 (dd, J = 11.6, 8.1 Hz, 1H), 4.19 (s, 3H), 1.93 (s, 6H).

^{13}C NMR (151 MHz, CDCl$_3$) δ 179.32, 163.23, 161.48, 153.47, 152.26, 149.72, 144.33, 133.16, 133.09, 130.57, 122.26, 122.23, 120.09, 120.06, 118.88, 117.24, 115.57, 115.42, 110.14, 37.58, 37.56, 31.17, 28.62.

^{19}F NMR (565 MHz, CDCl$_3$) δ -112.77 (dd, J = 11.6, 5.2 Hz).

IR (KBr plate, cm^{-1}): 3057.3, 2980.6, 2926.4, 2853.9, 1728.8, 1649.1, 1609.4, 1579.5, 1533.0, 1478.4, 1448.6, 1403.8, 1388.7, 1368.6, 1257.0, 1200.9, 1154.4, 1121.9, 1097.0, 971.5, 927.7, 864.4, 818.7, 783.8, 732.1, 695.5, 546.8

R_f: 0.26 (1:1 Hexane / EtOAc)

HRMS: Calc. $[M+Na]^+$ for $C_{18}H_{15}FN_2O$ = 317.1066 ; found = 317.1064

MP: 208.2 – 209.4 ˚C

Synthesis of **3i**

Following general procedure **C** and reacting BCB **1i** with indole **2a**, purification by flash chromatography (10:1 Hexanes / EtOAc) gave 53.0 mg of **3i** as an off-white solid in 79% yield.

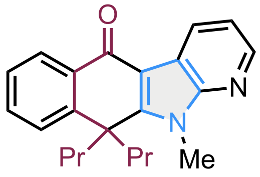

3i

^{1}H NMR (600 MHz, $CDCl_3$) δ 8.74 (dd, *J* = 7.6, 1.6 Hz, 1H), 8.42 (dd, *J* = 4.8, 1.7 Hz, 1H), 8.39 (d, *J* = 7.6 Hz, 1H), 7.62 (d, *J* = 3.4 Hz, 2H), 7.45 (dt, *J* = 8.1, 4.4 Hz, 1H), 7.28 (dd, *J* = 7.6, 4.7 Hz, 1H), 4.19 (s, 3H), 2.55 – 2.47 (m, 2H), 2.28 – 2.21 (m, 2H), 0.64 (s, 10H).

^{13}C NMR (151 MHz, $CDCl_3$) δ 181.00, 152.86, 149.65, 146.36, 144.10, 133.82, 132.49, 130.18, 126.78, 126.11, 124.92, 118.75, 117.17, 112.92, 47.28, 43.08, 31.06, 17.70, 14.05.

IR (KBr plate, cm^{-1}): 3059.6, 2958.5, 2871.8, 1643.9, 1599.8, 1579.7, 1524.1, 1485.1, 1460.0, 1431.2, 1398.0, 1376.9, 1346.4, 1305.3, 1269.2, 1217.5, 1196.8, 1180.8, 1123.6, 1101.1, 1033.9, 931.4, 886.7, 815.3, 792.1, 755.8, 705.5, 661.7, 589.7, 551.2, 498.3

R_f: 0.34 (1:1 Hexane / EtOAc)

HRMS: Calc. $[M+H]^+$ for $C_{22}H_{24}N_2O$ = 333.1967 ; found = 333.1967

MP: 176.9 – 177.8 ˚C

<u>Synthesis of **3j**</u>

Following general procedure **C** and reacting BCB **1j** with indole **2a**, purification by flash chromatography (8:1 Hexanes / Acetone) gave 51.9 mg of **3j** as a pale-yellow solid in 89% yield.

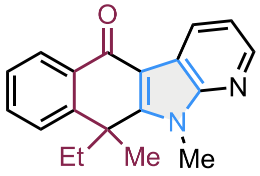

3j

^{1}H NMR (500 MHz, CDCl$_3$) δ 8.75 (dd, *J* = 7.7, 1.7 Hz, 1H), 8.42 (dd, *J* = 4.8, 1.6 Hz, 1H), 8.39 (d, *J* = 7.6 Hz, 1H), 7.65 – 7.60 (m, 2H), 7.46 (ddd, *J* = 8.1, 4.9, 3.3 Hz, 1H), 7.29 (dd, *J* = 7.7, 4.8 Hz, 1H), 4.19 (s, 3H), 2.62 (dq, *J* = 14.7, 7.3 Hz, 1H), 2.34 (dq, *J* = 14.8, 7.5 Hz, 1H), 1.91 (s, 3H), 0.34 (t, *J* = 7.4 Hz, 3H).

^{13}C NMR (126 MHz, CDCl$_3$) δ 180.82, 153.61, 149.73, 147.34, 144.16, 132.71, 132.43, 130.26, 126.89, 126.17, 125.52, 118.75, 117.18, 111.54, 42.64, 33.80, 31.06, 27.86, 9.21.

IR (KBr plate, cm^{-1}): 3059.8, 2968.2, 2933.0, 2873.4, 1644.0, 1600.2, 1523.4, 1485.2, 1463.6, 1431.2, 1399.8, 1285.1, 1220.6, 1190.7, 1159.2, 1121.9, 1093.5, 1033.3, 916.5, 883.8, 791.6, 764.8, 731.1, 694.5, 659.6, 551.1

R$_f$: 0.51 (2:1 Hexane / Acetone)

HRMS: Calc. $[M+H]^+$ for $C_{19}H_{18}N_2O$ = 291.1497 ; found = 291.1499

MP: 128.4 – 129.8 ˚C

Synthesis of **3k**

Following general procedure **C** and reacting BCB **1k** with indole **2a**, purification by flash chromatography (6:1 Hexanes / Acetone) gave 57.3 mg of **3k** as a white solid in 90% yield.

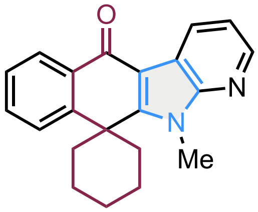

3k

^{1}H NMR (600 MHz, CDCl$_3$) δ 8.73 (dd, *J* = 7.7, 1.6 Hz, 1H), 8.45 (dd, *J* = 7.8, 1.7 Hz, 1H), 8.41 (dd, *J* = 4.8, 1.6 Hz, 1H), 8.12 (d, *J* = 8.1 Hz, 1H), 7.61 – 7.57 (m, 1H), 7.49 (t, *J* = 7.4 Hz, 1H), 7.28 (dd, *J* = 7.7, 4.8 Hz, 1H), 4.26 (s, 3H), 2.57 – 2.48 (m, 2H), 2.20 – 2.09 (m, 3H), 2.06 – 1.98 (m, 2H), 1.90 (dd, *J* = 15.3, 5.3 Hz, 2H), 1.75 (ddt, *J* = 14.4, 9.2, 4.3 Hz, 1H).

^{13}C NMR (151 MHz, CDCl$_3$) δ 180.45, 156.71, 150.74, 150.04, 144.20, 131.57, 130.28, 130.25, 126.96, 126.83, 126.82, 118.79, 117.11, 110.51, 38.45, 32.79, 32.11, 23.34, 21.67.

IR (KBr plate, cm^{-1}): 3060.6, 3006.5, 2933.1, 2875.6, 1643.7, 1601.0, 1581.7, 1521.9, 1484.0, 1460.3, 1430.9, 1399.9, 1370.8, 1355.4, 1305.9, 1260.8, 1216.2, 1194.2, 1174.3, 1122.2, 1091.3, 938.8, 911.8, 882.7, 791.7, 757.7, 719.9, 683.7, 666.4, 561.5

R$_f$: 0.44 (1:1 Hexane / EtOAc)

HRMS: calc. $[M+H]^+$ for $C_{21}H_{20}N_2O$ = 317.1654 ; found = 317.1648

MP: 184.6 – 185.9 ˚C

Synthesis of **3l**

Following general procedure **C** and reacting BCB **1l** with indole **2a**, purification by flash chromatography (8:1 Hexanes / Acetone) gave 46.4 mg of **3l** as a white solid in 73% yield.

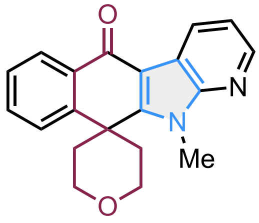

3l

^{1}H NMR (600 MHz, CDCl$_3$) δ 8.70 (dd, *J* = 7.7, 1.7 Hz, 1H), 8.42 (dt, *J* = 6.9, 1.6 Hz, 2H), 8.11 (d, *J* = 6.9 Hz, 1H), 7.64 (ddd, *J* = 8.1, 7.1, 1.7 Hz, 1H), 7.53 – 7.48 (m, 1H), 7.29 (dd, *J* = 7.6, 4.7 Hz, 1H), 4.31 (ddd, *J* = 12.3, 7.2, 2.2 Hz, 2H), 4.25 (s, 3H), 4.17 (td, *J* = 12.0, 4.3 Hz, 2H), 2.78 (ddd, *J* = 15.3, 11.8, 7.3 Hz, 2H), 1.92 (ddd, *J* = 15.4, 4.4, 2.1 Hz, 2H).

^{13}C NMR (151 MHz, CDCl$_3$) δ 179.99, 155.30, 150.59, 149.95, 144.45, 132.40, 130.30, 130.24, 127.20, 126.87, 126.42, 118.90, 116.99, 110.15, 64.25, 35.07, 32.84, 31.47.

IR (KBr plate, cm^{-1}): 3061.8, 3008.5, 2965.5, 2872.1, 1644.2, 1600.7, 1581.1, 1525.0, 1478.9, 1459.7, 1432.2, 1401.9, 1357.2, 1299.6, 1241.8, 1217.3, 1198.8, 1113.0, 1089.9, 1033.2, 957.9, 894.5, 877.9, 791.4, 756.9, 721.4, 685.0, 549.1, 520.7

R$_f$: 0.19 (1:1 Hexane / EtOAc)

HRMS: Calc. $[M+H]^+$ for $C_{20}H_{18}N_2O_2$ = 319.1447 ; found = 319.1442

MP: 179.2 – 180.5 ˚C

Synthesis of **3m**

Following general procedure **C** and reacting BCB **1a** with indole **2b**, purification by flash chromatography (10:1 Hexane / EtOAc) gave 56.3 mg of **3m** as a pale yellow solid in 79% yield.

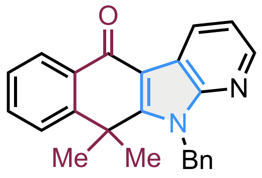

3m

^{1}H NMR (500 MHz, $CDCl_3$) δ 8.83 (dd, *J* = 7.7, 1.7 Hz, 1H), 8.43 – 8.40 (m, 1H), 8.38 (dd, *J* = 4.7, 1.7 Hz, 1H), 7.61 (dd, *J* = 5.0, 1.2 Hz, 2H), 7.46 (ddd, *J* = 8.1, 5.0, 3.2 Hz, 1H), 7.31 (dd, *J* = 7.8, 4.8 Hz, 1H), 7.27 – 7.17 (m, 3H), 6.87 (d, *J* = 6.8 Hz, 2H), 5.99 (s, 2H), 1.76 (s, 6H).

^{13}C NMR (126 MHz, $CDCl_3$) δ 180.74, 155.21, 149.76, 149.49, 144.89, 137.29, 132.43, 130.89, 130.55, 128.80, 127.31, 126.92, 126.30, 126.13, 125.43, 119.07, 117.12, 110.10, 47.38, 37.67, 29.05.

IR (KBr plate, cm^{-1}): 3061.4, 3028.5, 2979.0, 2926.6, 2852.5, 1643.8, 1599.9, 1486.2, 1466.7, 1454.3, 1433.7, 1416.7, 1257.3, 1183.7, 1087.8, 1036.1, 880.0, 792.4, 761.4, 695.9, 631.5, 560.5

R_f: 0.50 (2:1 Hexane / EtOAc)

HRMS: Calc. $[M+H]^+$ for $C_{24}H_{20}N_2O$ = 353.1654 ; found = 353.1653

MP: 155.3 – 155.9 ˚C

Synthesis of **3n**

Following general procedure **C** and reacting BCB **1a** with indole **2c**, purification by flash chromatography (6:1 Hexanes / EtOAc) gave 44.5 mg of **3n** as a tan solid in 80% yield.

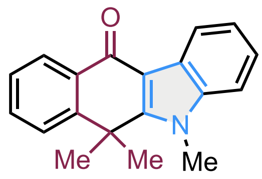

3n

^{1}H NMR (500 MHz, CDCl$_3$) δ 8.61 – 8.56 (m, 1H), 8.42 (dd, *J* = 7.8, 1.5 Hz, 1H), 7.65 (d, *J* = 6.6 Hz, 1H), 7.61 (td, *J* = 7.5, 1.5 Hz, 1H), 7.46 (ddd, *J* = 8.2, 6.9, 1.4 Hz, 1H), 7.42 – 7.35 (m, 3H), 4.07 (s, 3H), 1.89 (s, 6H).

^{13}C NMR (126 MHz, CDCl$_3$) δ 180.63, 154.63, 149.33, 138.67, 132.02, 131.33, 126.79, 126.27, 126.09, 124.60, 123.68, 122.79, 122.44, 110.97, 109.18, 37.19, 32.79, 28.40.

IR (KBr plate, cm^{-1}): 3058.5, 2980.6, 2935.3, 1637.9, 1597.9, 1521.6, 1490.6, 1466.1, 1418.3, 1375.1, 1324.9, 1236.2, 1124.8, 1085.4, 1037.0, 880.0, 749.2, 693.3

R$_f$: 0.54 (1:1 Hexane / EtOAc)

HRMS: Calc. $[M+Na]^+$ for $C_{19}H_{17}NO$ = 298.1208 ; found = 298.1207

MP: 68.5 – 70.1 ˚C

<u>Synthesis of **3o**</u>

Following general procedure **C** and reacting BCB **1a** with indole **2d**, purification by flash chromatography (4:1 Hexanes / EtOAc) gave 28.7 mg of **3o** as a pale-yellow oil in 50% yield.

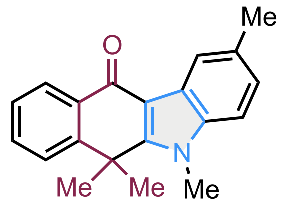

3o

^{1}H NMR (600 MHz, CDCl$_3$) δ 8.42 (d, *J* = 7.8 Hz, 1H), 8.40 (s, 1H), 7.64 (d, *J* = 8.1 Hz, 1H), 7.63 – 7.59 (m, 1H), 7.46 (t, *J* = 6.6 Hz, 1H), 7.28 (d, *J* = 8.2 Hz, 1H), 7.19 (d, *J* = 8.4 Hz, 1H), 4.05 (s, 3H), 2.53 (s, 3H), 1.89 (s, 6H).

^{13}C NMR (151 MHz, CDCl$_3$) δ 180.59, 154.59, 149.29, 137.03, 132.48, 131.92, 131.42, 126.76, 126.28, 126.04, 125.05, 124.77, 122.28, 110.62, 108.81, 37.19, 32.80, 28.41, 21.45.

IR (KBr plate, cm^{-1}): 3060.0, 3027.9, 2980.2, 2922.8, 2860.9, 1639.3, 1597.8, 1519.7, 1472.0, 1410.1, 1374.6, 1236.9, 1086.5, 1036.4, 918.8, 796.1, 766.2, 731.5, 692.7, 682.7, 595.4

R$_f$: 0.45 (2:1 Hexane / EtOAc)

HRMS: Calc. [M+Na]$^+$ for $C_{20}H_{19}NO$ = 312.1364; found = 312.1361

Synthesis of **3p**

Following general procedure **C** and reacting BCB **1a** with indole **2e**, purification by flash chromatography (6:1 Hexanes / EtOAc) gave 42.0 mg of **3p** as a brown solid in 68% yield.

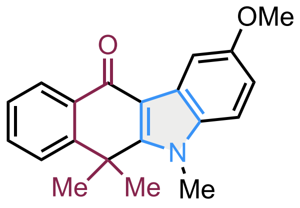

3p

^{1}H NMR (600 MHz, CDCl$_3$) δ 8.40 (dd, J = 7.8, 1.5 Hz, 1H), 8.09 (d, J = 2.5 Hz, 1H), 7.65 (d, J = 6.6 Hz, 1H), 7.61 (td, J = 7.5, 1.5 Hz, 1H), 7.48 – 7.43 (m, 1H), 7.28 (d, J = 8.8 Hz, 1H), 7.00 (dd, J = 8.8, 2.5 Hz, 1H), 4.05 (s, 3H), 3.95 (s, 3H), 1.89 (s, 6H).

^{13}C NMR (151 MHz, CDCl$_3$) δ 180.64, 156.59, 154.53, 149.28, 133.46, 131.99, 131.34, 126.81, 126.21, 126.06, 125.28, 113.88, 110.77, 110.01, 103.74, 55.94, 37.25, 32.87, 28.45.

IR (KBr plate, cm^{-1}): 3060.7, 2995.9, 2937.3, 2832.3, 1637.0, 1472.2, 1412.6, 1377.5, 1289.8, 1265.4, 1236.5, 1202.3, 1138.3, 1086.5, 1044.8, 1021.1, 863.6, 758.3, 693.1

R$_f$: 0.52 (1:1 Hexane / EtOAc)

HRMS: Calc. [M+H]$^+$ for $C_{20}H_{19}NO_2$ = 306.1494 ; found = 306.1492

MP: 178.9 – 179.9 ˚C

Synthesis of **3q**

Following general procedure **C** and reacting BCB **1a** with indole **2f**, purification by flash chromatography (5:1 Hexanes / EtOAc) gave 50.0 mg of **3q** as a tan solid in 65% yield.

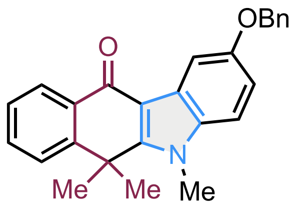

3q

^{1}H NMR (500 MHz, $CDCl_3$) δ 8.42 (dd, *J* = 7.7, 1.6 Hz, 1H), 8.23 (d, *J* = 2.6 Hz, 1H), 7.67 – 7.59 (m, 2H), 7.53 (d, *J* = 7.6 Hz, 2H), 7.49 – 7.44 (m, 1H), 7.41 (t, *J* = 7.6 Hz, 2H), 7.33 (t, *J* = 7.4 Hz, 1H), 7.28 (d, *J* = 8.9 Hz, 1H), 7.08 (dd, *J* = 8.9, 2.5 Hz, 1H), 5.23 (s, 2H), 4.03 (s, 3H), 1.88 (s, 6H).

^{13}C NMR (126 MHz, $CDCl_3$) δ 180.64, 155.74, 154.65, 149.30, 137.45, 133.66, 132.02, 131.33, 128.55, 127.85, 127.71, 126.81, 126.21, 126.10, 125.27, 114.45, 110.78, 110.08, 105.18, 70.68, 37.25, 32.87, 28.42.

IR (KBr plate, cm^{-1}): 3062.6, 3031.4, 2981.7, 2934.0, 2867.6, 1722.6, 1638.4, 1597.9, 1517.4, 1471.0, 1412.5, 1377.2, 1287.8, 1264.1, 1236.5, 1189.3, 1139.0, 1086.7, 1037.1, 1018.5, 909.7, 865.2, 766.9, 734.3, 693.9

R_f: 0.40 (2:1 Hexane / EtOAc)

HRMS: Calc. $[M+H]^+$ for $C_{26}H_{23}NO_2$ = 382.1807 ; found = 382.1806

MP: 188.8 – 189.4 ˚C

Synthesis of **3r**

Following general procedure **C** and reacting BCB **1a** with indole **2g**, purification by flash chromatography (5:1 Hexanes / EtOAc) gave 57.6 mg of **3r** as a white solid in 81% yield. Crystals suitable for analysis by X-Ray diffraction were obtained by slow evaporation of a solution of 4.7 mg of **3r** in 1.0 mL Et_2O and 0.3 mL acetone in a 4 mL vial with a septum cap pierced by a syringe needle.

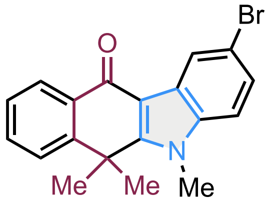

3r

^{1}H NMR (500 MHz, CDCl$_3$) δ 8.71 (d, *J* = 2.0 Hz, 1H), 8.38 (dd, *J* = 7.8, 1.1 Hz, 1H), 7.67 – 7.59 (m, 2H), 7.45 (ddd, *J* = 8.1, 6.6, 1.7 Hz, 1H), 7.42 (dd, *J* = 8.6, 2.0 Hz, 1H), 7.23 (d, *J* = 8.7 Hz, 1H), 4.05 (s, 3H), 1.88 (s, 6H).

^{13}C NMR (126 MHz, CDCl$_3$) δ 180.42, 155.20, 149.20, 137.38, 132.28, 131.03, 126.90, 126.52, 126.30, 126.11, 126.09, 124.94, 116.25, 110.64, 110.45, 37.25, 32.93, 28.35.

IR (KBr plate, cm^{-1}): 3062.7, 2982.0, 2936.4, 1639.2, 1598.7, 1521.9, 1478.3, 1452.7, 1407.7, 1369.1, 1243.4, 1234.8, 1085.9, 1035.7, 889.0, 795.0, 762.5, 691.9, 667.0, 592.7, 556.6

R$_f$: 0.48 (1:1 Hexane / EtOAc)

HRMS: Calc. [M+H]$^+$ for $C_{19}H_{16}BrNO$ = 354.0494 ; found = 354.0497

MP: 196.6 – 197.4 ˚C

Synthesis of **3s**

Following general procedure **C** and reacting BCB **1a** with indole **2h**, purification by flash chromatography (8:1 Hexanes / EtOAc) gave 46.2 mg of **3s** as a yellow solid in 54% yield.

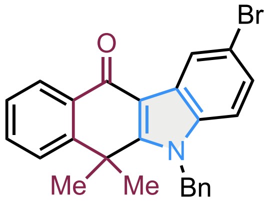

3s

^{1}H NMR (600 MHz, CDCl$_3$) δ 8.78 (d, *J* = 1.9 Hz, 1H), 8.43 (d, *J* = 7.8 Hz, 1H), 7.63 (d, *J* = 4.0 Hz, 2H), 7.48 (dq, *J* = 8.1, 4.3 Hz, 1H), 7.32 – 7.26 (m, 4H), 6.94 (dd, *J* = 9.7, 7.8 Hz, 3H), 5.77 (s, 2H), 1.82 (s, 6H).

^{13}C NMR (151 MHz, CDCl$_3$) δ 180.63, 155.28, 149.37, 136.79, 135.80, 132.36, 130.98, 129.10, 127.84, 126.96, 126.83, 126.41, 126.39, 126.09, 125.51, 125.13, 116.48, 112.01, 110.94, 49.21, 37.49, 29.03.

IR (KBr plate, cm^{-1}): 3088.7, 3064.0, 3030.7, 2981.1, 2929.0, 2870.7, 1640.7, 1600.0, 1522.2, 1479.9, 1449.3, 1419.2, 1370.6, 1356.2, 1244.5, 1184.1, 1087.2, 1036.6, 908.3, 888.7, 765.5, 732.1, 694.3, 665.4

R$_f$: 0.57 (2:1 Hexane / EtOAc)

HRMS: Calc. [M+H]$^+$ for $C_{25}H_{20}BrNO$ = 430.0807 ; found = 430.0805

MP: 246.6 – 247.5 ˚C

Synthesis of **3t**

Following general procedure **C** and reacting BCB **1a** with indole **2i**, purification by flash chromatography (10:1 Hexanes / EtOAc) gave 29.5 mg of **3t** as a yellow solid in 30% yield.

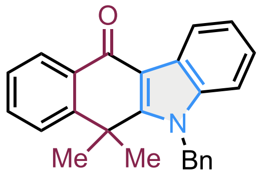

3t

^{1}H NMR (500 MHz, CDCl$_3$) δ 8.63 (dt, *J* = 7.9, 1.0 Hz, 1H), 8.47 – 8.43 (m, 1H), 7.65 – 7.59 (m, 2H), 7.47 (ddd, *J* = 8.1, 6.3, 2.0 Hz, 1H), 7.35 (ddd, *J* = 7.9, 7.1, 1.0 Hz, 1H), 7.31 – 7.20 (m, 5H), 7.10 (d, *J* = 8.3 Hz, 1H), 6.96 (d, *J* = 6.5 Hz, 2H), 5.81 (s, 2H), 1.83 (s, 6H).

^{13}C NMR (126 MHz, CDCl$_3$) δ 180.86, 154.68, 149.51, 138.10, 136.26, 132.11, 131.26, 129.00, 127.64, 126.84, 126.33, 126.07, 125.60, 124.86, 123.90, 122.92, 122.51, 111.45, 110.53, 49.08, 37.44, 29.08.

IR (KBr plate, cm^{-1}): 3062.5, 3031.2, 2980.1, 2929.9, 2870.2, 2246.0, 1639.6, 1598.9, 1522.2, 1490.5, 1463.8, 1428.6, 1393.8, 1381.3, 1348.0, 1253.5, 1185.5, 1087.1, 1037.7, 908.9, 878.4, 731.5, 694.2

R_f: 0.53 (2:1 Hexane / EtOAc)

HRMS: Calc. $[M+Na]^+$ for $C_{25}H_{21}NO$ = 374.1521 ; found = 374.1521

MP: 205.7 – 206.9 ˚C

Synthesis of **3u**

Following general procedure **C** and reacting BCB **1a** with indole **2j**, purification by flash chromatography (4:1 Hexanes / EtOAc) gave 38.2 mg of **3u** as a white solid in 54% yield.

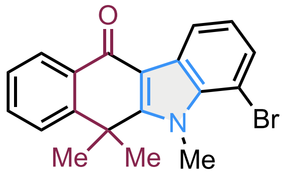

3u

^{1}H NMR (500 MHz, $CDCl_3$) δ 8.63 (d, J = 7.8 Hz, 1H), 8.38 (d, J = 7.8 Hz, 1H), 7.63 (d, J = 9.7 Hz, 2H), 7.53 – 7.41 (m, 2H), 7.15 (t, J = 7.8 Hz, 1H), 4.47 (s, 3H), 1.92 (s, 6H).

^{13}C NMR (126 MHz, $CDCl_3$) δ 180.47, 155.55, 149.87, 134.80, 132.34, 130.79, 129.67, 128.04, 126.84, 126.28, 126.19, 123.82, 121.78, 110.71, 103.66, 37.25, 36.27, 28.38.

IR (KBr plate, cm^{-1}): 3062.0, 2982.6, 2930.9, 1643.5, 1599.9, 1558.8, 1526.9, 1488.0, 1467.4, 1428.9, 1401.4, 1367.8, 1292.6, 1252.1, 1193.9, 1116.3, 1082.6, 1037.7, 899.4, 862.6, 780.0, 764.8, 738.2

R_f: 0.43 (4:1 Hexane / EtOAc)

HRMS: Calc. $[M+H]^+$ for $C_{19}H_{16}BrNO$ = 354.0494 ; found = 354.0496

MP: 198.6 – 199.9 ˚C

Synthesis of **3v**

Following general procedure **C** and reacting BCB **1a** with indole **2k**, purification by flash chromatography (4:1 Hexanes / EtOAc) gave 49.9 mg of **3v** as a viscous tan oil in 86% yield.

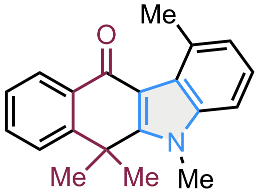

3v

^{1}H NMR (500 MHz, $CDCl_3$) δ 8.43 – 8.38 (m, 1H), 7.61 (d, *J* = 6.4 Hz, 2H), 7.45 (ddd, *J* = 8.1, 6.4, 1.8 Hz, 1H), 7.27 (t, *J* = 7.7 Hz, 1H), 7.21 (d, *J* = 7.9 Hz, 1H), 7.14 (d, *J* = 7.1 Hz, 1H), 4.05 (s, 3H), 3.15 (s, 3H), 1.90 (s, 6H).

^{13}C NMR (126 MHz, $CDCl_3$) δ 179.63, 154.94, 148.69, 139.54, 134.17, 131.82, 131.78, 126.74, 125.72, 125.10, 123.96, 123.92, 112.43, 106.72, 36.97, 33.16, 28.72, 24.06.

IR (KBr plate, cm^{-1}): 3057.2, 2979.5, 2925.6, 1643.6, 1597.4, 1577.8, 1507.7, 1457.0, 1426.1, 1406.2, 1381.6, 1368.6, 1332.1, 1258.4, 1239.7, 1202.5, 1129.2, 1067.2, 1038.1, 1010.6, 960.9, 916.3, 863.9, 766.2, 748.2, 732.2, 693.0, 652.4

R_f: 0.52 (2:1 Hexanes / EtOAc)

HRMS: Calc. $[M+H]^+$ for $C_{20}H_{19}NO$ = 290.1545 ; found = 290.1544

Synthesis of **3w**

Following general procedure **C** and reacting BCB **1a** with indole **2l**, purification by flash chromatography (20:1 Hexanes / EtOAc) gave 28.4 mg of **3w** as a pale-yellow solid in 54% yield.

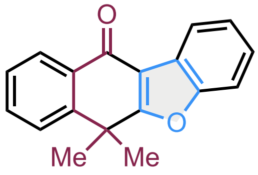

3w

^{1}H NMR (500 MHz, $CDCl_3$) δ 8.40 (d, *J* = 7.7 Hz, 1H), 8.32 – 8.28 (m, 1H), 7.67 (d, *J* = 4.5 Hz, 2H), 7.58 (dd, *J* = 6.2, 3.1 Hz, 1H), 7.53 – 7.48 (m, 1H), 7.44 – 7.38 (m, 2H), 1.81 (s, 7H).

^{13}C NMR (126 MHz, $CDCl_3$) δ 181.43, 173.94, 154.85, 147.53, 132.77, 131.45, 127.16, 126.85, 126.01, 125.39, 124.53, 124.05, 122.40, 113.81, 111.28, 37.87, 28.66.

IR (KBr plate, cm^{-1}): 3061.5, 2977.0, 2932.2, 2870.2, 1660.4, 1600.9, 1565.0, 1486.5, 1467.2, 1450.1, 1413.4, 1306.6, 1281.6, 1230.9, 1190.7, 1165.0, 1148.3, 1090.2, 1037.5, 1008.2, 976.0, 891.9, 874.8, 750.2, 694.2, 619.7, 552.4

R_f: 0.62 (4:1 Hexane / EtOAc)

HRMS: Calc. $[M+H]^+$ for $C_{18}H_{14}O_2$ = 263.1072 ; found = 263.1074

MP: 143.0 – 144.2 ˚C

Synthesis of **3x**

Following general procedure **C** and reacting BCB **1a** with indole **2m**, purification by flash chromatography (20:1 Hexanes / EtOAc) gave 31.5 mg of **3x** as a tan solid in 57% yield.

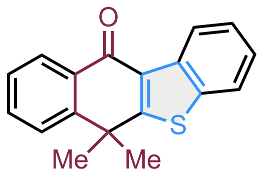

3x

^{1}H NMR (500 MHz, $CDCl_3$) δ 8.98 (d, *J* = 8.1 Hz, 1H), 8.44 (dd, *J* = 7.9, 1.5 Hz, 1H), 7.87 (d, *J* = 8.0 Hz, 1H), 7.69 – 7.62 (m, 2H), 7.56 – 7.48 (m, 2H), 7.46 – 7.41 (m, 1H), 1.85 (s, 6H).

^{13}C NMR (126 MHz, $CDCl_3$) δ 179.82, 169.16, 149.33, 137.56, 136.82, 132.48, 131.48, 127.89, 127.31, 127.13, 125.89, 125.72, 125.53, 121.86, 39.78, 34.35.

IR (KBr plate, cm^{-1}): 3062.1, 2968.8, 2926.9, 2860.0, 1648.0, 1599.5, 1526.0, 1465.2, 1435.8, 1383.0, 1261.2, 1223.5, 1140.9, 1058.5, 862.0, 832.1, 763.2, 735.5, 705.1, 669.6, 635.6

R_f: 0.64 (4:1 Hexane / EtOAc)

HRMS: Calc. $[M+H]^+$ for $C_{18}H_{14}OS$ = 279.0844 ; found = 279.0842

MP: 122.3 – 123.6 ˚C

Synthesis of **3y**

Following general procedure **C** and reacting BCB **1a** with thiophene **2n**, purification by flash chromatography (10:1 Hexanes / EtOAc) gave 5.4 mg of **3y** as a brown oil in 12% yield.

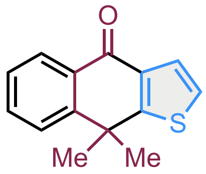

3y

^{1}H NMR (600 MHz, $CDCl_3$) δ 8.36 (d, *J* = 7.9 Hz, 1H), 7.66 – 7.61 (m, 2H), 7.59 (d, *J* = 5.3 Hz, 1H), 7.48 – 7.44 (m, 1H), 7.27 (d, *J* = 5.3 Hz, 1H), 1.80 (s, 6H).

^{13}C NMR (151 MHz, $CDCl_3$) δ 179.38, 165.21, 150.20, 135.04, 132.65, 130.89, 127.55, 126.97, 125.95, 125.06, 124.01, 39.33, 34.69.

IR (KBr plate, cm^{-1}): 3085.0, 3064.2, 2967.9, 2926.3, 2856.1, 1656.8, 1599.7, 1531.1, 1470.7, 1449.1, 1409.4, 1364.3, 1299.8, 1275.2, 1183.2, 1119.4, 1087.9, 1032.1, 919.9, 866.3, 811.2, 764.4, 696.8, 660.5, 631.8, 556.7

R_f: 0.36 (8:1 Hexane / EtOAc)

HRMS: Calc. $[M+H]^+$ for $C_{14}H_{12}OS$ = 229.0687 ; found = 229.0684

Product Derivatizations

Synthesis of **4**

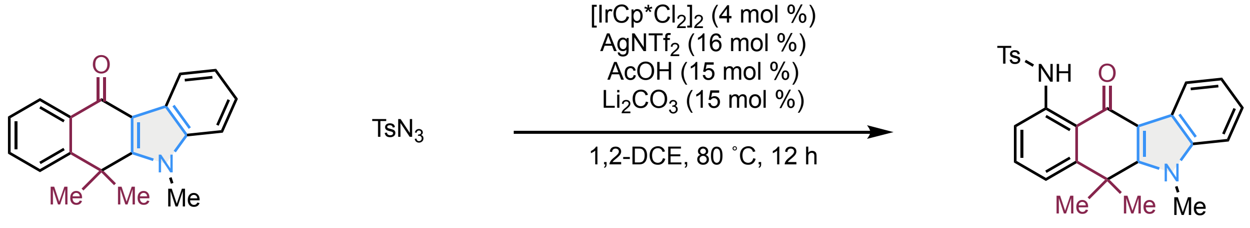

3n **4**

3n (27.5 mg, 0.1 mmol, 1 equiv.), TsN_3 (19.7 mg, 0.1 mmol, 1 equiv.), AcOH (0.9 mg, 0.015 mmol, 0.15 equiv.), and Li_2CO_3 (1.1 mg, 0.015 mmol, 0.15 equiv.) were added to a flame-dried 4 mL vial equipped with a stir bar. The vial was then transferred to an inert atmosphere glovebox. $[IrCp^*Cl_2]_2$ (3.2 mg, 0.004 mmol, 0.04 equiv.), $AgNTf_2$ (6.7 mg, 0.016 mmol, 0.16 equiv.), and 1,2-dichloroethane (0.5 mL) were added to the vial. The vial was sealed, transferred out of the glovebox, and the reaction mixture was heated to 80 ˚C with stirring for 12 hours. Upon completion, the reaction mixture was filtered through celite with EtOAc and concentrated under reduced pressure. Purification by flash chromatography (2:1 Hexane / Acetone) gave 42.2 mg of **4** as a pale yellow solid in 95% isolated yield.

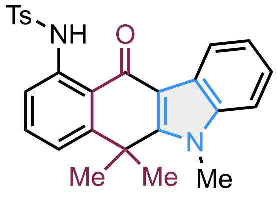

4

^{1}H NMR (500 MHz, CDCl$_3$) δ 13.73 (s, 1H), 8.54 – 8.50 (m, 1H), 7.85 (d, *J* = 8.4 Hz, 2H), 7.58 (dd, *J* = 8.3, 1.0 Hz, 1H), 7.42 (t, *J* = 8.1 Hz, 1H), 7.40 – 7.35 (m, 3H), 7.23 (dd, *J* = 8.0, 1.1 Hz, 1H), 7.20 (d, *J* = 7.8 Hz, 2H), 4.04 (s, 3H), 2.31 (s, 3H), 1.81 (s, 6H).

^{13}C NMR (126 MHz, CDCl$_3$) δ 184.80, 154.40, 151.80, 143.42, 141.10, 138.79, 137.27, 132.91, 129.57, 127.39, 124.35, 124.08, 123.21, 122.39, 120.62, 116.55, 115.99, 110.72, 109.40, 37.57, 32.83, 28.73, 21.51.

IR (KBr plate, cm^{-1}): 3046.6, 2982.0, 2922.0, 2866.0, 2851.4, 1612.2, 1597.3, 1462.7, 1365.9, 1318.4, 1224.4, 1196.3, 1160.1, 1091.2, 988.8, 876.8, 817.7, 733.0, 656.0, 560.0, 546.5, 506.4

R_f: 0.80 (1:1 Hexane / Acetone)

HRMS: Calc. $[M+H]^+$ for $C_{26}H_{24}N_2O_3S$ = 445.1586 ; found = 445.1584

MP: 222.3 – 222.8 ˚C

Synthesis of **5**

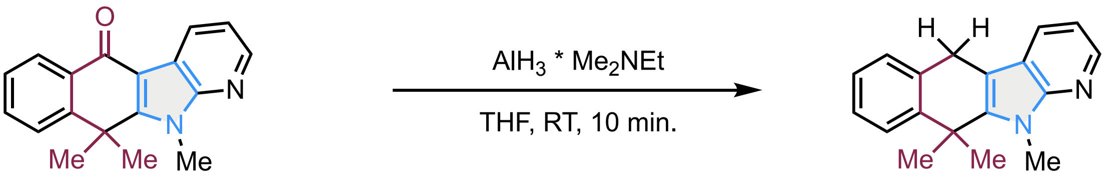

3a **5**

AlH_3 *N,N*-dimethylethylamine complex (0.5 M in THF, 0.3 mL, 0.15 mmol, 1.5 equiv.) was added *via* syringe to a solution of **3a** (27.6 mg, 0.1 mmol, 1 equiv.) in THF (5 mL) at room temperature under inert atmosphere. After 10 minutes, the reaction was quenched by the addition of 5 mL of 1:1 THF / water and was stirred for an additional 10 minutes. The solution was filtered through celite with THF and concentrated under reduced pressure. The crude mixture was dissolved in EtOAc and was washed with saturated aqueous Na_2CO_3 solution, dried over $MgSO_4$, and concentrated under reduced pressure. Purification by flash chromatography (8:1 Hexane / Acetone) gave 25.3 mg of **5** as a yellow solid in 90% isolated yield.

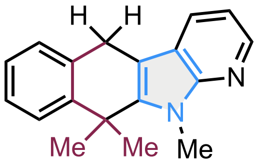

5

^{1}H NMR (500 MHz, $CDCl_3$) δ 8.33 (dd, *J* = 4.8, 1.6 Hz, 1H), 7.85 (dd, *J* = 7.7, 1.6 Hz, 1H), 7.61 (d, *J* = 8.1 Hz, 1H), 7.33 (t, *J* = 7.5 Hz, 2H), 7.24 (d, *J* = 7.3 Hz, 1H), 7.07 (dd, *J* = 7.6, 4.8 Hz, 1H), 4.16 (s, 2H), 4.11 (s, 3H), 1.84 (s, 6H).

^{13}C NMR (126 MHz, $CDCl_3$) δ 149.55, 144.72, 142.19, 140.29, 131.97, 129.08, 126.89, 126.52, 125.97, 125.92, 118.85, 115.26, 107.94, 103.95, 36.41, 30.85, 30.45, 26.37.

IR (KBr plate, cm^{-1}): 3054.7, 2977.4, 2934.5, 2866.7, 1466.1, 1442.6, 1402.8, 1307.4, 1264.0, 1230.8, 1151.8, 1083.1, 1039.8, 762.2, 752.4, 587.6, 554.3

R$_f$: 0.38 (4:1 Hexane / Acetone)

HRMS: Calc. $[M+H]^+$ for $C_{18}H_{18}N_2$ = 263.1548 ; found = 263.1547

MP: 128.2 – 129.4 ˚C

<u>Synthesis of **6**</u>

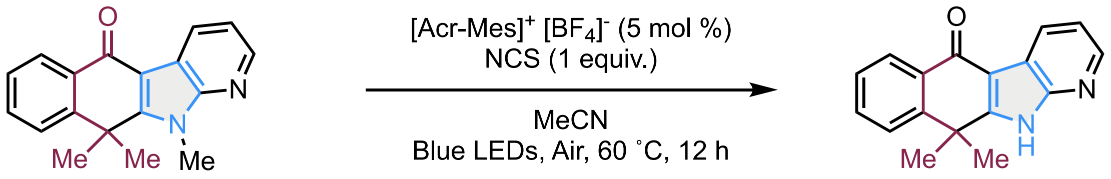

3a **6**

3a (27.6 mg, 0.1 mmol, 1 equiv.), [Acr-Mes][BF_4] (2.0 mg, 0.005 mmol, 0.05 equiv.), *N*-chlorosuccinimide (13.3 mg, 0.1 mmol, 1 equiv.), and MeCN were added to a vial equipped with a stir bar. The reaction vial was fitted with a septum cap and a balloon of air was connected *via* the septum. The reaction vial was placed in a glass oil bath with fresh, transparent silicon oil at 60 ˚C with stirring. A blue LED lamp was set up pointing directly at the oil bath and light was shined at the reaction vial through the oil bath. After 12 hours, the reaction was quenched by the addition of saturated aqueous $NaHCO_3$. This mixture was extracted three times with EtOAc, dried over Na_2SO_4, and concentrated under reduced pressure. Purification by flash chromatography (2:1 Hexane / EtOAc) gave 10.0 mg of azaindole **6** as a tan solid in 38% isolated yield.

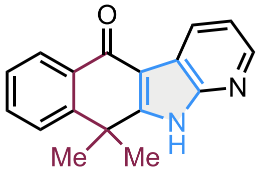

6

^{1}H NMR (500 MHz, CDCl$_3$) δ 13.45 (s, 1H), 8.82 (dd, *J* = 7.8, 1.7 Hz, 1H), 8.56 (dd, *J* = 5.0, 1.7 Hz, 1H), 8.46 (dd, *J* = 7.8, 1.6 Hz, 1H), 7.72 (dd, *J* = 8.0, 1.4 Hz, 1H), 7.68 (td, *J* = 7.5, 1.6 Hz, 1H), 7.52 (t, *J* = 8.1 Hz, 1H), 7.41 (dd, *J* = 7.7, 4.9 Hz, 1H), 1.98 (s, 6H).

^{13}C NMR (126 MHz, CDCl$_3$) δ 180.41, 157.65, 149.90, 147.77, 143.00, 132.28, 131.79, 131.36, 127.12, 126.76, 126.01, 118.75, 118.39, 109.33, 36.59, 30.65.

IR (KBr plate, cm^{-1}): 3360.0, 3067.1, 3025.7, 2962.3, 2925.3, 2850.7, 2789.0, 1645.0, 1594.4, 1483.8, 1470.6, 1457.8, 1411.5, 1286.2, 1268.4, 1209.1, 1181.5, 1081.6, 1036.1, 892.7, 756.3, 671.3, 631.0

R$_f$: 0.21 (1:1 Hexane / EtOAc)

HRMS: Calc. $[M+H]^+$ for $C_{17}H_{14}N_2O$ = 263.1184 ; found = 263.1184

MP: > 260 ˚C

Synthesis of **7**

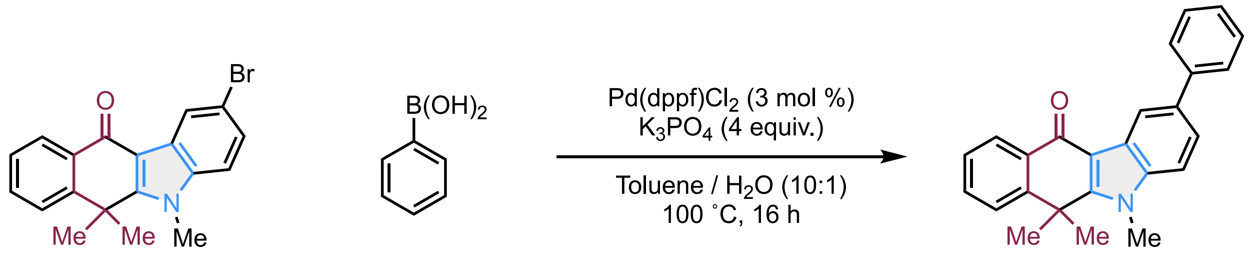

3r **7**

3r (24.5 mg, 0.07 mmol, 1 equiv.), phenylboronic acid (17.1 mg, 0.14 mmol, 2 equiv.), and Pd(dppf)Cl$_2$ (1.5 mg, 0.0021 mmol, 0.03 equiv.) were added to a flame-dried 4 mL vial equipped with a magnetic stir bar. The vial was then transferred to an inert atmosphere glovebox. K_3PO_4 (59.4 mg, 0.28 mmol, 4 equiv.), toluene (1.4 mL), and water (0.14 mL) were added to the vial. The vial was sealed, removed from the glovebox, and heated at 100 ˚C with stirring for 16 hours. The reaction was quenched by the addition of 1.0 M HCl. The aqueous phase was extracted three times with CH_2Cl_2. The combined organic phases were washed with 1.0 M HCl, dried over $MgSO_4$, and concentrated under reduced pressure. Purification by flash chromatography (4:1 Hexane / EtOAc) gave 22.3 mg of **47**as a white solid in 90% isolated yield.

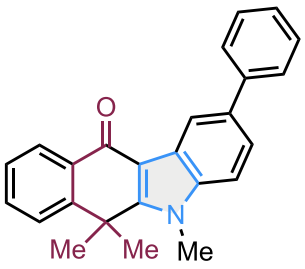

7

^{1}H NMR (500 MHz, CDCl$_3$) δ 8.85 (d, *J* = 1.8 Hz, 1H), 8.43 (dd, *J* = 7.8, 1.5 Hz, 1H), 7.75 (d, *J* = 7.3 Hz, 2H), 7.68 – 7.60 (m, 3H), 7.49 – 7.44 (m, 4H), 7.34 (t, *J* = 7.4 Hz, 1H), 4.10 (s, 3H), 1.92 (s, 6H).

^{13}C NMR (126 MHz, CDCl$_3$) δ 180.61, 155.14, 149.28, 141.78, 138.18, 136.31, 132.08, 131.32, 128.69, 127.55, 126.86, 126.74, 126.31, 126.08, 125.12, 123.25, 120.87, 111.22, 109.45, 37.28, 32.92, 28.40.

IR (KBr plate, cm^{-1}): 3061.3, 3027.9, 2982.3, 2926.3, 2853.9, 1737.8, 1637.8, 1598.0, 1521.4, 1471.6, 1442.5, 1409.4, 1373.6, 1236.0, 1204.5, 1086.2, 1033.0, 899.2, 764.3, 732.0, 694.5, 683.0

R$_f$: 0.42 (2:1 Hexane / EtOAc)

HRMS: Calc. [M+H]$^+$ for $C_{25}H_{21}NO$ = 352.1701 ; found = 352.1703

MP: 201.5 – 202.4 ˚C

Synthesis of **8**

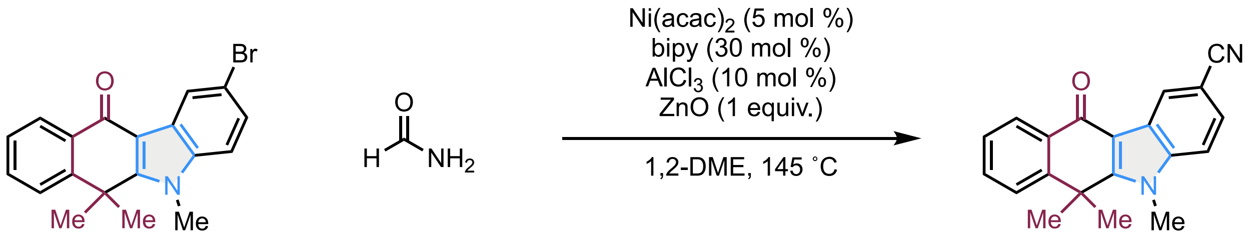

3r **8**

3r (24.5 mg, 0.07 mmol, 1 equiv.), Ni(acac)$_2$ (0.9 mg, 0.0035 mmol, 0.05 equiv.), 2,2'-bipyridine (3.3 mg, 0.021, 0.3 equiv.), ZnO (5.7 mg, 0.07 mmol, 1 equiv.), and formamide (0.35 mL) were added to a flame-dried 4 mL vial equipped with a magnetic stir bar. The vial was transferred to an inert atmosphere glovebox. Aluminum trichloride (0.9 mg, 0.007 mmol, 0.1 equiv.) was added to

the vial. The vial was sealed, removed from the glovebox, and heated at 145 ˚C with stirring for 20 hours. Upon completion, the reaction was quenched by the addition of brine. The aqueous phase was extracted three times with EtOAc. The combined organics were washed with brine, dried over $MgSO_4$, and concentrated under reduced pressure. Purification by flash chromatography (2:1 Hexane / EtOAc) gave 10.9 mg of **8** as a white solid in 52% isolated yield.

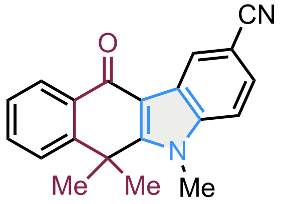

8

^{1}H NMR (500 MHz, $CDCl_3$) δ 8.90 (s, 1H), 8.38 (d, *J* = 7.8 Hz, 1H), 7.66 (d, *J* = 6.2 Hz, 2H), 7.59 (dd, *J* = 8.5, 1.6 Hz, 1H), 7.52 – 7.44 (m, 2H), 4.12 (s, 3H), 1.93 (s, 6H).

^{13}C NMR (126 MHz, $CDCl_3$) δ 156.33, 149.13, 140.28, 132.64, 130.75, 127.51, 127.10, 126.86, 126.43, 126.16, 120.05, 111.17, 110.14, 105.96, 37.37, 33.10, 28.34.

IR (KBr plate, cm^{-1}): 3063.4, 2981.5, 2924.8, 2853.4, 2222.4, 1642.8, 1599.0, 1481.4, 1409.4, 1369.6, 1237.1, 1035.0, 919.0, 810.4, 765.8, 729.4, 692.4, 681.9, 619.9

R_f: 0.16 (2:1 Hexane / EtOAc)

HRMS: Calc. $[M+H]^+$ for $C_{20}H_{16}N_2O$ = 301.1341 ; found = 301.1339

MP: 246.3 – 246.7 ˚C

Mechanistic Experiments

Synthesis of **2a'-THF**

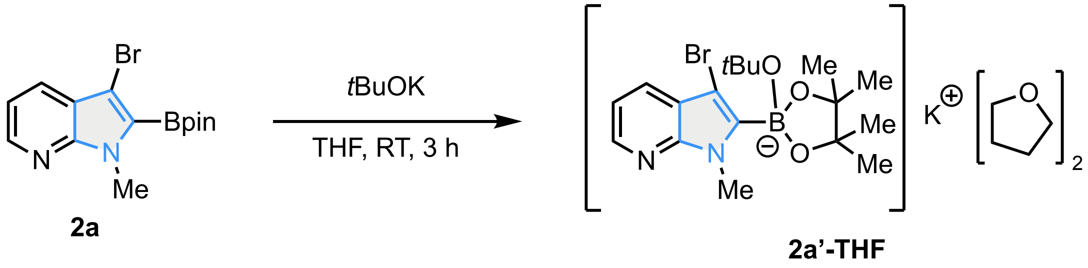

2a (1.0010 g, 3 mmol, 1 equiv.) was added to a flame-dried 40 mL vial equipped with a stir bar. The vial was then transferred into an inert atmosphere glovebox. *t*BuOK (336.6 mg, 3 mmol, 1 equiv.) and anhydrous THF (10 mL) were added. The vial was sealed and the reaction was stirred at room temperature in the glovebox for 3 hours. Upon completion, about half of the solvent was removed under vacuum. 10 mL of cold anhydrous pentane was added to precipitate the product. The mixture was filtered and the collected solids dried to give 0.8203 g of **2a'-THF** as a white solid in 46% yield.

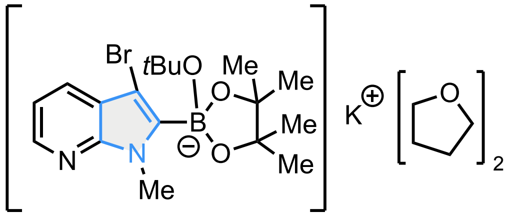

2a'-THF

^{1}H NMR (500 MHz, C_6D_6) δ 8.30 (d, *J* = 4.7 Hz, 1H), 7.87 (d, *J* = 7.7 Hz, 1H), 6.88 (dd, *J* = 7.7, 4.8 Hz, 1H), 4.57 (s, 3H), 3.54 (s, 8H, THF), 1.41 (q, *J* = 3.7 Hz, 8H, THF), 1.36 – 1.11 (m, 22H).

^{13}C NMR (126 MHz, C_6D_6) δ 149.31, 143.91, 140.74, 124.13, 121.35, 115.12, 68.24, 67.46 (THF), 32.42, 32.07, 31.52, 25.41 (THF).

^{11}B NMR (160 MHz, C_6D_6) δ 4.92.

Synthesis of **1a-2**

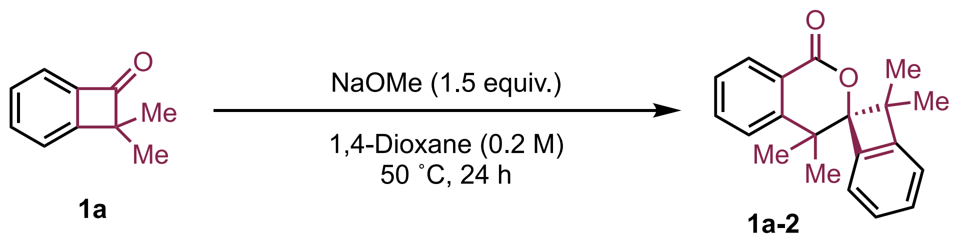

1a (73.0 mg, 0.5 mmol, 1 equiv.) was added to a flame-dried 8 mL vial equipped with a stir bar. The vial was transferred to an inert atmosphere glovebox. NaOMe (40.5 mg, 0.75 mmol, 1.5 equiv.) and 1,4-dioxane (2.5 mL) were added. The vial was sealed, removed from the glovebox, and heated at 50 ˚C with stirring for 24 hours. Upon completion, the crude mixture was filtered through a short silica plug with EtOAc and was concentrated under reduced pressure. Purification by preparative TLC (10:1 Pentane / EtOAc) gave 18.0 mg of dimer **1a-2** as a clear oil in 25% isolated yield.

Running this reaction at 0.1 mmol scale and using *t*BuOK (16.8 mg, 0.15 mmol, .5 equiv.) instead of NaOMe gave **1a-2** in 12% crude NMR yield using CH_2Br_2 as an internal standard.

Running this reaction at 0.1 mmol scale and using catalytic *t*BuOK (2.2 mg, 0.02 mmol, 0.2 equiv.) instead of NaOMe gave **1a-2** in 17% crude NMR yield using CH_2Br_2 as an internal standard with full conversion of **1a** as assessed by crude NMR.

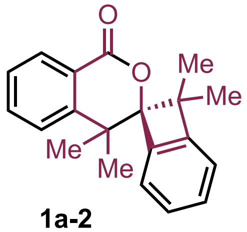

1a-2

^{1}H NMR (600 MHz, $CDCl_3$) δ 8.10 (d, *J* = 7.8 Hz, 1H), 7.60 (t, *J* = 7.7 Hz, 1H), 7.44 – 7.35 (m, 4H), 7.31 (t, *J* = 7.5 Hz, 1H), 7.10 (d, *J* = 7.2 Hz, 1H), 1.62 (s, 3H), 1.33 (s, 3H), 1.28 (s, 3H), 1.05 (s, 3H).

^{13}C NMR (151 MHz, $CDCl_3$) δ 165.67, 152.40, 148.06, 140.28, 134.16, 130.75, 130.10, 127.88, 127.20, 124.92, 123.43, 123.21, 120.25, 95.25, 56.62, 40.12, 30.45, 28.64, 24.08, 22.49.

IR (KBr plate, cm^{-1}): 3069.6, 2959.1, 2867.3, 1723.1, 1604.6, 1456.0, 1383.0, 1367.8, 1342.0, 1298.0, 1270.7, 1248.7, 1189.8, 1153.8, 1113.0, 1075.0, 1035.0, 990.8, 912.6, 760.3, 704.0, 674.2, 551.6.

R_f: 0.51 (4:1 Hexane / EtOAc)

HRMS: Calc. $[M+H]^+$ for $C_{20}H_{10}O_2$ = 293.1542 ; found = 293.1542

Procedure for Synthesis of 3n Using 3-halo-*N*-methylindoles

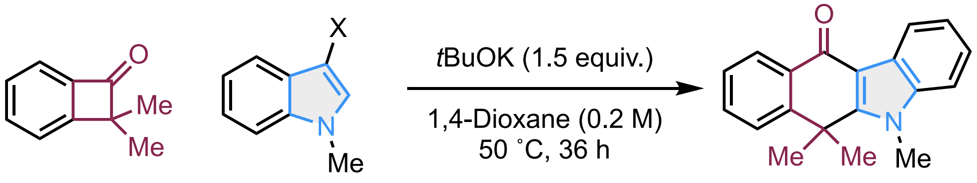

1a 1.5 equiv. **3n**

1a (14.6 mg, 0.1 mmol, 1 equiv.) and 3-bromo-*N*-methylindole[61] (0.15 mmol, 1.5 equiv.) were added to a flame-dried 4 mL vial equipped with a stir bar. The vial was transferred to an inert atmosphere glovebox. *t*BuOK (16.8 mg, 0.15 mmol, 1.5 equiv.) and 1,4-dioxane (0.5 mL) were added. The vial was sealed, removed from the glovebox, and heated at 50 ˚C with stirring for 24 hours. Upon completion, the crude mixture was filtered through a short silica plug with EtOAc and was concentrated under reduced pressure. Purification by flash chromatography (6:1 Hexane / EtOAc) gave 6.0 mg of **3n** in 21% isolated yield.

This procedure was repeated using the corresponding 3-chloro-,[62] 3-fluoro-,[63] and 3-iodo-*N*-methylindoles[64] (0.15 mmol, 1.5 equiv.). Yields of **3n** were determined by crude NMR analysis using CH_2Br_2 as an internal standard. Results are tabulated below.

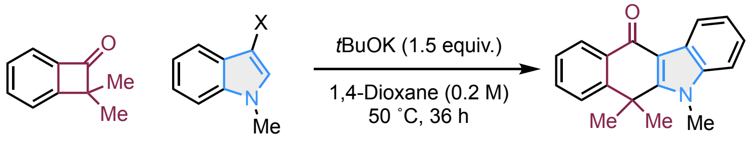

1a 1.5 equiv. **3n**

Entry	X =	Yield (NMR)
1	F	8%
2	Cl	20%
3	Br	21% (Isolated)
4	I	17%

Table S1. Reactions of **1a** with various 3-halo-*N*-methylindoles.

Synthesis of **3n’**

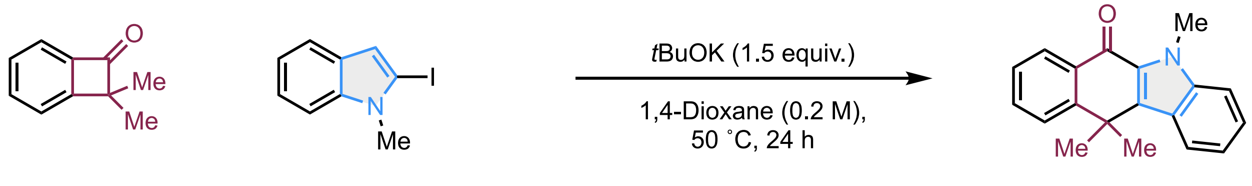

Following the aforementioned procedure and reacting BCB **1a** with 2-iodo-*N*-methylindole[65], purification by flash chromatography (2:1 Hexanes / EtOAc) gave 3.0 mg of **3n’** as a colorless oil in 11% yield.

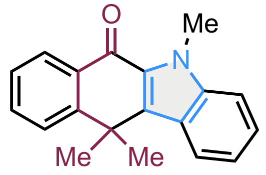

3n’

^{1}H NMR (500 MHz, CDCl$_3$) δ 8.40 (dd, *J* = 7.8, 1.5 Hz, 1H), 8.00 (d, *J* = 8.2 Hz, 1H), 7.75 (d, *J* = 6.6 Hz, 1H), 7.65 (td, *J* = 7.5, 1.5 Hz, 1H), 7.51 – 7.45 (m, 3H), 7.24 (ddd, *J* = 8.1, 6.2, 1.7 Hz, 1H), 4.29 (s, 3H), 1.89 (s, 6H).

^{13}C NMR (126 MHz, CDCl$_3$) δ 178.32, 151.82, 140.71, 134.26, 132.26, 132.09, 128.38, 126.64, 126.51, 126.43, 126.15, 123.42, 122.90, 119.95, 110.72, 37.42, 31.67, 30.80.

IR (KBr plate, cm^{-1}): 3062.4, 2971.5, 2928.7, 2855.2, 1737.1, 1645.1, 1610.7, 1599.5, 1530.2, 1485.2, 1463.4, 1432.4, 1371.4, 1288.5, 1266.4, 1242.3, 1154.9, 1085.9, 1019.6, 951.0, 804.6, 744.0, 718.0, 586.4

R_f: 0.88 (1:1 Hexane / EtOAc)

HRMS: Calc. $[M+H]^+$ for $C_{19}H_{17}NO$ = 276.1388 ; found = 276.1387

Procedures for control reactions testing indole substitution

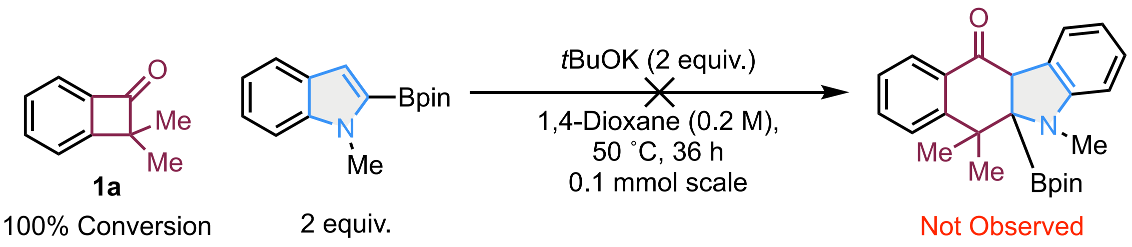

1a (14.6 mg, 0.1 mmol, 1 equiv.) and (1-methyl-2-(4,4,5,5-tetramethyl-1,3,2-dioxaborolan-2-yl)-1H-indole[66] (51.4 mg, 0.2 mmol, 2 equiv.) were added to a flame-dried 4 mL vial equipped with a stir bar. The vial was transferred to an inert atmosphere glovebox. *t*BuOK (16.8 mg, 0.15 mmol, 1.5 equiv.) and 1,4-dioxane (0.5 mL) were added. The vial was sealed, removed from the glovebox, and heated at 50 ˚C with stirring for 36 hours. Upon completion, the crude mixture was filtered through a short silica plug with EtOAc and was concentrated under reduced pressure. Crude GC/MS and crude NMR analysis using CH_2Br_2 as an internal standard indicated full consumption of **1a** and no desired [4+2] product.

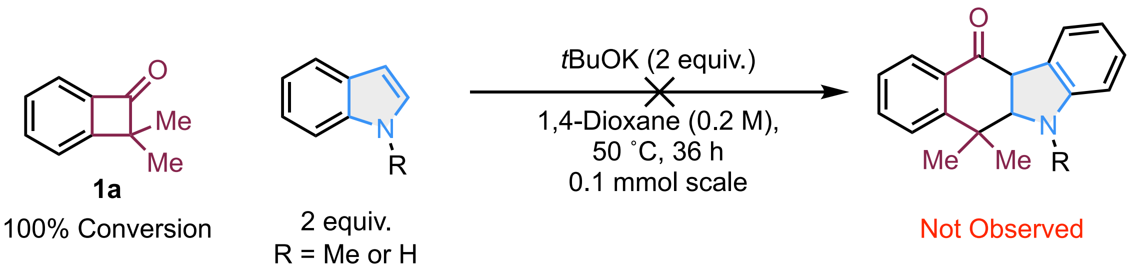

This process was repeated using either indole or *N*-methylindole (0.2 mmol, 2 equiv.) instead of the 2-borylindole above. Crude GC/MS and crude NMR analysis using CH_2Br_2 as an internal standard indicated full consumption of **1a** and no desired [4+2] product. The same result was obtained when the reaction using *N*-methylindole was carried out at 100 ˚C for 24 hours.

<u>Procedure for *in-situ* formation of *-ate* complex</u>

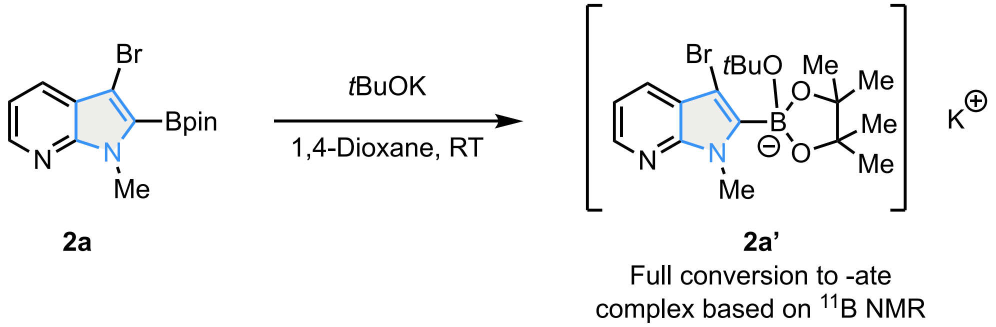

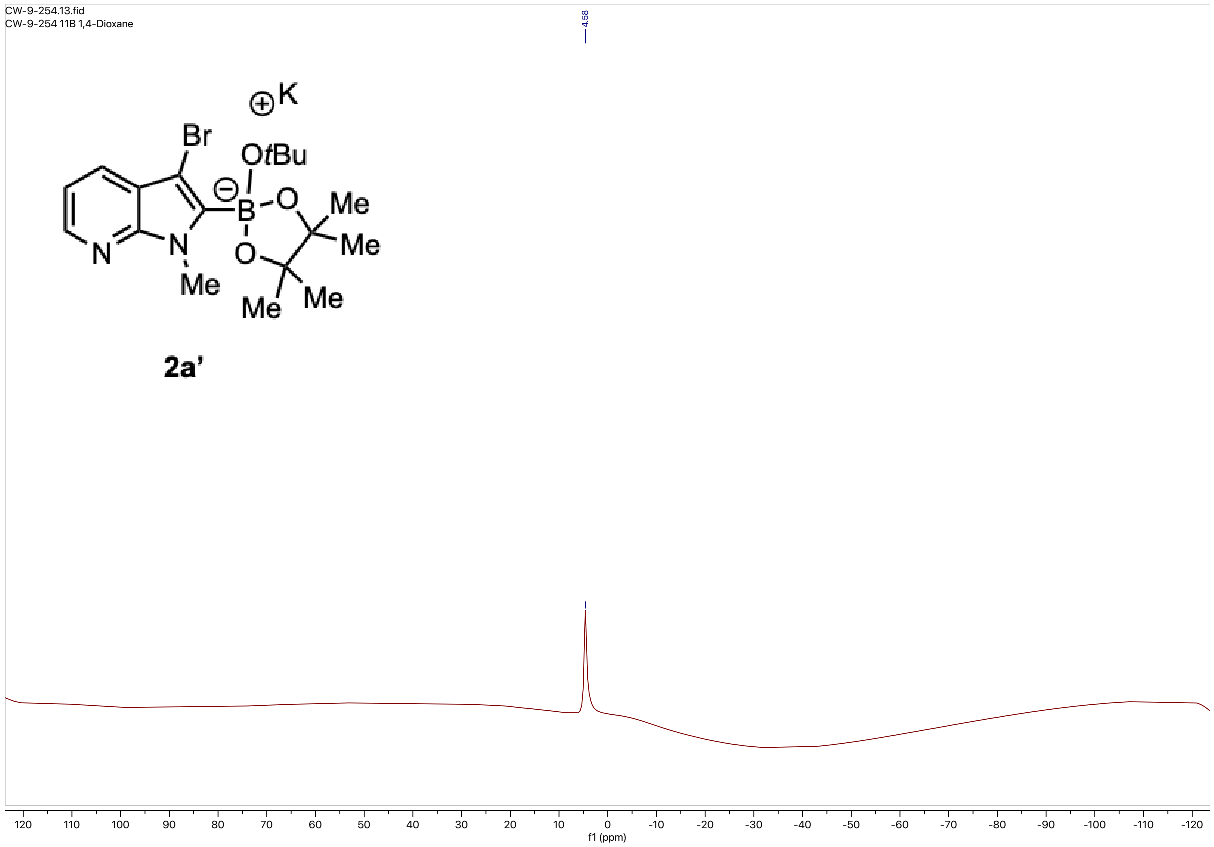

Figure S1. ^{11}B NMR spectrum in 1,4-Dioxane showing *in-situ* formation of -*ate* complex **2a'**.

2a (33.7 mg, 0.1 mmol, 1 equiv.) was added to a flame-dried 4 mL vial equipped with a stir bar. The vial was transferred to an inert atmosphere glovebox. *t*BuOK (16.8 mg, 0.15 mmol, 1.5 equiv.) and 1,4-dioxane (0.5 mL) were added. The vial was sealed and the reaction mixture was stirred at room temperature for 3 minutes to ensure homogeneity. The solution was then transferred to a NMR tube, sealed, and taken out of the glovebox. The mixture was analyzed by ^{11}B NMR without

solvent locking. The obtained ^{11}B NMR spectrum indicated only one signal at 4.58 ppm, indicating full conversion to proposed *-ate* complex **2a'** with no uncoordinated **2a** detected.

Procedure for stoichiometric reaction of 2a'-THF

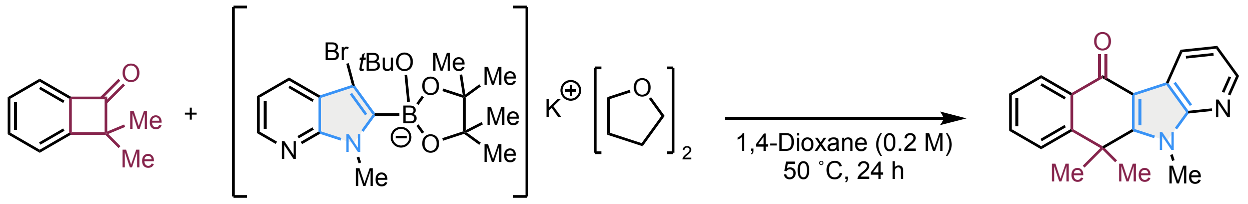

1a **2a'-THF** **3a**

99.3% Yield (NMR)

1a (14.6 mg, 0.1 mmol, 1 equiv.) was added to a flame-dried 4 mL vial equipped with a stir bar. The vial was transferred to an inert atmosphere glovebox. **2a'-THF** (89.0 mg, 0.15 mmol, 1.5 equiv.) and 1,4-dioxane (0.5 mL) were added. The vial was sealed, removed from the glovebox, and heated at 50 ˚C with stirring for 24 hours. Upon completion, the crude mixture was filtered through a short silica plug with EtOAc and was concentrated under reduced pressure. Crude NMR analysis using CH_2Br_2 as an internal standard indicated 99.3% yield of **3a**.

General Procedure for Kinetic Analyses

All kinetic data regarding the formation of **3a** were collected using GC/MS analysis with 1,3,5-trimethoxybenzene as an internal standard. A calibration curve was generated using samples with measured quantities of both **3a** and 1,3,5-trimethoxybenzene. Plotting the yield of **3a**, based on mmol of **3a** that would be produced in a 0.1 mmol scale reaction (i.e., the 40% yield mark on the y-axis would correspond to the sample with 0.04 mmol of **3a**), vs. the observed ratio of signal areas for **3a** and 1,3,5-trimethoxybenzene yielded a calibration curve. Linear regression of these data points yielded a R^2 value of 0.9944. Yields were calculated by taking the ratio of the signal areas of **3a** to 1,3,5-trimethoxybenzene in reaction samples and plugging them into the equation for the line of best fit from the calibration curve. Plots of the kinetic profiles of each reaction are included.

Determination of order for each reactant was carried out by comparing initial rates of formation of **3a** as a function of varying the initial concentration of the respective reactant while keeping the

standard concentrations of all other reactants (0.2 M for **1a**, 0.3 M for **2a**, and 0.3 M for *t*BuOK) and the same reaction volume of 1,4-dioxane (0.5 mL).

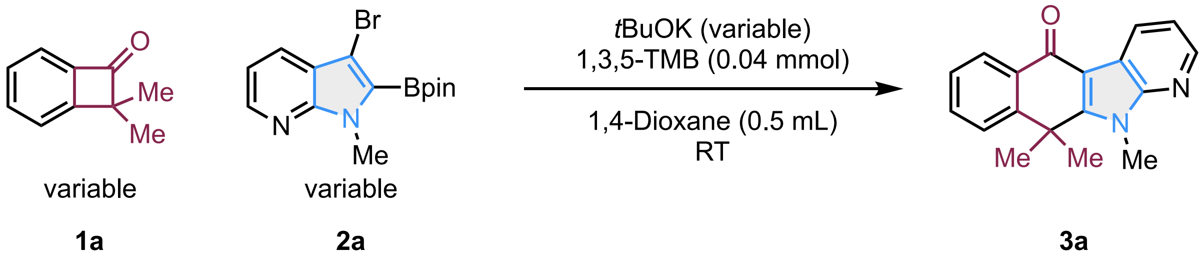

1a (variable), **2a** (variable), and 1,3,5-trimethoxybenzene (6.7 mg, 0.04 mmol, 0.4 equiv.) as internal standard were added to a flame-dried 4 mL vial equipped with a stir bar. The vial was transferred to an inert atmosphere glovebox. The vial was transferred to an inert atmosphere glovebox. *t*BuOK (variable) and 1,4-dioxane (0.5 mL) were added. Once homogenous, a 2 µL aliquot was taken and diluted to a volume of 2.0 mL with EtOAc. The solution was then filtered through a 0.2 µm syringe filter and analyzed by GC/MS. The reaction was stirred at room temperature in the glovebox and was sampled in the aforementioned manner at the indicated time points.

In the kinetic analysis of the reaction of **1a** with **2a'-THF**, **1a** (14.6 mg, 0.1 mmol, 1 equiv.) was added to a flame-dried 4 mL vial equipped with a stir bar. The vial was transferred to an inert atmosphere glovebox. **2a'-THF** (89.0 mg, 0.15 mmol, 1.5 equiv.) and 1,4-dioxane (0.5 mL) were added. Once homogenous, a 2 µL aliquot was taken and diluted to a volume of 2.0 mL with EtOAc. The solution was then filtered through a 0.2 µm syringe filter and analyzed by GC/MS. The reaction was stirred at room temperature in the glovebox and was sampled in the aforementioned manner at the indicated time points.

**1a** (0.2 M, Standard Condition)	
Time (min.)	**3a** Yield
0	1.13%
10	1.70%
30	2.35%
60	3.34%
90	4.76%
120	6.35%
180	9.01%
240	11.86%

Table S2. Initial rate data for standard conditions (0.2 M **1a**, 0.3 M **2a**, 0.3 M *t*BuOK).

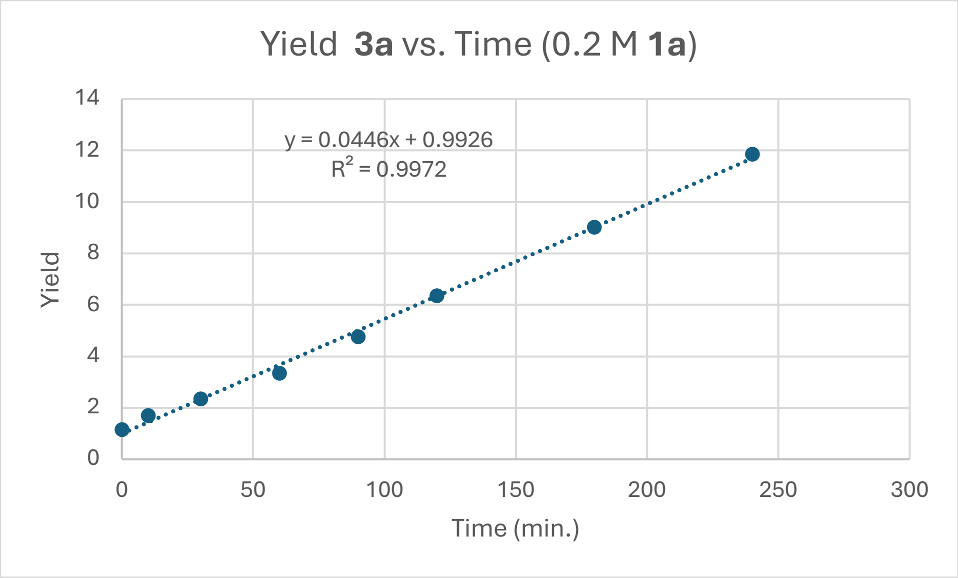

Figure S2. Initial rate profile for standard conditions (0.2 M **1a**, 0.3 M **2a**, 0.3 M *t*BuOK).

**1a** (0.3 M)	
Time (min.)	Product Yield
0	1.84%
10	4.79%
30	6.74%
60	9.59%
90	12.43%
120	14.61%
180	18.54%
240	22.15%

Table S3. Initial rate data for varying initial **1a** concentration to 0.3 M.

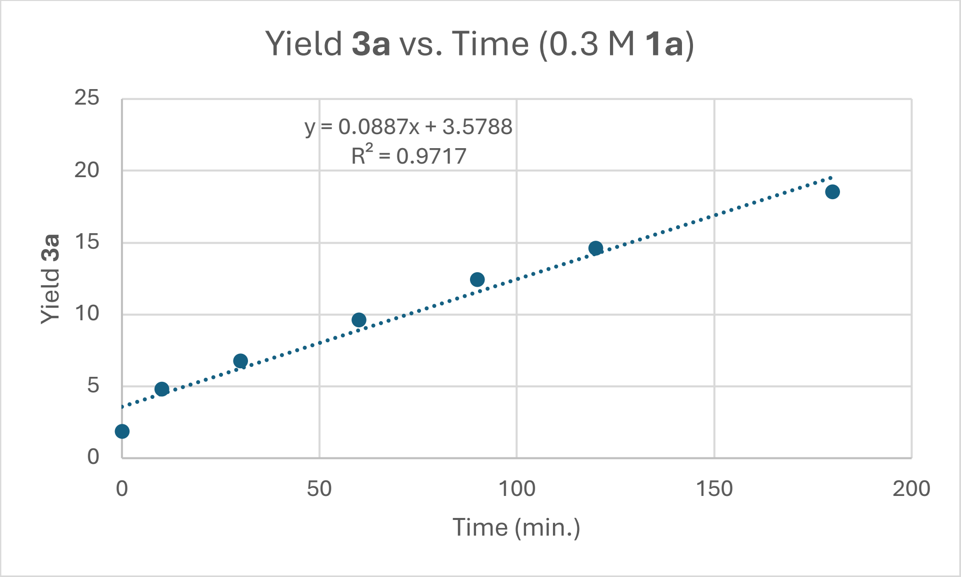

Figure S3. Initial rate profile varying initial **1a** concentration to 0.3 M.

**1a** (0.4 M)	
Time (min.)	**3a** Yield
0	0.60%
5	1.34%
10	1.90%
20	2.62%
30	3.77%
60	6.69%
90	9.56%
120	12.31%
180	17.11%

Table S4. Initial rate data for varying initial **1a** concentration to 0.4 M.

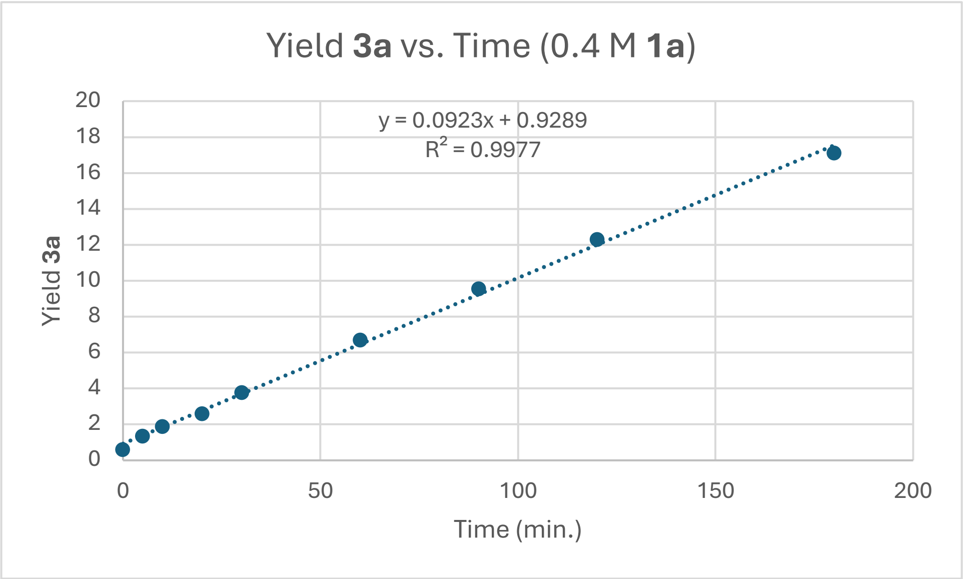

Figure S4. Initial rate profile varying initial **1a** concentration to 0.4 M.

**1a** (0.1 M)	
Time (min.)	**3a** Yield
0	0.71%
30	1.68%
60	3.08%
90	4.56%
120	6.06%
180	8.44%
240	10.42%

Table S5. Initial rate data for varying initial **1a** concentration to 0.1 M.

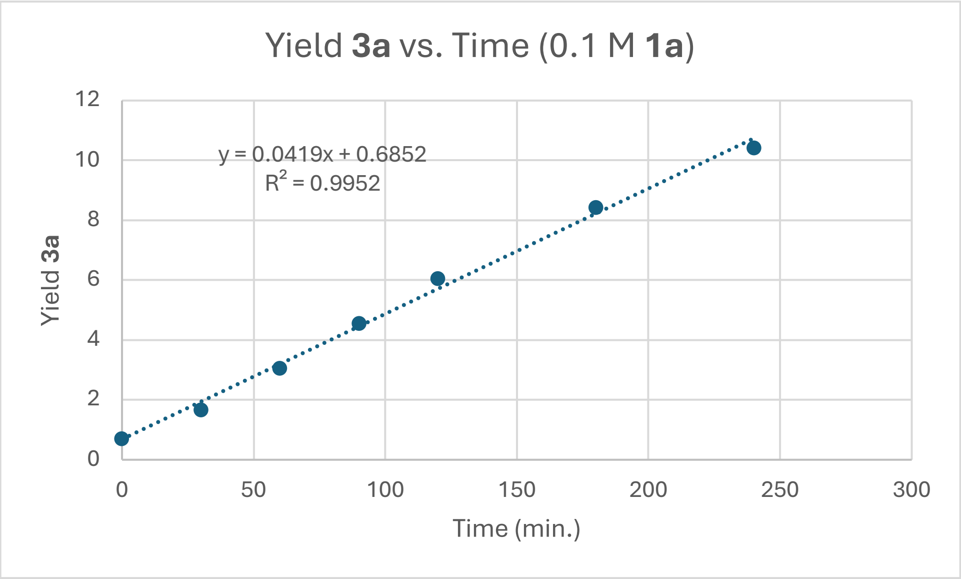

Figure S5. Initial rate profile varying initial **1a** concentration to 0.1 M.

**1a** (0.05 M)	
Time (min.)	**3a** Yield
0	0%
30	0.54%
60	1.11%
90	1.86%
120	2.61%
180	3.92%
240	5.08%
300	6.26%
360	7.07%

Table S6. Initial rate data for varying initial **1a** concentration to 0.05 M.

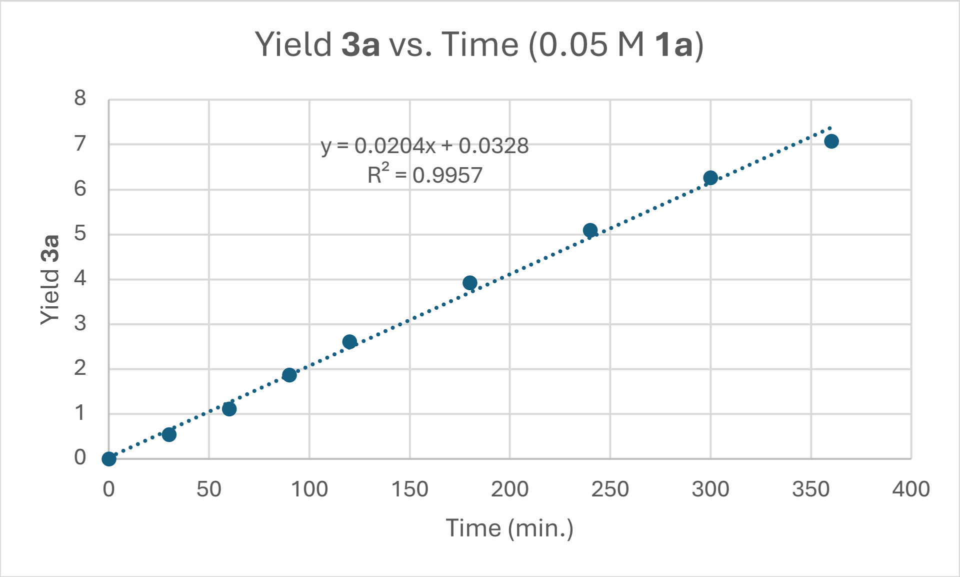

Figure S6. Initial rate profile varying initial **1a** concentration to 0.05 M.

**1a** Kinetic Order			
[**1a**] (M)	*k*	ln([**1a**])	ln(*k*)
0.05	0.0204	-2.96	-3.89
0.10	0.0419	-2.29	-3.17
0.21	0.0446	-1.55	-3.11
0.30	0.0887	-1.21	-2.42
0.42	0.0923	-0.86	-2.38

Table S7. Data for determination of kinetic order in **1a**.

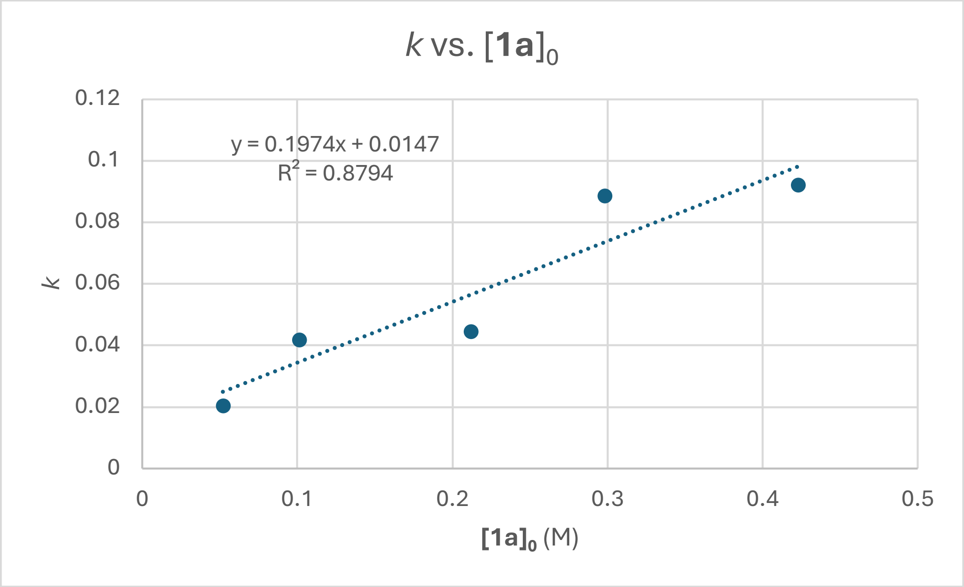

Figure S7. Plot of initial rate constant k vs. [**1a**]$_0$.

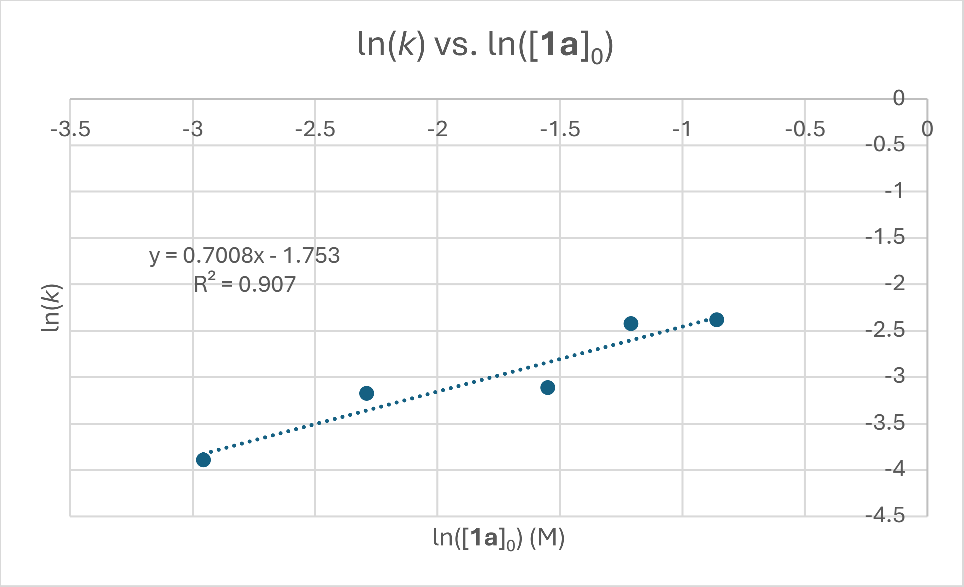

Figure S8. Plot of ln(k) vs. ln([**1a**]$_0$).

**2a** (0.25 M)	
Time (min.)	**3a** Yield
0	2.23%
10	4.09%
30	6.11%
60	8.20%
90	10.27%
120	12.20%
180	16.11%
240	18.78%

Table S8. Initial rate data for varying initial **2a** concentration to 0.25 M.

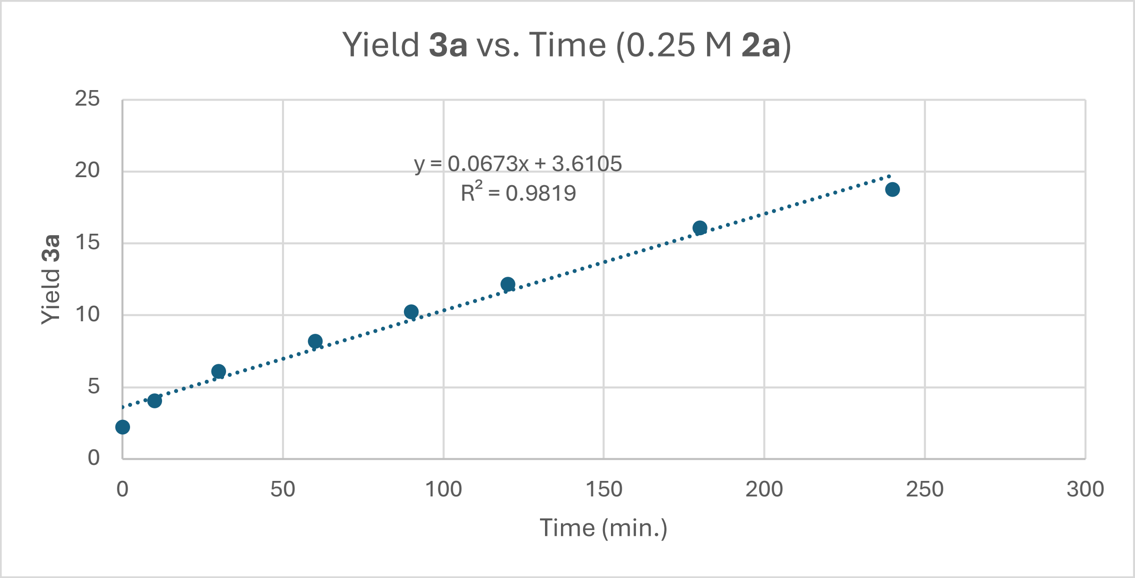

Figure S9. Initial rate profile varying initial **2a** concentration to 0.25 M.

**2a** (0.2 M)	
Time (min.)	**3a** Yield
0	3.00%
10	5.48%
30	7.37%
60	9.24%
90	10.77%
120	12.11%
180	14.66%
240	17.30%

Table S9. Initial rate data for varying initial **2a** concentration to 0.2 M.

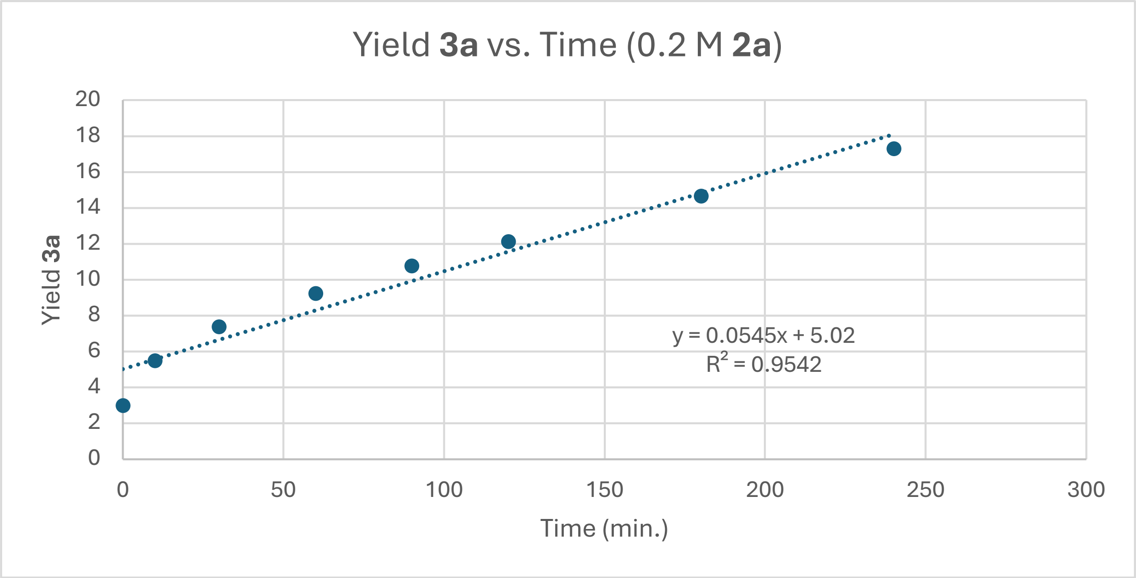

Figure S10. Initial rate profile varying initial **2a** concentration to 0.2 M.

**2a** (0.1 M)	
Time (min.)	**3a** Yield
0	2.66%
5	3.71%
10	4.39%
20	4.91%
30	5.25%
60	5.69%
90	5.68%
120	7.06%

Table S10. Initial rate data for varying initial **2a** concentration to 0.1 M.

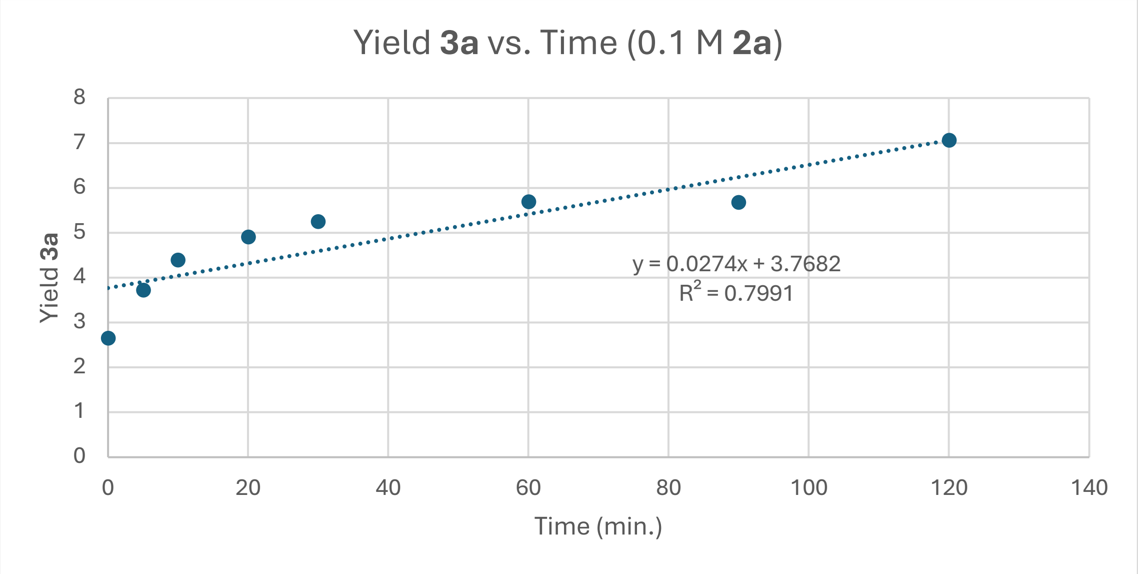

Figure S11. Initial rate profile varying initial **2a** concentration to 0.1 M.

**2a** (0.45 M)	
Time (min.)	**3a** Yield
0	0.37%
10	0.42%
30	0.45%
60	0.47%
90	0.54%
120	0.58%
180	0.65%
240	0.76%

Table S11. Initial rate data for varying initial **2a** concentration to 0.5 M. Ratio of **2a** to *t*BuOK is greater than 1. This presumably causes formation of -*ate* complex to be highly favored and thus not leaving unbound base available to react with **1a**, effectively shutting down the reaction.

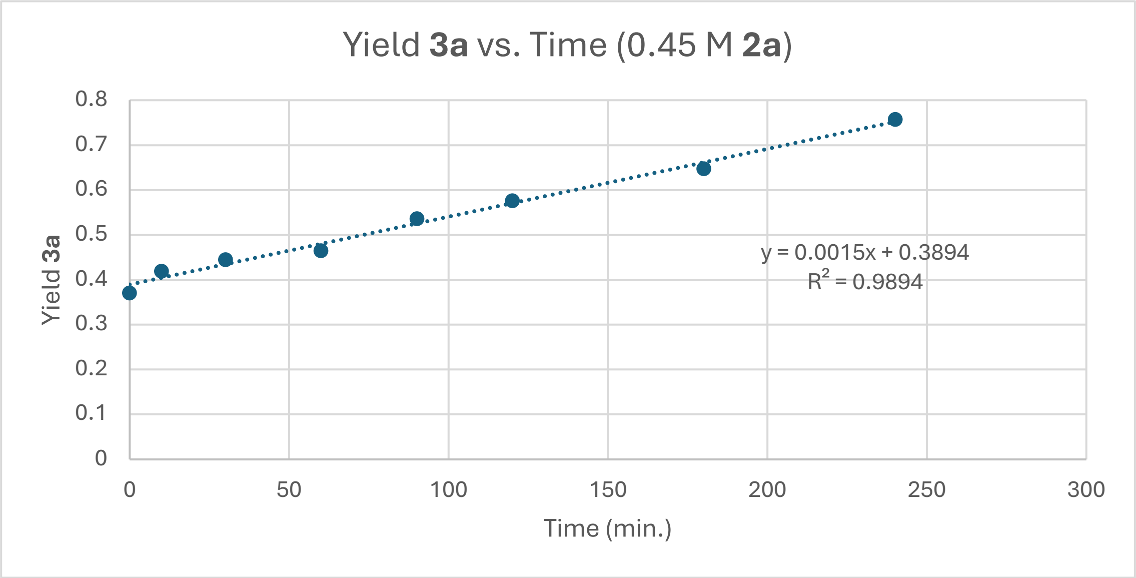

Figure S12. Initial rate profile varying initial **2a** concentration to 0.5 M.

2a (0.35 M)	
Time (min.)	**3a** Yield
0	0.73%
30	0.76%
60	0.87%
90	0.86%
120	1.01%
180	0.95%
240	1.13%
300	1.33%

Table S12. Initial rate data for varying initial **2a** concentration to 0.35 M. Ratio of **2a** to *t*BuOK is greater than 1. This presumably causes formation of -*ate* complex to be highly favored and thus not leaving unbound base available to react with **1a**, effectively shutting down the reaction.

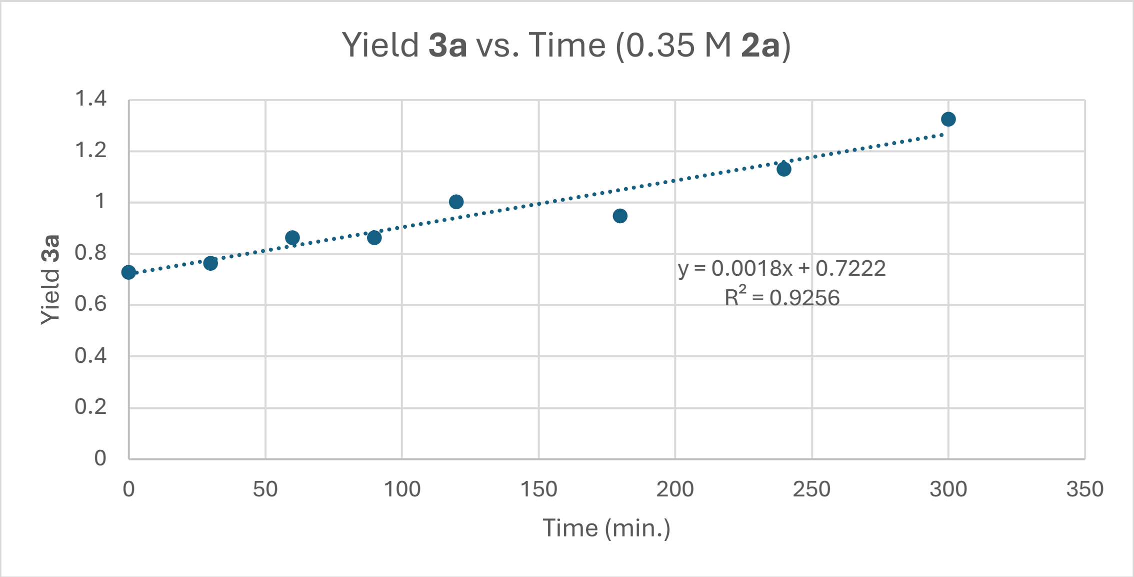

Figure S13. Initial rate profile varying initial **2a** concentration to 0.35 M.

**2a** Kinetic Order			
[**2a**] (M)	*k*	ln([**2a**])	ln(*k*)
0.20	0.0545	-1.57	-2.91
0.26	0.0673	-1.36	-2.70
0.31	0.0446	-1.18	-3.11
0.36	0.0018	-1.03	-6.32
0.45	0.0015	-0.79	-6.50

Table S13. Data for attempted determination of kinetic order in **2a**.

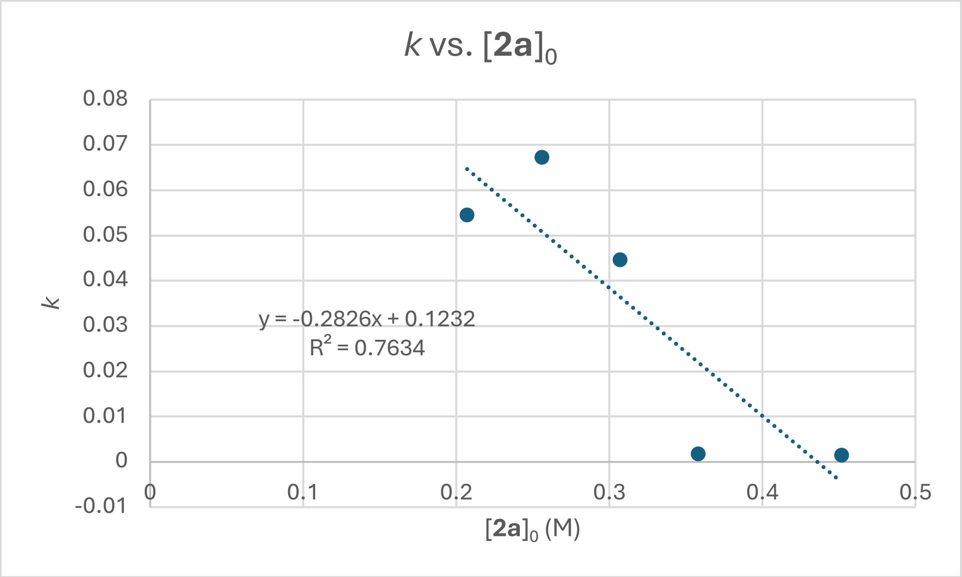

Figure S14. Plot of initial rate constant *k* vs. [**2a**]$_0$.

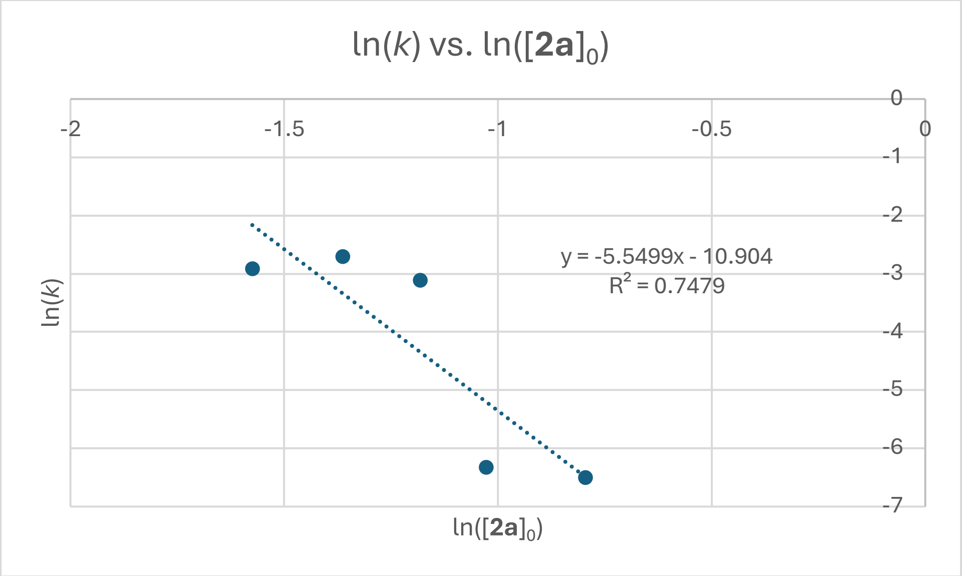

Figure S15. Plot of ln(*k*) vs. ln([**2a**]$_0$).

*t*BuOK (0.35 M)	
Time (min.)	**3a** Yield
0	0.78%
10	2.68%
30	4.51%
60	6.73%
90	9.22%
120	11.12%
180	12.28%
240	13.88%

Table S14. Initial rate data for varying initial *t*BuOK concentration to 0.35 M.

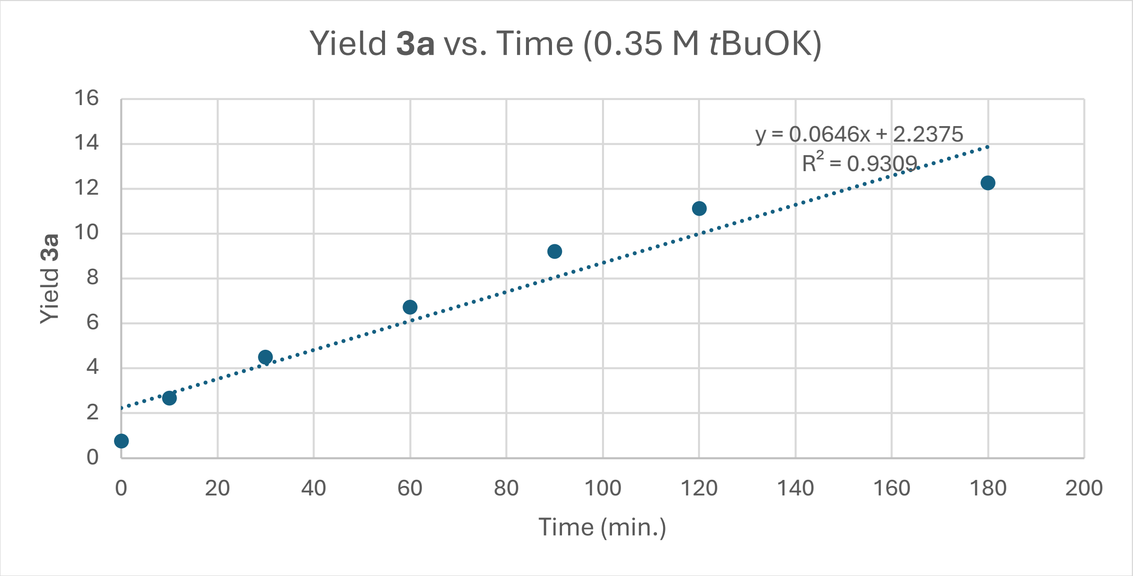

Figure S16. Initial rate profile varying initial *t*BuOK concentration to 0.35 M.

*t*BuOK (0.4 M) *t*BuOK	
Time (min.)	**3a** Yield
0	2.83%
10	4.76%
30	6.60%
60	7.12%
90	10.22%
120	13.27%
180	16.69%
240	18.45%

Table S15. Initial rate data for varying initial *t*BuOK concentration to 0.4 M.

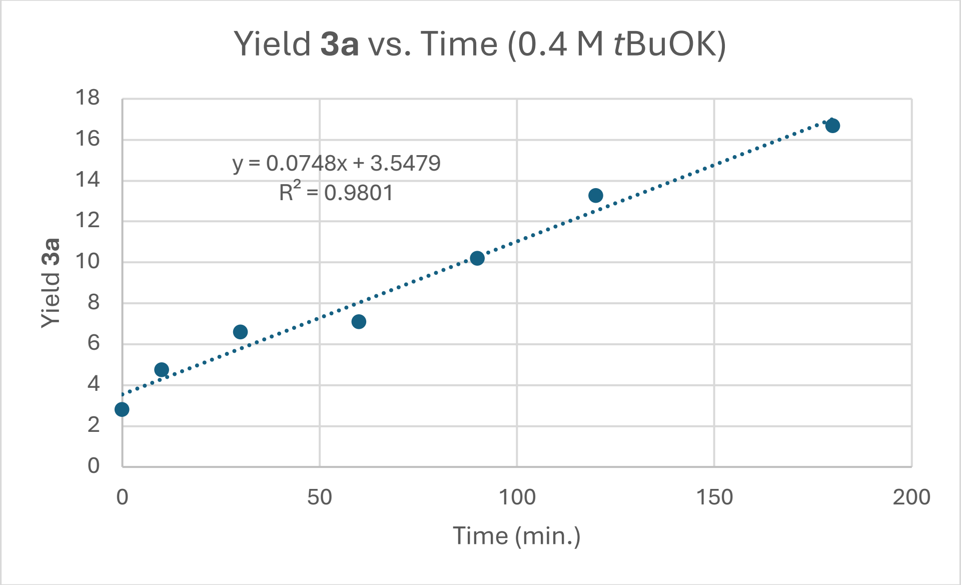

Figure S17. Initial rate profile varying initial *t*BuOK concentration to 0.4 M.

*t*BuOK (0.45 M)	
Time (min.)	**3a** Yield
0	4.73%
10	6.43%
30	9.44%
60	12.56%
90	14.05%
120	16.50%
180	21.36%
240	23.61%

Table S16. Initial rate data for varying initial *t*BuOK concentration to 0.45 M.

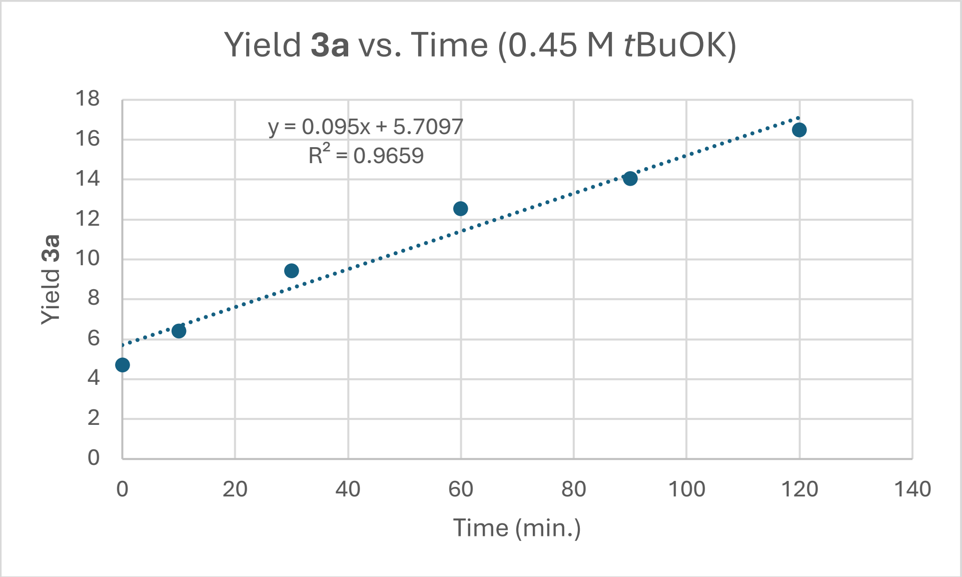

Figure S18. Initial rate profile varying initial *t*BuOK concentration to 0.45 M.

*t*BuOK (0.5 M)	
Time (min.)	**3a** Yield
0	2.05%
10	4.26%
30	4.78%
60	7.14%
90	8.53%
120	10.71%
180	12.76%

Table S17. Initial rate data for varying initial *t*BuOK concentration to 0.5 M.

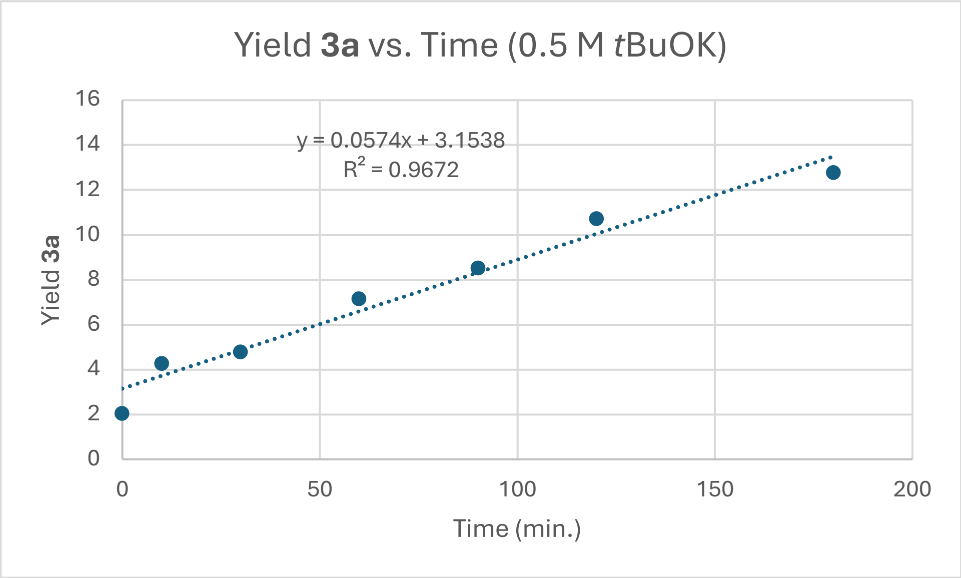

Figure S19. Initial rate profile varying initial *t*BuOK concentration to 0.5 M.

*t*BuOK (0.25 M)	
Time (min.)	**3a** Yield
0	0
10	0
30	0.46%
60	0.44%
90	0.50%
120	1.62%
180	0.76%
240	0.99%

Table S18. Initial rate data varying initial *t*BuOK concentration to 0.25 M. Ratio of **2a** to *t*BuOK is greater than 1. This presumably causes formation of -*ate* complex to be highly favored and thus not leaving unbound base available to react with **1a**, effectively shutting down the reaction.

*t*BuOK Kinetic Order			
[*t*BuOK]$_0$ (M)	*k*	Ln([*t*BuOK]$_0$)	ln(*k*)
0.30	0.0446	-1.18	-3.11
0.35	0.0646	-1.04	-2.74
0.41	0.0748	-0.89	-2.59
0.46	0.095	-0.76	-2.35

Table S19. Data for determination of kinetic order in *t*BuOK.

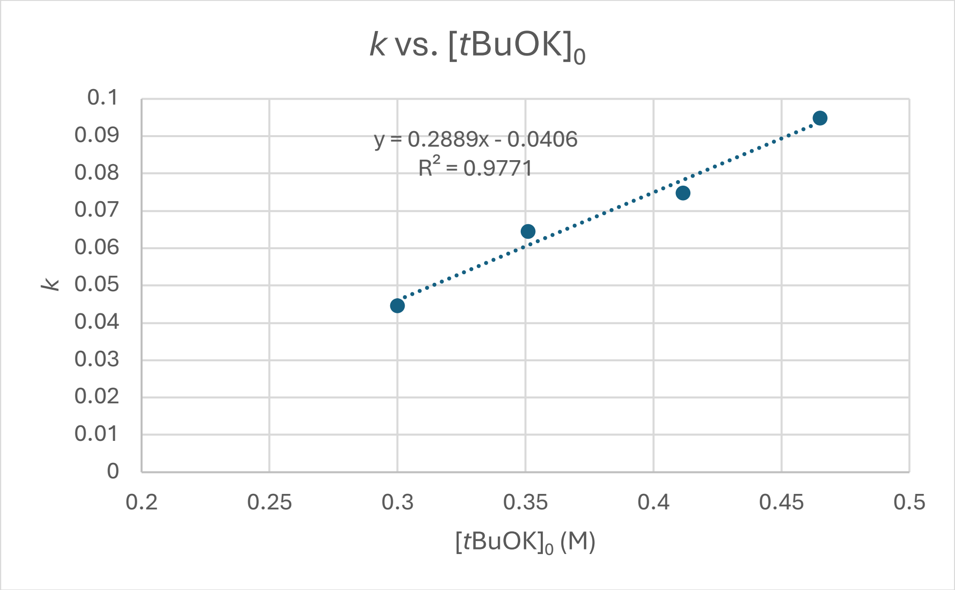

Figure S20. Plot of initial rate constant *k* vs. [*t*BuOK]$_0$.

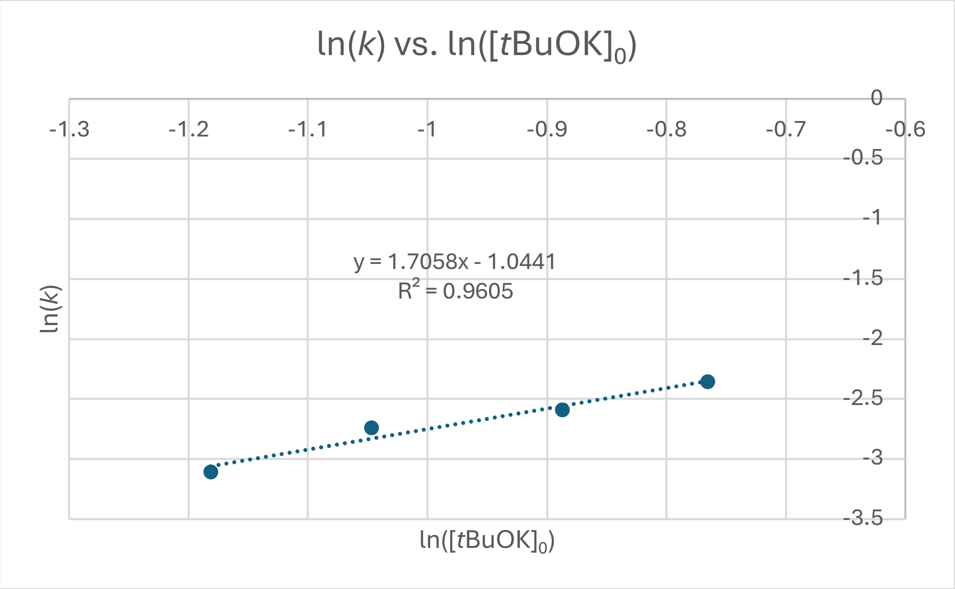

Figure S21. Plot of ln(*k*) vs. ln([*t*BuOK]$_0$).

References

47) S. Ochi, Z. Zhang, Y. Xia, G. Dong, "Rhodium-Catalyzed (4+1) Cycloaddition between Benzocyclobutenones and Styrene-Type Alkenes" *Angew Chem Int Ed* **2022**, *61*, e202202703.
48) P. Álvarez-Bercedo, A. Flores-Gaspar, A. Correa, R. Martin, "Pd-Catalyzed Intramolecular Acylation of Aryl Bromides via C−H Functionalization: A Highly Efficient Synthesis of Benzocyclobutenones" *J. Am. Chem. Soc.* **2010**, *132*, 466–467.
49) J. N. Humke, R. G. Belli, E. E. Plasek, S. S. Kargbo, A. Q. Ansel, C. C. Roberts, "Nickel binding enables isolation and reactivity of previously inaccessible 7-aza-2,3-indolynes" *Science* **2024**, *384*, 408–414.
50) C. C. M. Roberts, J. N. Humke, A. Q. Ansel, R. G. Belli, S. S. Kargbo, E. E. Plasek, *Synthesis of Heteroarynes and Use Thereof*, **2025**, US20250002518A1.
51) M. Terada, Y. Nishii, M. Miura, "Synthesis, crystal structure and reactivity of η^2 -thiophyne Ni complexes" *Chem. Commun.* **2018**, *54*, 2918–2921.
52) X. Gu, H. Li, B. Shan, Z. Liu, Q. Miao, "Synthesis, Structure, and Properties of Tetrabenzo[7]circulene" *Org. Lett.* **2017**, *19*, 2246–2249.
53) M. F. Boselli, I. Ghosh, N. Intini, M. Fattalini, A. Puglisi, B. König, M. Benaglia, "Visible-Light Photoredox Catalytic Direct *N* -(Het)Arylation of Lactams" *Chemistry A European J* **2025**, *31*, e202404385.
54) M. R. Michaelides, A. S. Judd, S. R. Fix-Stenzel, R. F. Clark, B. K. Sorensen, Z. Ji, *Pyrrolopyridine and Pyrrolopyrimidine Inhibitors of Kinases*, **2011**, US20110281842A1.
55) J.-Y. Ouyang, F.-F. Shen, H.-Q. Zhao, J.-J. Chen, Z.-D. Wen, H.-M. Jiang, J.-H. Qin, Q. Sun, J.-H. Li, X.-H. Ouyang, "Aryldiazonium Salt-Triggered [2 + 2 + 1] Heteroannulation of Indoles by an Arylhydrazone Radical-Relayed 1,5-Hydrogen Atom Transfer" *Org. Lett.* **2023**, *25*, 6549–6554.
56) S. Ferrario, S. Rossi, N. Intini, J. Bruno-Colmenarez, M. Baumann, M. Benaglia, "Photocatalytic Addition of *N* -Oxazolidinone Radicals to Arenes and Heteroarenes in Batch and in Flow Mode" *Org. Lett.* **2025**, *27*, 12276–12280.
57) S. Song, X. Sun, X. Li, Y. Yuan, N. Jiao, "Efficient and Practical Oxidative Bromination and Iodination of Arenes and Heteroarenes with DMSO and Hydrogen Halide: A Mild Protocol for Late-Stage Functionalization" *Org. Lett.* **2015**, *17*, 2886–2889.
58) G. L. Tolnai, S. Ganss, J. P. Brand, J. Waser, "C2-Selective Direct Alkynylation of Indoles" *Org. Lett.* **2013**, *15*, 112–115.
59) T. Hao, L. Huang, Y. Wei, M. Shi, "Copper-Catalyzed Synthesis of Indolyl Benzo[*b*]carbazoles and Their Photoluminescence Property" *Org. Lett.* **2021**, *23*, 5133–5137.
60) S. Kato, T. Furuya, M. Nitani, N. Hasebe, Y. Ie, Y. Aso, T. Yoshihara, S. Tobita, Y. Nakamura, "A Series of π-Extended Thiadiazoles Fused with Electron-Donating

Heteroaromatic Moieties: Synthesis, Properties, and Polymorphic Crystals" *Chemistry A European J* **2015**, *21*, 3115–3128.

61) D. Saha, R. Ghosh, A. Sarkar, "3-Indolylphosphines as ligand for palladium in Suzuki–Miyaura coupling reaction of chloroarenes: substituent effects" *Tetrahedron* **2013**, *69*, 3951–3960.

62) S.-B. Yan, Z. Li, X.-Q. Hu, S. Zhang, W.-L. Duan, "Pd/Chiral Phosphoric Acid-Enabled Asymmetric Intramolecular Double C–H Activation Reaction for the Synthesis of *P*-Stereogenic Benzophosphole Oxides" *Org. Lett.* **2025**, *27*, 1481–1486.

63) X. Yuan, J.-F. Yao, Z.-Y. Tang, "Decarboxylative Fluorination of Electron-Rich Heteroaromatic Carboxylic Acids with Selectfluor" *Org. Lett.* **2017**, *19*, 1410–1413.

64) S. Motsch, C. Schütz, P. H. Huy, "Systematic Evaluation of Sulfoxides as Catalysts in Nucleophilic Substitutions of Alcohols" *Eur J Org Chem* **2018**, *2018*, 4541–4547.

65) C. A. Merlic, D. M. McInnes, "Synthesis of indolocarbazoles via sequential palladium catalyzed cross-coupling and benzannulation reactions" *Tetrahedron Letters* **1997**, *38*, 7661–7664.

66) K. Shigemori, M. Watanabe, J. Kong, K. Mitsudo, A. Wakamiya, H. Mandai, S. Suga, "Iodide-Mediated or Iodide-Catalyzed Demethylation and Friedel–Crafts C–H Borylative Cyclization Leading to Thiophene-Fused 1,2-Oxaborine Derivatives" *Org. Lett.* **2019**, *21*, 2171–2175.

X-Ray Crystallography Data

Table S20. Crystal data and structure refinement for 3r (CCDC Deposition #2541836).

Identification code	1968_cole_dong_1
Empirical formula	$C_{19}H_{16}BrNO$
Formula weight	354.24
Temperature/K	100(2)
Crystal system	orthorhombic
Space group	Pnma
a/Å	16.5088(9)
b/Å	7.0006(4)
c/Å	13.1085(6)
α/°	90
β/°	90
γ/°	90
Volume/Å^3	1514.97(14)
Z	4
ρ_{calc}g/cm^3	1.553
μ/mm^{-1}	2.714
F(000)	720.0
Crystal size/mm^3	0.028 × 0.023 × 0.015
Radiation	MoKα (λ = 0.71073)
2Θ range for data collection/°	3.968 to 62.34
Index ranges	$-23 \le h \le 23$, $-10 \le k \le 9$, $-19 \le l \le 15$
Reflections collected	31383
Independent reflections	2515 [R_{int} = 0.0518, R_{sigma} = 0.0378]
Data/restraints/parameters	2515/0/132
Goodness-of-fit on F^2	1.076
Final R indexes [I>=2σ (I)]	R_1 = 0.0283, wR_2 = 0.0673
Final R indexes [all data]	R_1 = 0.0510, wR_2 = 0.0719
Largest diff. peak/hole / e Å^{-3}	0.33/-0.53

$R_{int} = \Sigma | F_o^2 - \langle F_o^2 \rangle | / \Sigma | F_o^2|$

$R1 = \Sigma || F_o| - | F_c|| / \Sigma| F_o|$

$wR2 = [\Sigma [w (F_o^2 - F_c^2)^2] / \Sigma [w (F_o^2)^2]]^{1/2}$

Goodness-of-fit $= [\Sigma [w (F_o^2 - F_c^2)^2] / (n-p)^{1/2}$

n: number of independent reflections; p: number of refined parameters

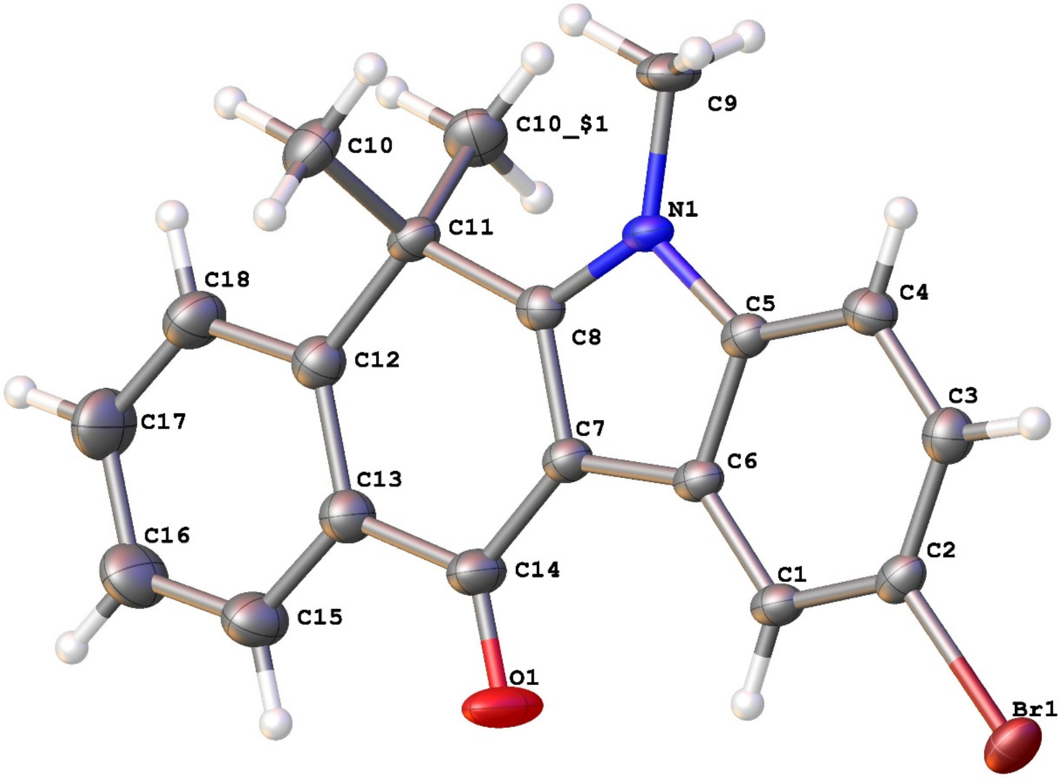
C9
C10_$1
C10
N1
C11
C18
C4
C5
C8
C12
C3
C17
C7
C6
C13
C16
C2
C14
C1
C15
O1
Br1

[1]+X,1/2-Y,+Z

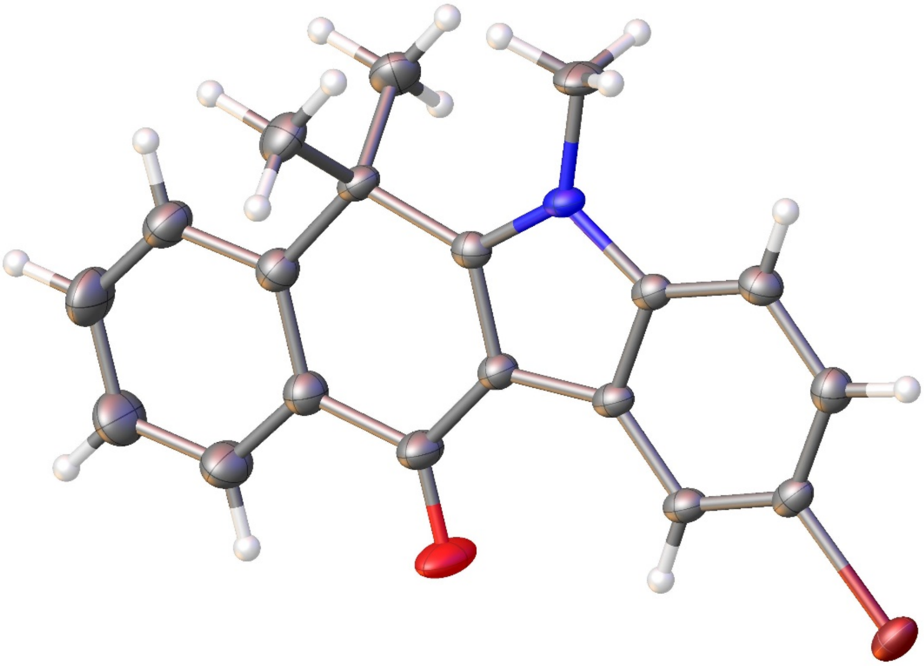

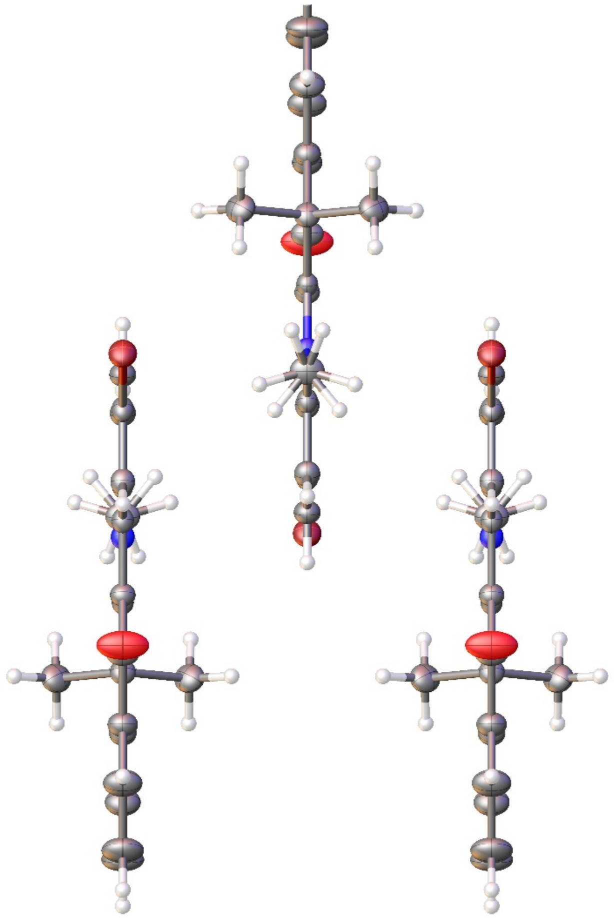

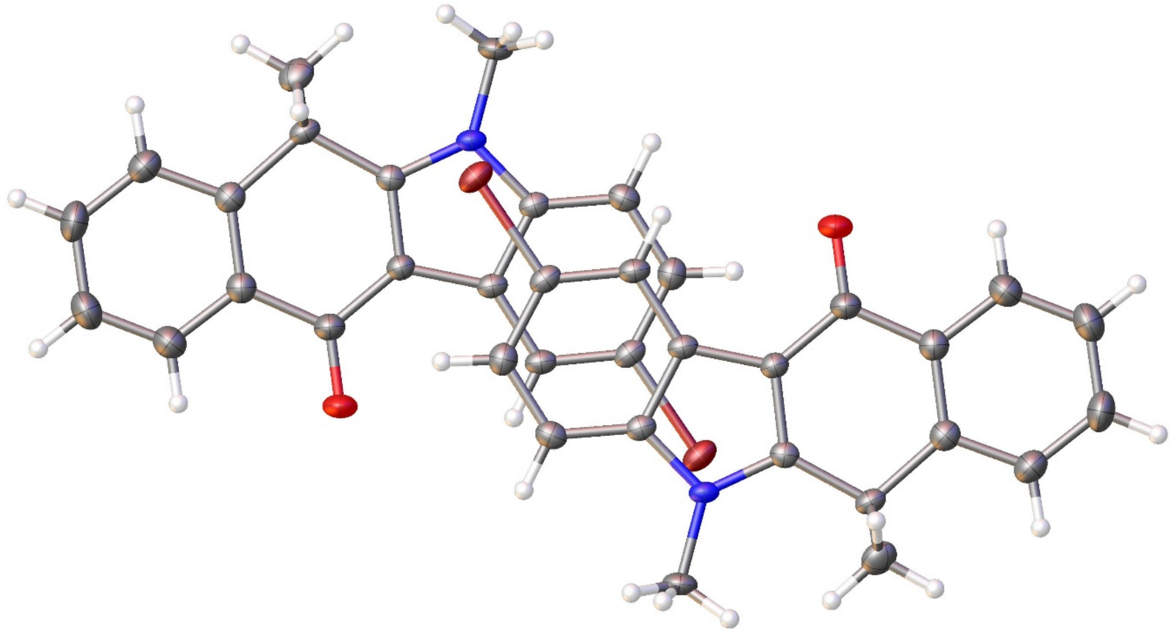

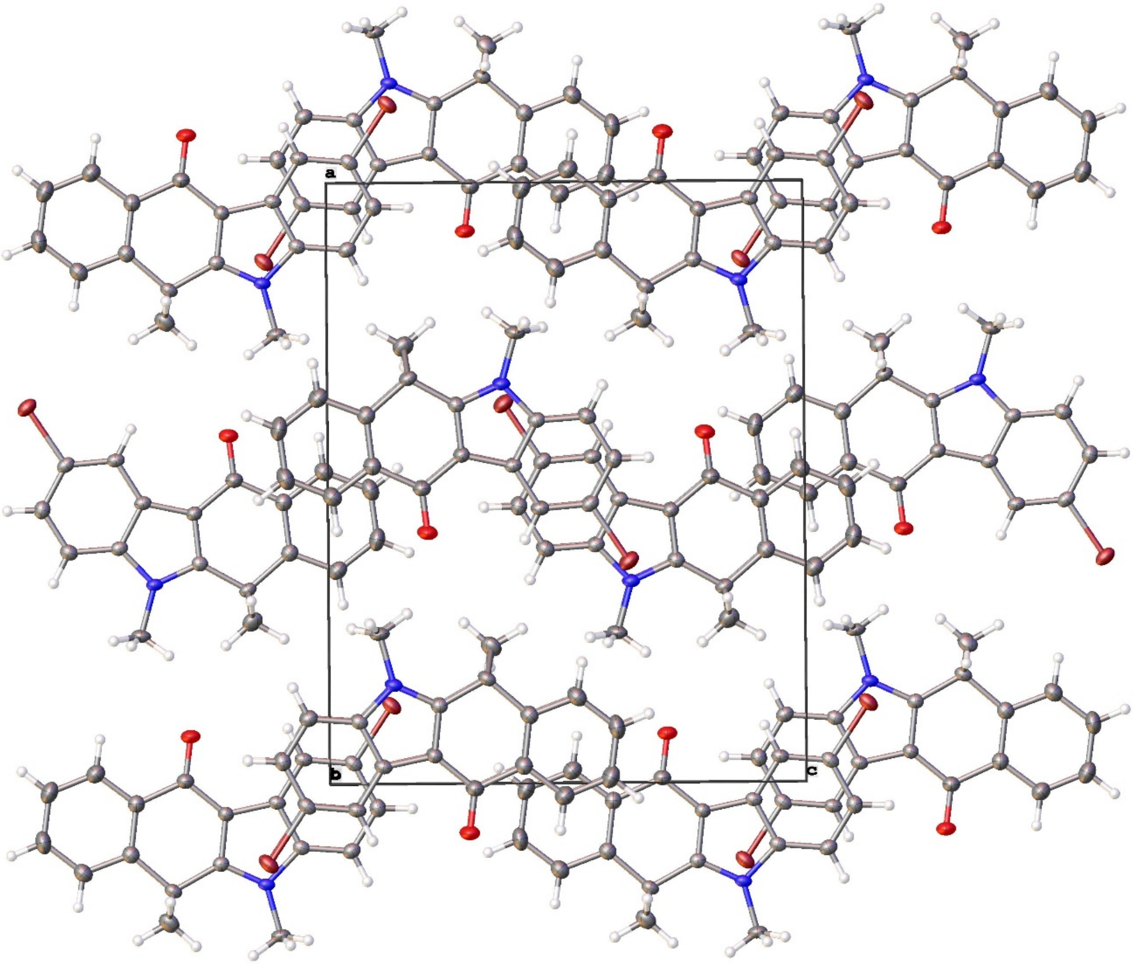

a
b
c

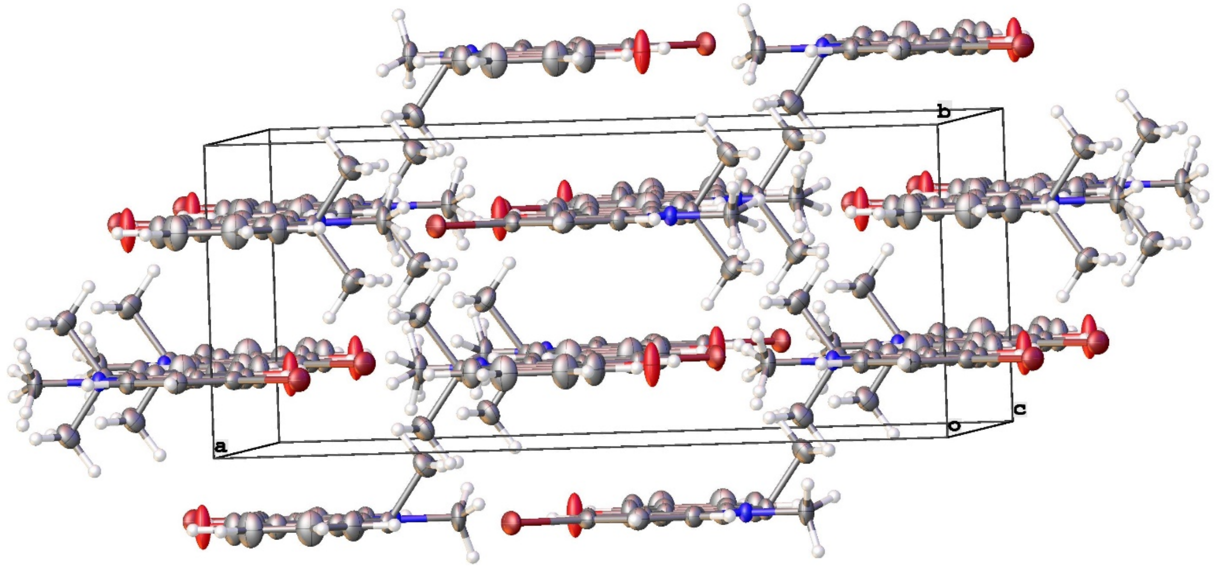

b
a
o
c

NMR Spectra

^{1}H NMR for **S2** (600 MHz, $CDCl_3$)

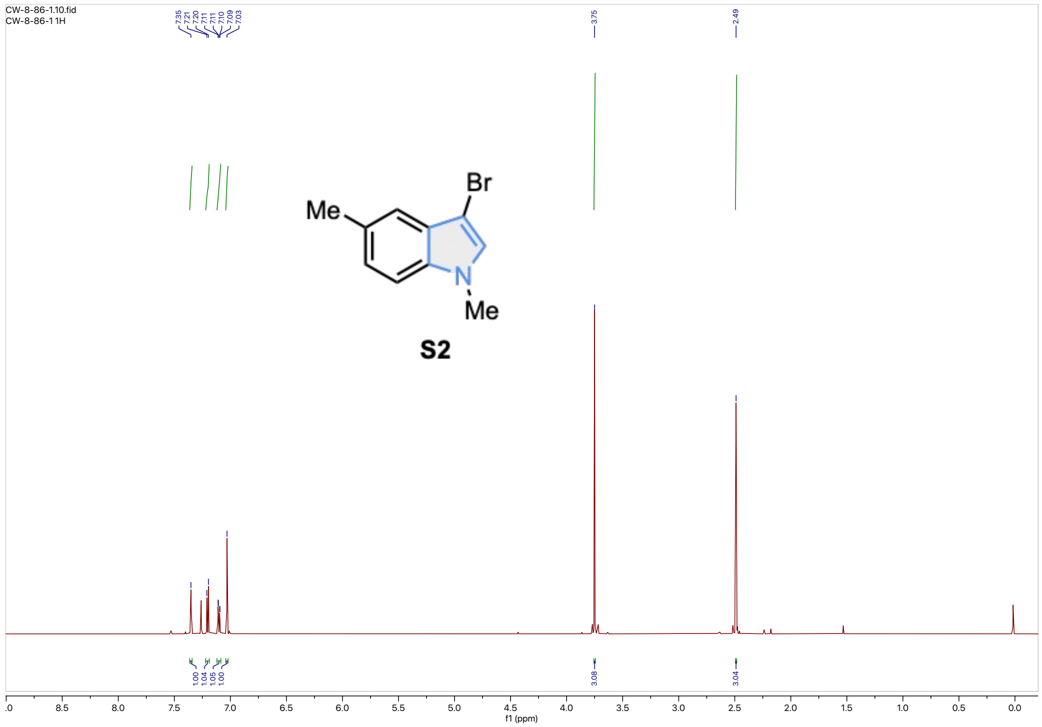

^{13}C NMR for **S2** (151 MHz, $CDCl_3$)

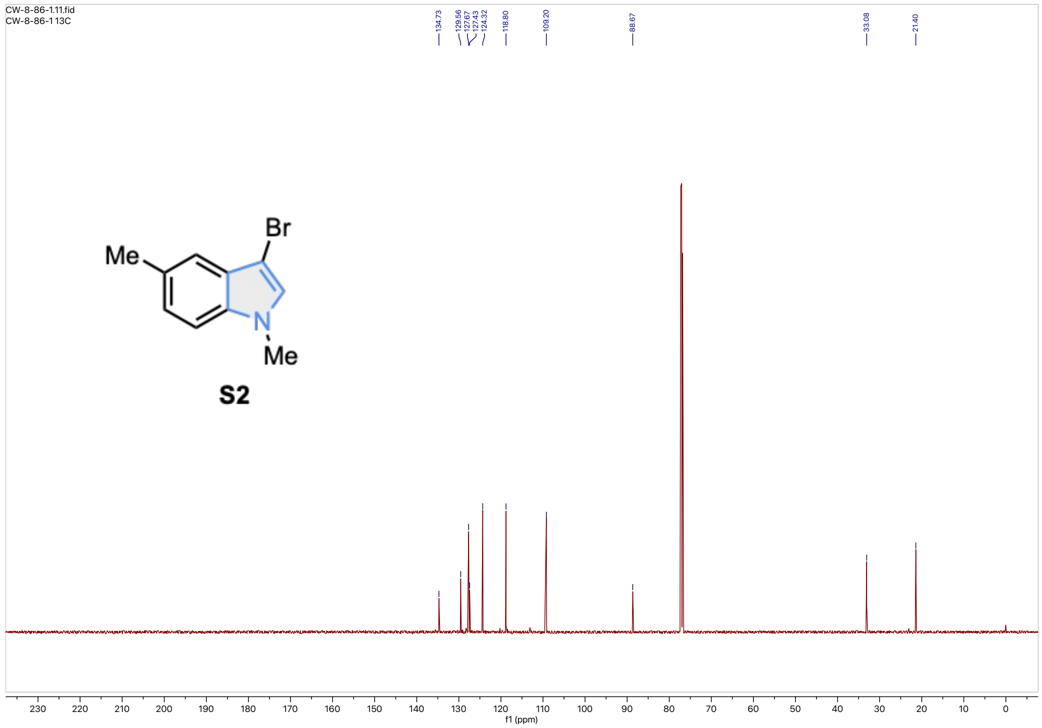

^{1}H NMR for **S5** (500 MHz, $CDCl_3$)

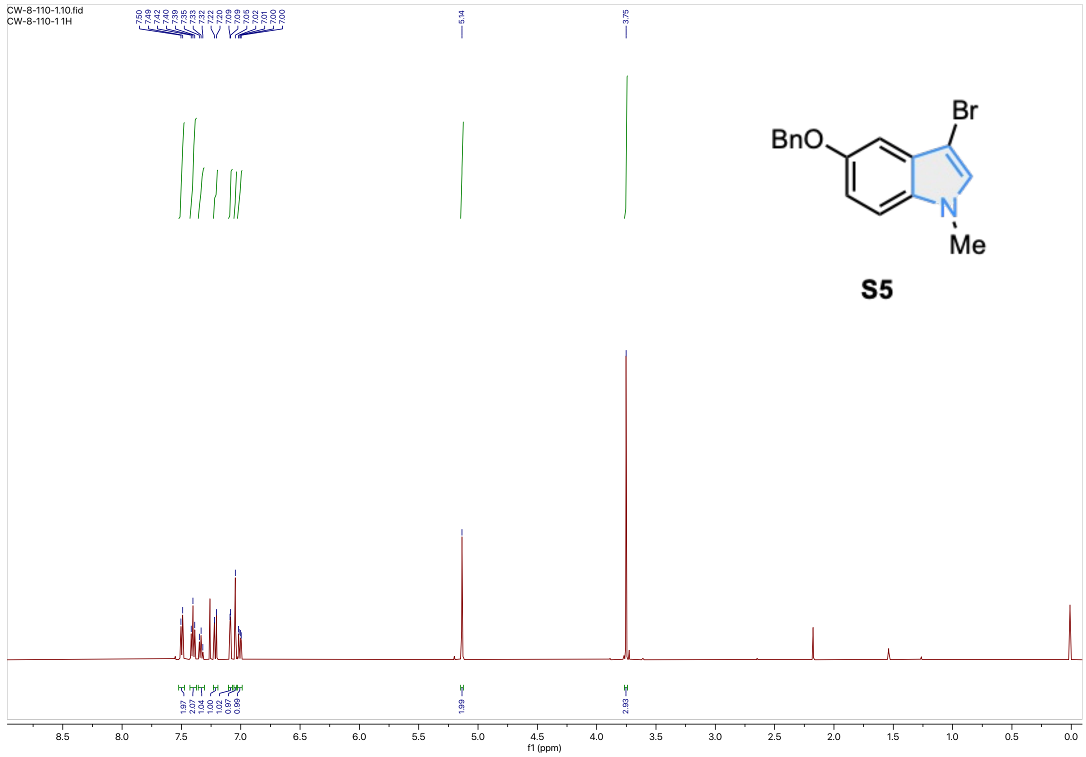

^{13}C NMR for **S5** (126 MHz, $CDCl_3$)

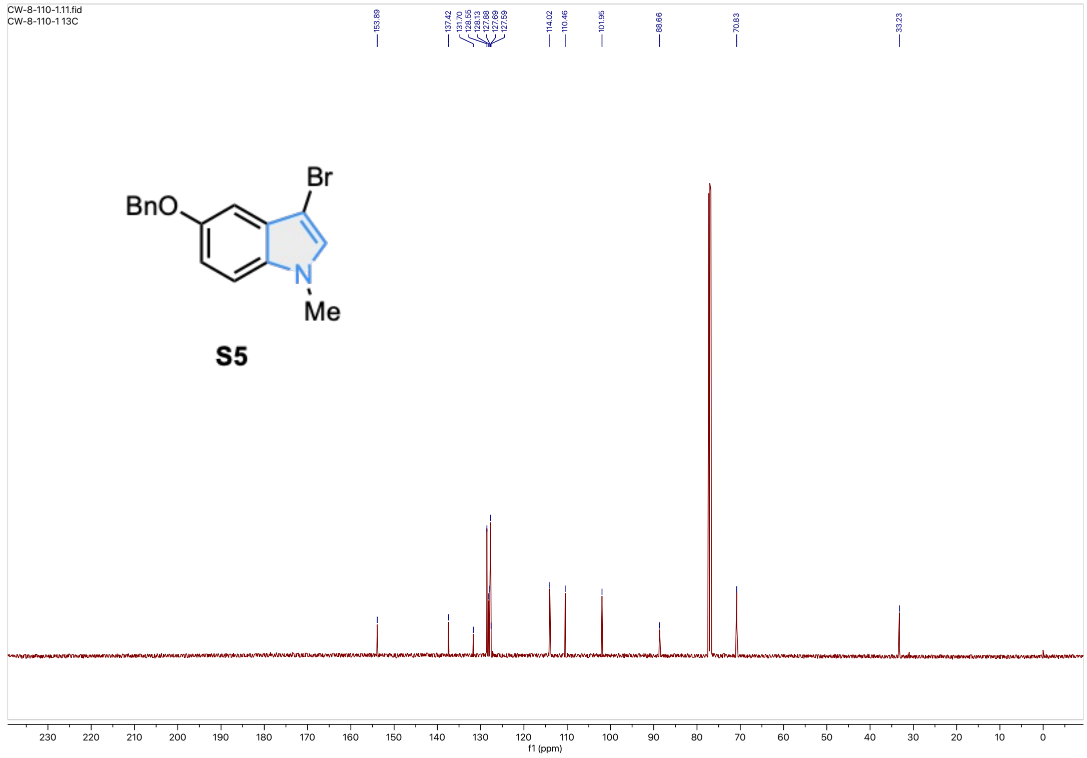

^{1}H NMR for **S7** (500 MHz, $CDCl_3$)

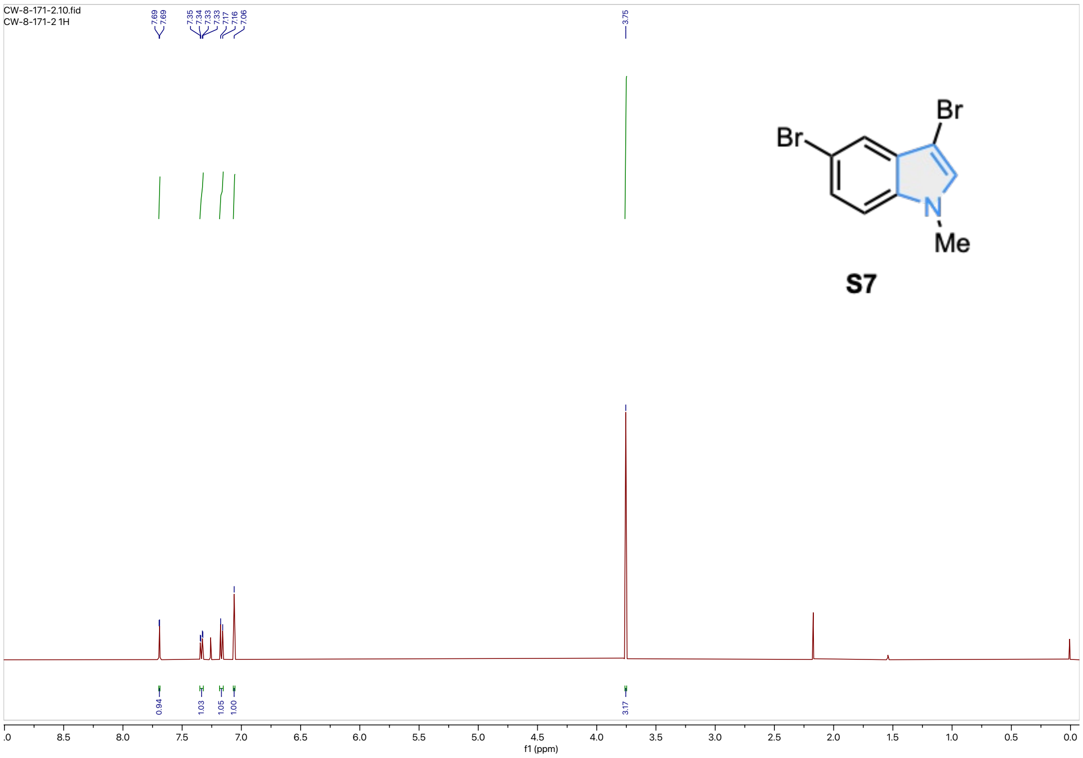

^{13}C NMR for **S7** (126 MHz, $CDCl_3$)

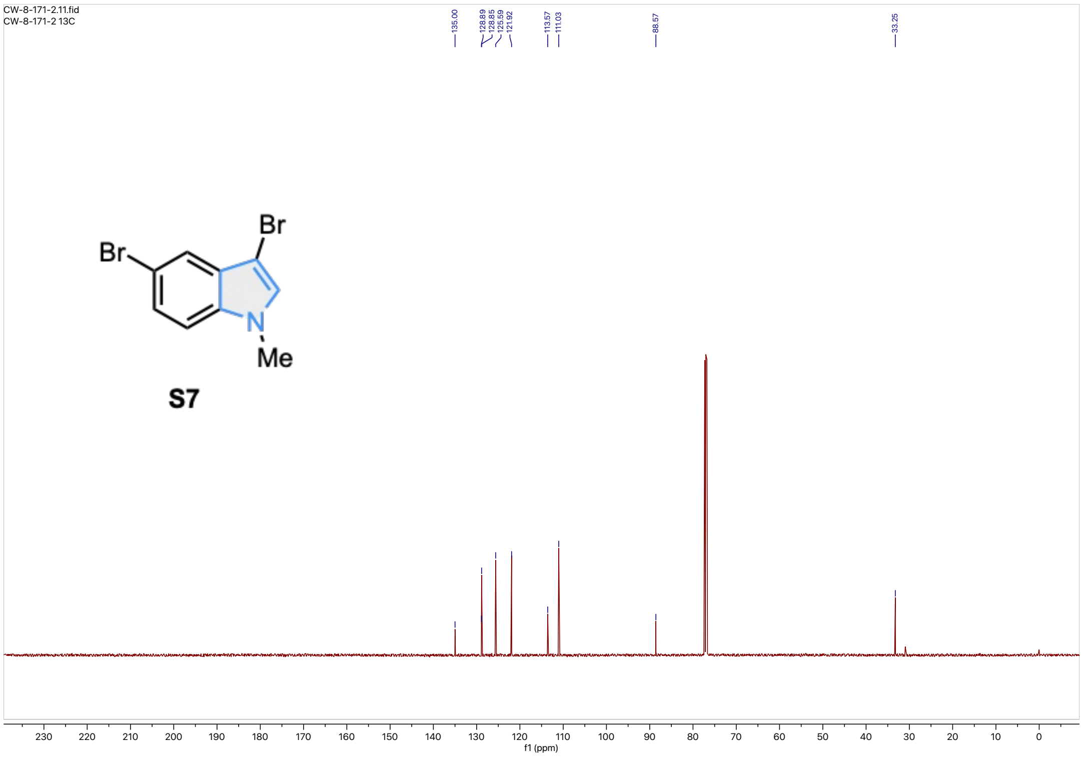

^{1}H NMR for **S10** (500 MHz, $CDCl_3$)

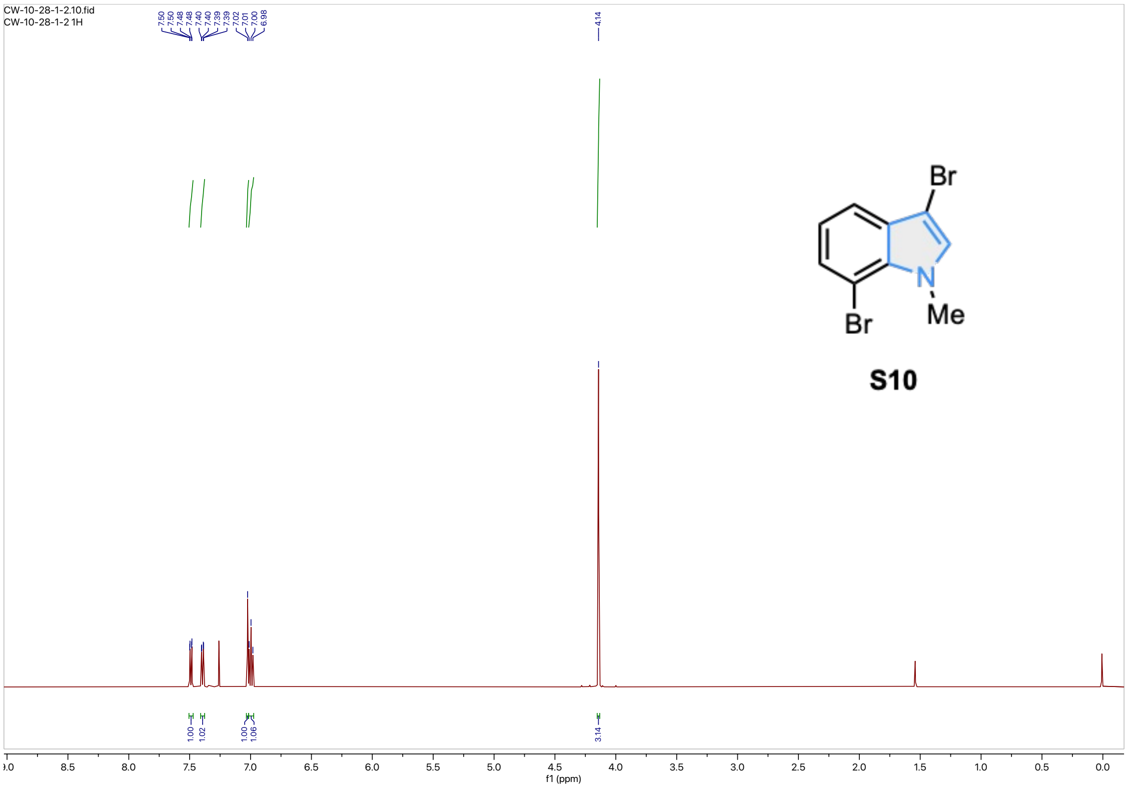

^{13}C NMR for **S10** (126 MHz, $CDCl_3$)

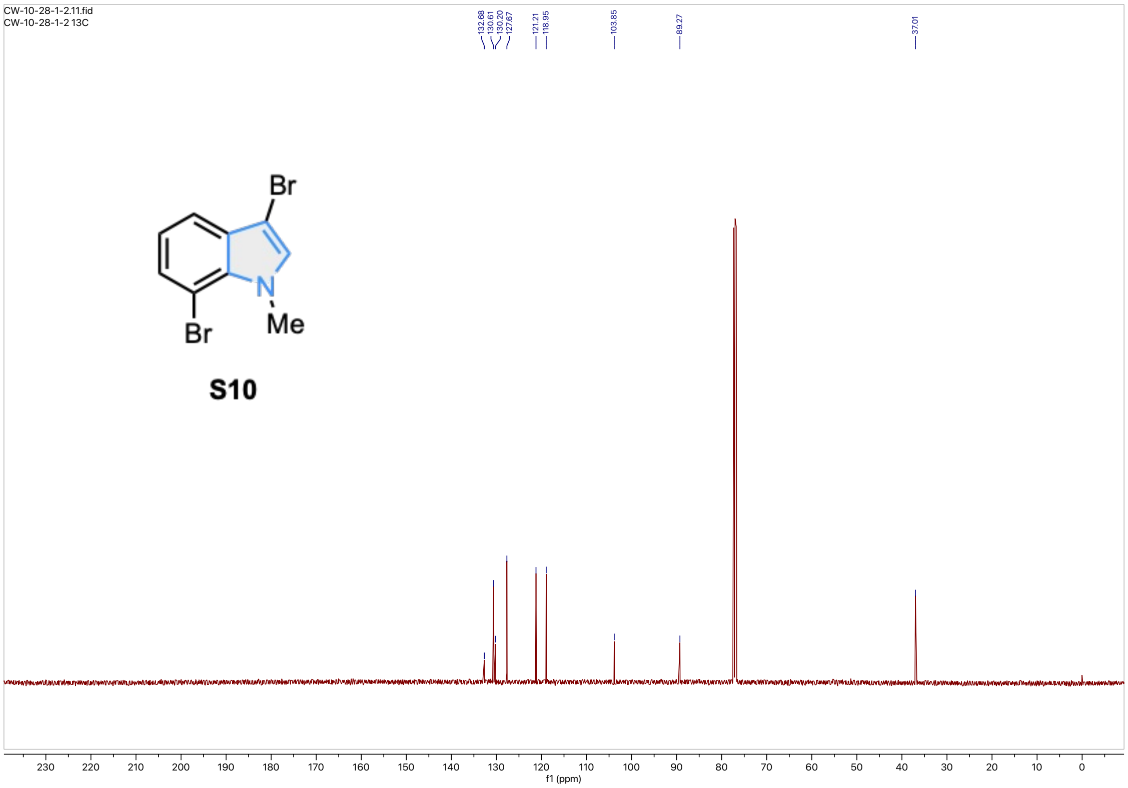

^{1}H NMR for **2d** (500 MHz, $CDCl_3$)

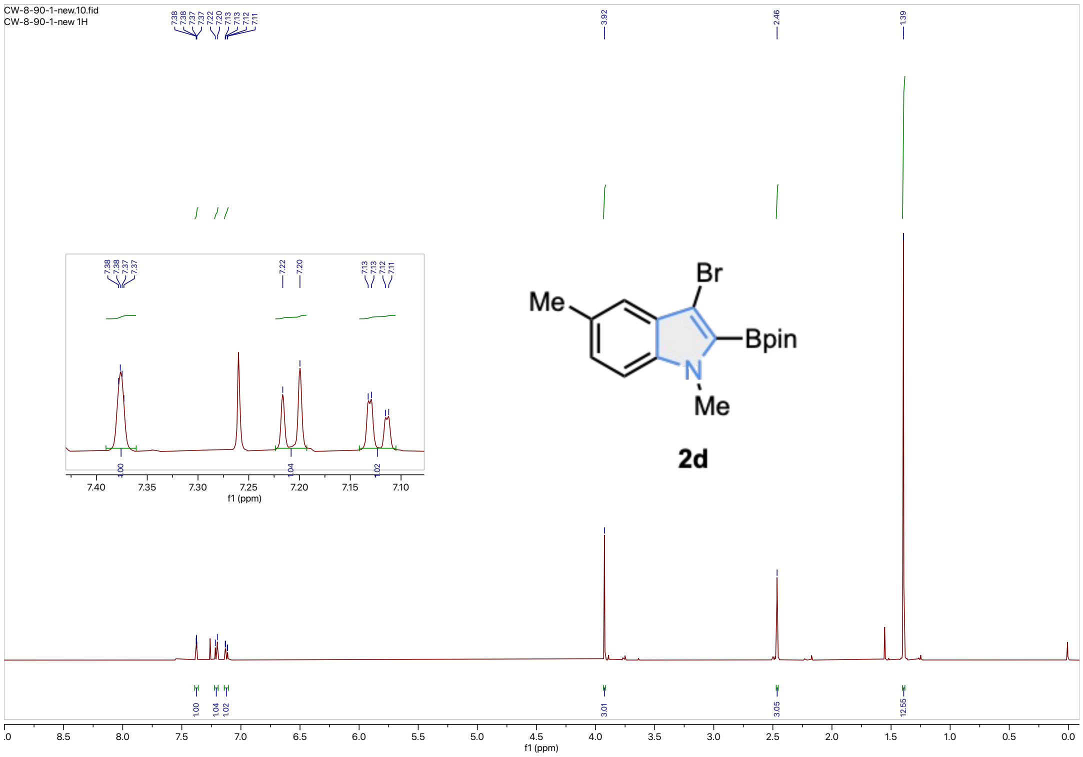

^{13}C NMR for **2d** (126 MHz, $CDCl_3$)

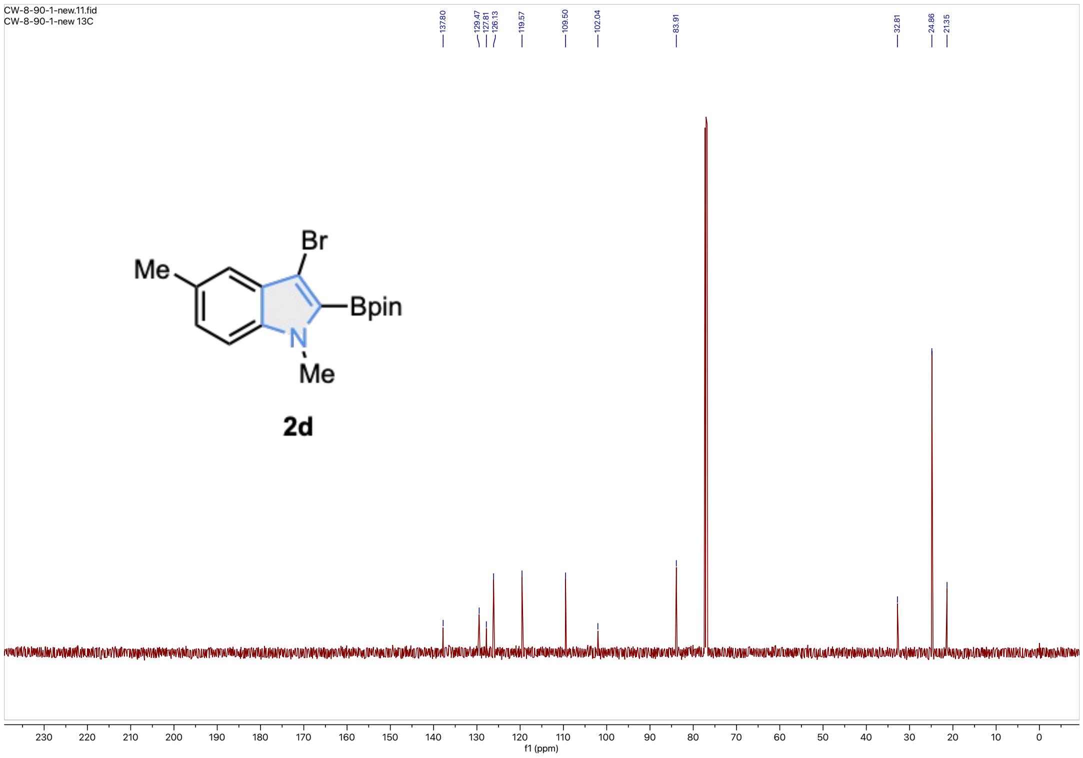

^{11}B NMR for **2d** (160 MHz, $CDCl_3$)

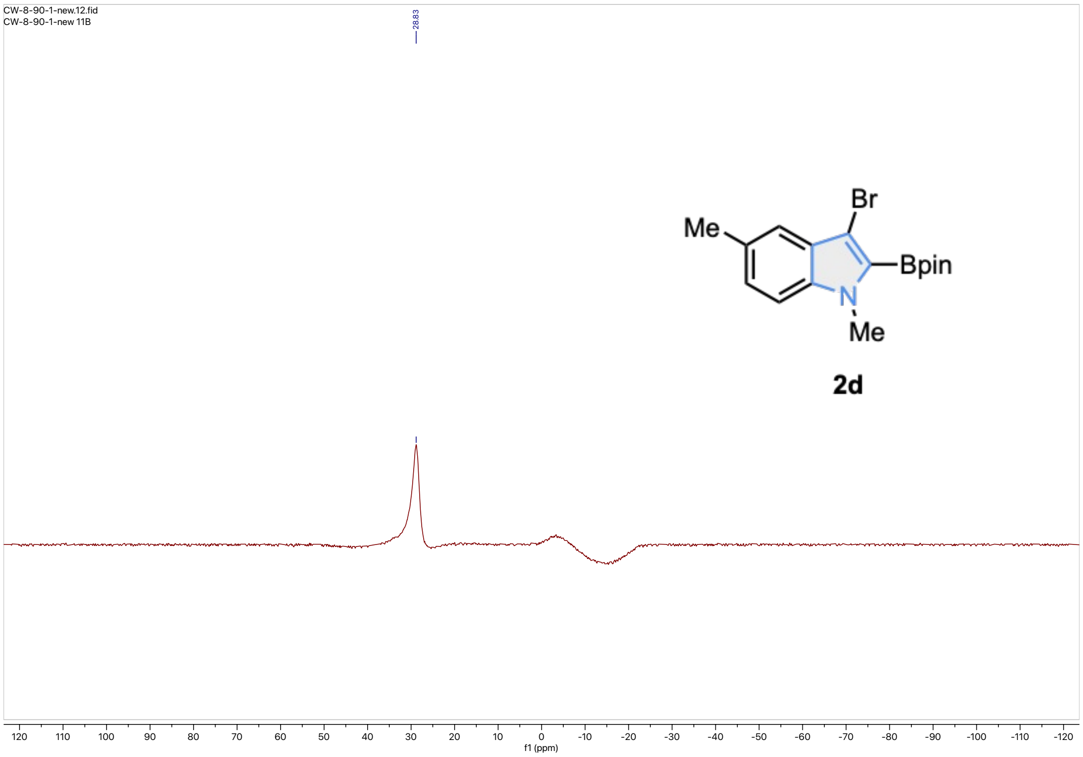

^{1}H NMR for **2e** (500 MHz, $CDCl_3$)

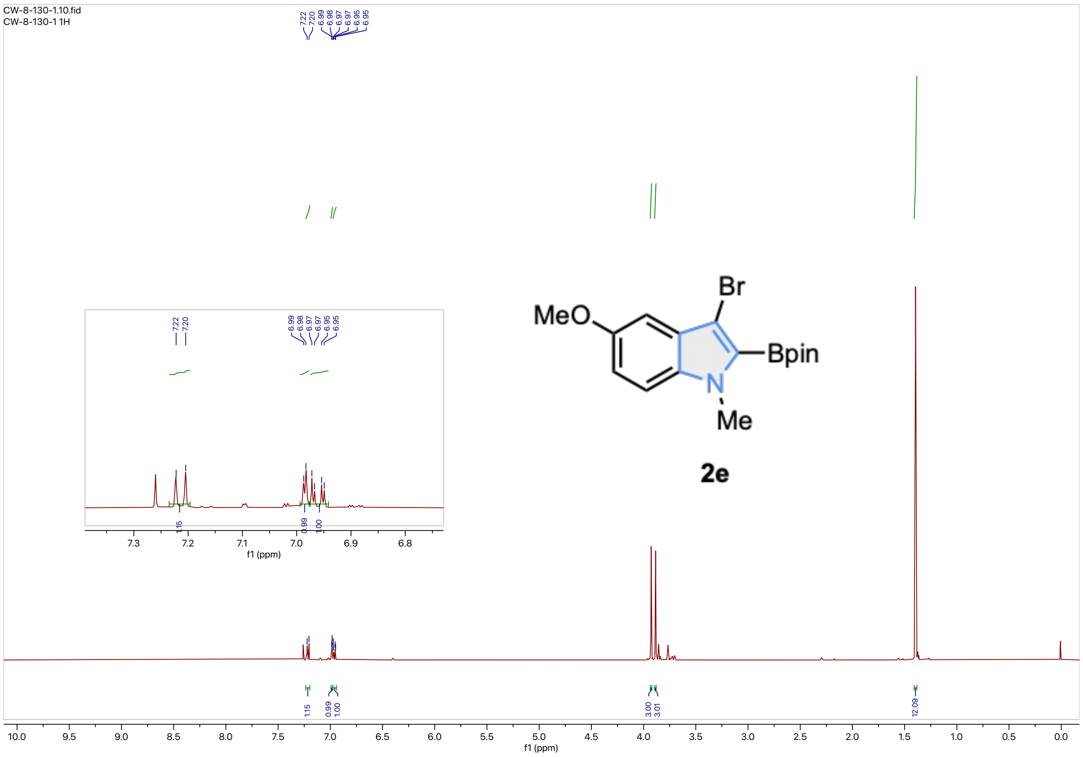

^{13}C NMR for **2e** (126 MHz, $CDCl_3$)

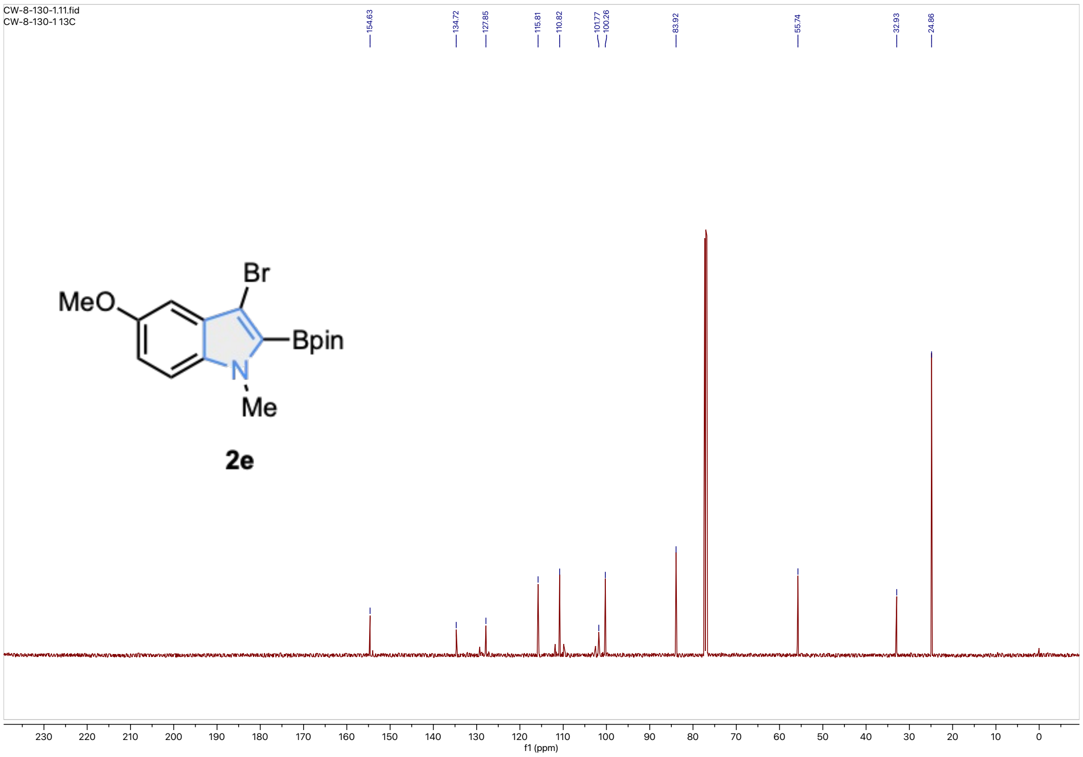

^{11}B NMR for **2e** (160 MHz, $CDCl_3$)

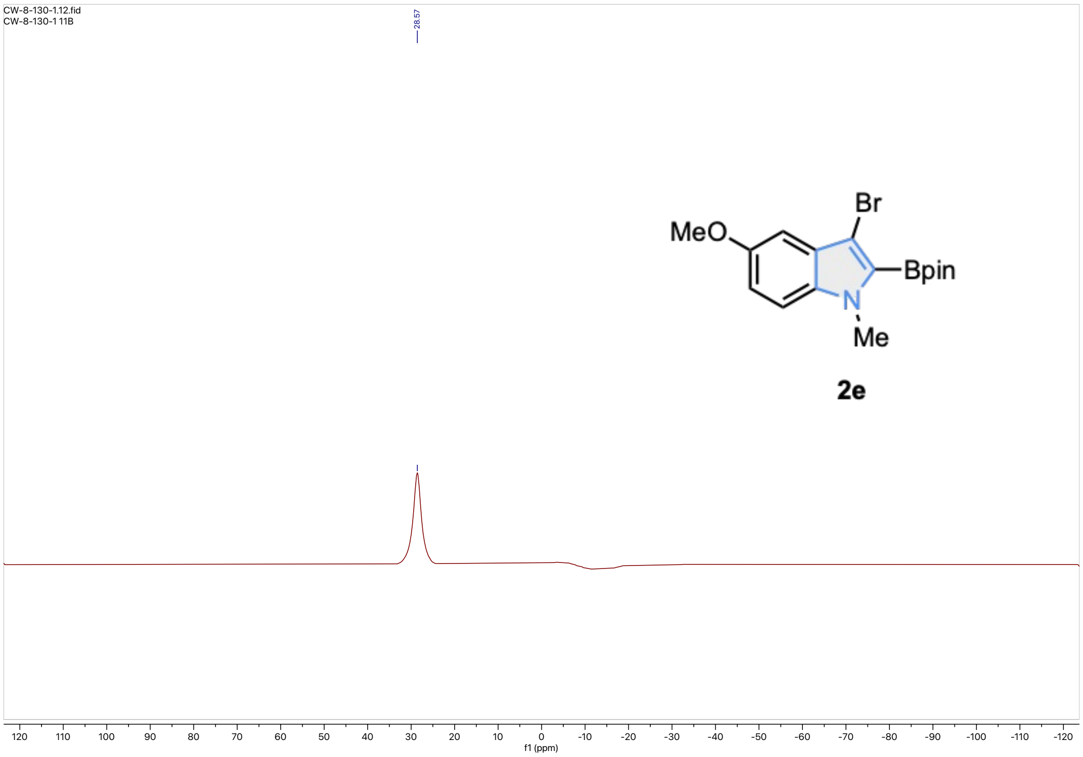

^{1}H NMR for **2f** (500 MHz, $CDCl_3$)

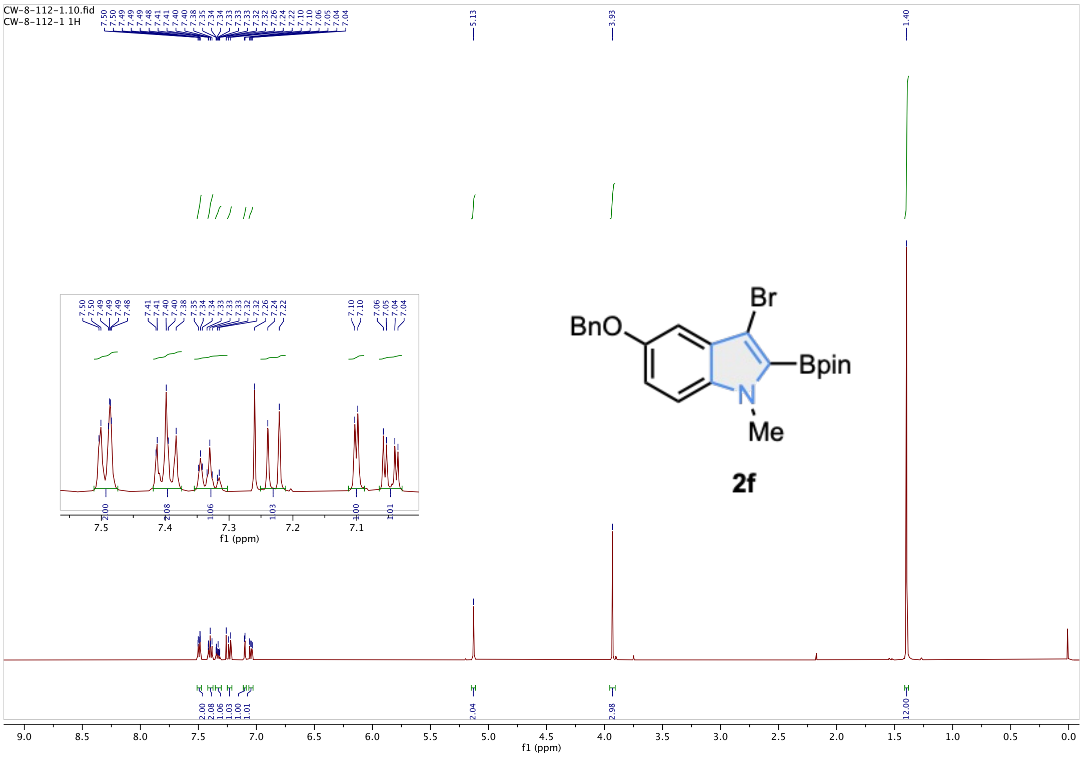

^{13}C NMR for **2f** (126 MHz, $CDCl_3$)

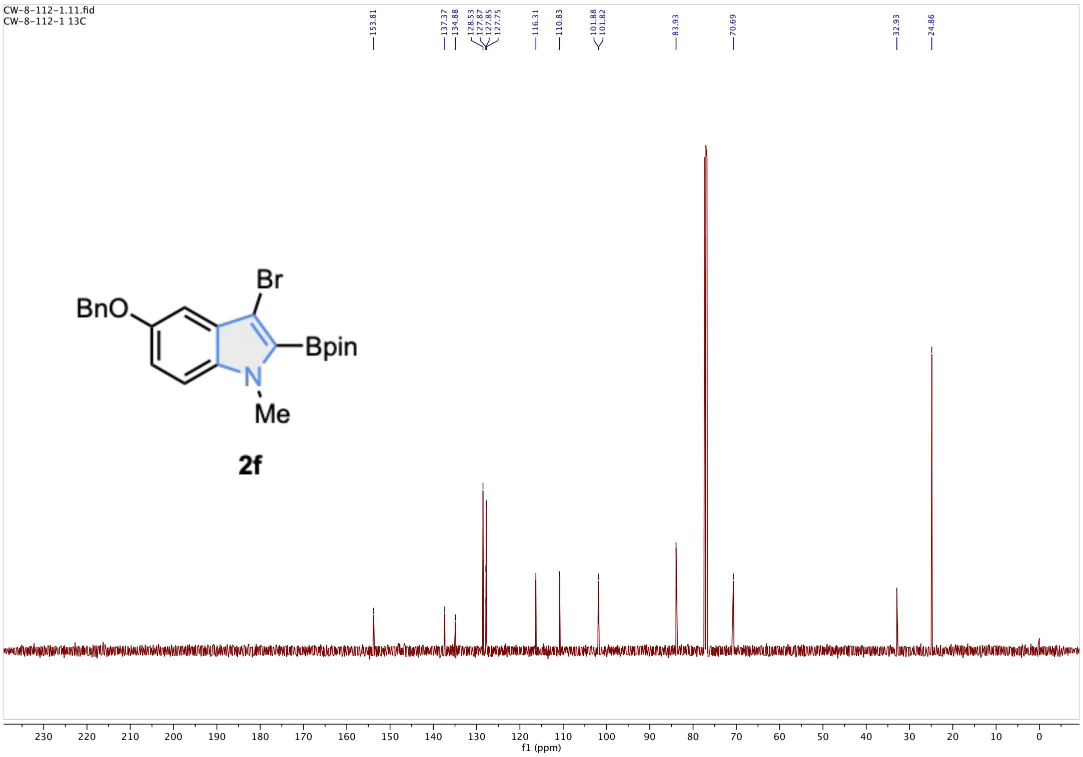

^{11}B NMR for **2f** (160 MHz, $CDCl_3$)

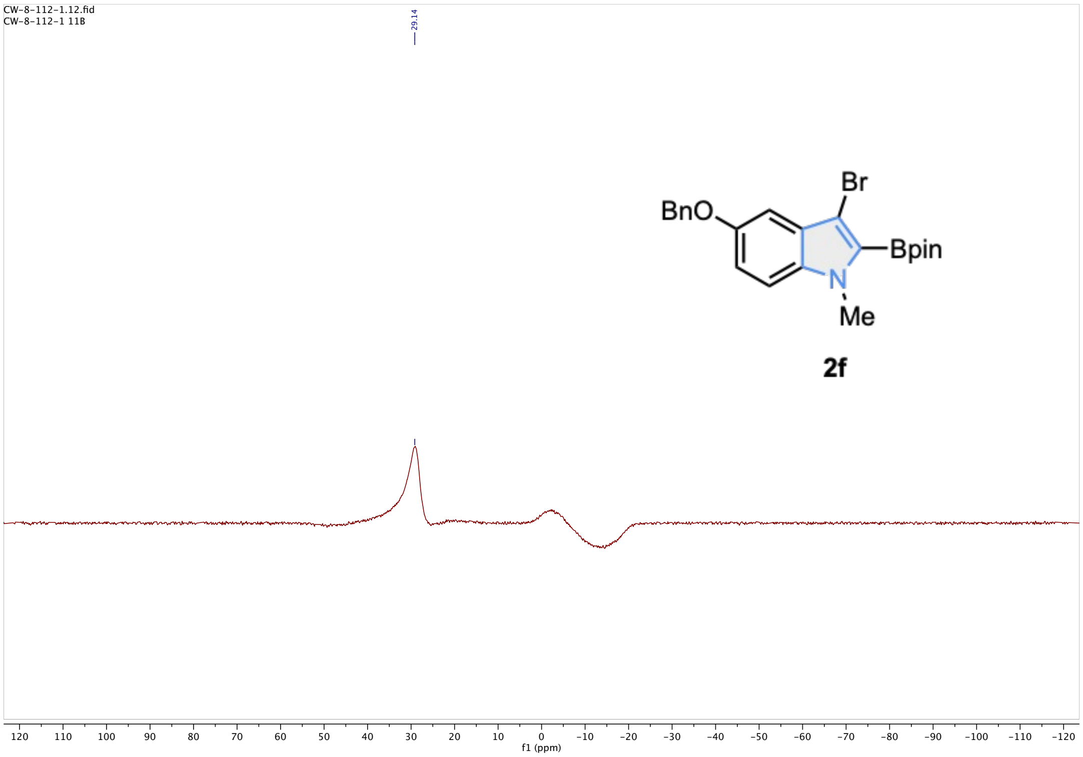

^{1}H NMR for **2g** (500 MHz, $CDCl_3$)

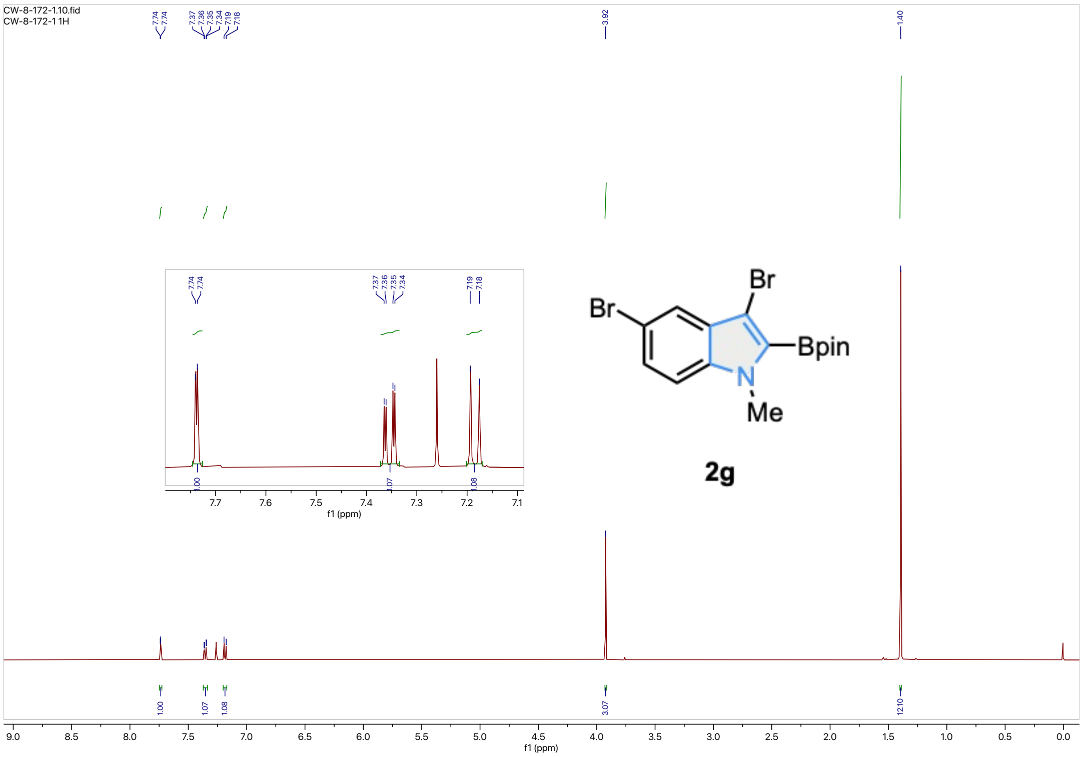

^{13}C NMR for **2g** (126 MHz, $CDCl_3$)

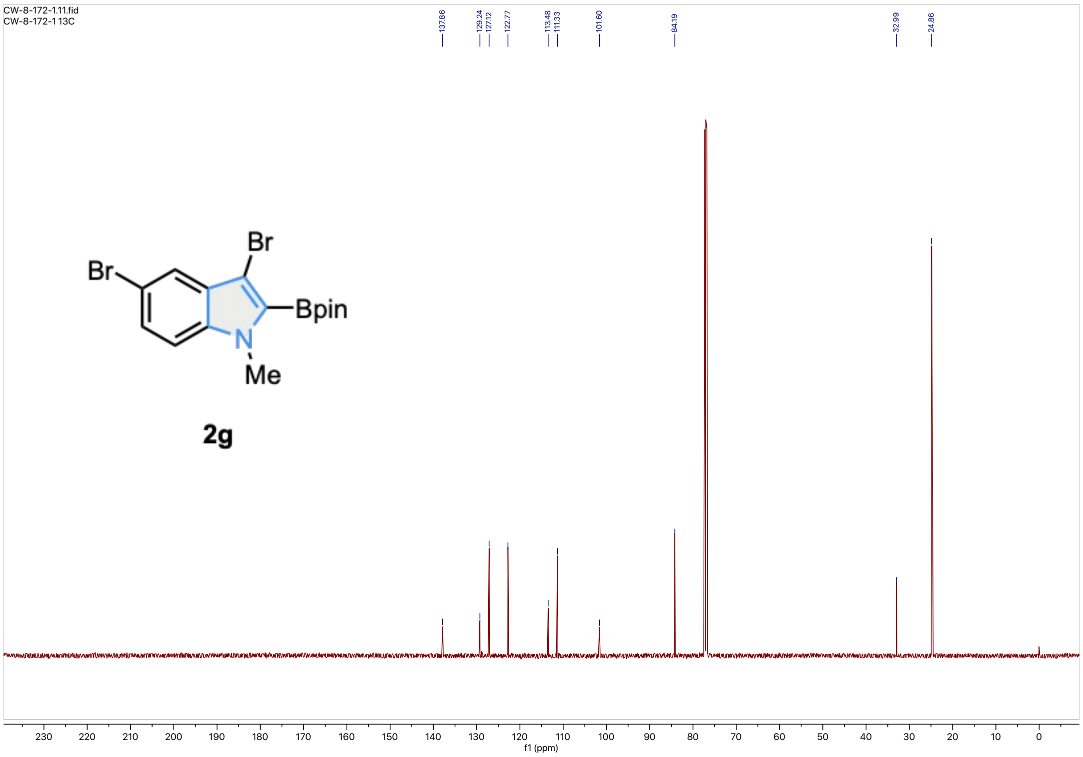

^{11}B NMR for **2g** (160 MHz, $CDCl_3$)

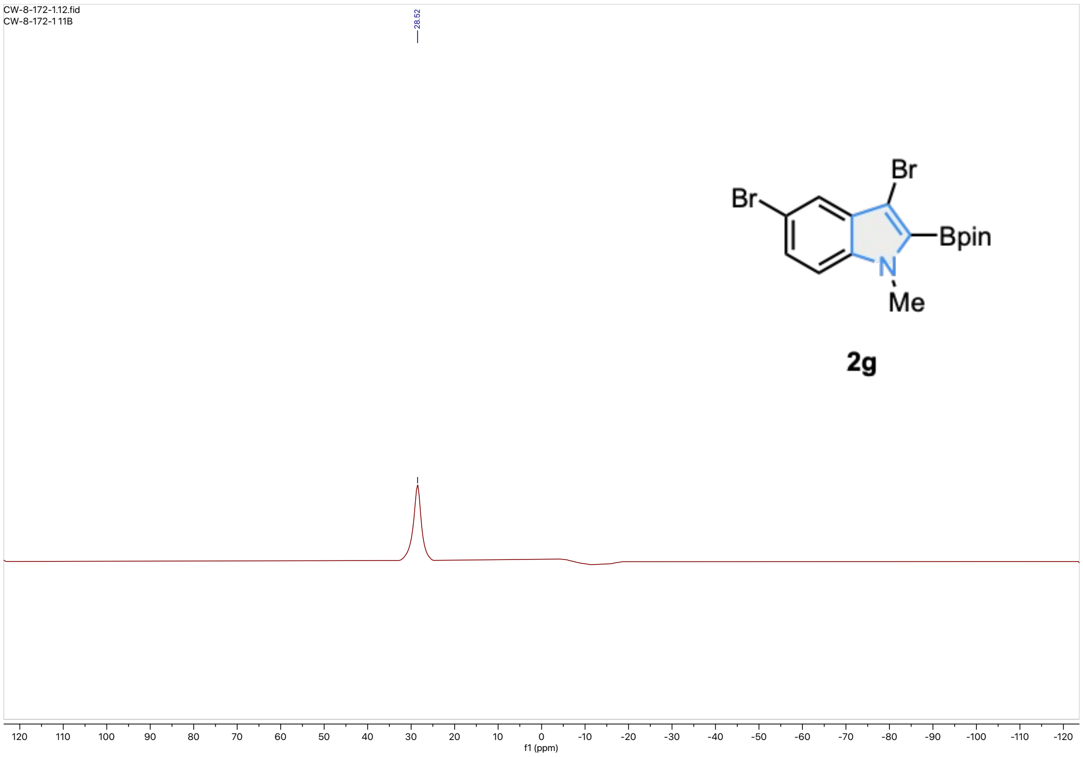

^{1}H NMR for **2h** (500 MHz, $CDCl_3$)

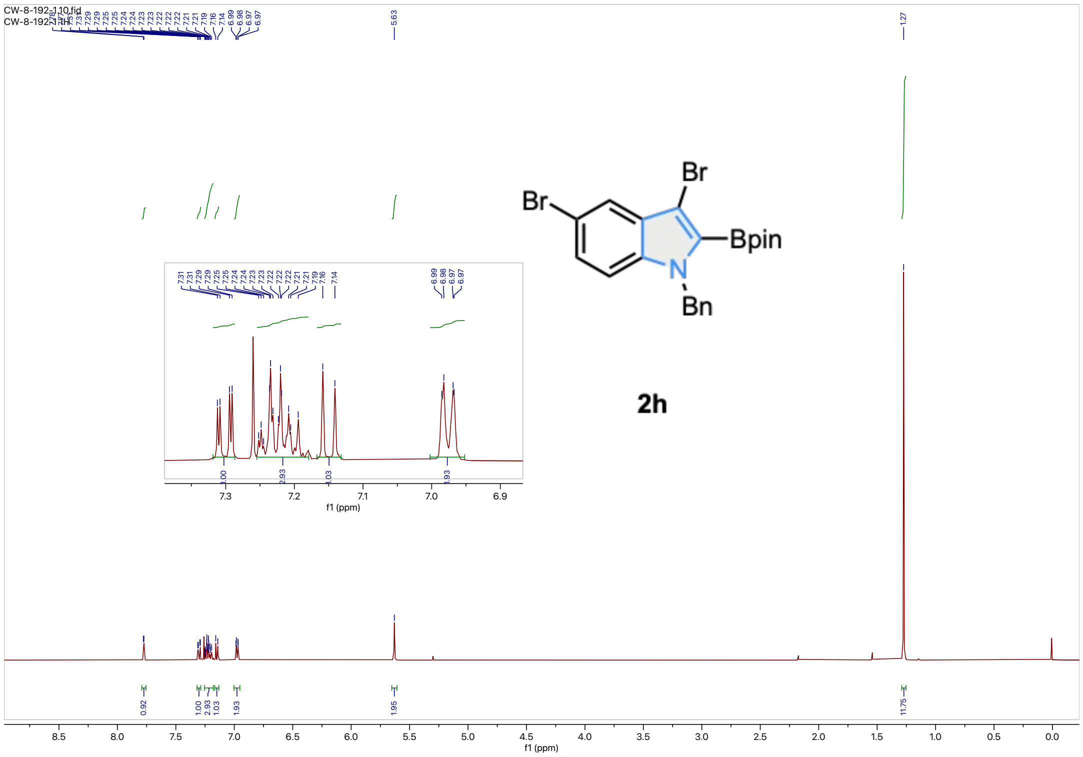

^{13}C NMR for **2h** (126 MHz, $CDCl_3$)

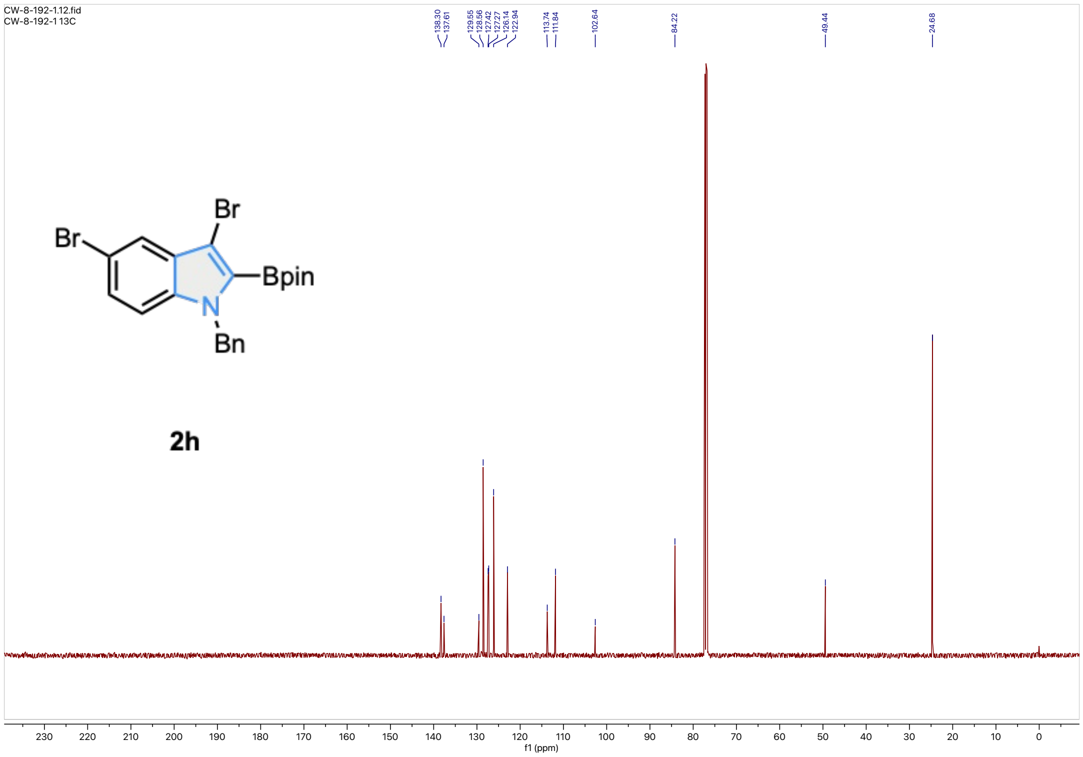

^{11}B NMR for **2h** (160 MHz, $CDCl_3$)

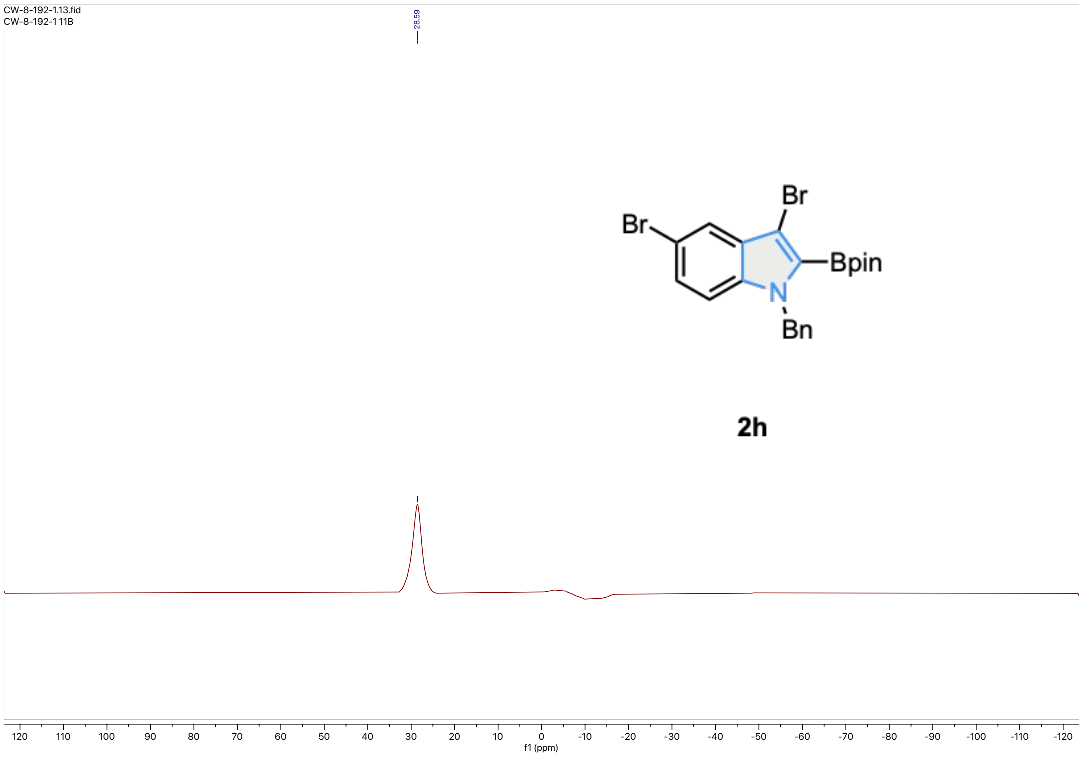

^{1}H NMR for **2j** (500 MHz, $CDCl_3$)

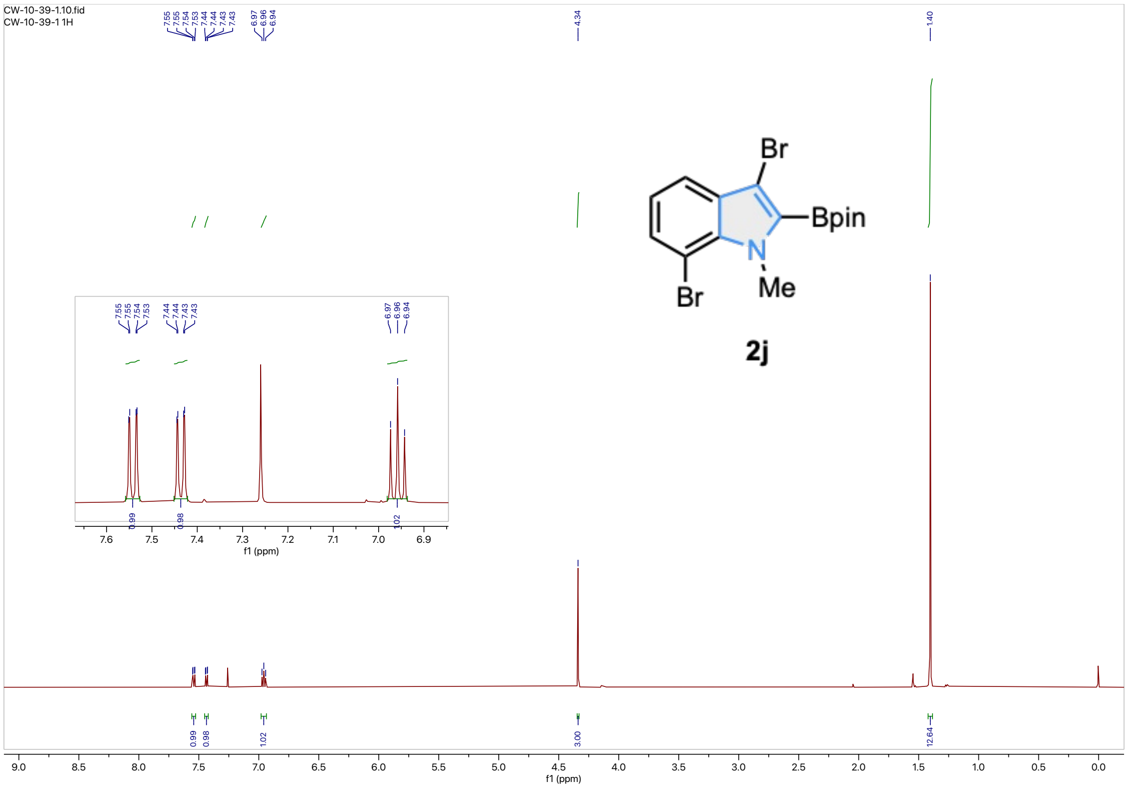

^{13}C NMR for **2j** (126 MHz, $CDCl_3$)

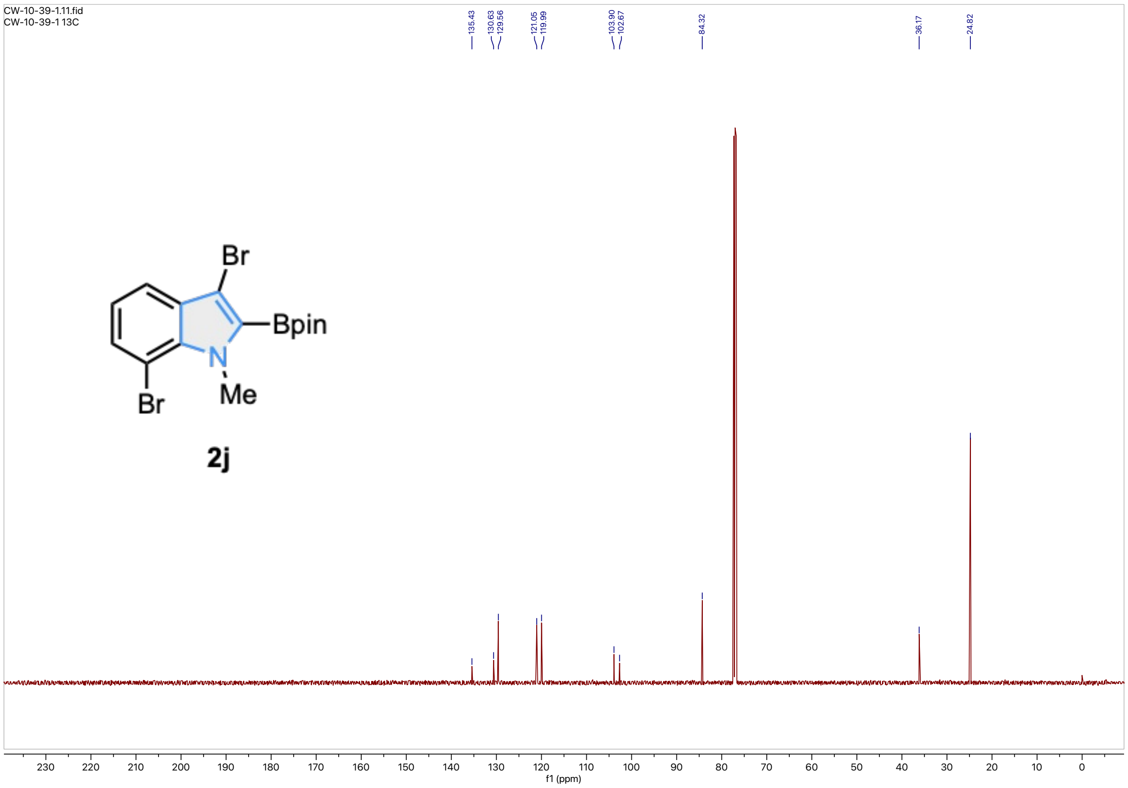

^{11}B NMR for **2j** (160 MHz, $CDCl_3$)

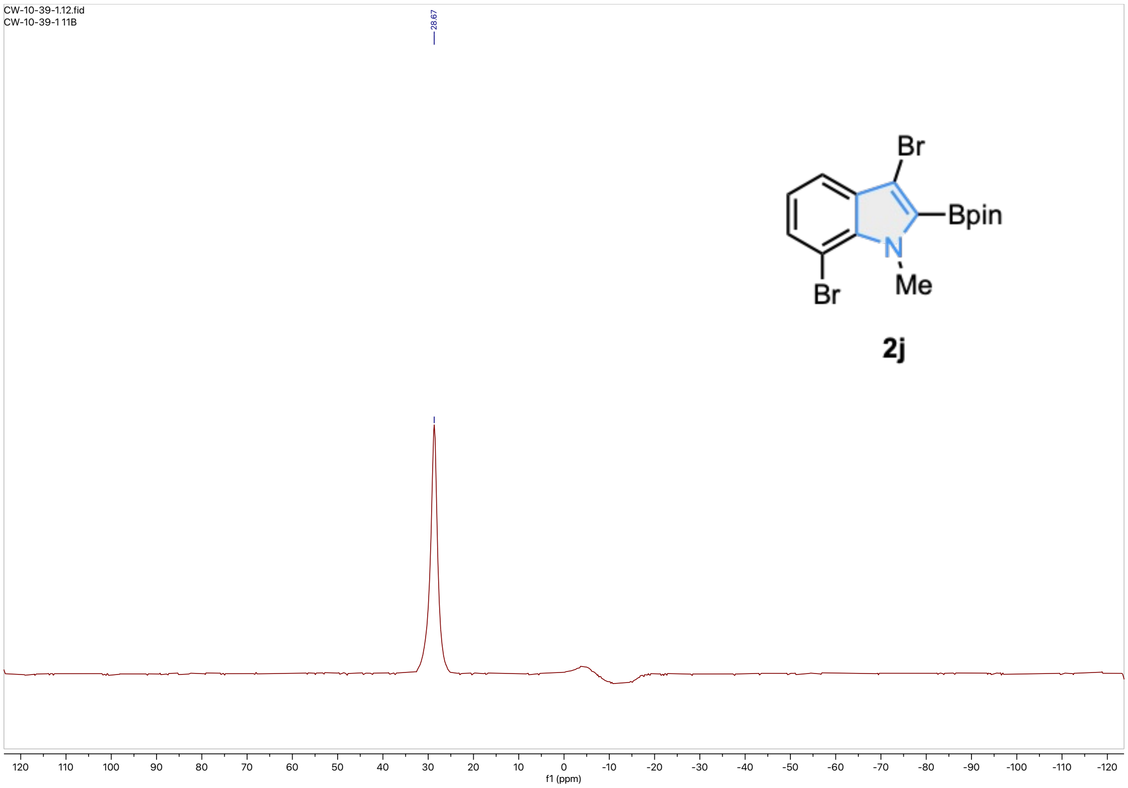

^{1}H NMR for **2k** (600 MHz, $CDCl_3$)

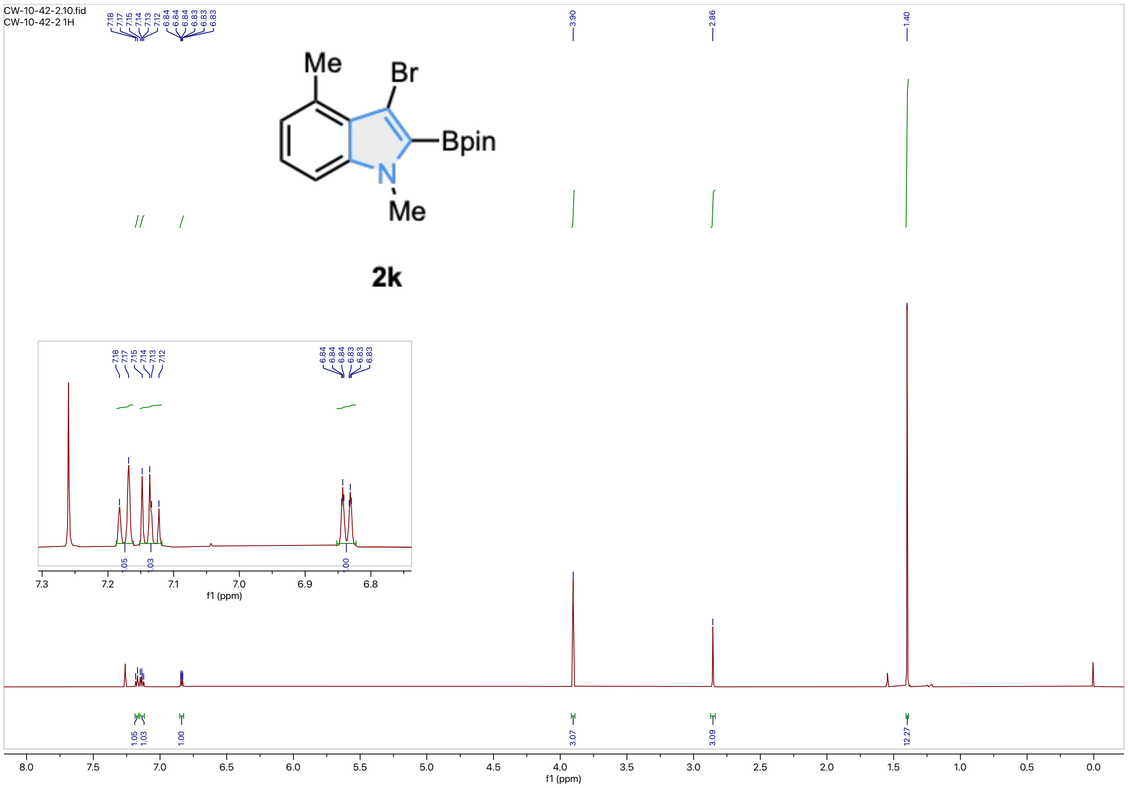

^{13}C NMR for **2k** (151 MHz, $CDCl_3$)

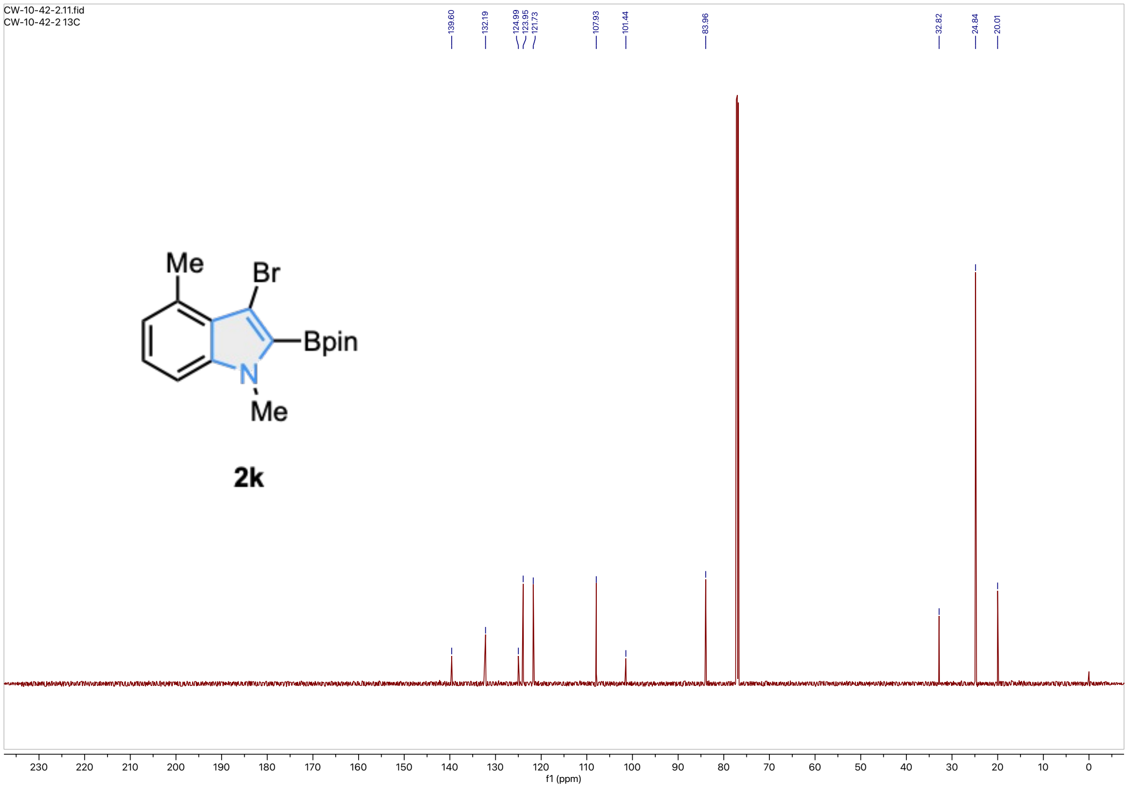

^{11}B NMR for **2k** (193 MHz, $CDCl_3$)

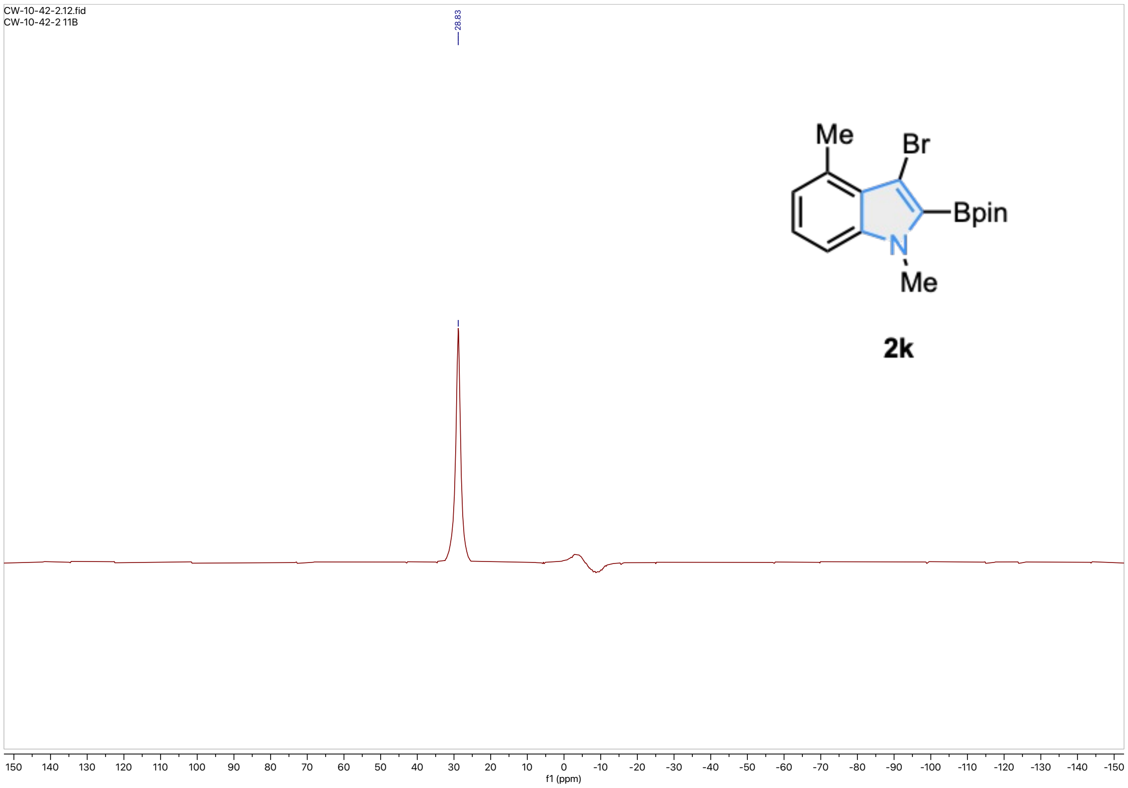

^{1}H NMR for **2l** (500 MHz, $CDCl_3$)

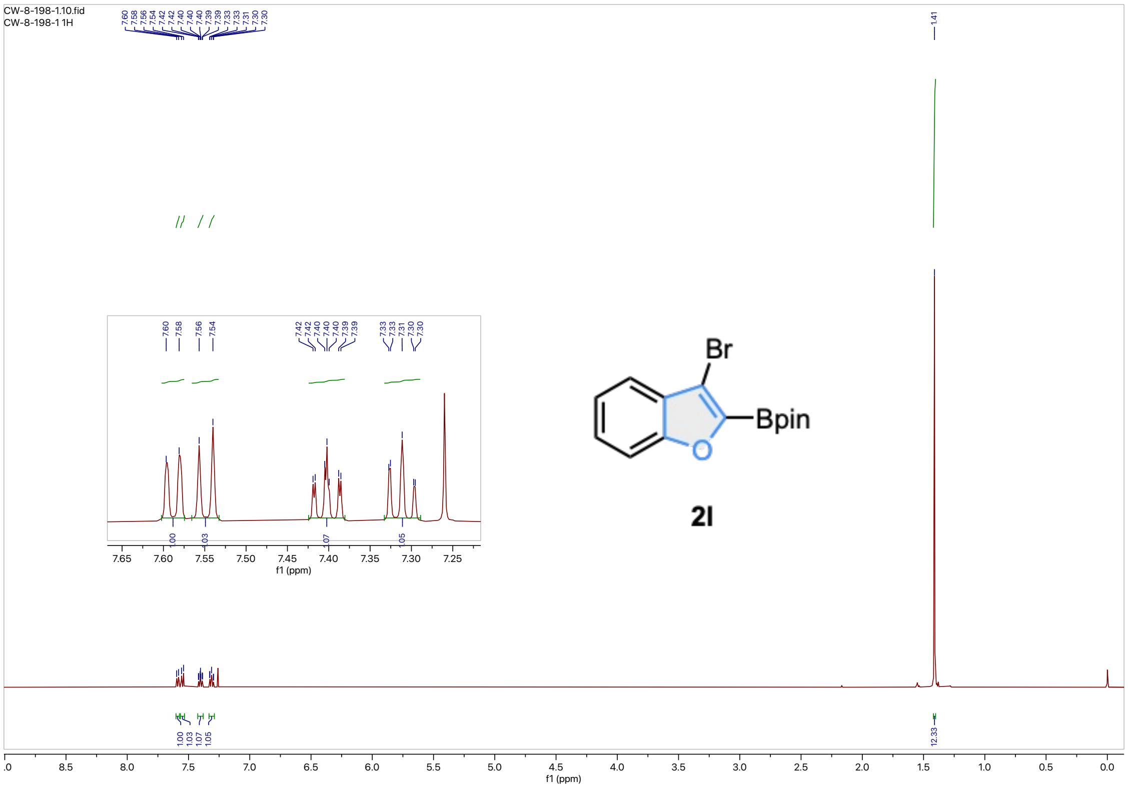

^{13}C NMR for **2l** (126 MHz, $CDCl_3$)

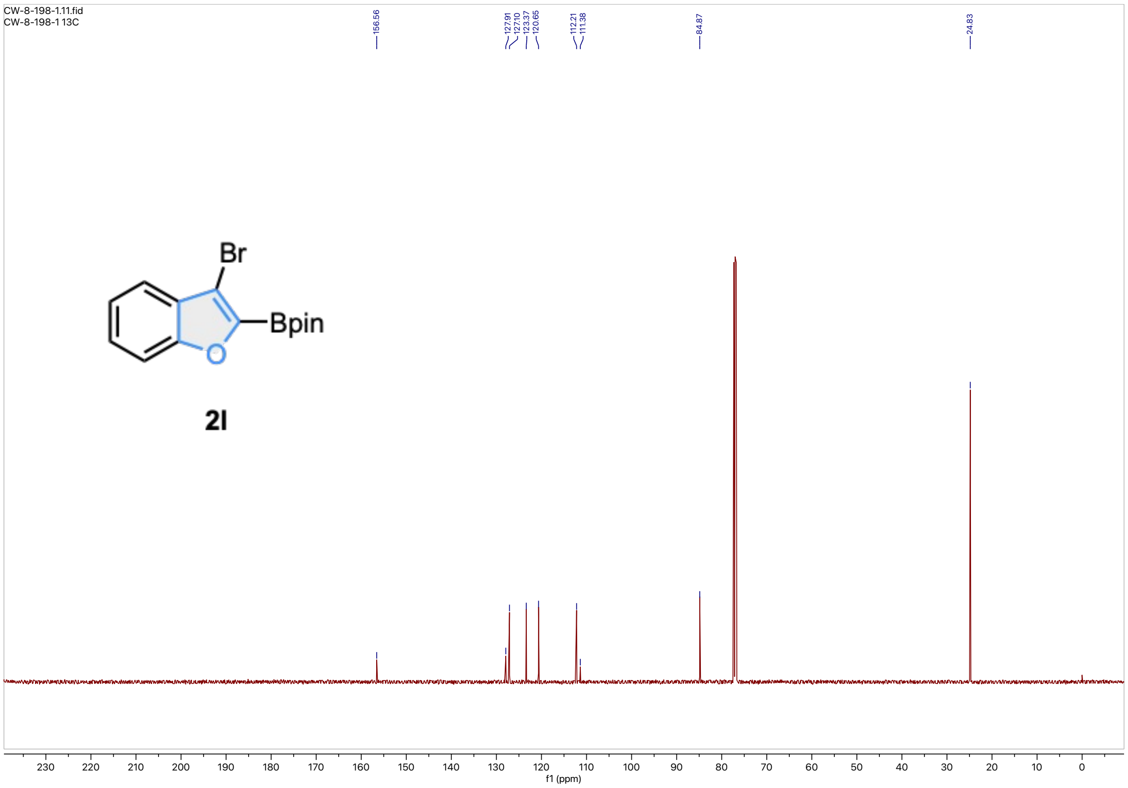

^{11}B NMR for **2l** (160 MHz, $CDCl_3$)

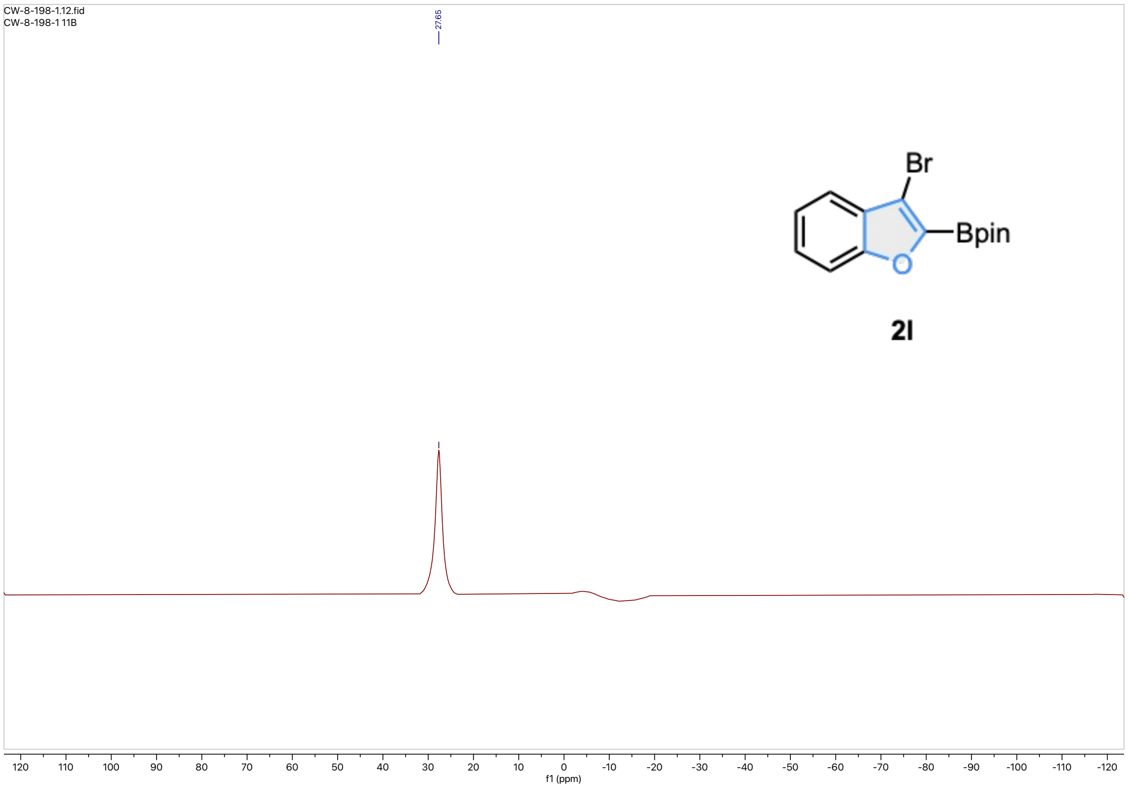

^{1}H NMR for **3a** (600 MHz, CDCl$_3$)

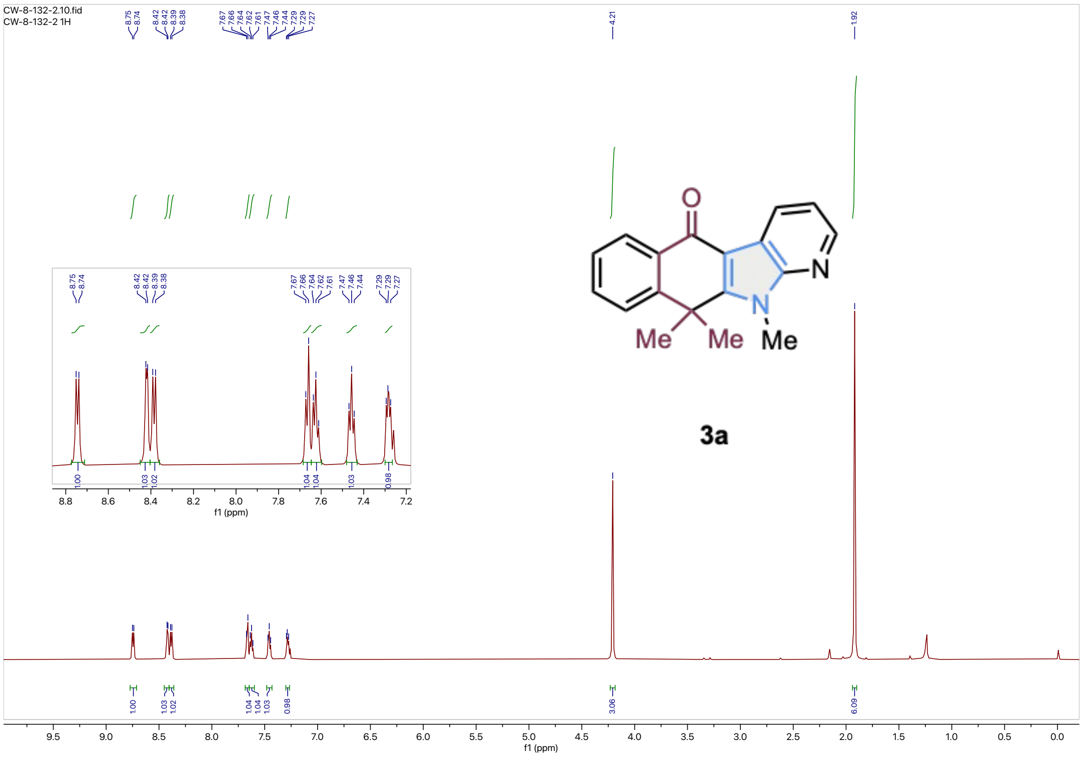

^{13}C NMR for **3a** (151 MHz, CDCl$_3$)

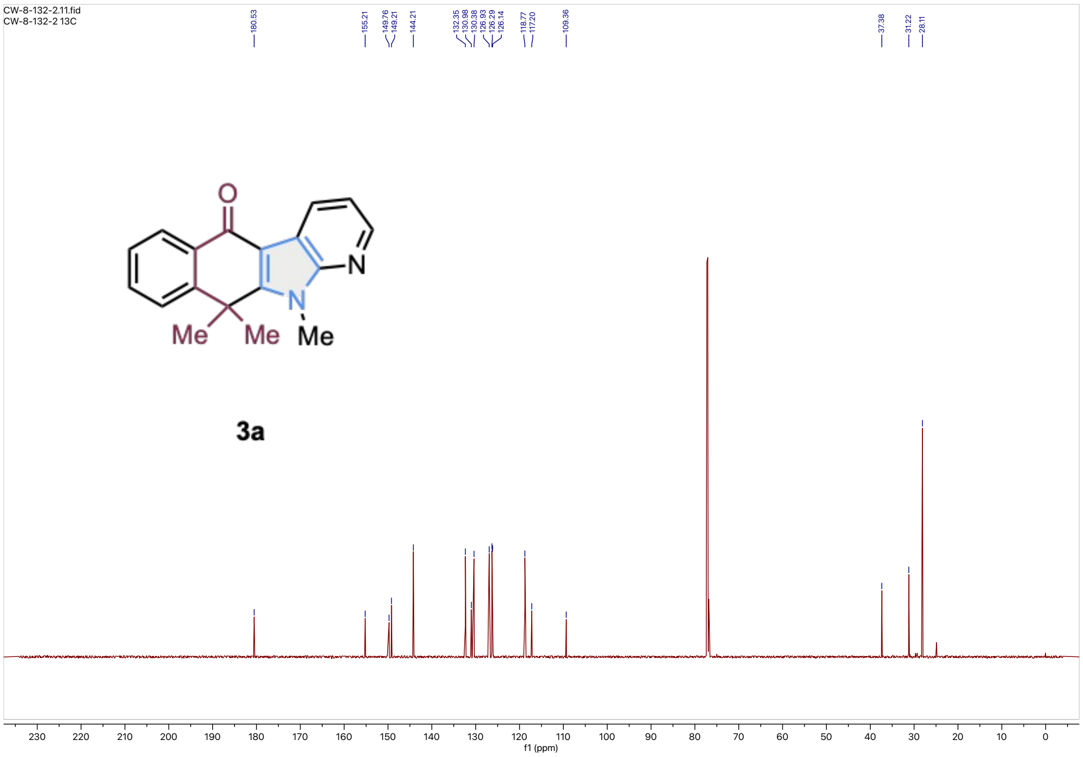

^{1}H NMR for **3b** (500 MHz, $CDCl_3$)

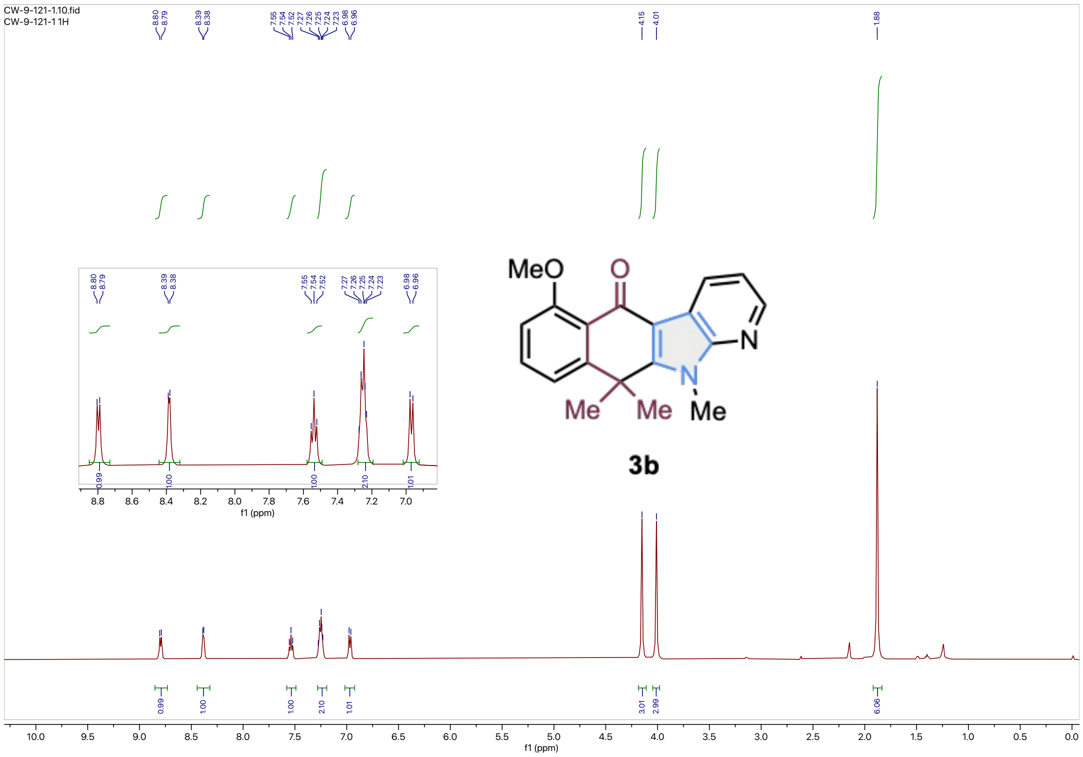

^{13}C NMR for **3b** (126 MHz, $CDCl_3$)

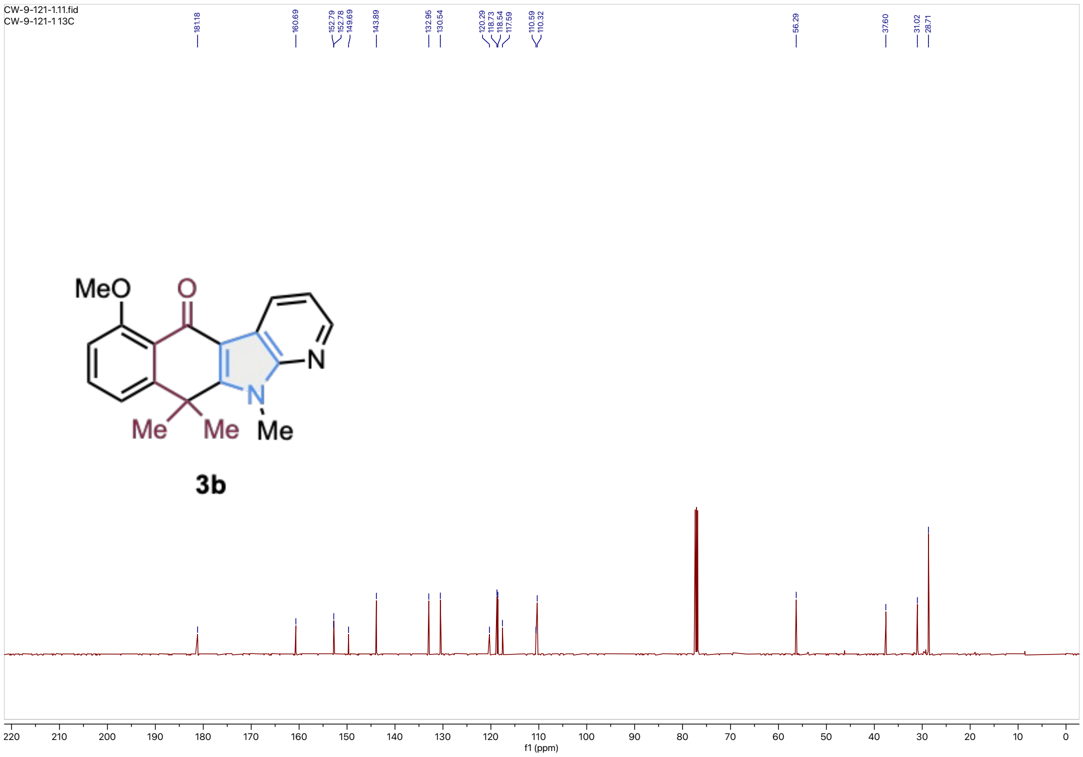

^{1}H NMR for **3c** (500 MHz, $CDCl_3$)

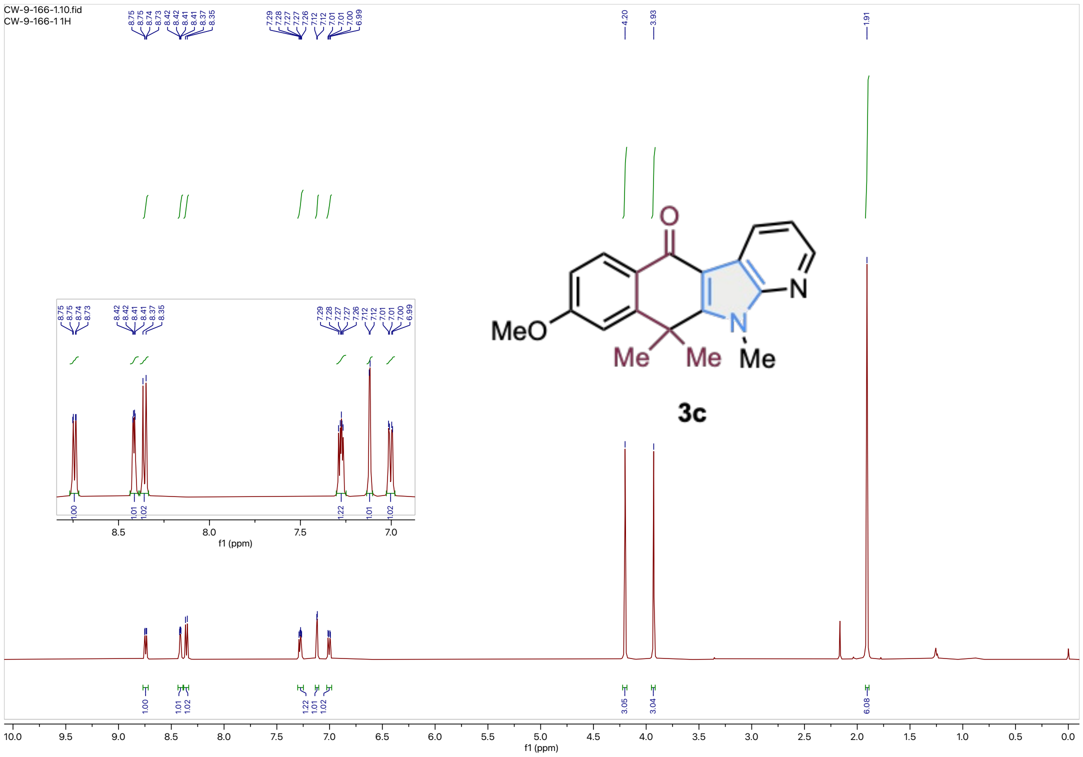

^{13}C NMR for **3c** (126 MHz, $CDCl_3$)

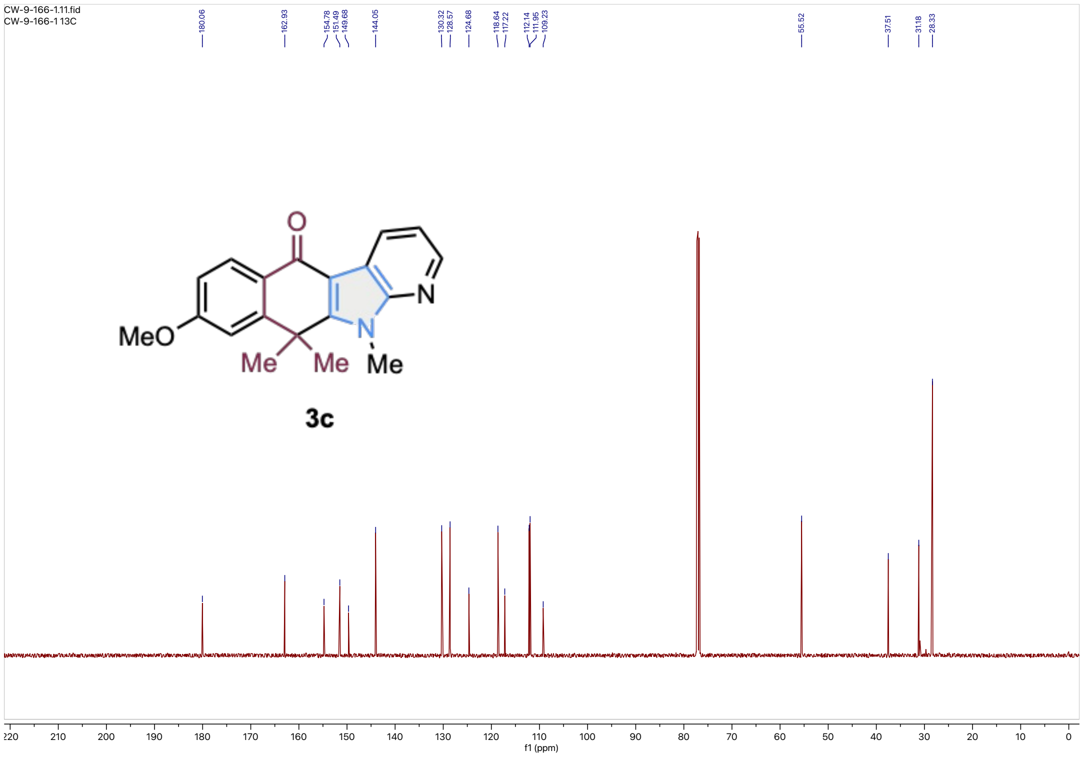

^{1}H NMR for **3d** (500 MHz, $CDCl_3$)

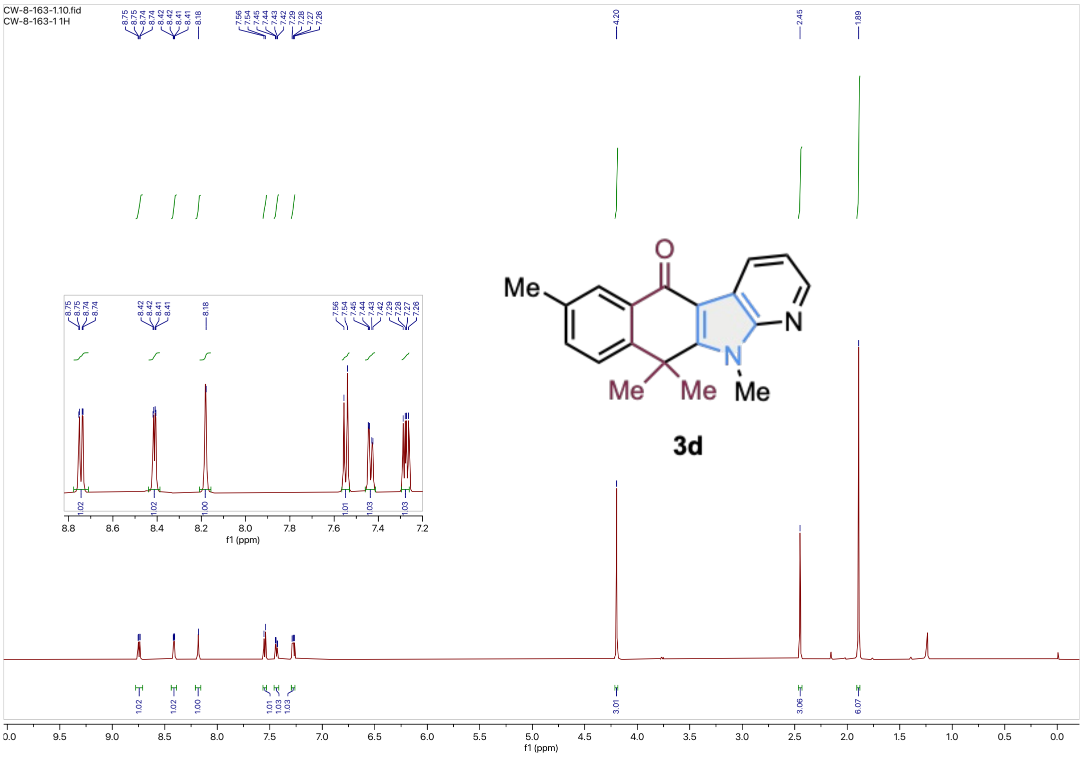

^{13}C NMR for **3d** (126 MHz, $CDCl_3$)

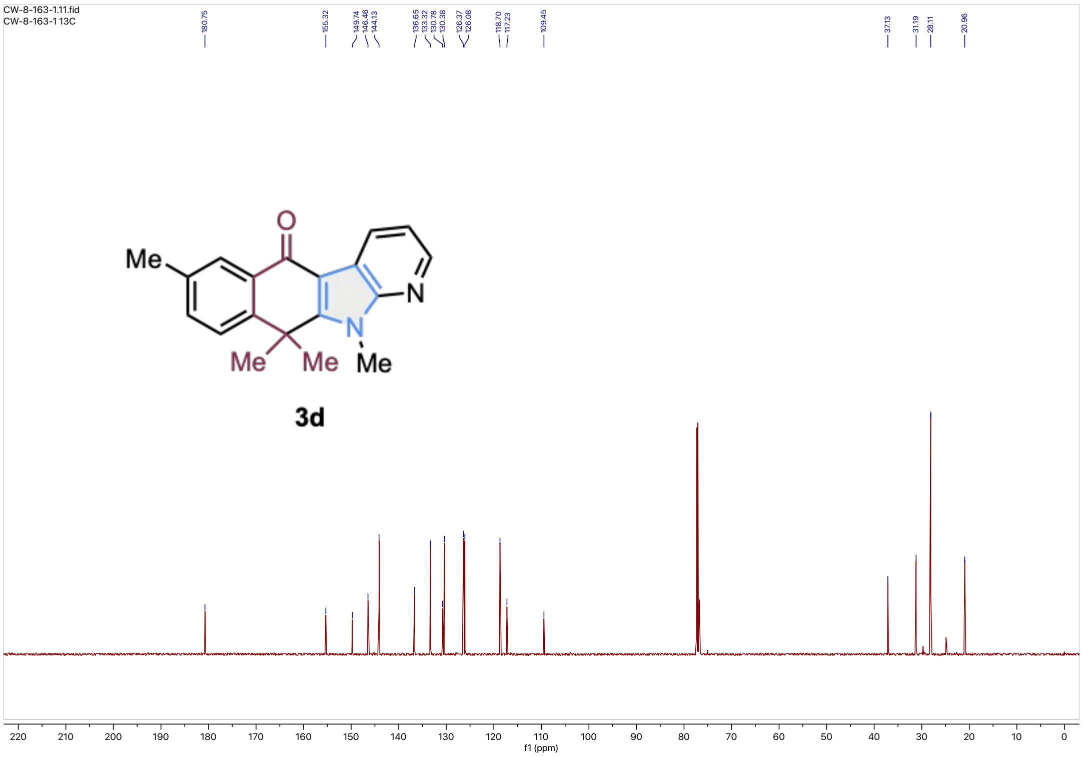

^{1}H NMR for **3e** (500 MHz, $CDCl_3$)

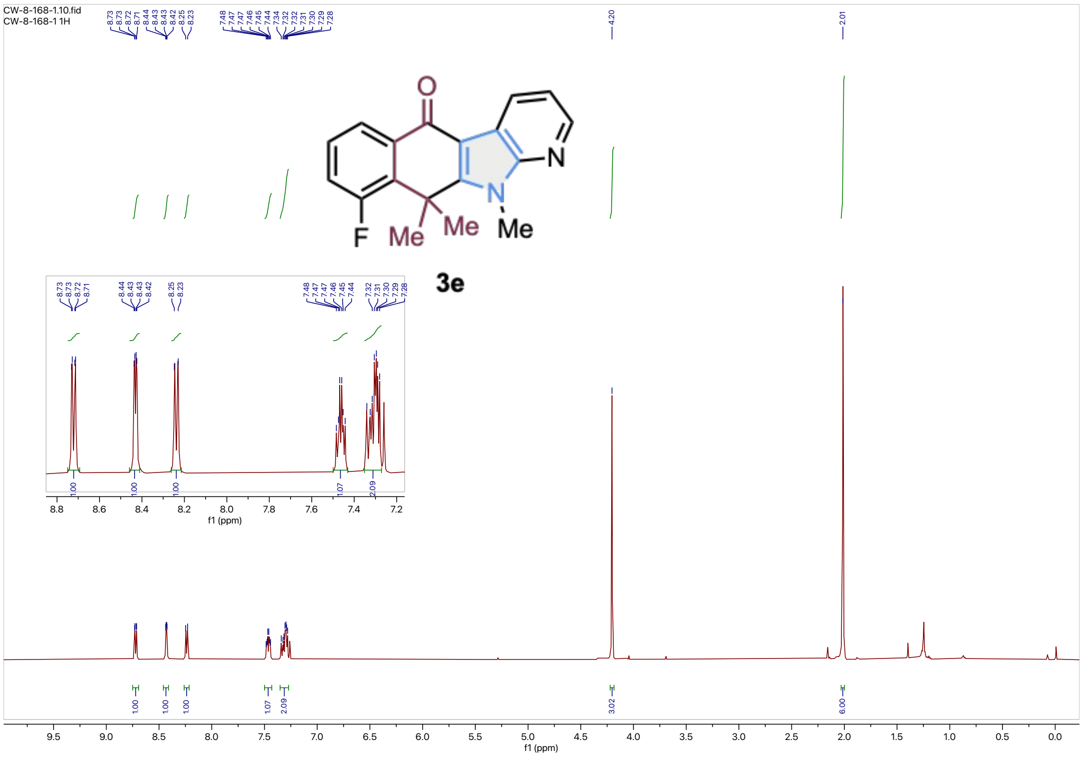

^{13}C NMR for **3e** (126 MHz, $CDCl_3$)

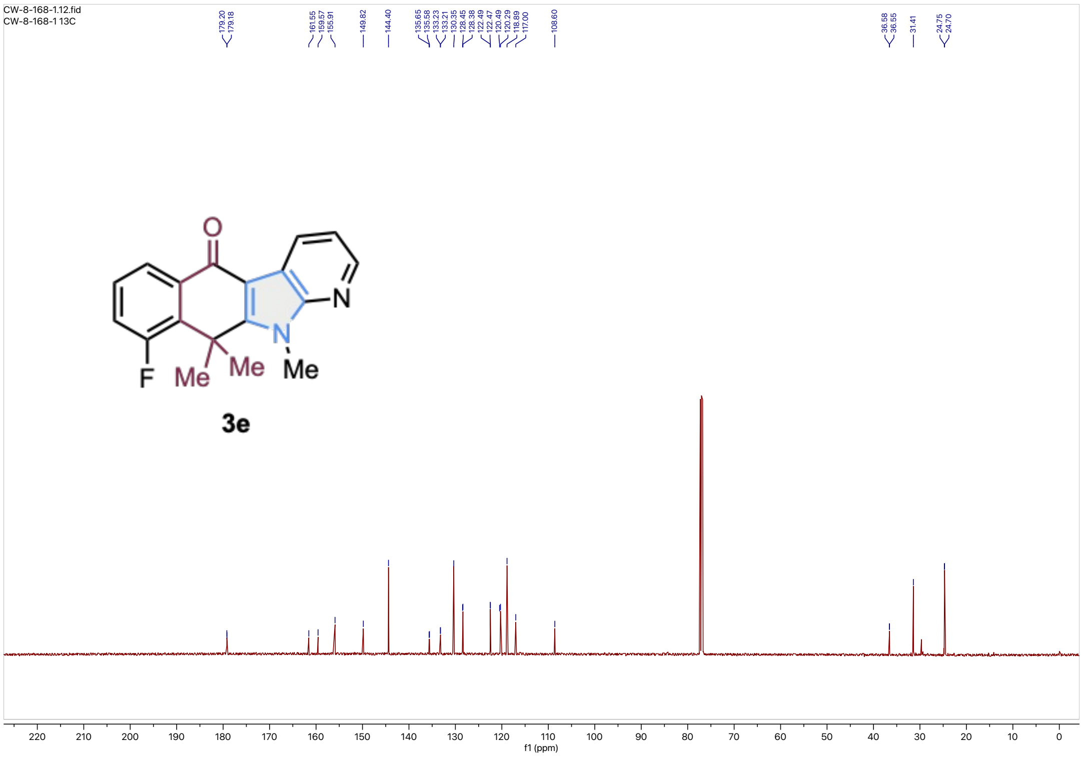

^{19}F NMR for **3e** (470 MHz, $CDCl_3$)

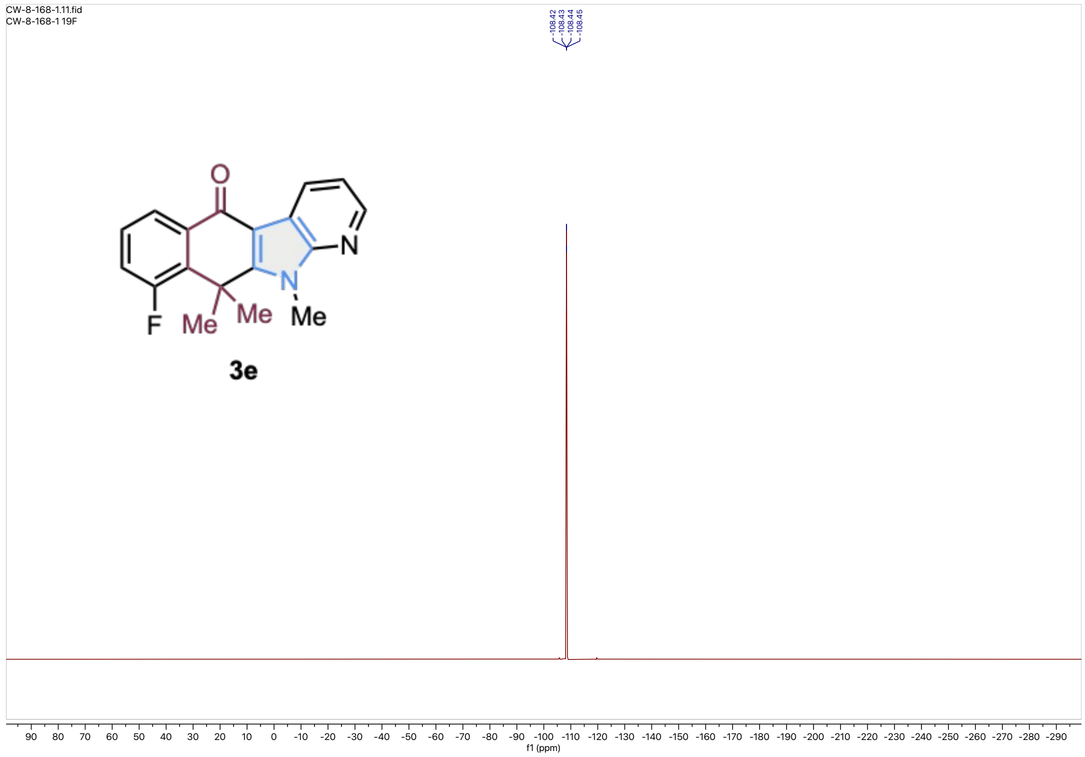

^{1}H NMR for **3f** (500 MHz, $CDCl_3$)

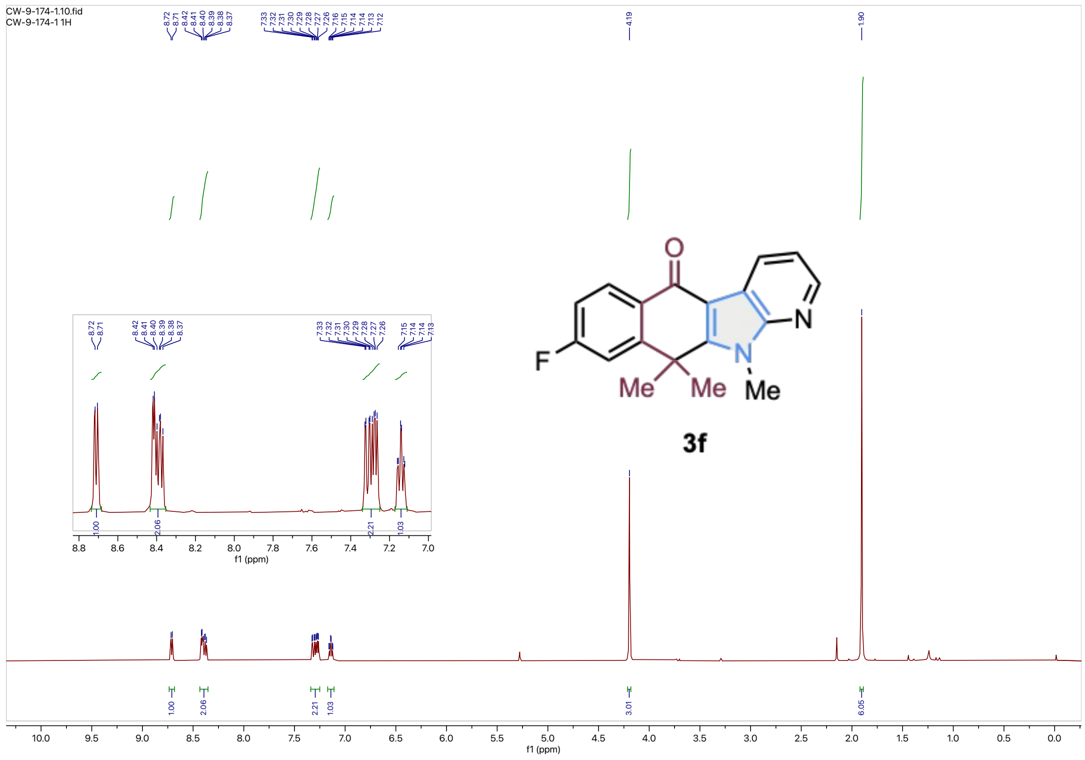

^{13}C NMR for **3f** (126 MHz, $CDCl_3$)

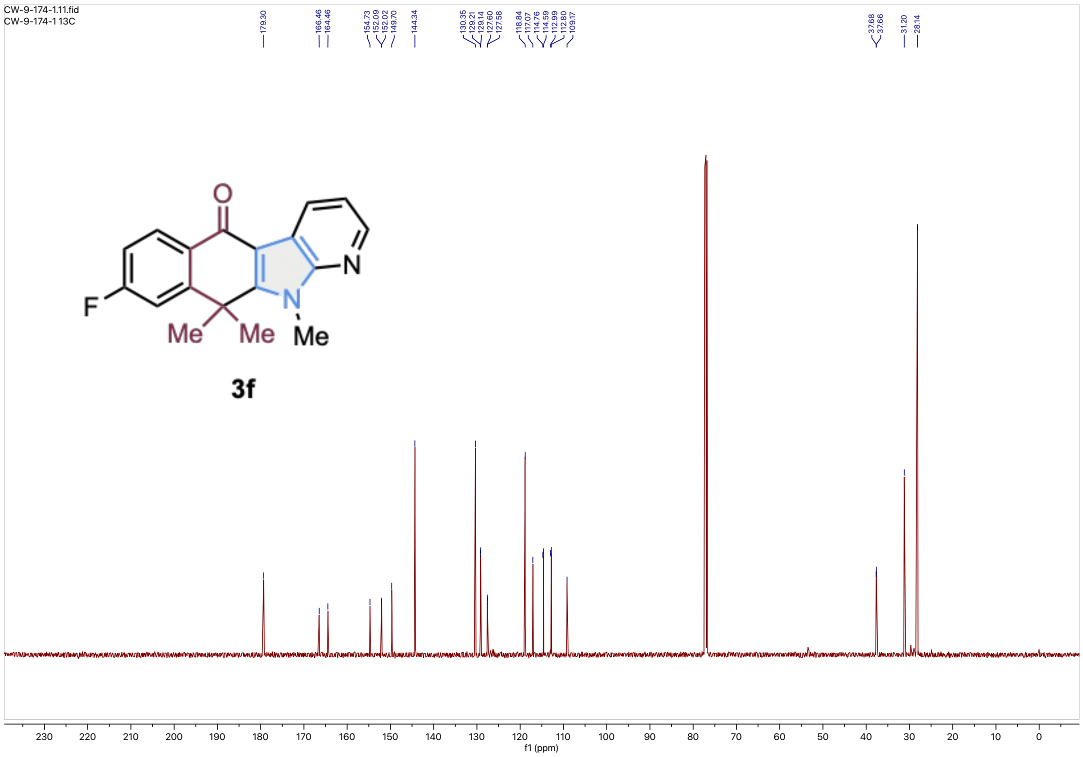

^{19}F NMR for **3f** (470 MHz, $CDCl_3$)

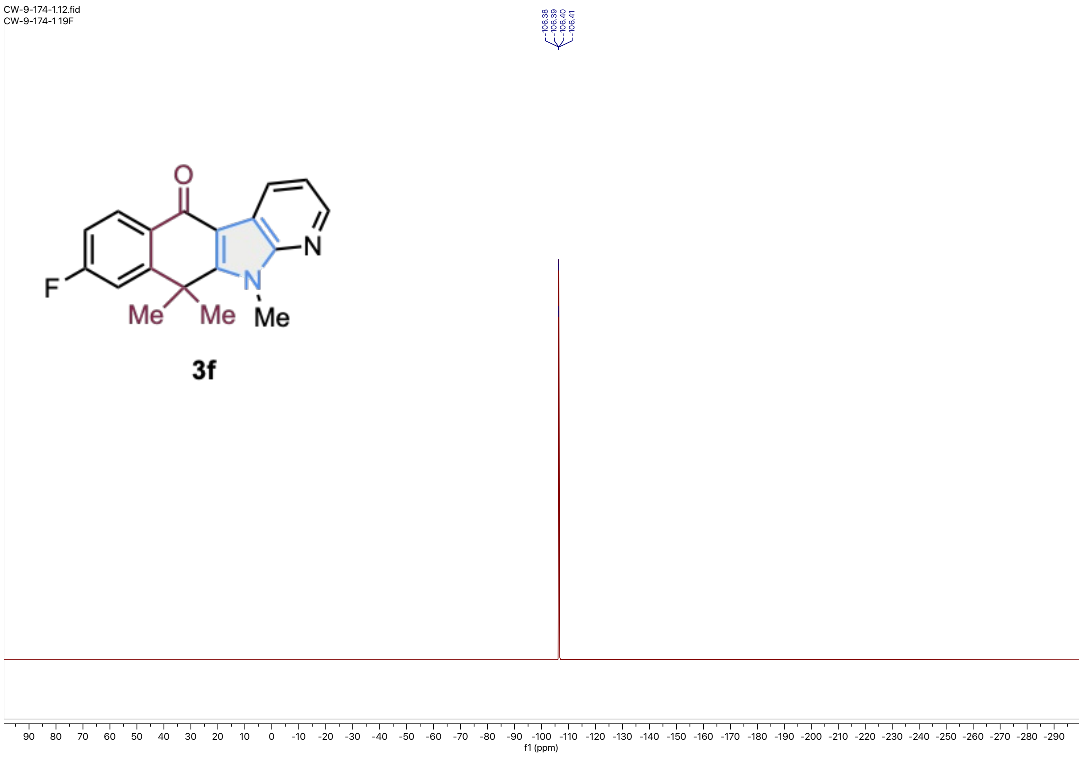

^{1}H NMR for **3g**, ~87% purity (500 MHz, $CDCl_3$)

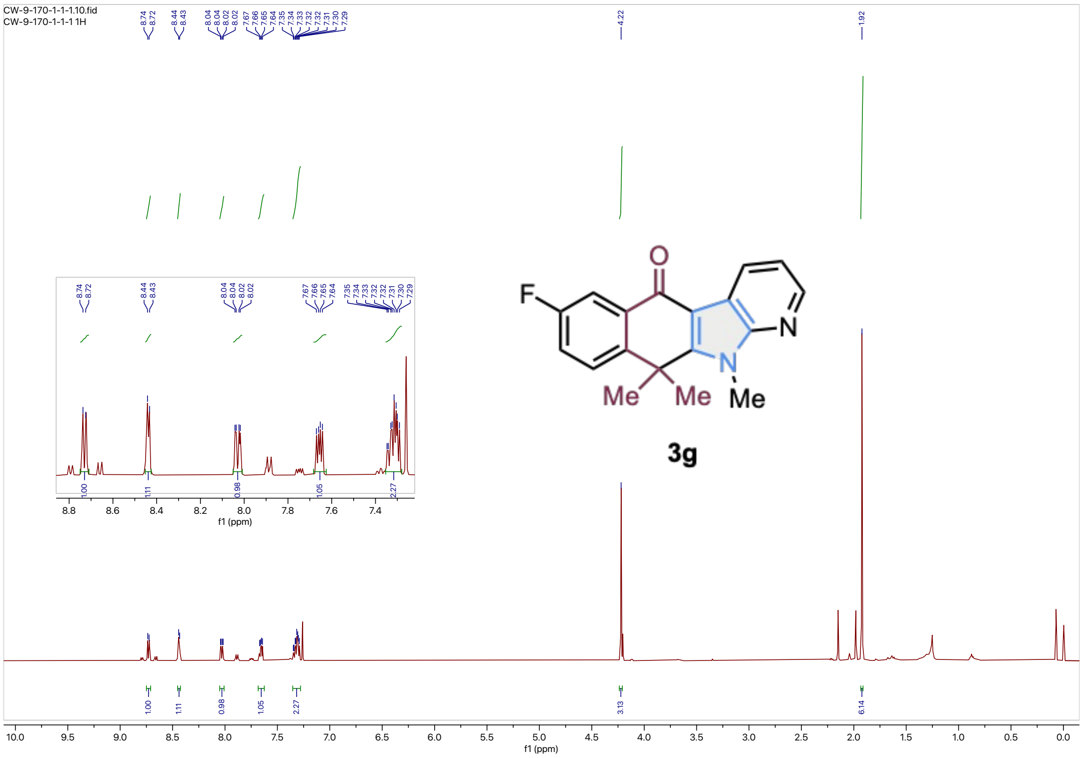

^{13}C NMR for **3g**, ~87% purity (126 MHz, $CDCl_3$)

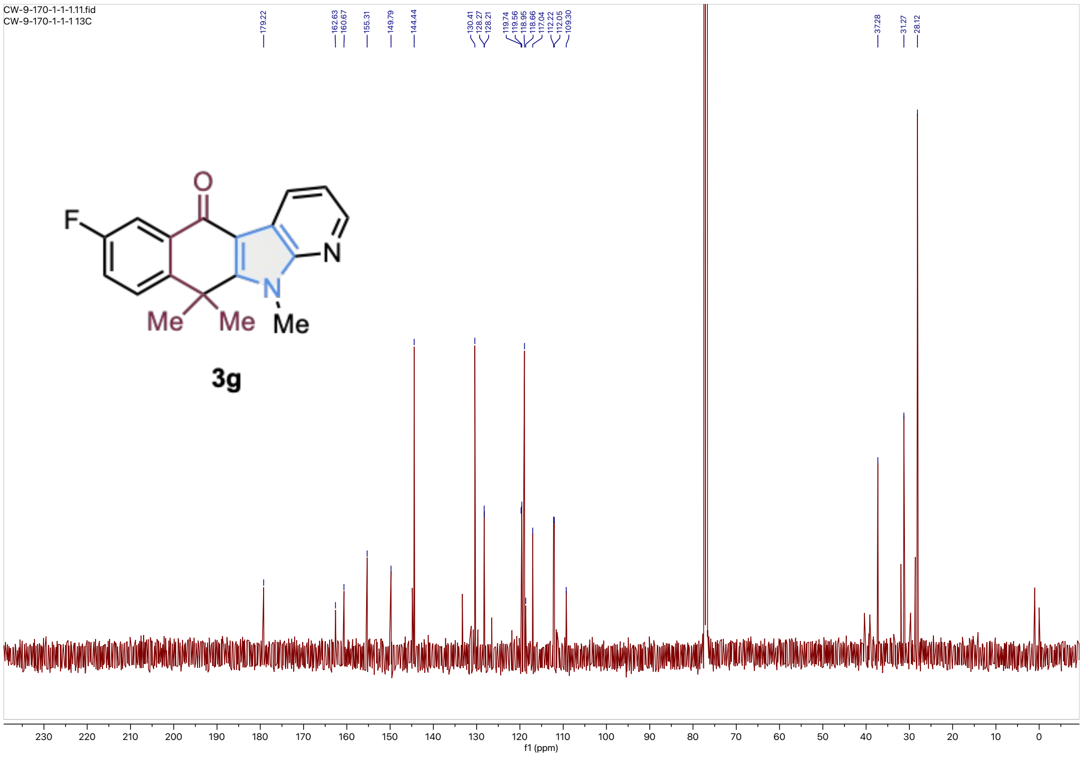

^{19}F NMR for **3g**, ~87% purity (470 MHz, $CDCl_3$)

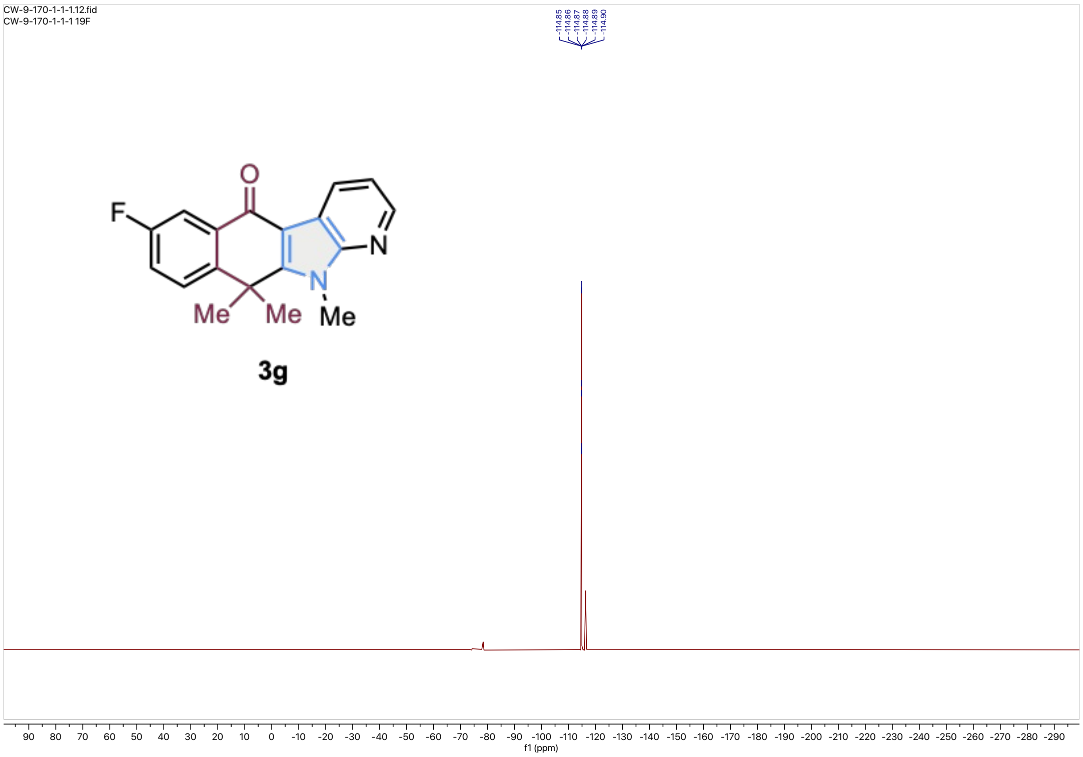

^{1}H NMR for **3h** (600 MHz, $CDCl_3$)

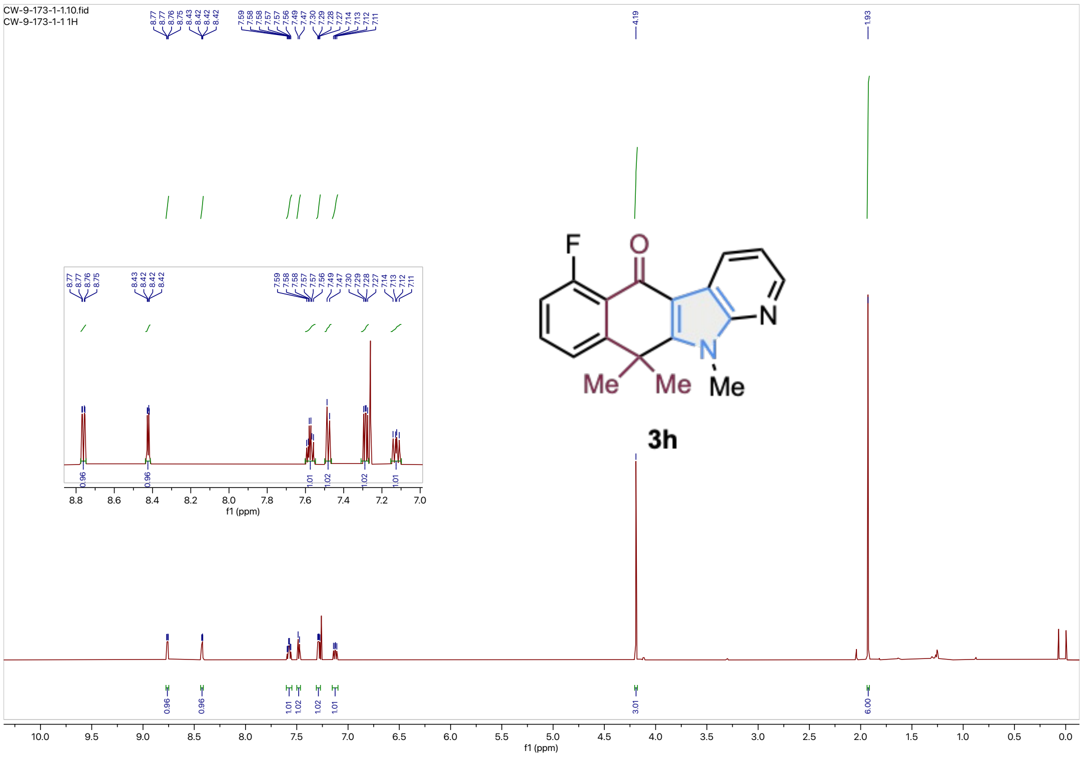

^{13}C NMR for **3h** (151 MHz, $CDCl_3$)

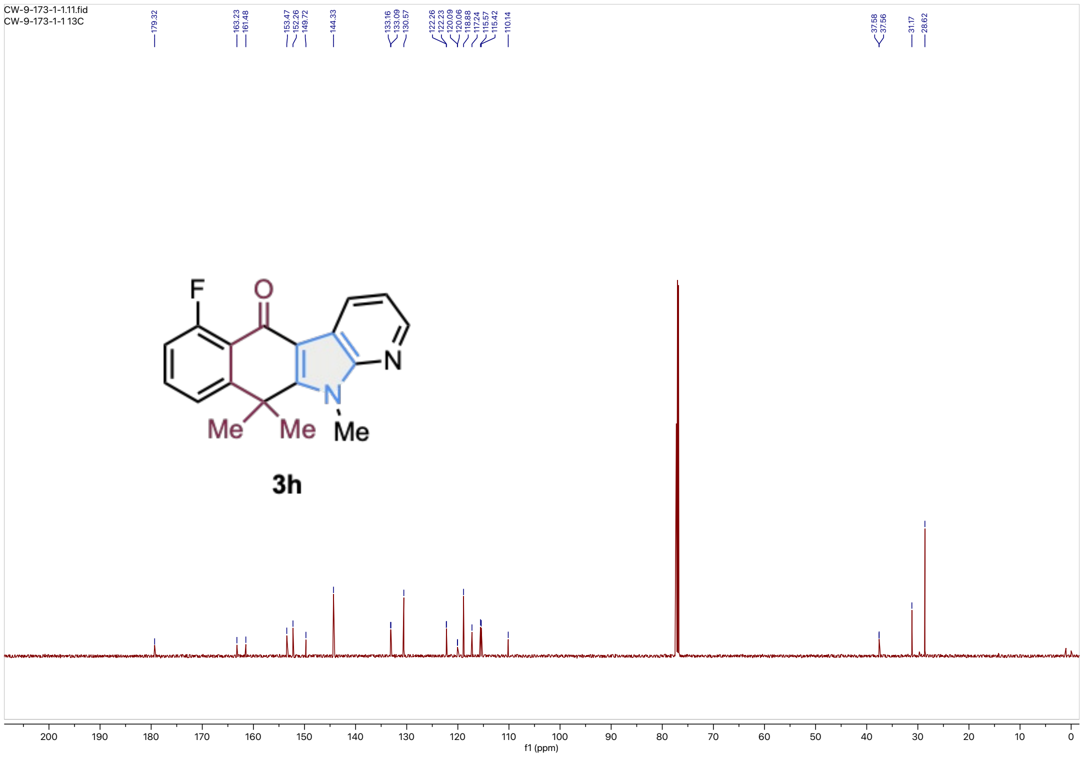

^{19}F NMR for **3h** (565 MHz, $CDCl_3$)

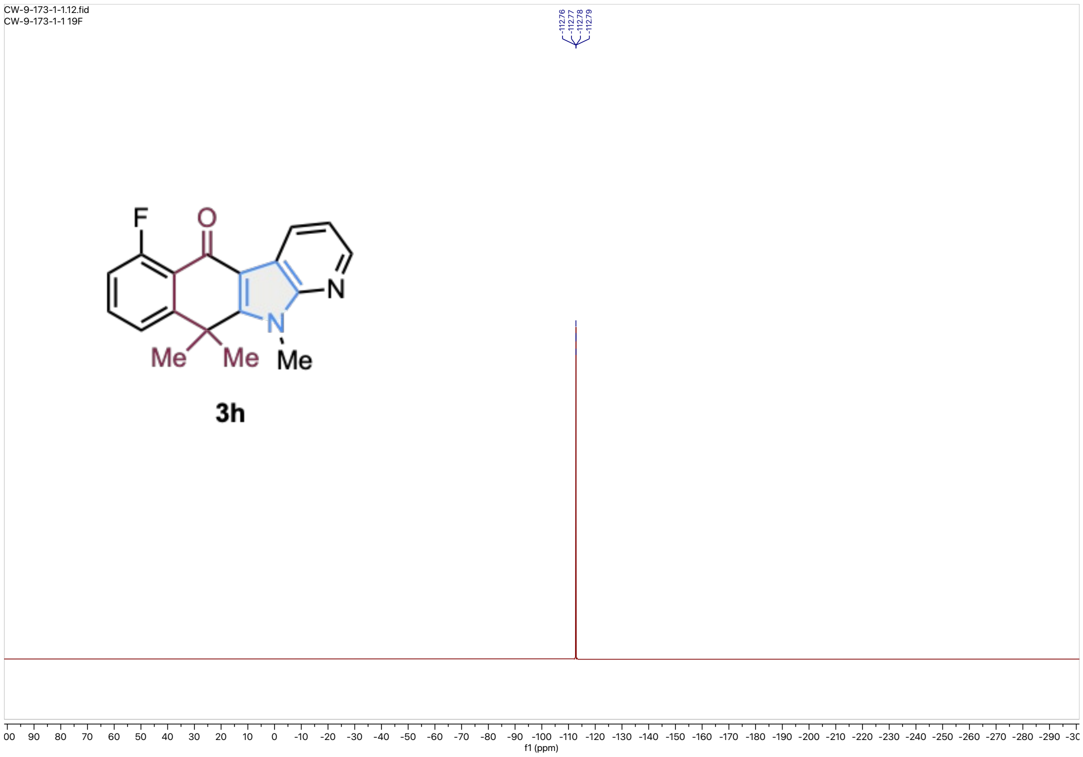

^{1}H NMR for **3i** (600 MHz, $CDCl_3$)

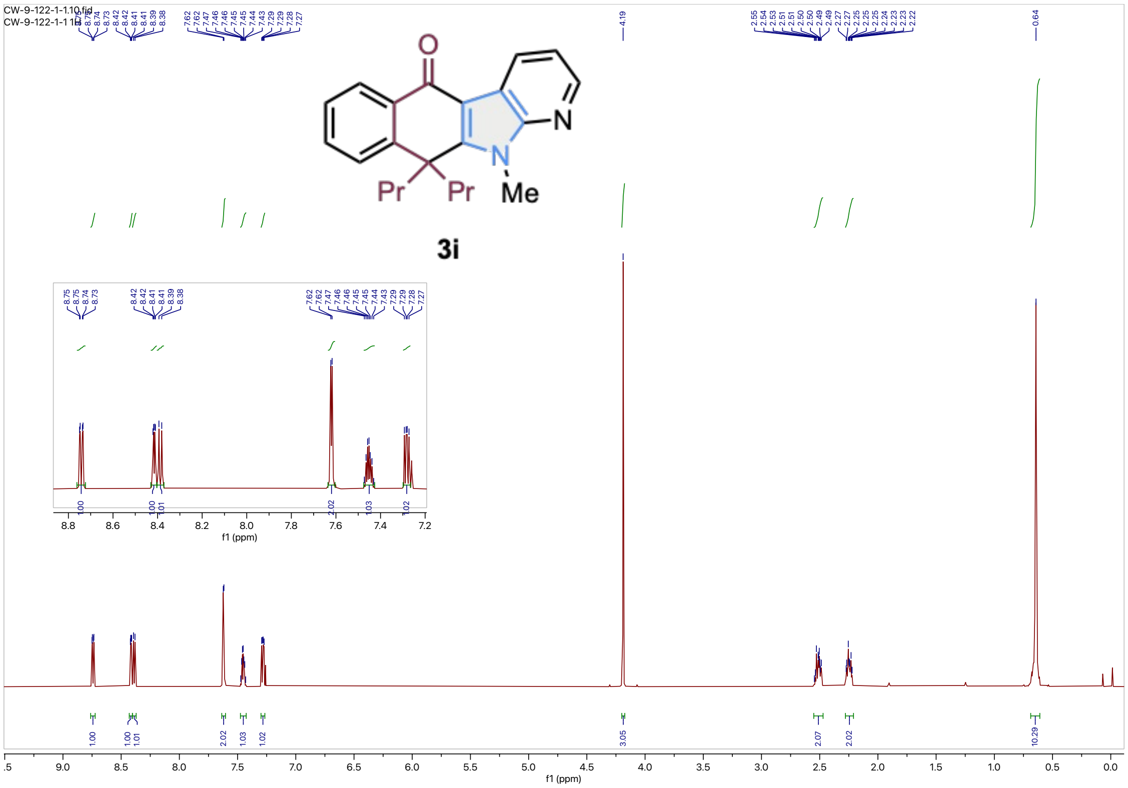

^{13}C NMR for **3i** (151 MHz, $CDCl_3$)

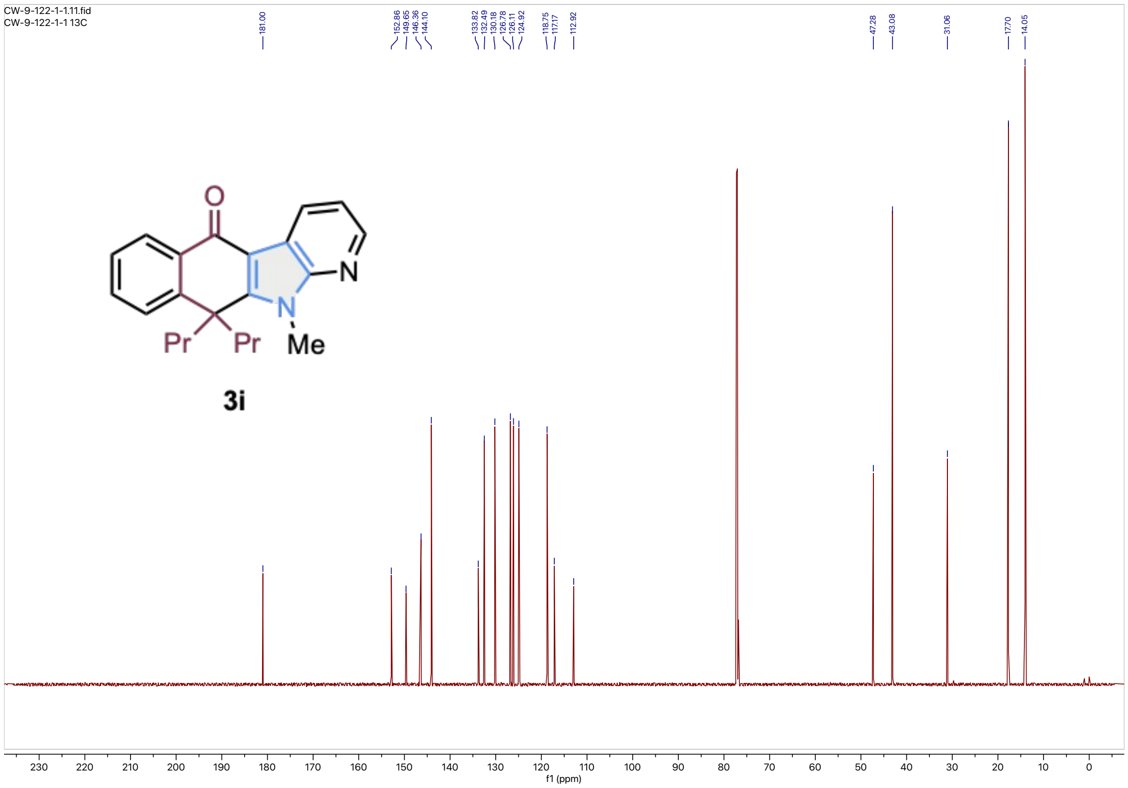

^{1}H NMR for **3j** (500 MHz, $CDCl_3$)

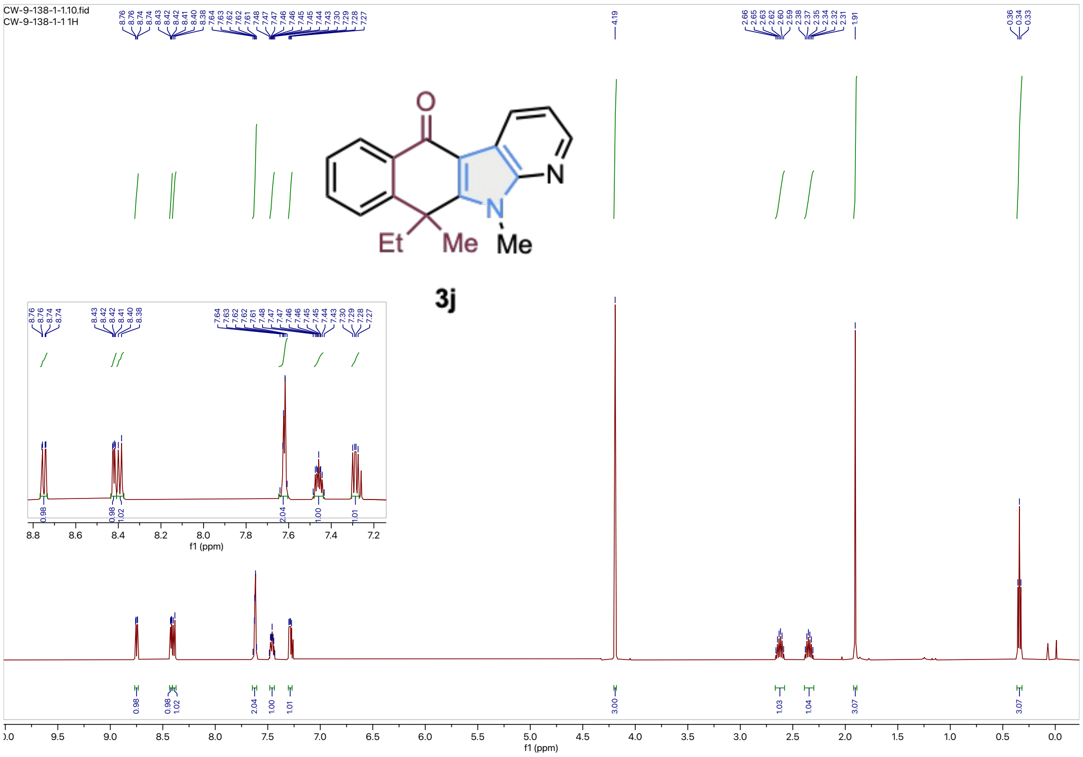

^{13}C NMR for **3j** (126 MHz, $CDCl_3$)

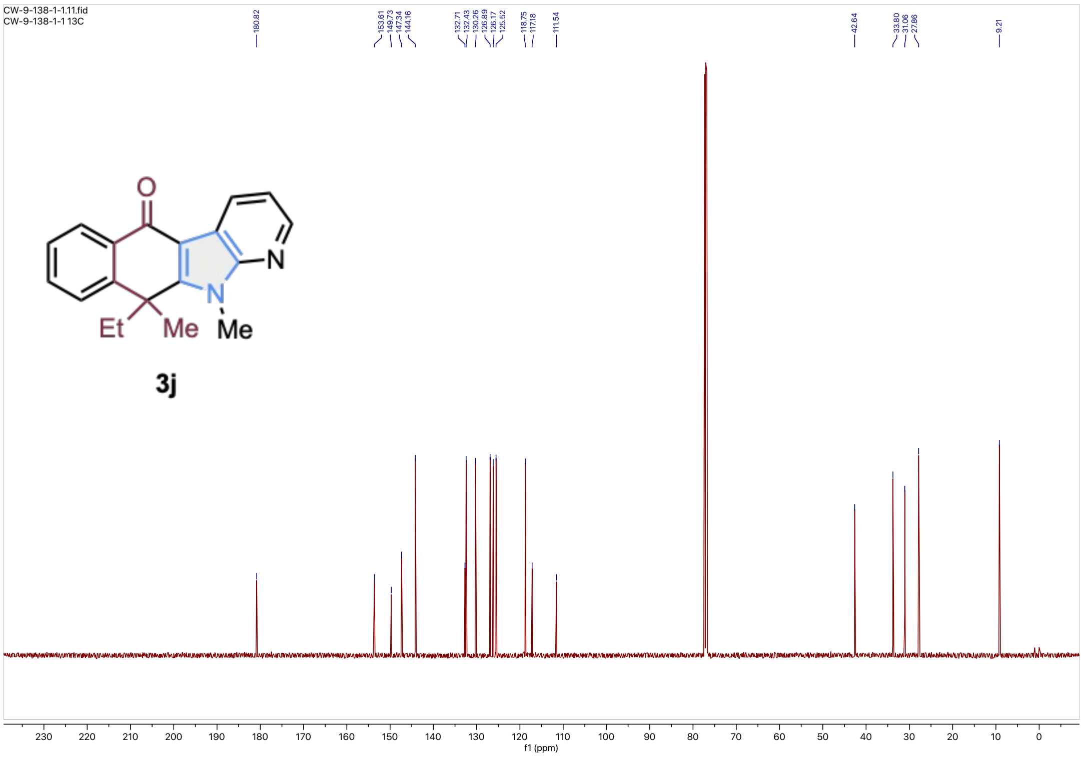

^{1}H NMR for **3k** (600 MHz, $CDCl_3$)

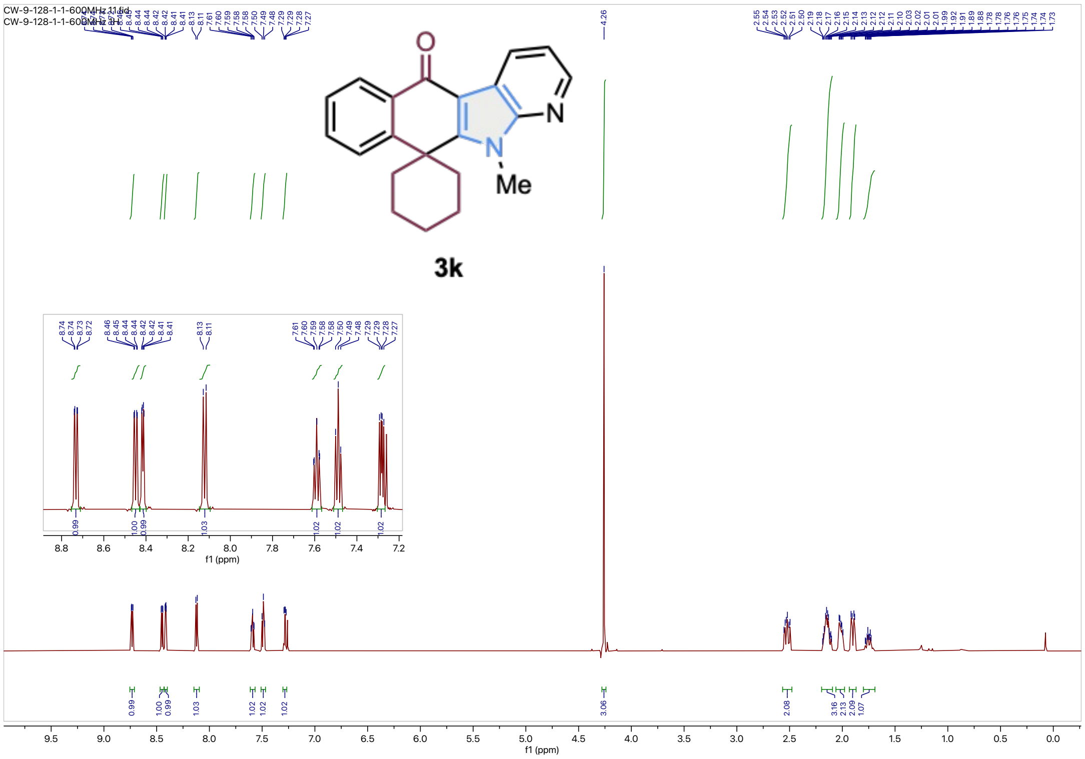

^{13}C NMR for **3k** (151 MHz, $CDCl_3$)

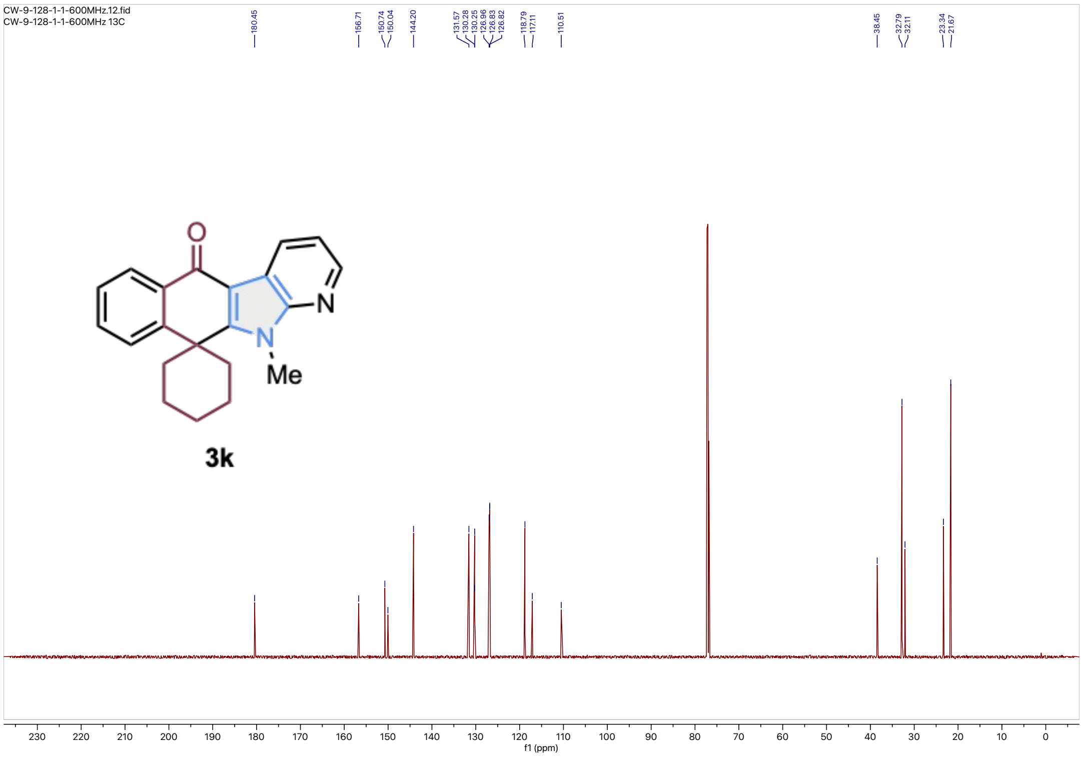

^{1}H NMR for **3l** (600 MHz, $CDCl_3$)

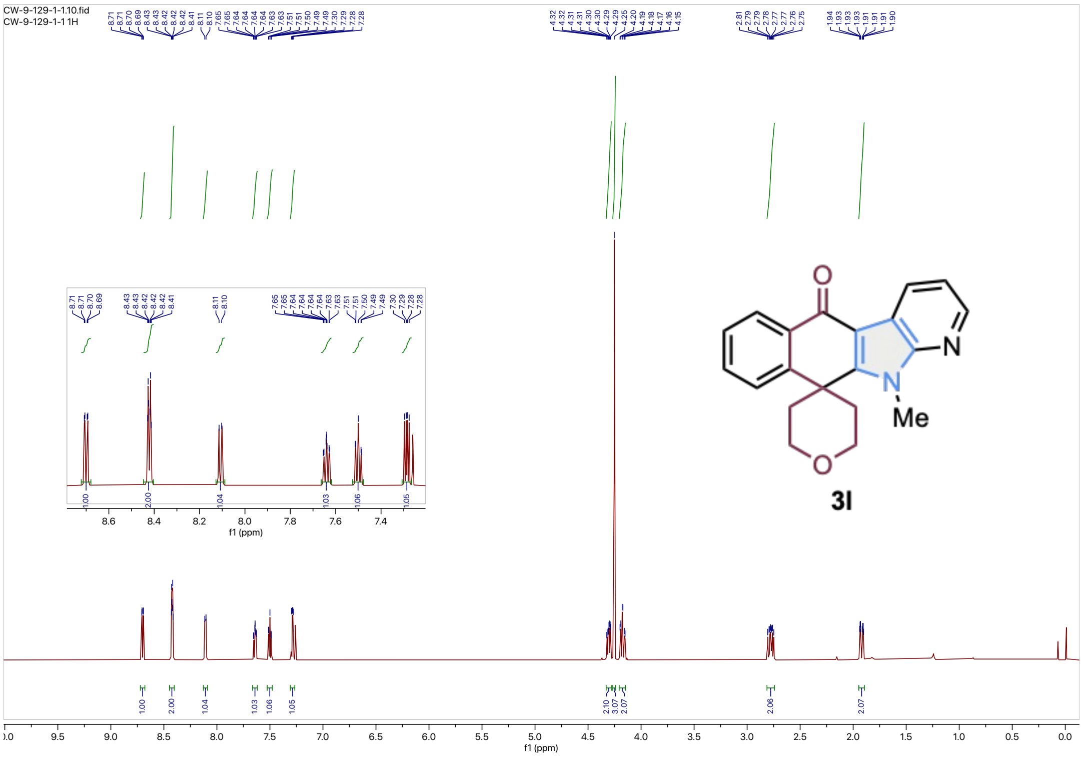

^{13}C NMR for **3l** (151 MHz, $CDCl_3$)

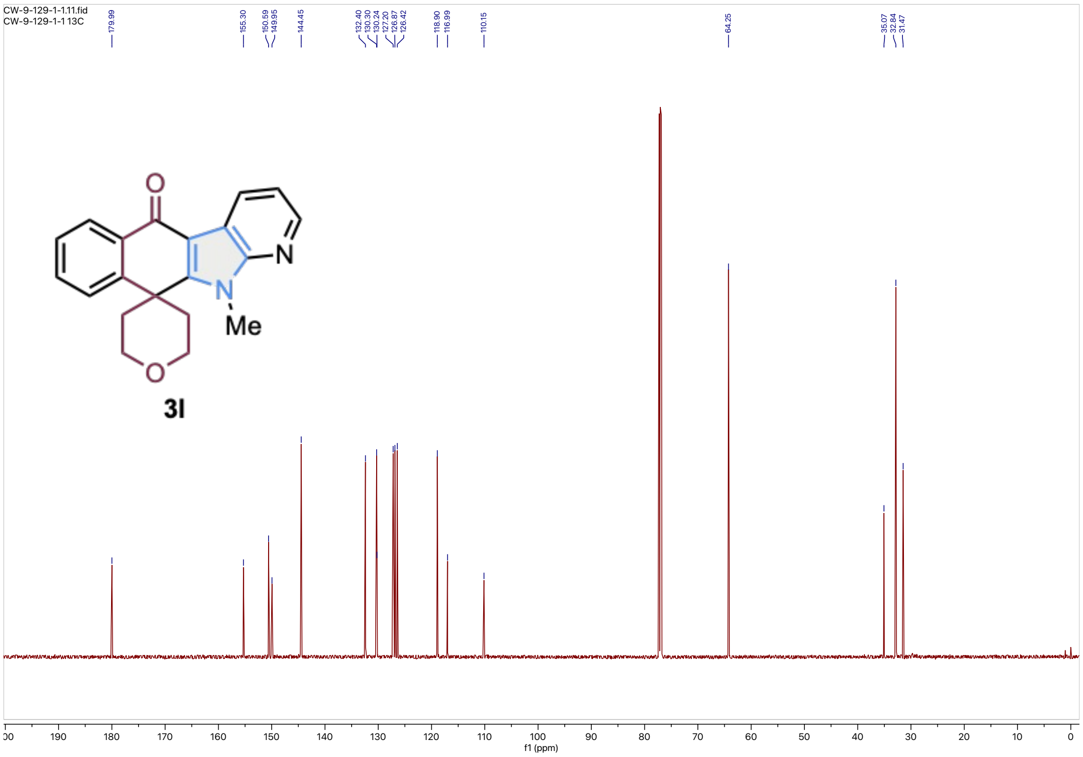

^{1}H NMR for **3m** (500 MHz, $CDCl_3$)

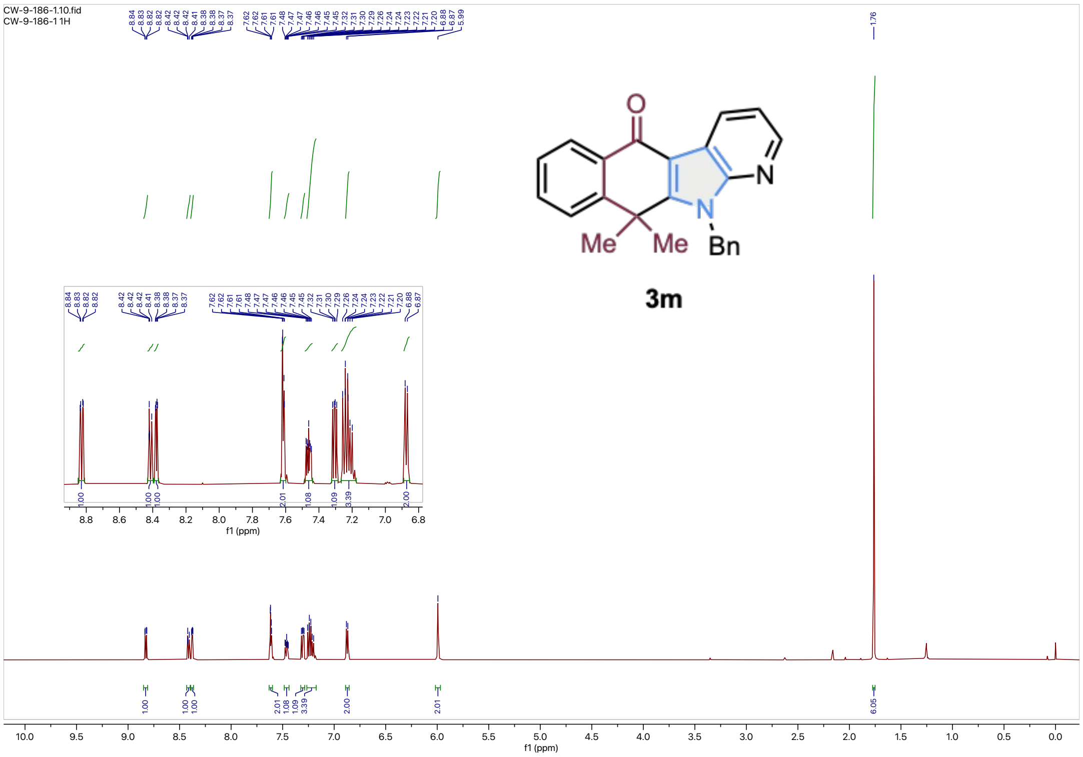

^{13}C NMR for **3m** (126 MHz, $CDCl_3$)

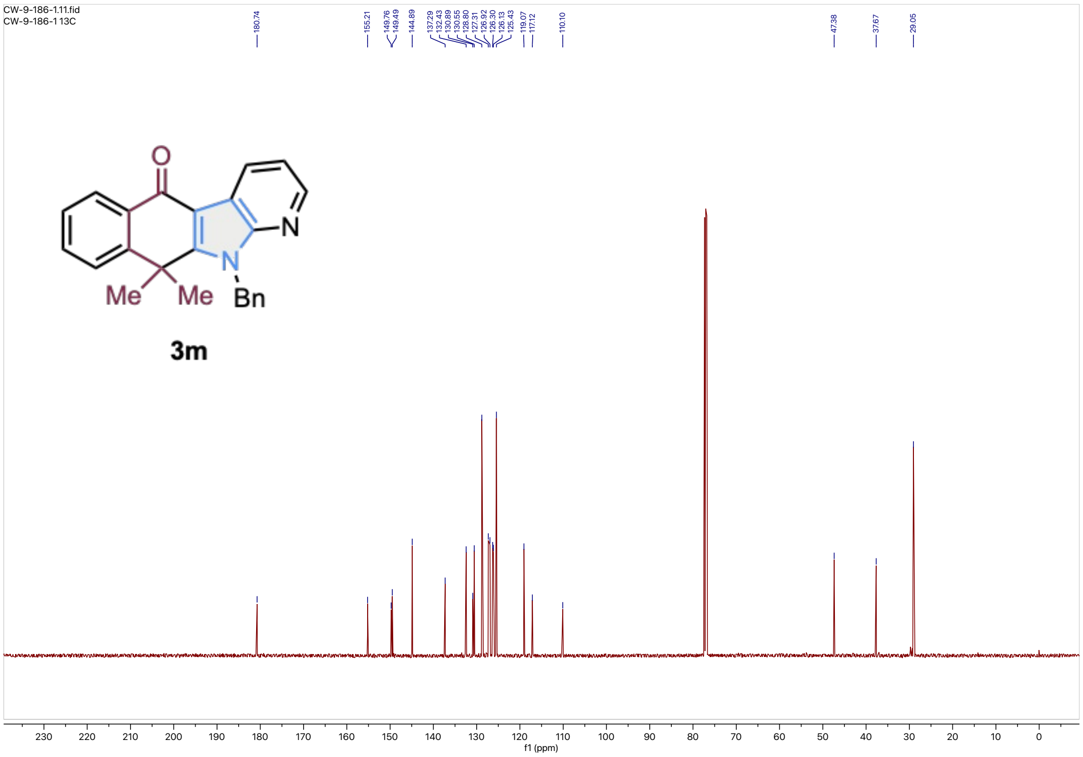

^{1}H NMR for **3n** (500 MHz, $CDCl_3$)

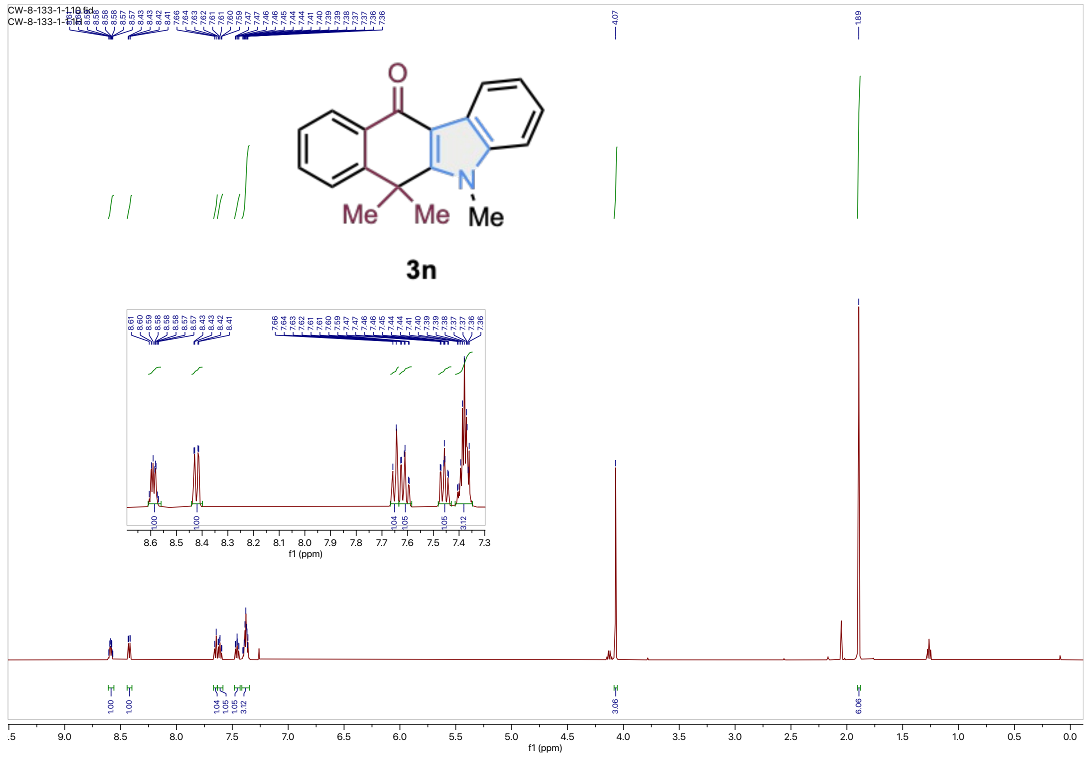

^{13}C NMR for **3n** (126 MHz, $CDCl_3$)

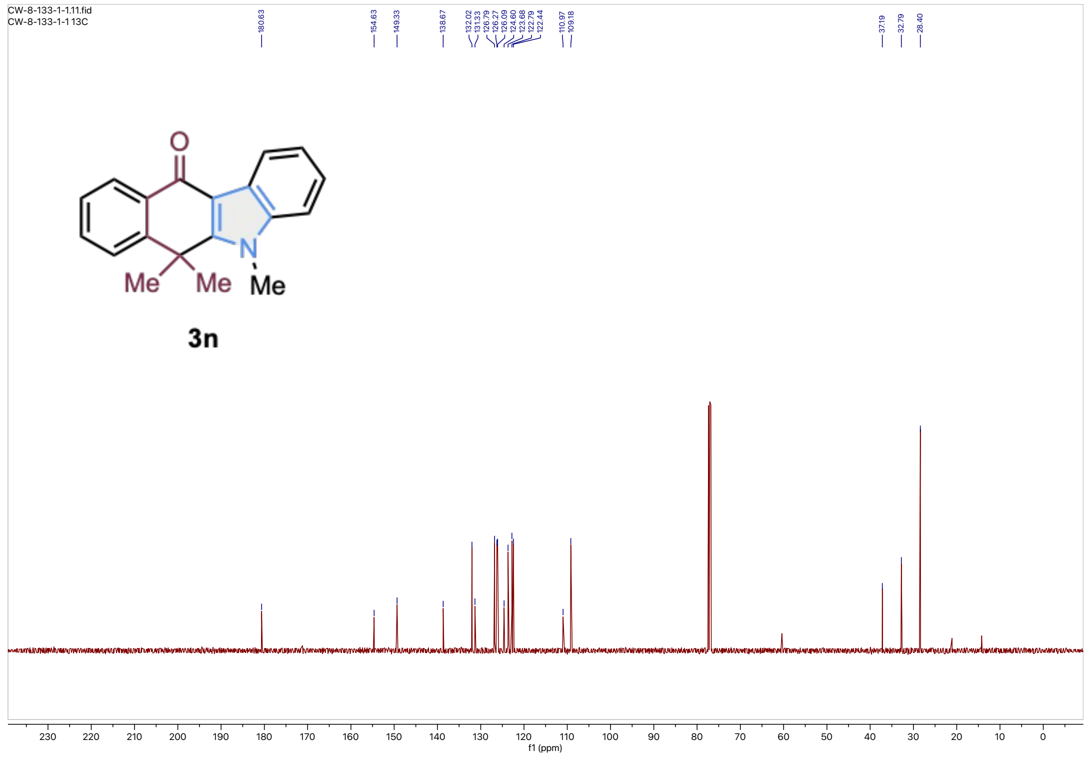

^{1}H NMR for **3o** (600 MHz, $CDCl_3$)

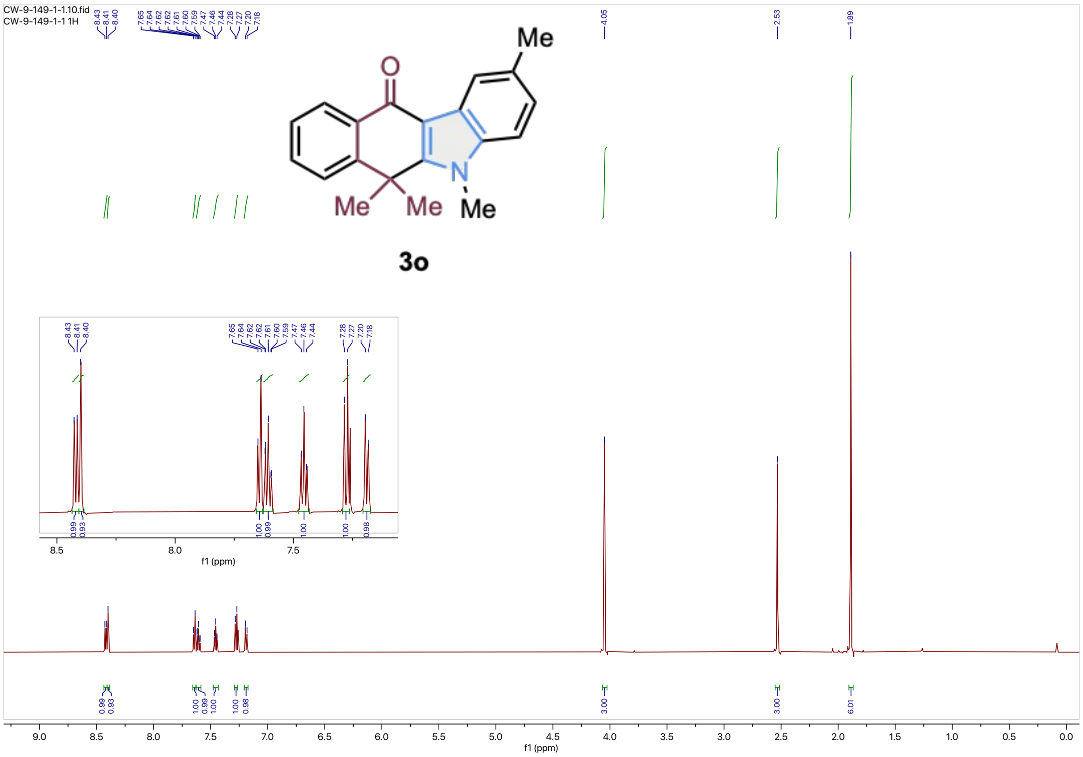

^{13}C NMR for **3o** (151 MHz, $CDCl_3$)

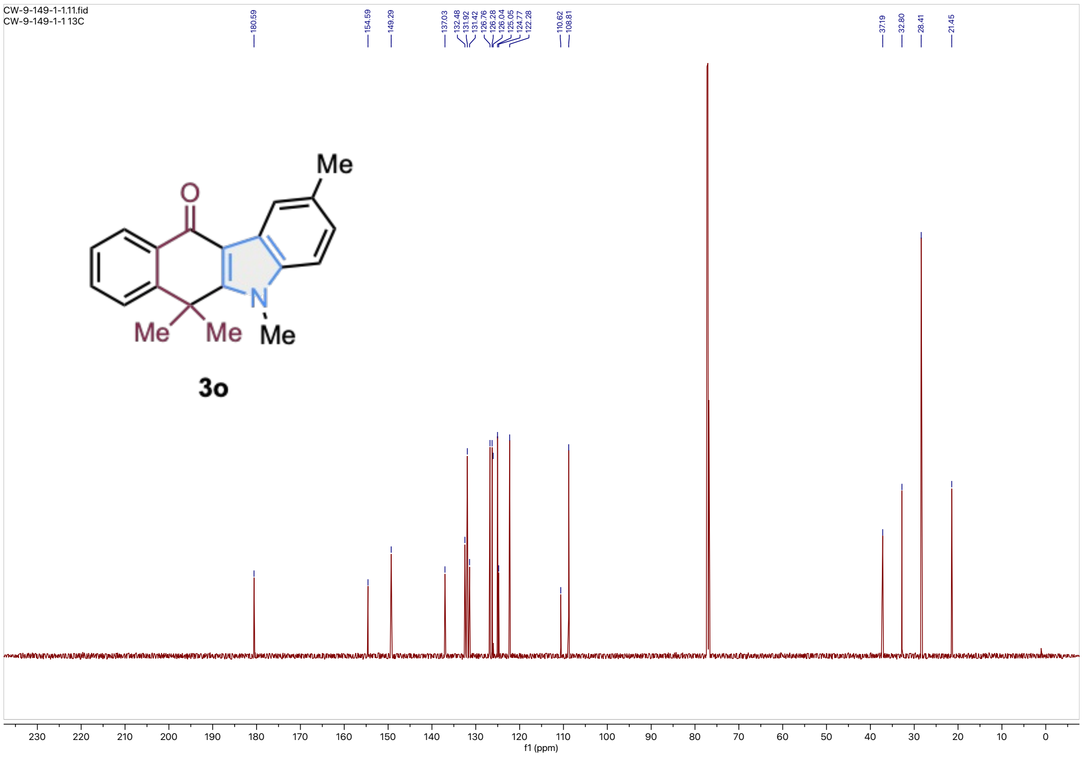

^{1}H NMR for **3p** (600 MHz, $CDCl_3$)

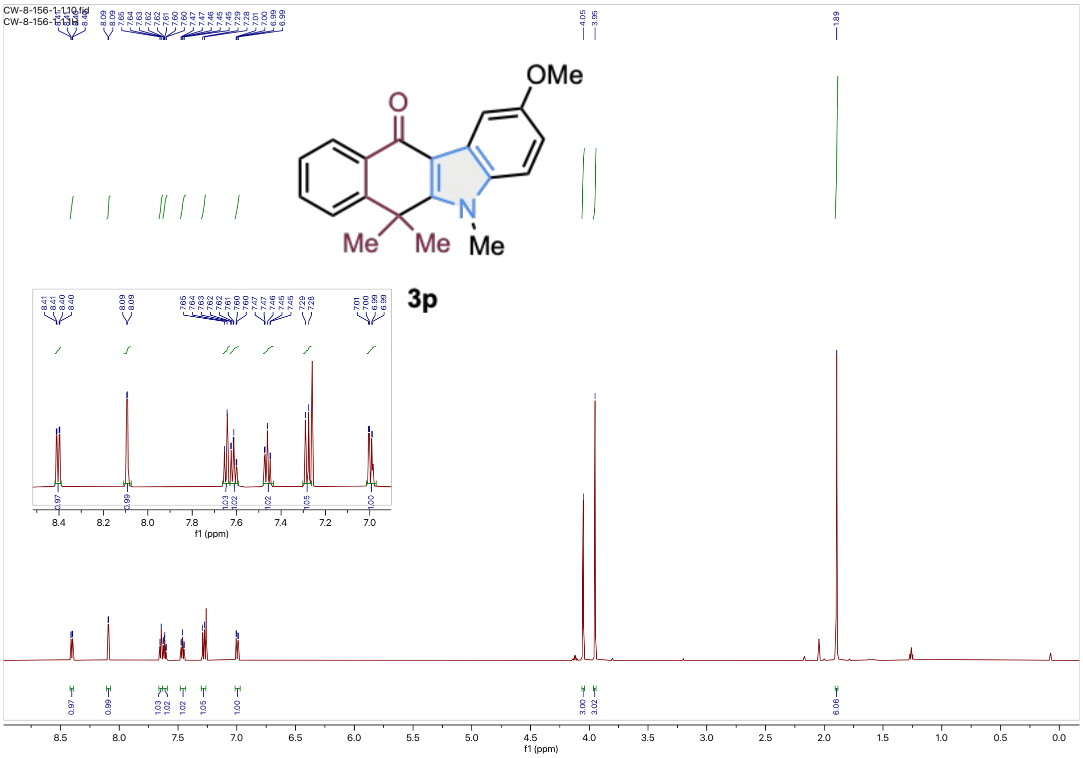

^{13}C NMR for **3p** (151 MHz, $CDCl_3$)

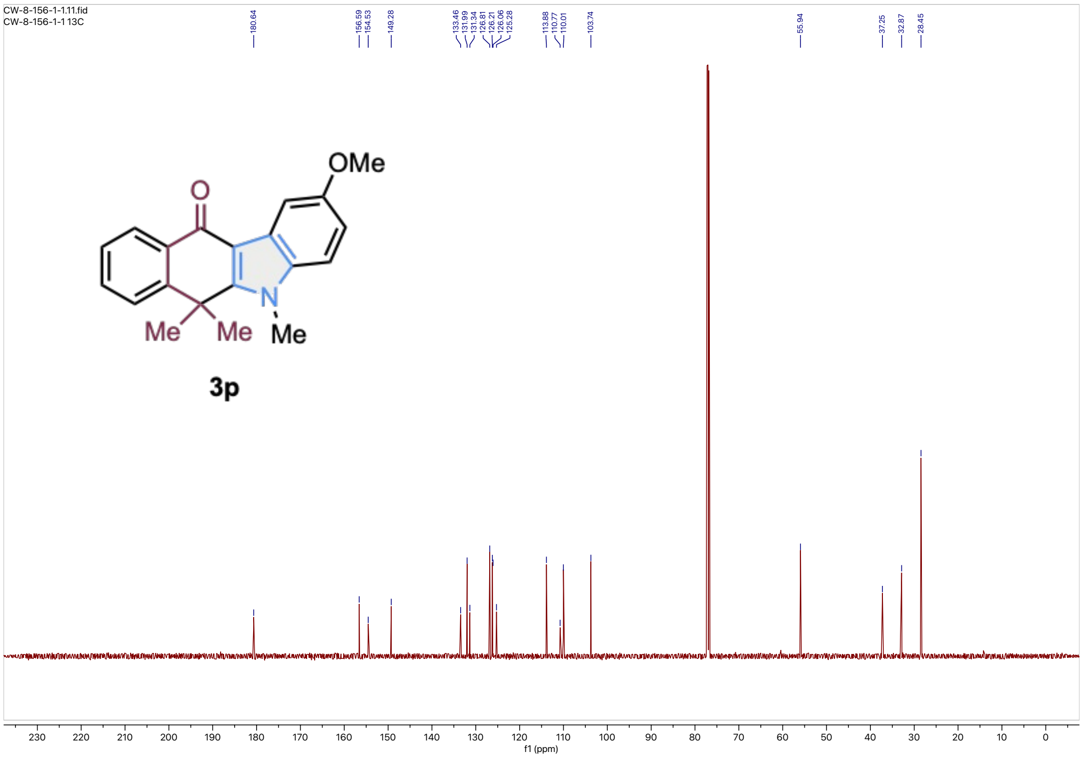

^{1}H NMR for **3q** (500 MHz, $CDCl_3$)

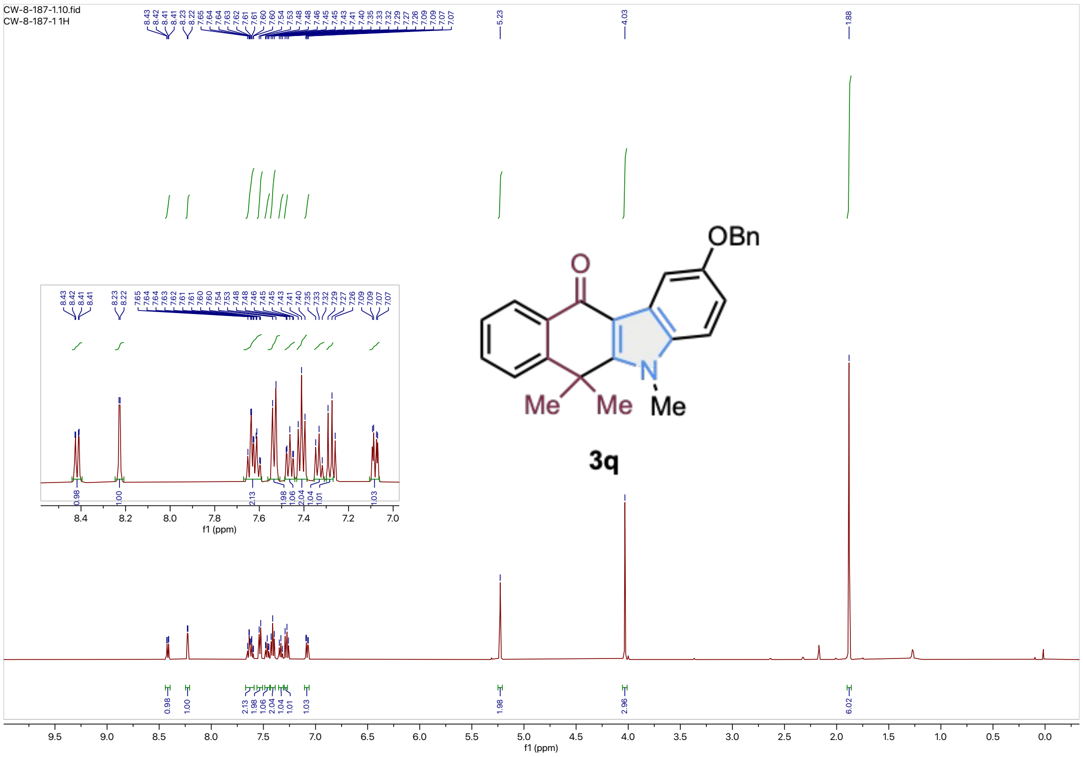

^{13}C NMR for **3q** (126 MHz, $CDCl_3$)

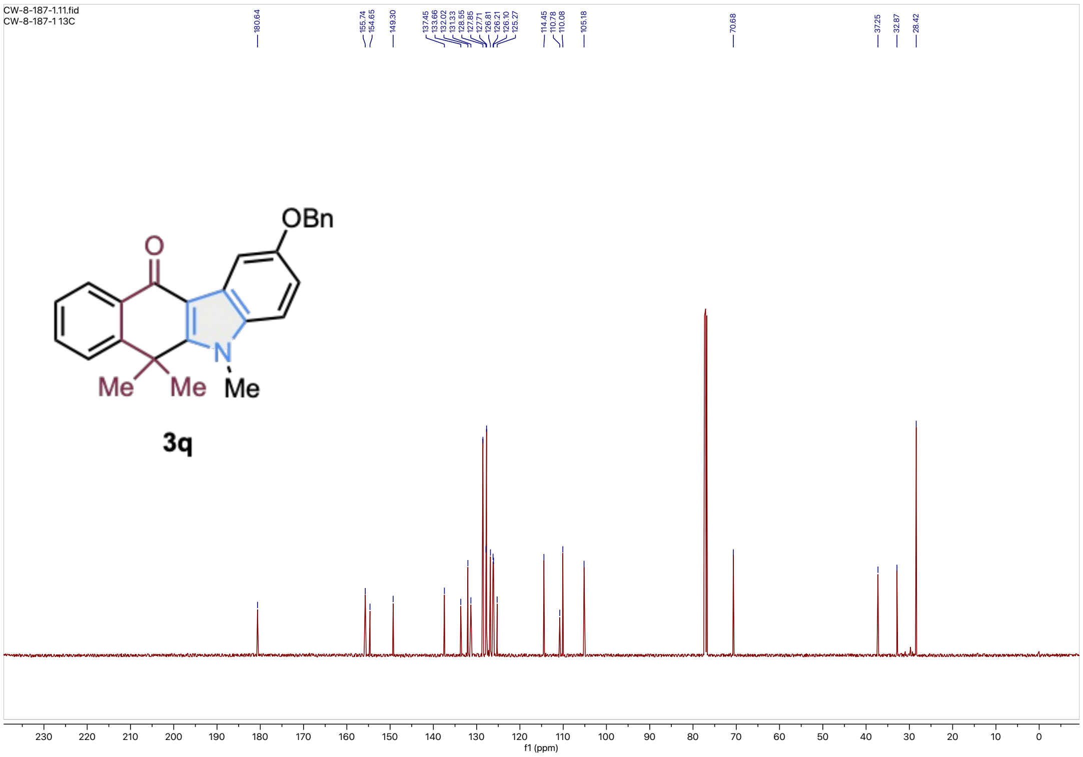

^{1}H NMR for **3r** (500 MHz, $CDCl_3$)

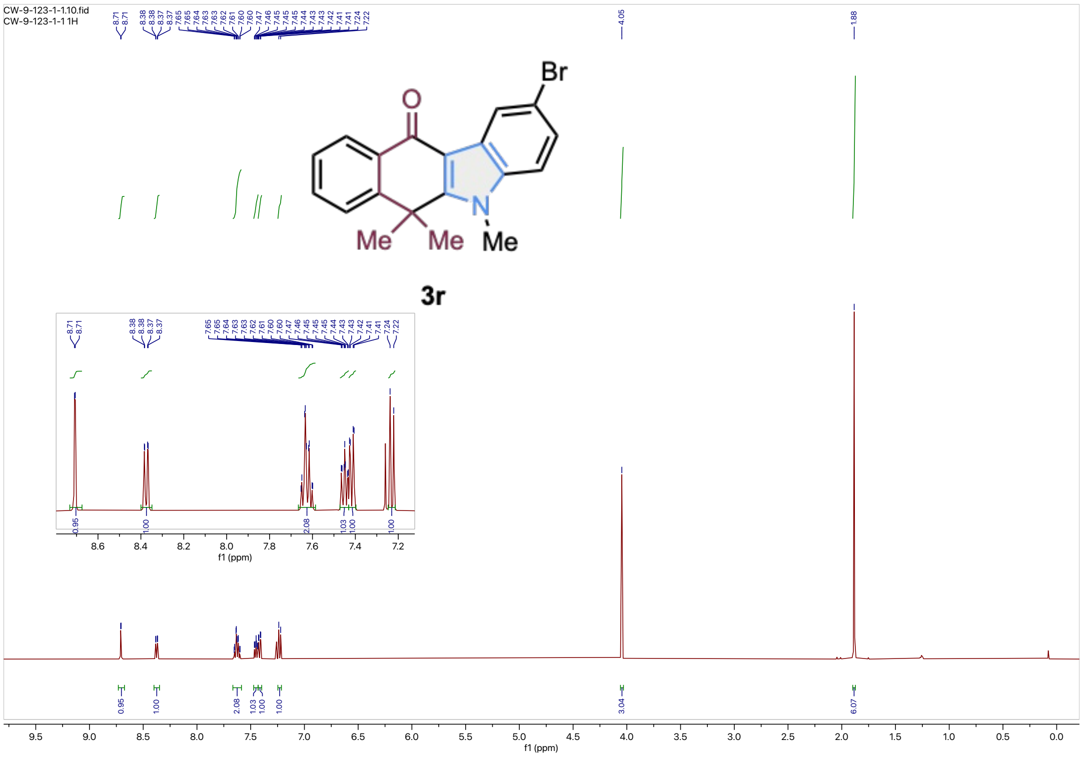

^{13}C NMR for **3r** (126 MHz, $CDCl_3$)

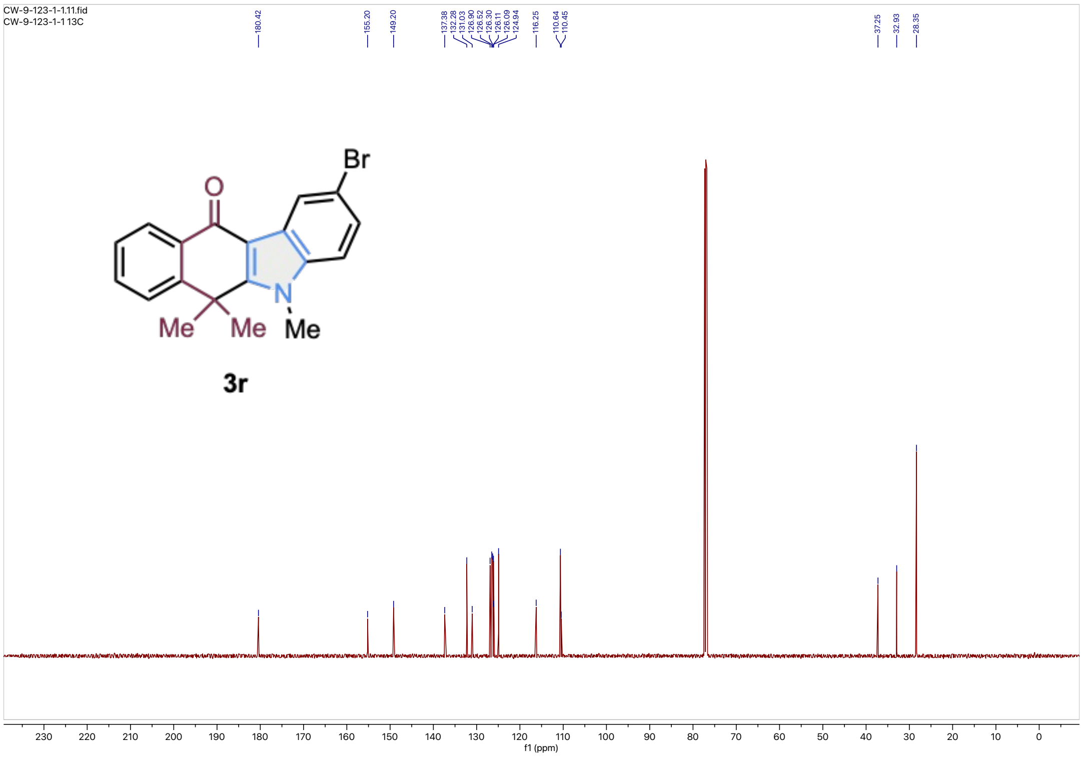

^{1}H NMR for **3s** (600 MHz, $CDCl_3$)

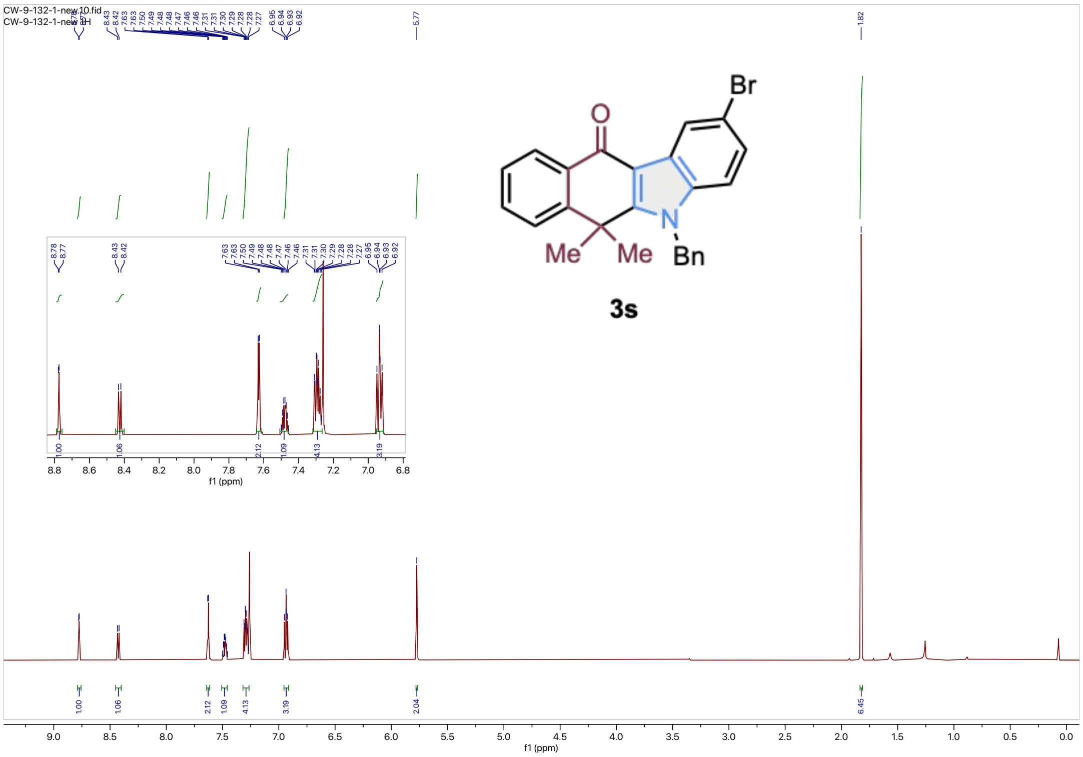

^{13}C NMR for **2s** (151 MHz, $CDCl_3$)

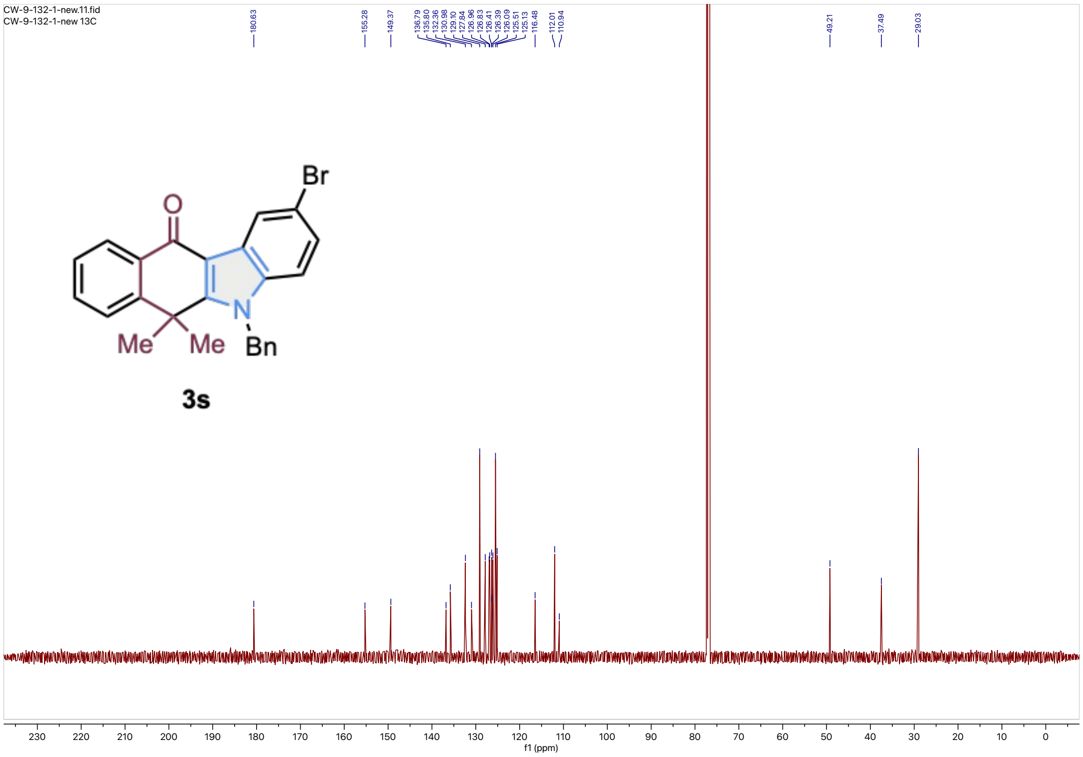

^{1}H NMR for **3t** (500 MHz, $CDCl_3$)

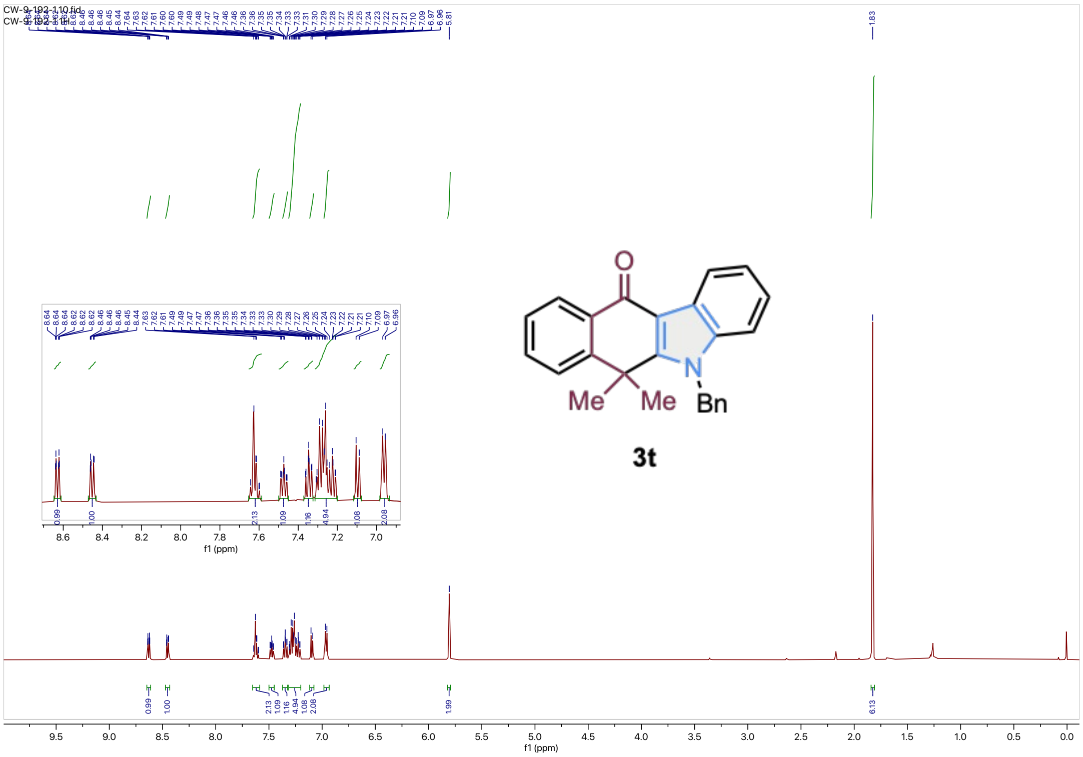

^{13}C NMR for **3t** (126 MHz, $CDCl_3$)

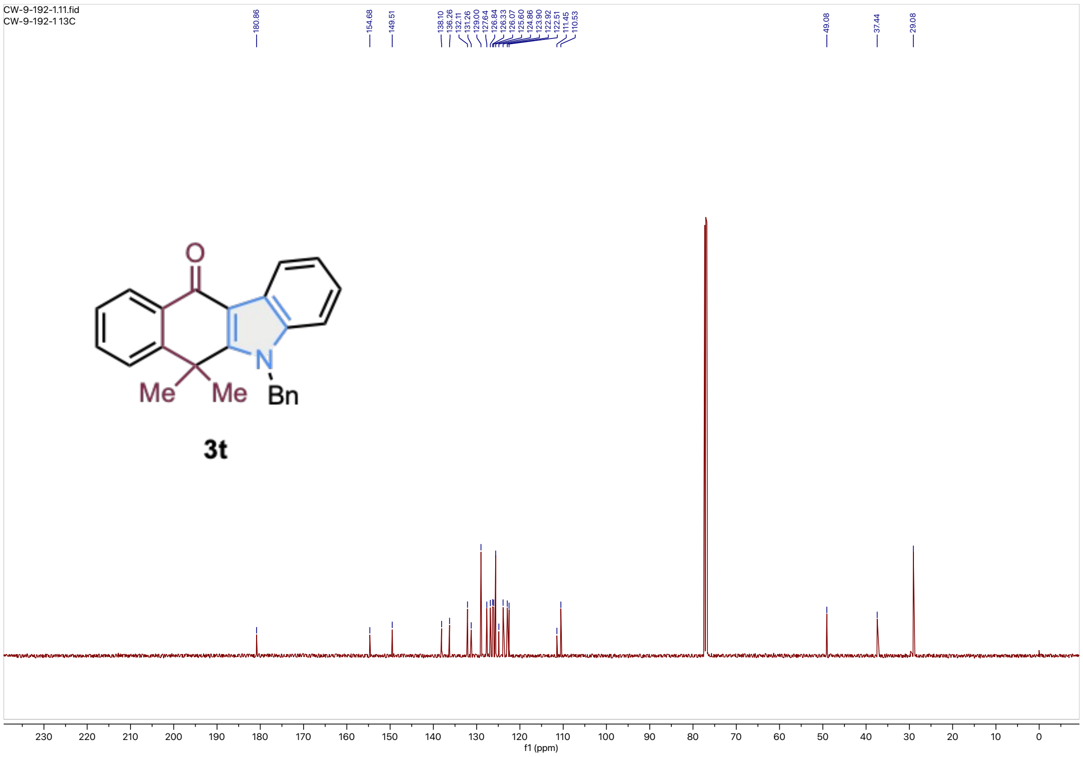

^{1}H NMR for **3u** (500 MHz, $CDCl_3$)

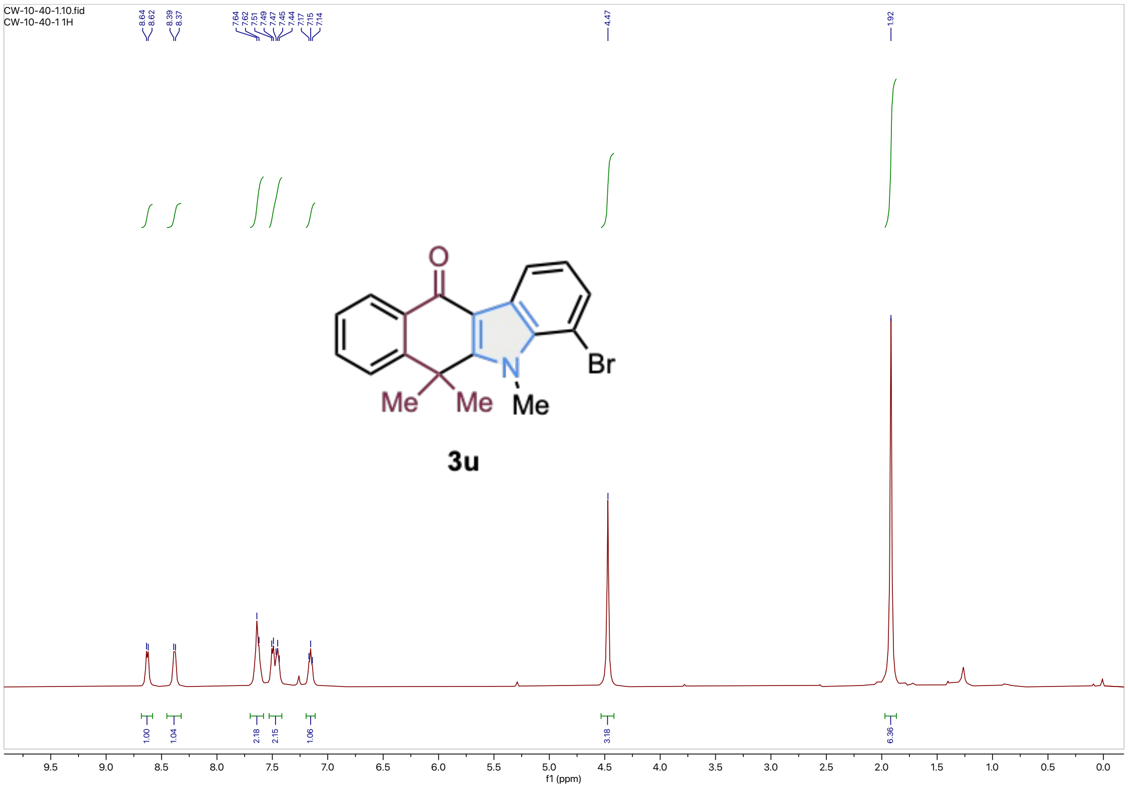

^{13}C NMR for **3u** (126 MHz, $CDCl_3$)

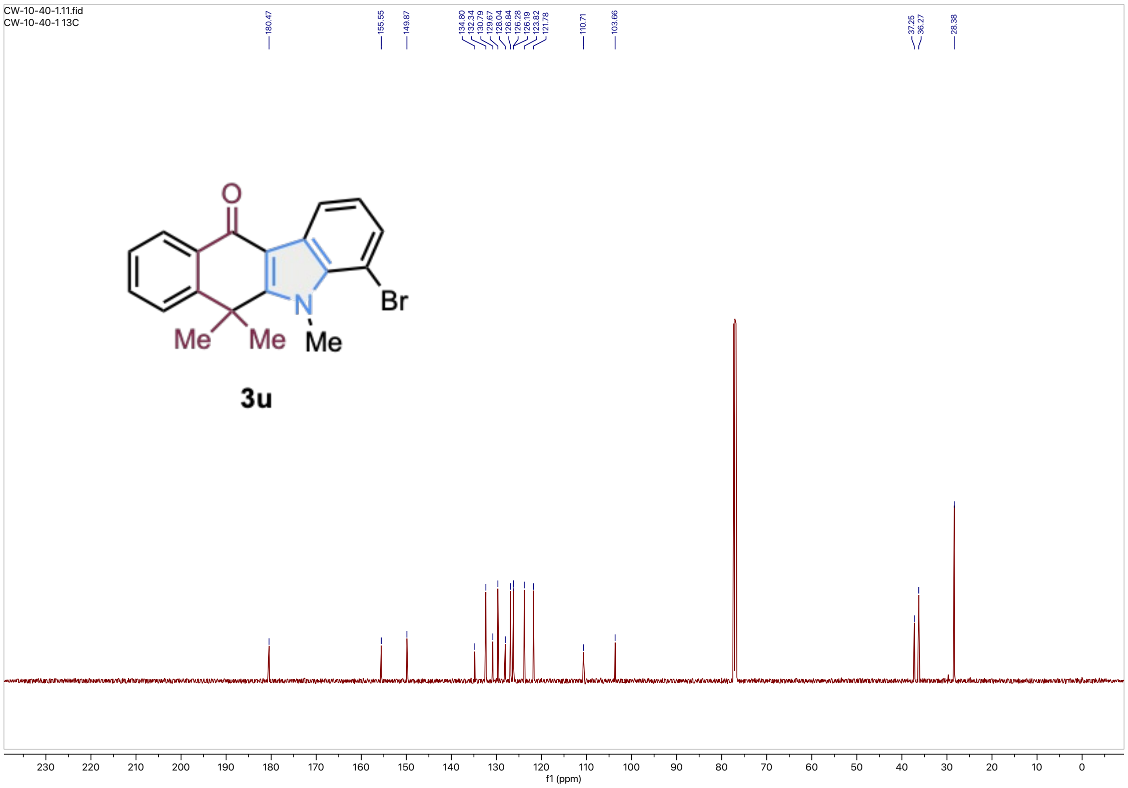

^{1}H NMR for **3v** (500 MHz, $CDCl_3$)

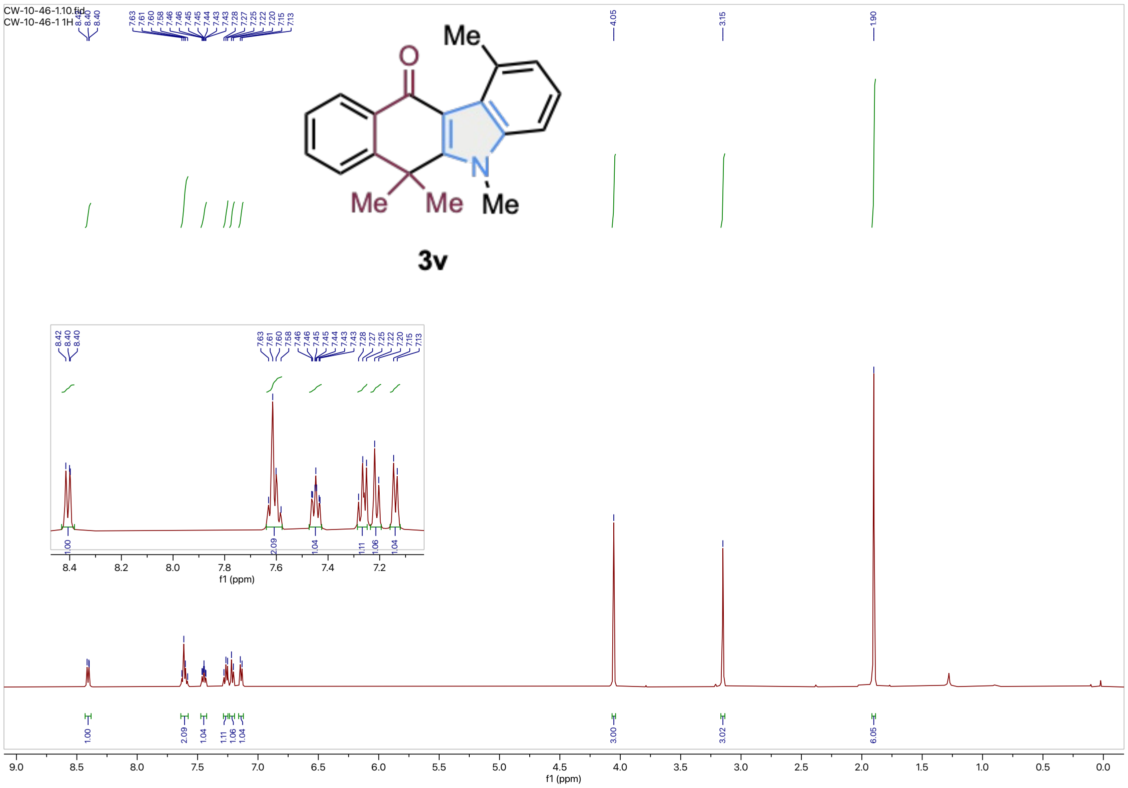

^{13}C NMR for **3v** (126 MHz, $CDCl_3$)

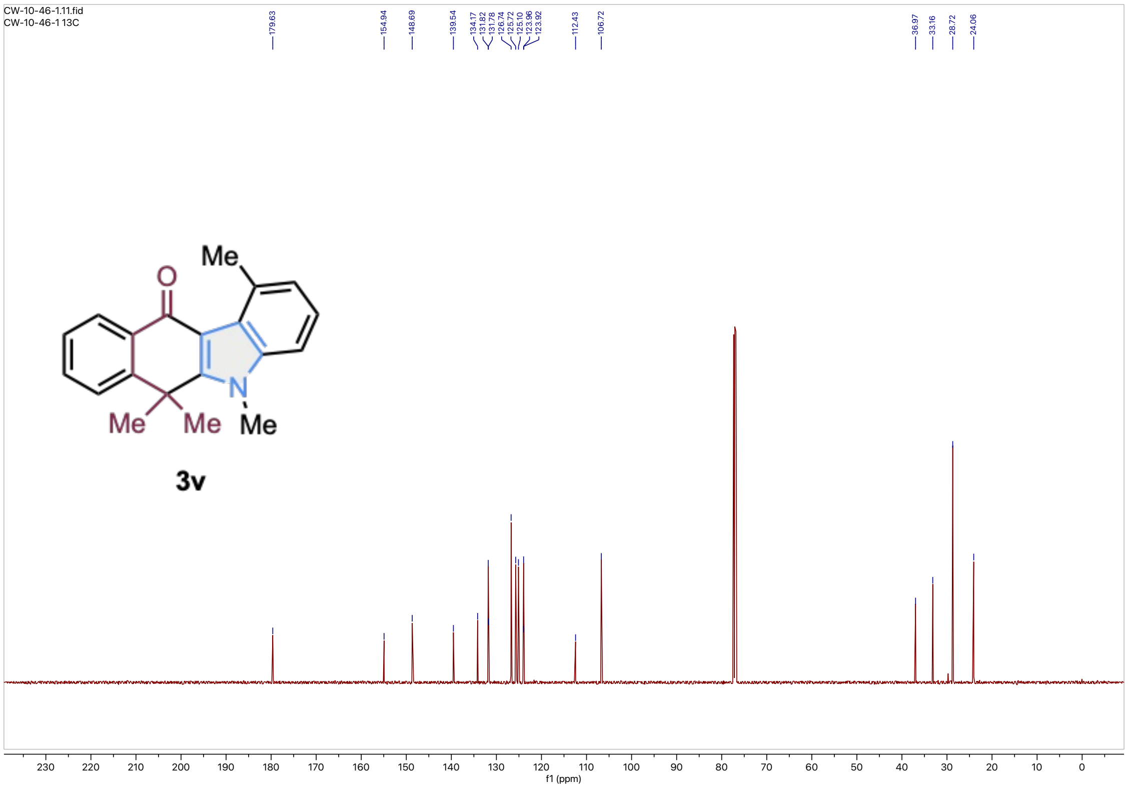

^{1}H NMR for **3w** (500 MHz, $CDCl_3$)

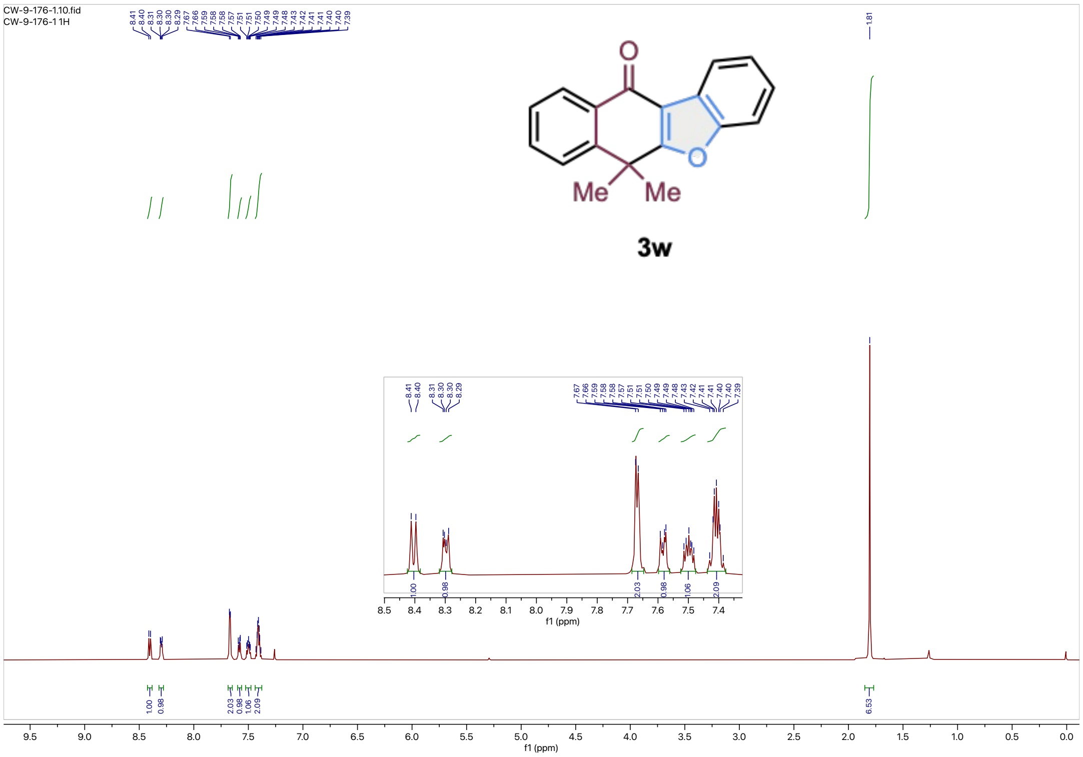

^{13}C NMR for **3w** (126 MHz, $CDCl_3$)

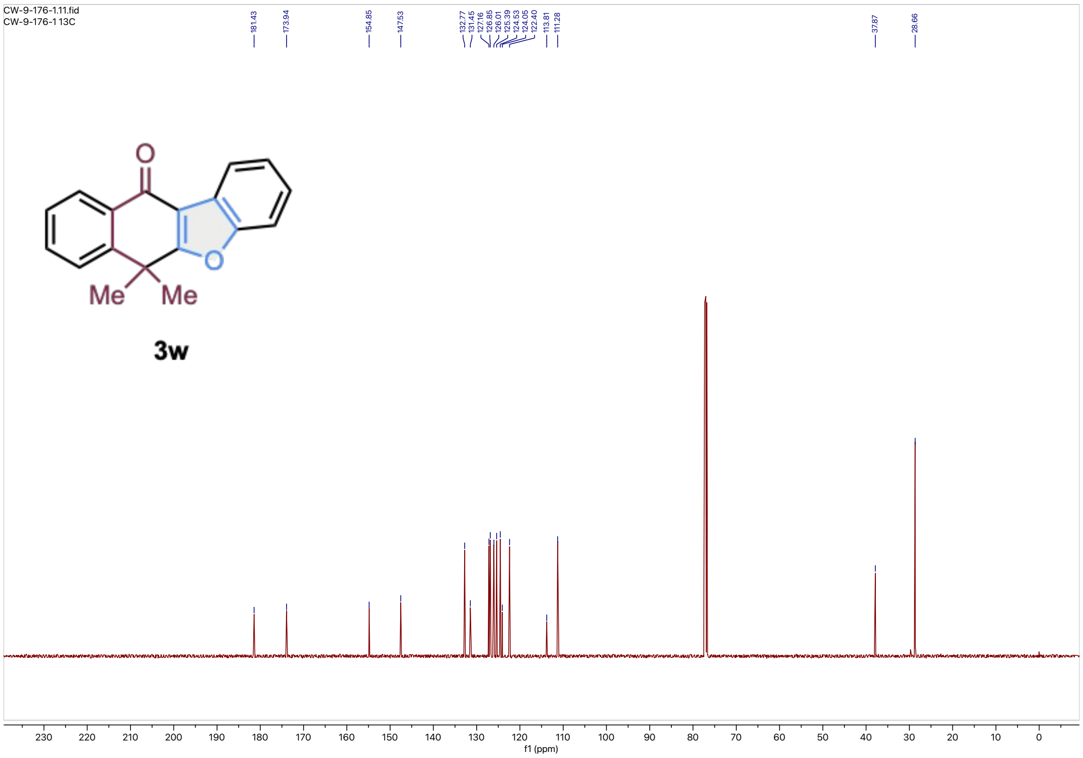

COSY NMR for **3w**

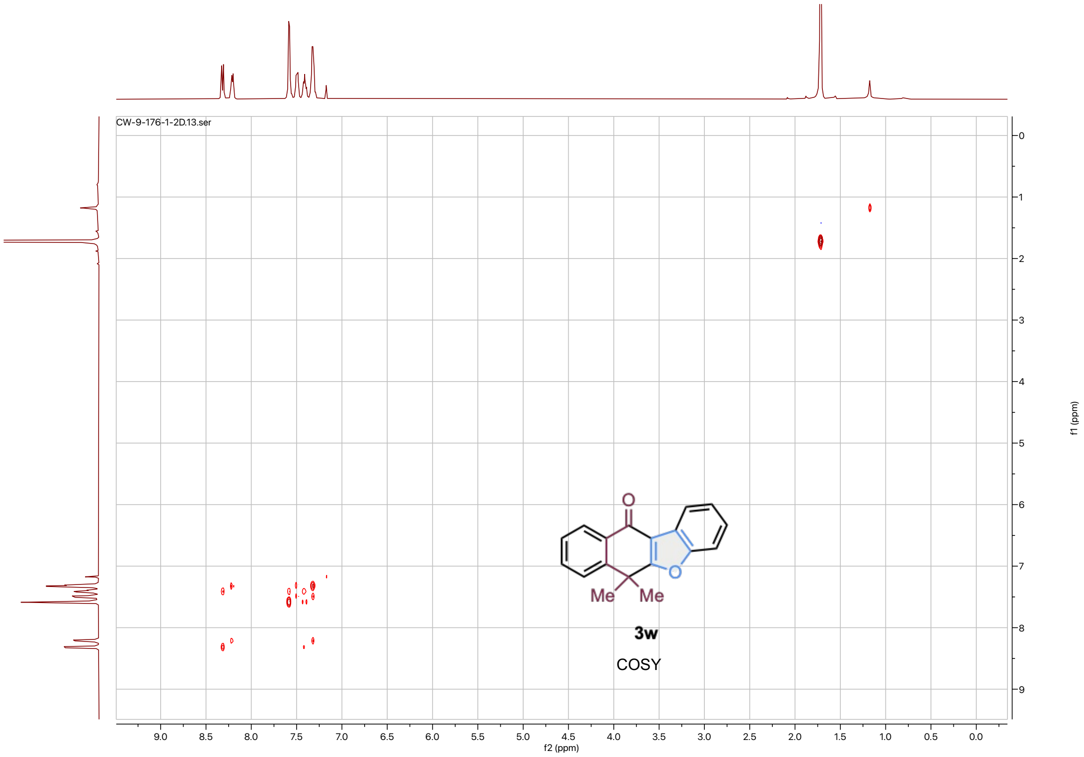

HSQC NMR for **3w**

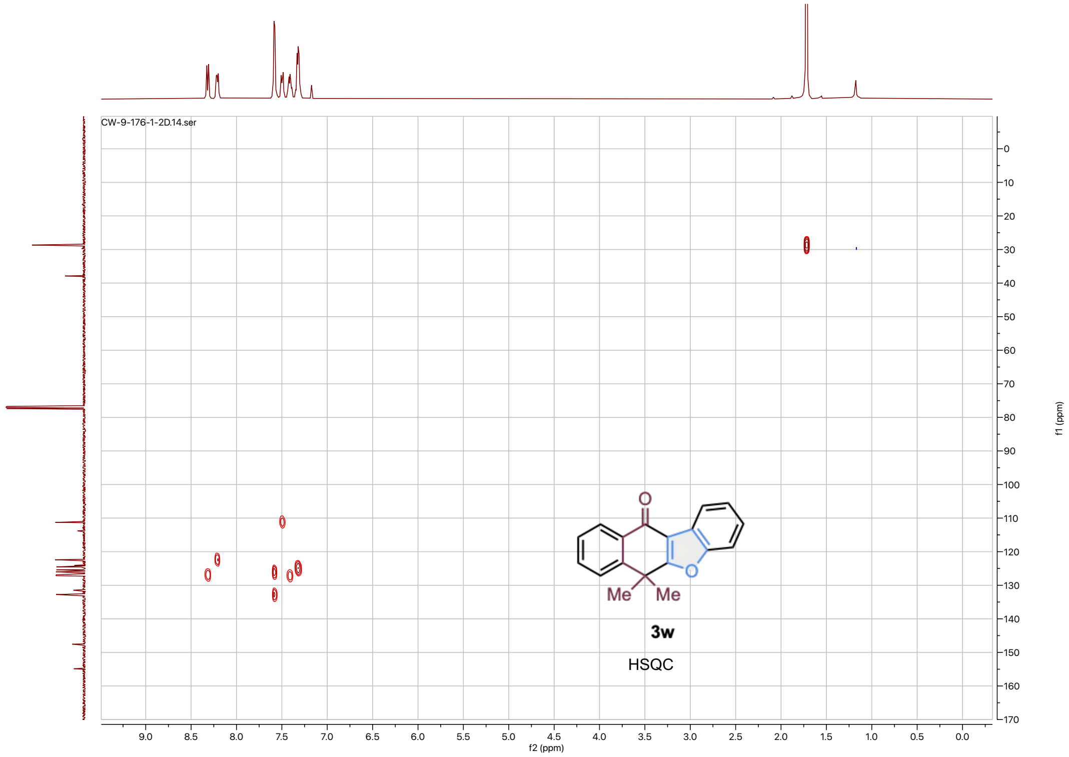

HMBC NMR for **3w**

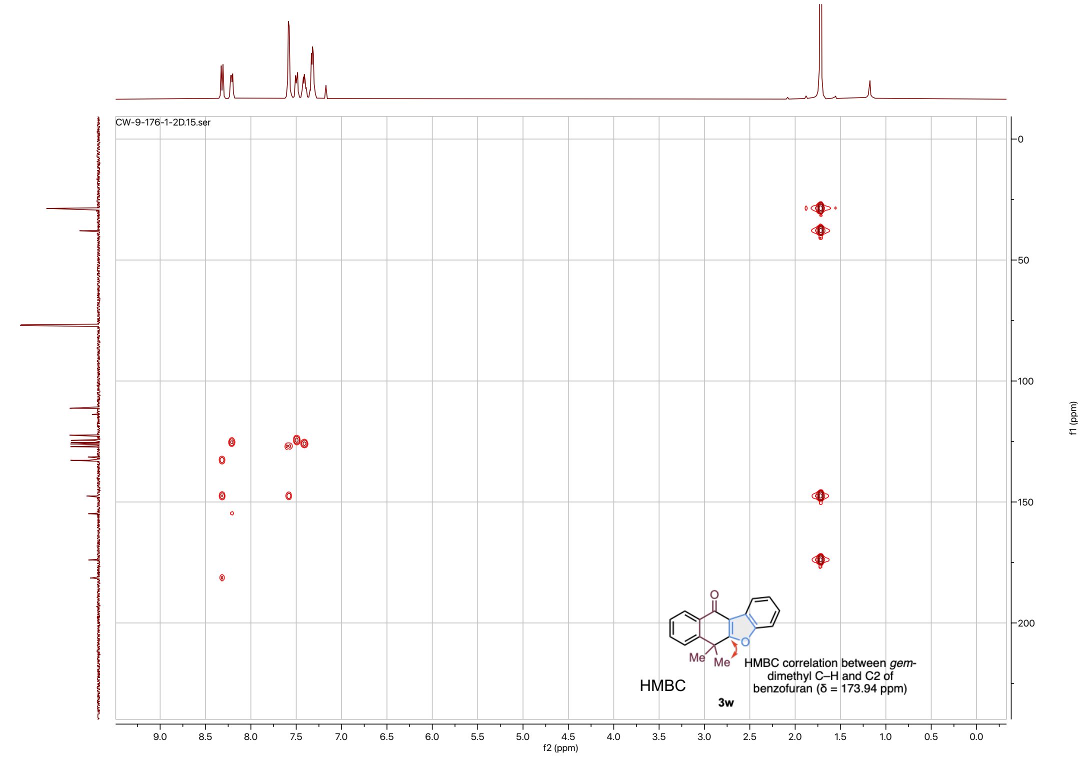

2D NOE NMR for **3w**

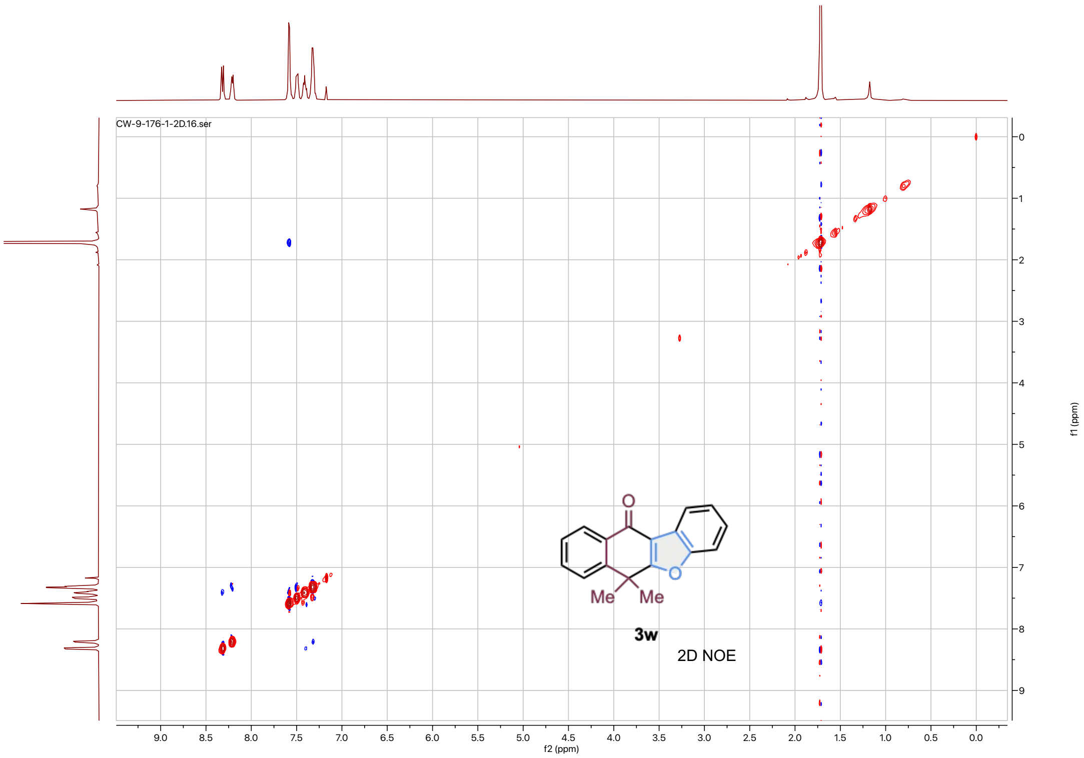

^{1}H NMR for **3x** (500 MHz, $CDCl_3$)

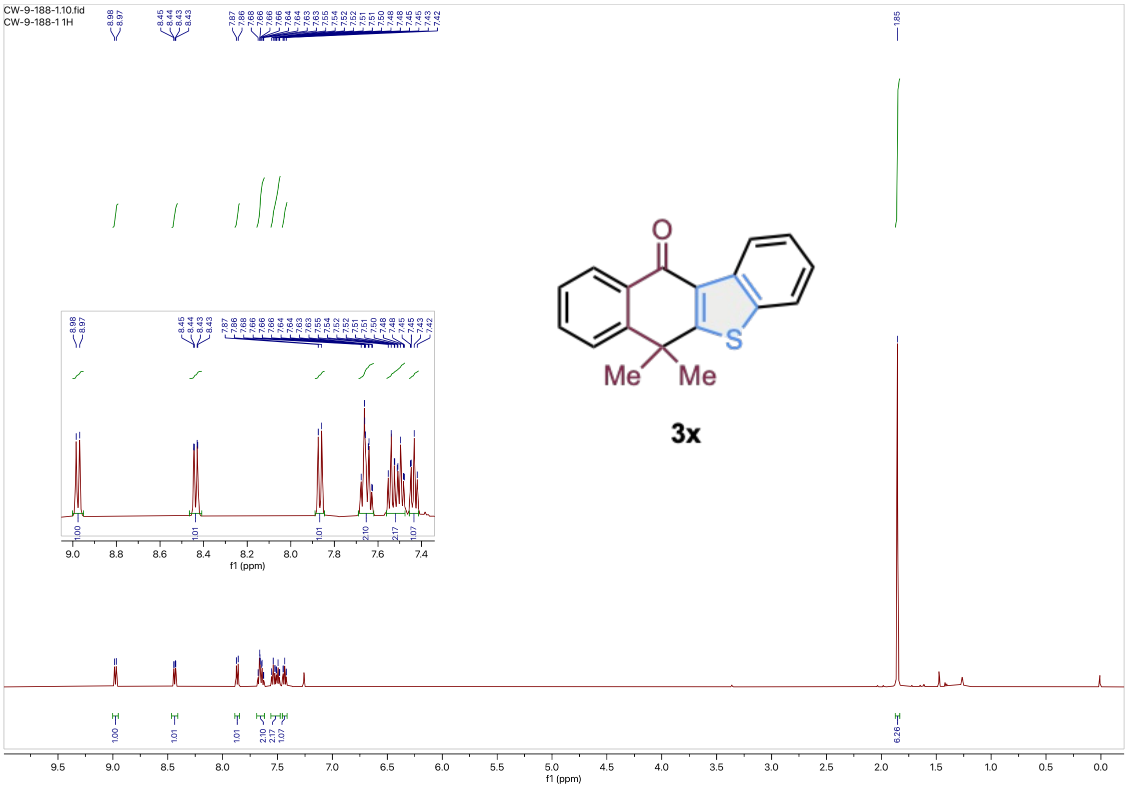

^{13}C NMR for **3x** (126 MHz, $CDCl_3$)

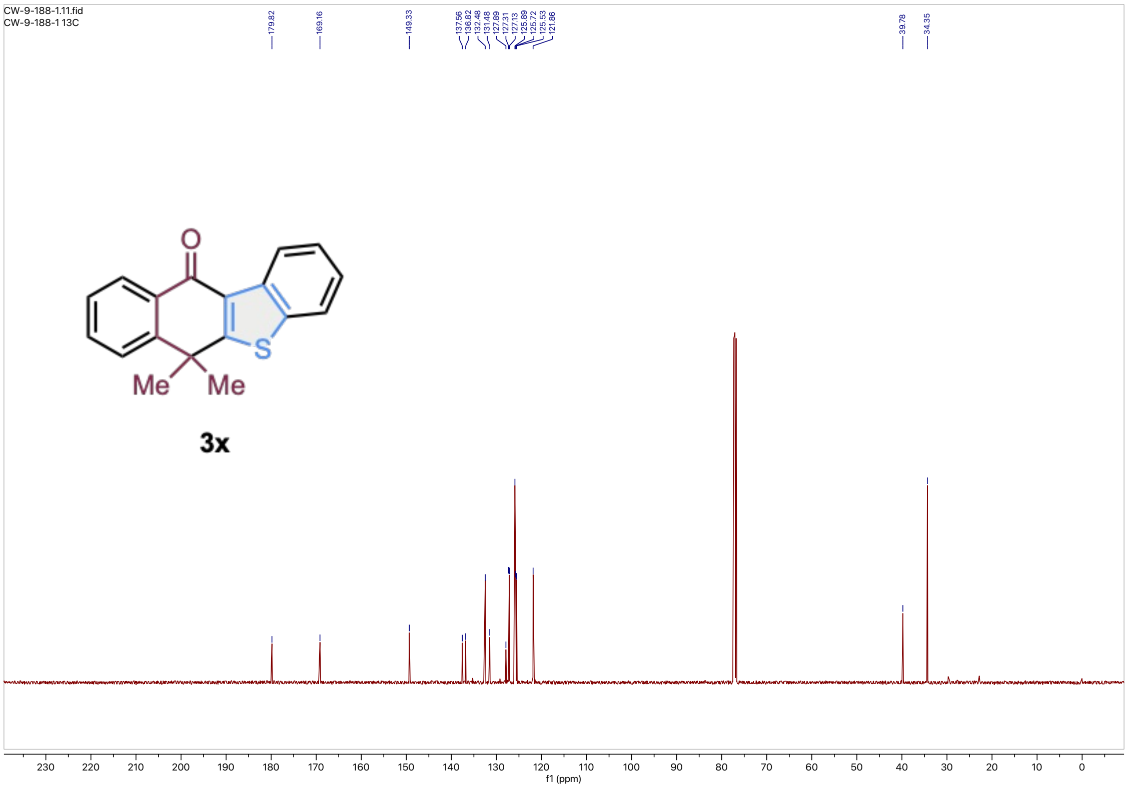

^{1}H NMR for **3y** (600 MHz, $CDCl_3$)

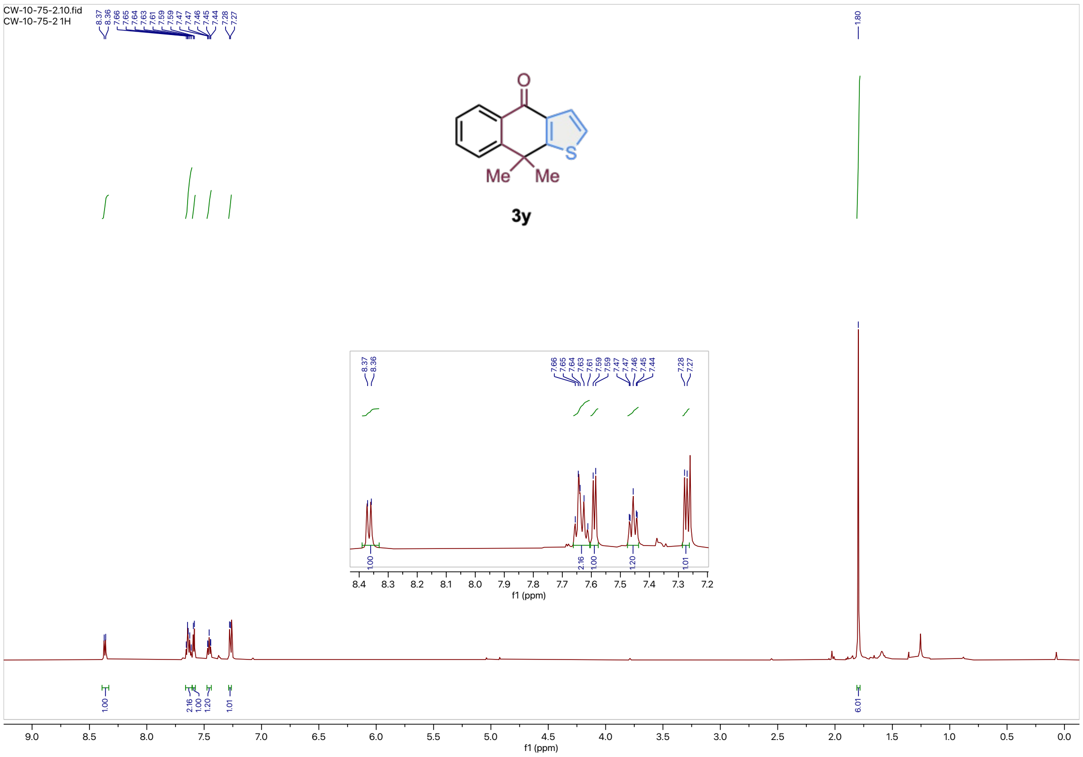

^{13}C NMR for **3y** (151 MHz, $CDCl_3$)

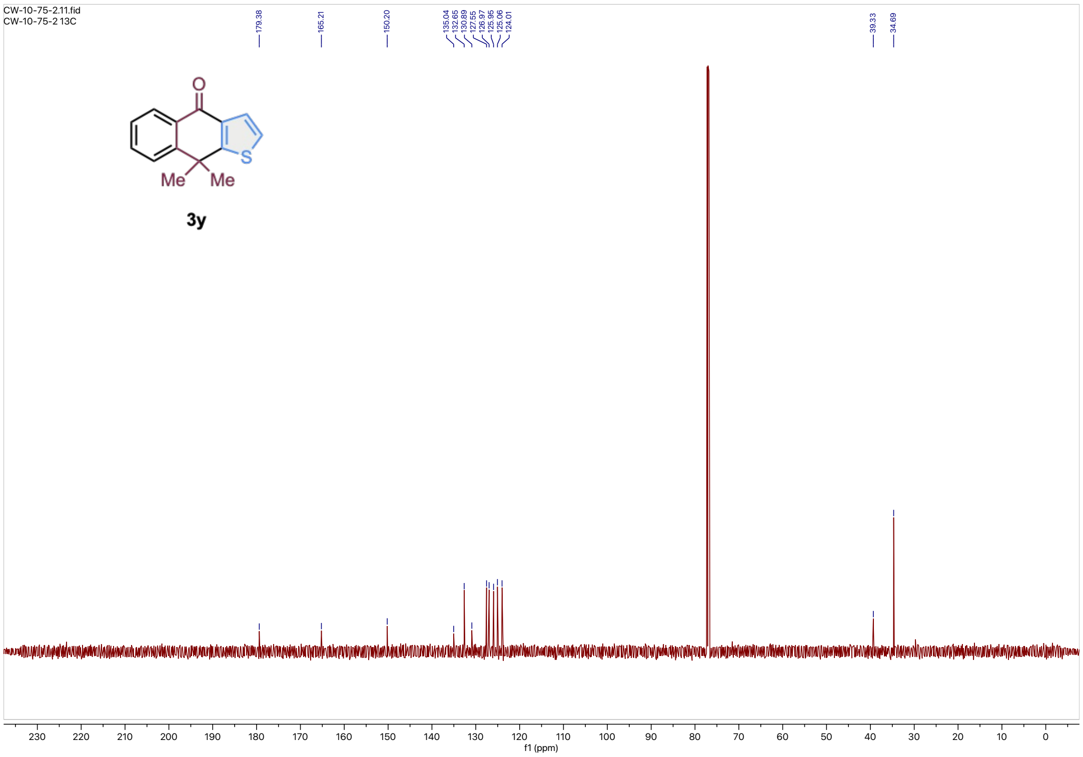

^{1}H NMR for **3n’** (500 MHz, $CDCl_3$)

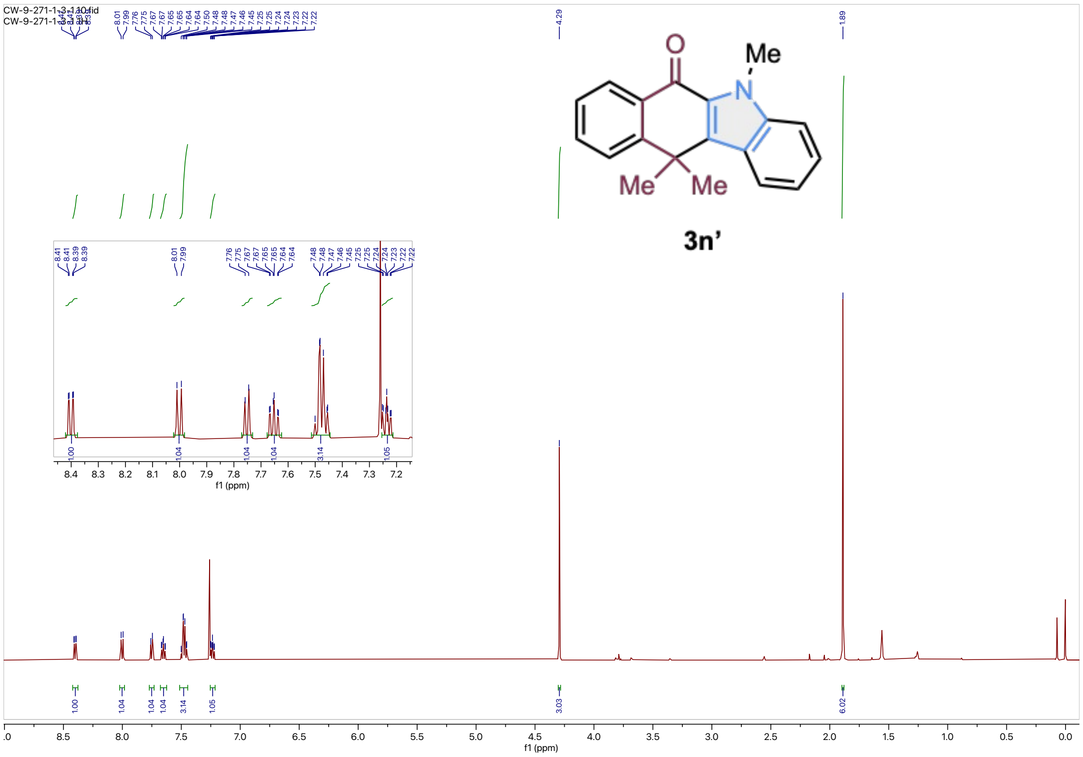

^{13}C NMR for **3n’** (126 MHz, $CDCl_3$)

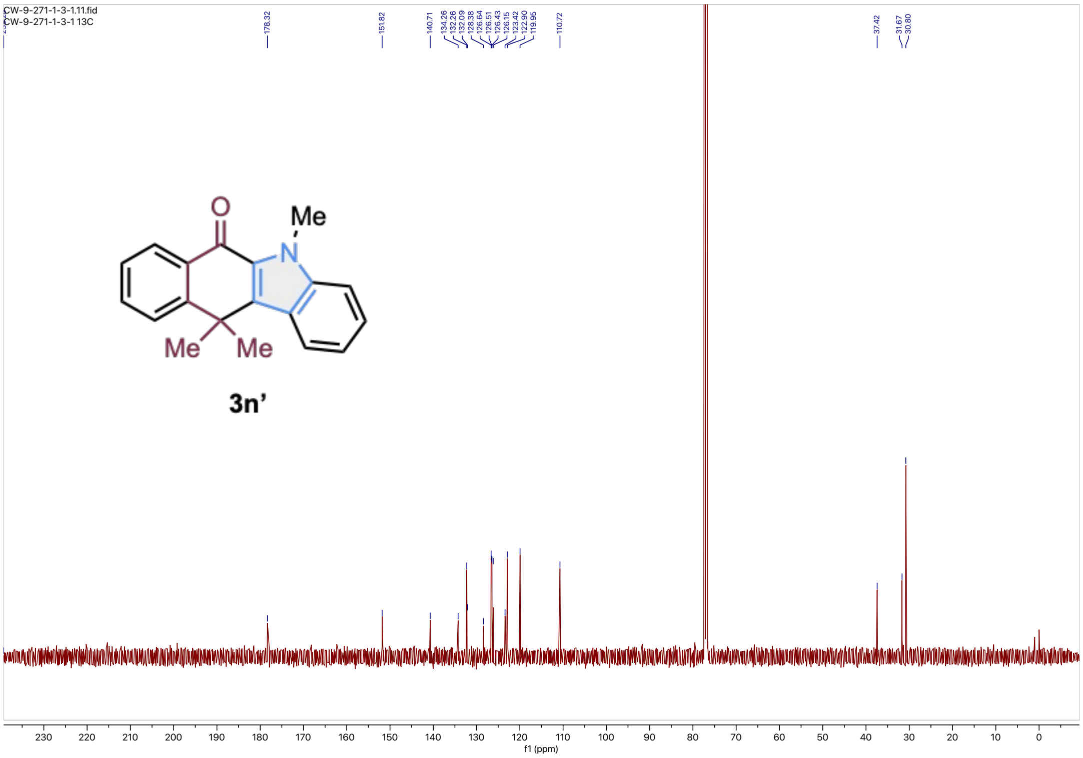

COSY NMR for **3n'**

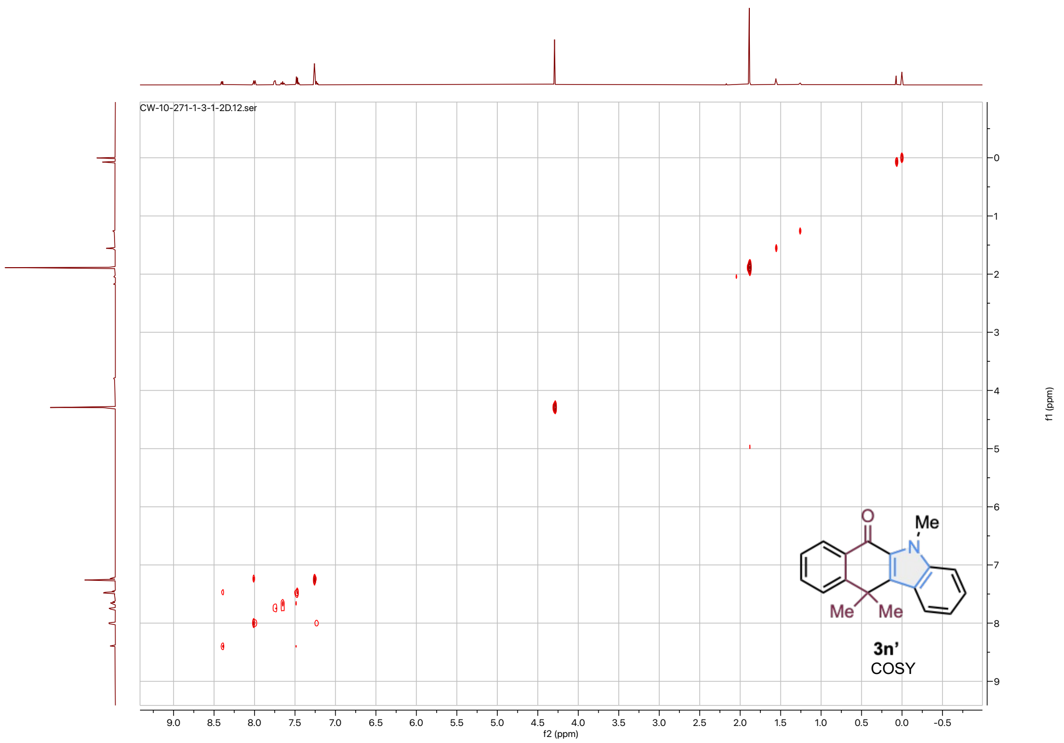

HSQC NMR for **3n'**

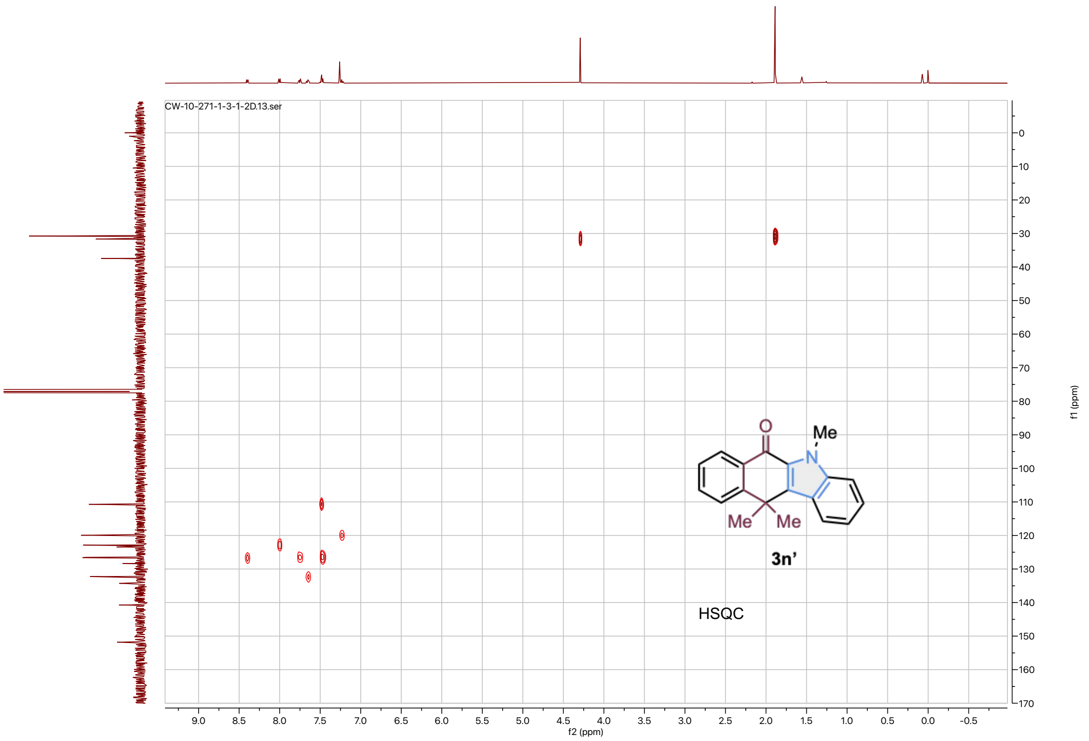

HMBC NMR for **3n’**

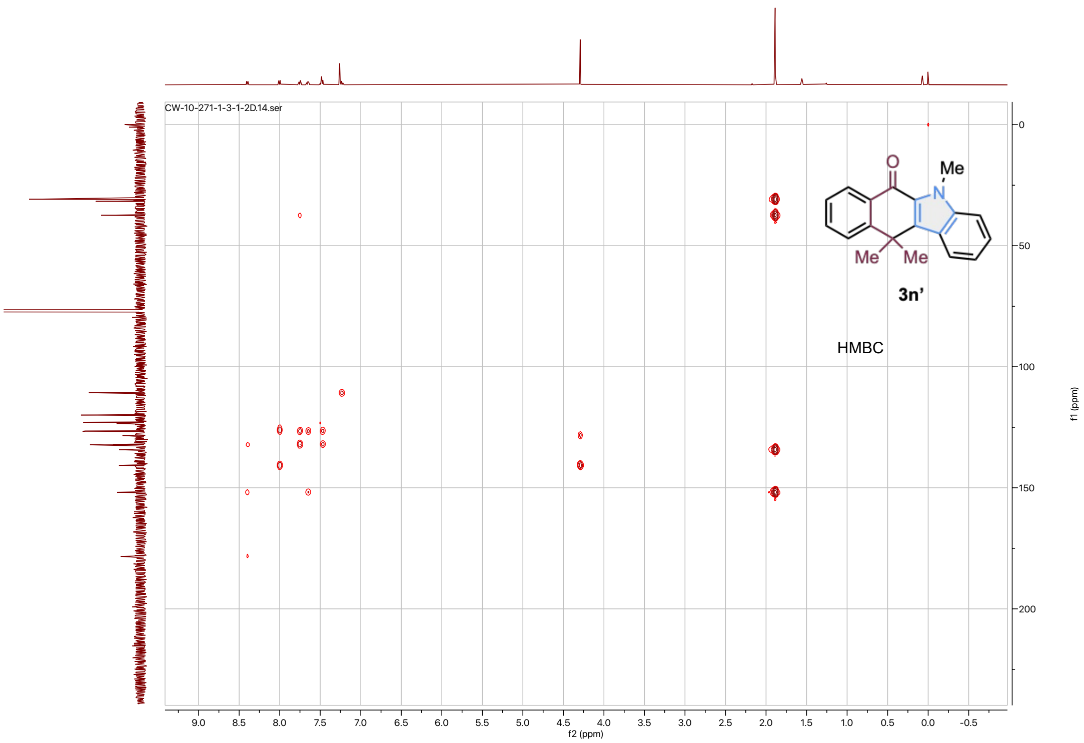

2D NOE NMR for **3n’**

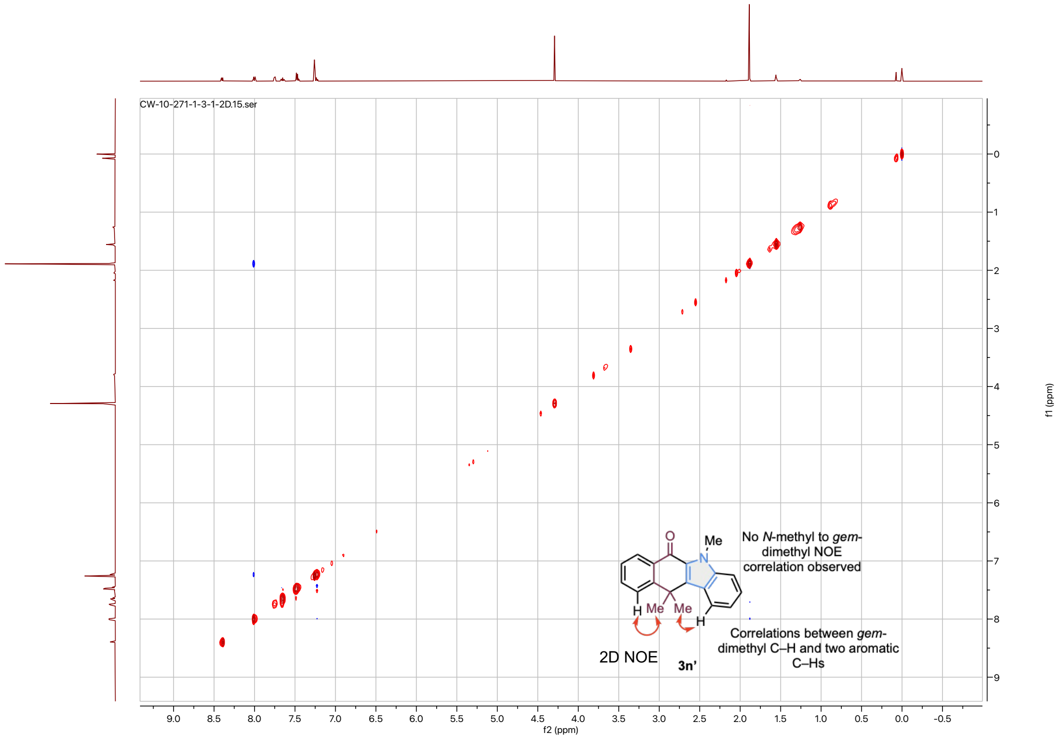

^{1}H NMR for **1a-2** (500 MHz, $CDCl_3$)

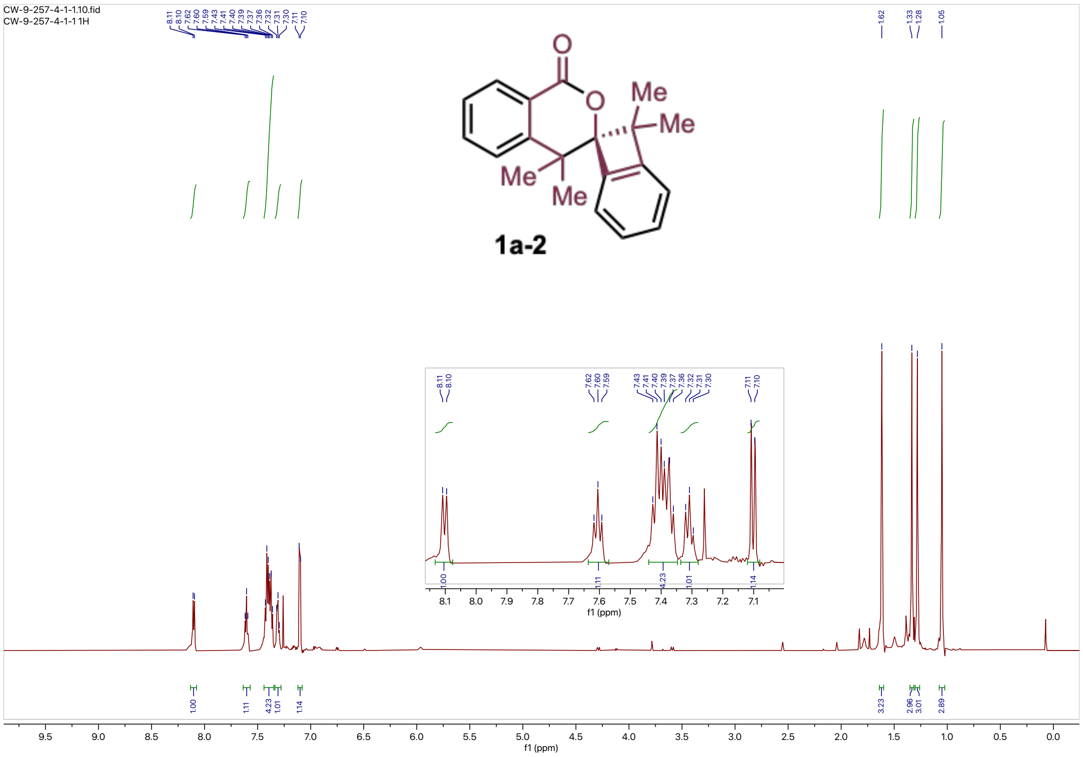

^{13}C NMR for **1a-2** (126 MHz, $CDCl_3$)

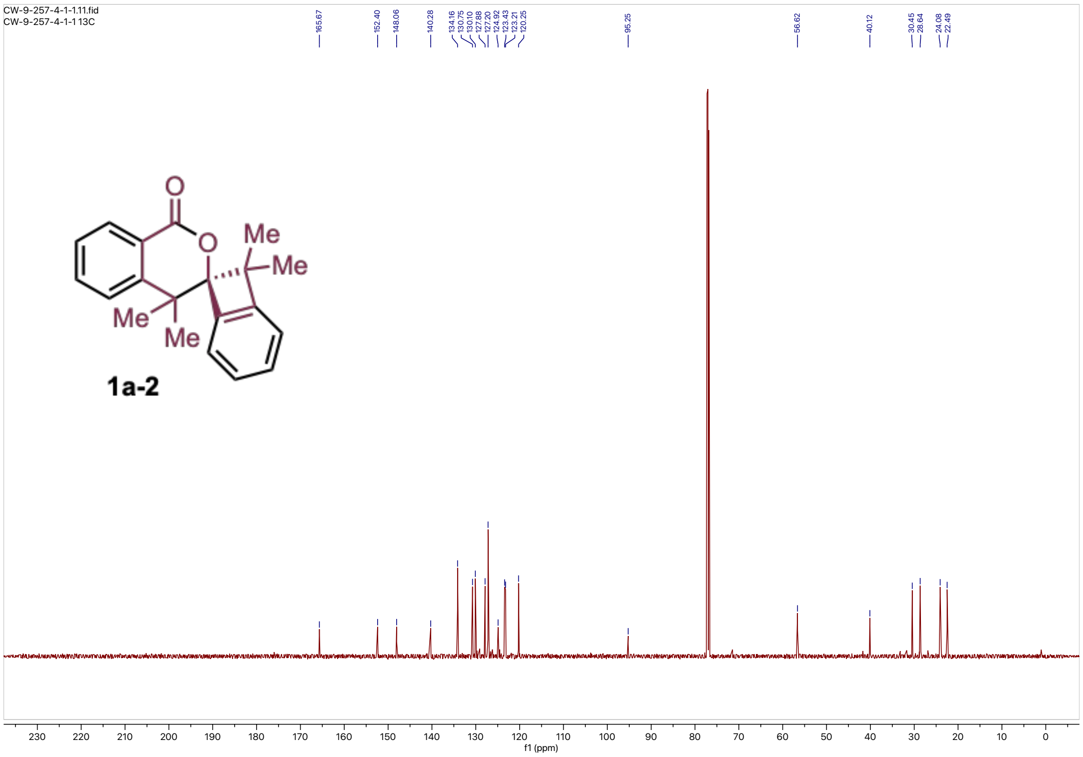

COSY NMR for **1a-2**

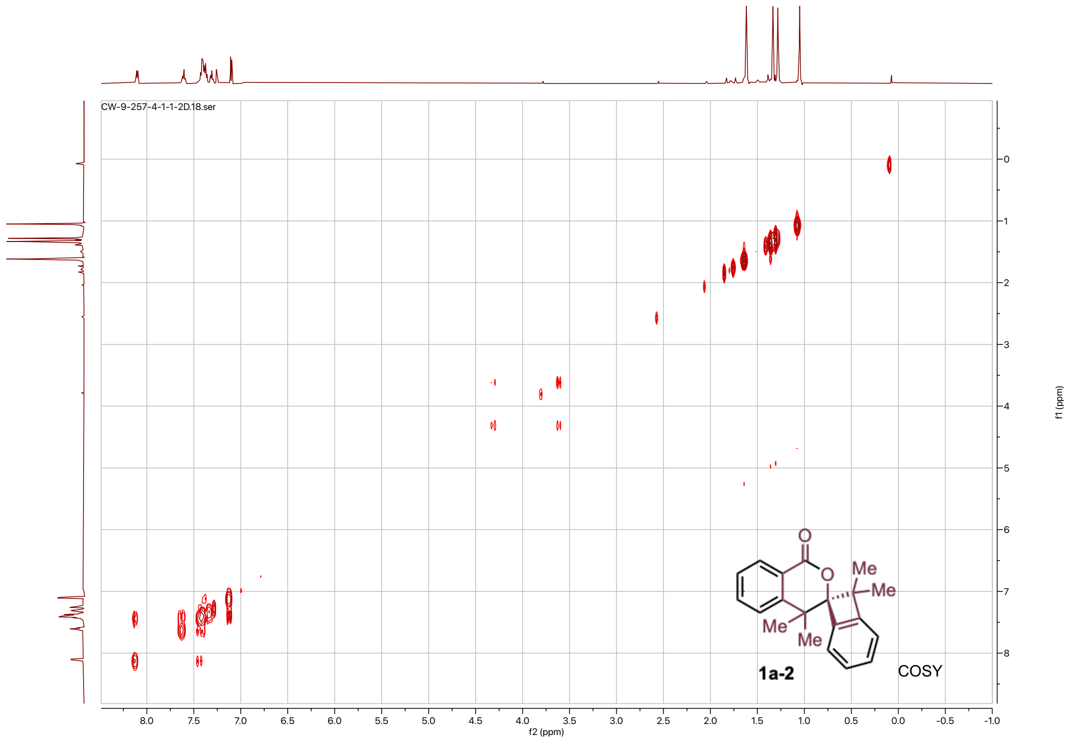

HSQC NMR for **1a-2**

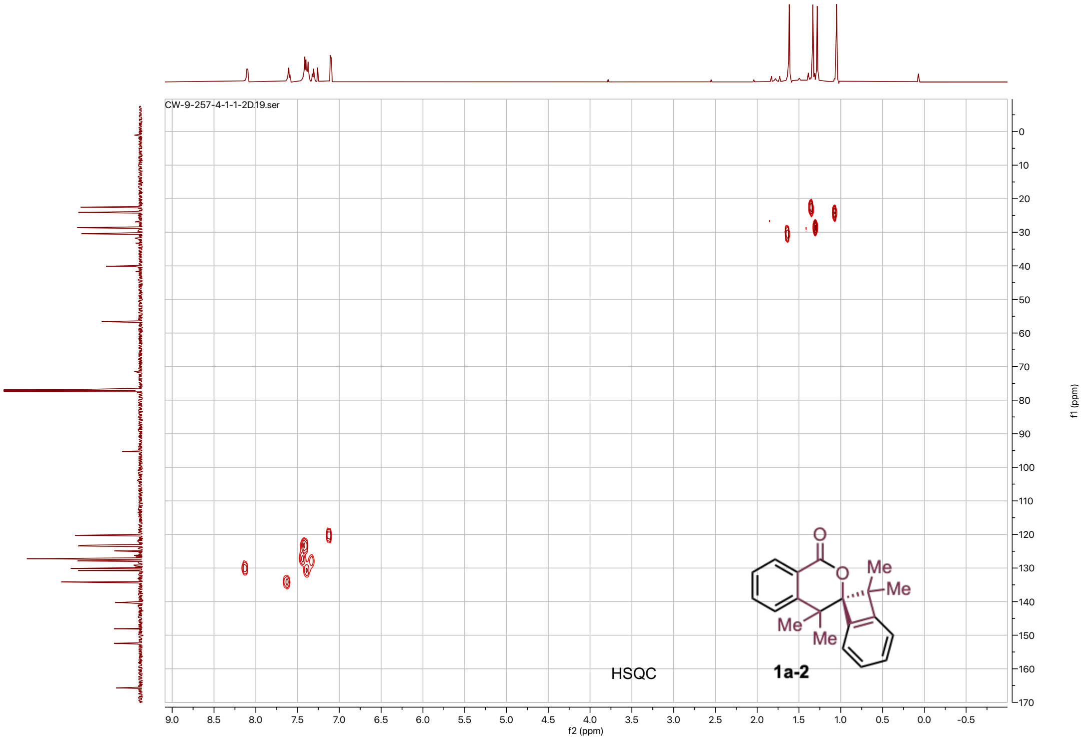

HMBC NMR for **1a-2**

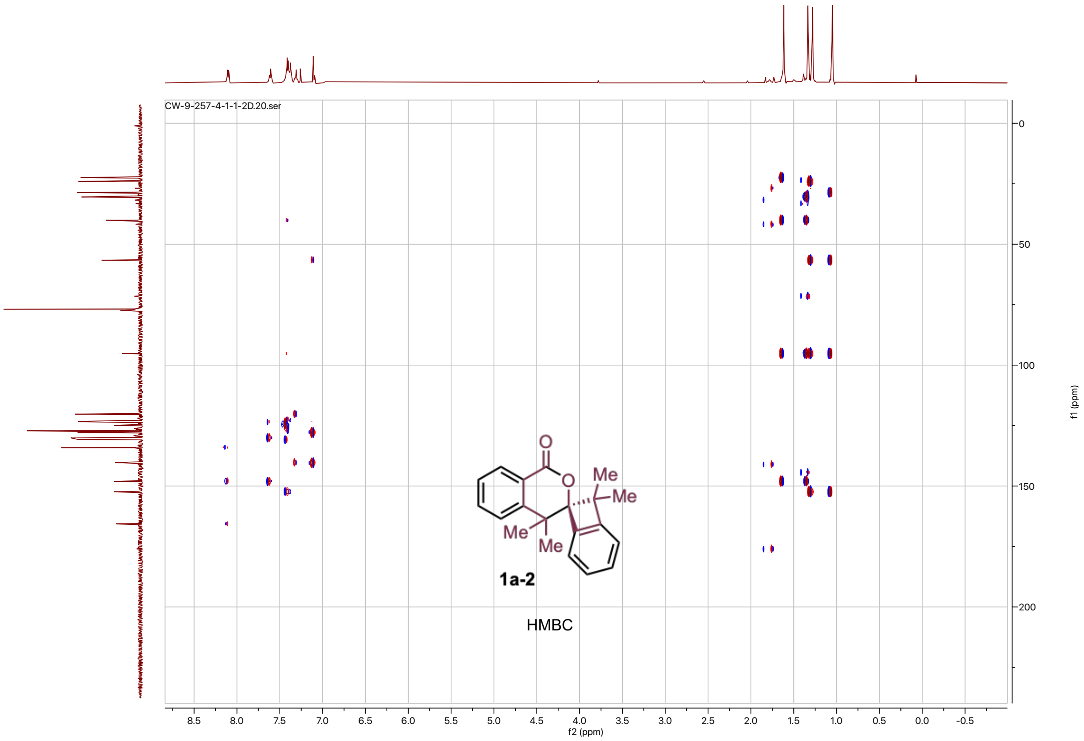

^{1}H NMR for **2a'-THF** (500 MHz, C_6D_6)

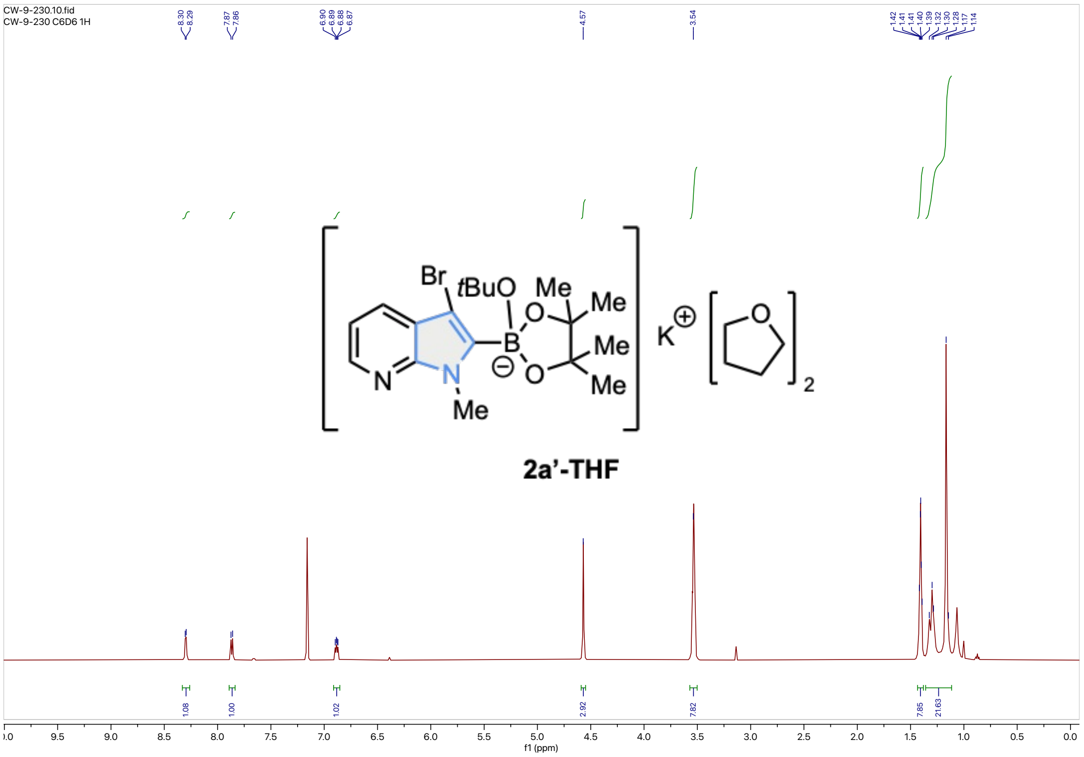

^{13}C NMR for **2a'-THF** (126 MHz, C_6D_6)

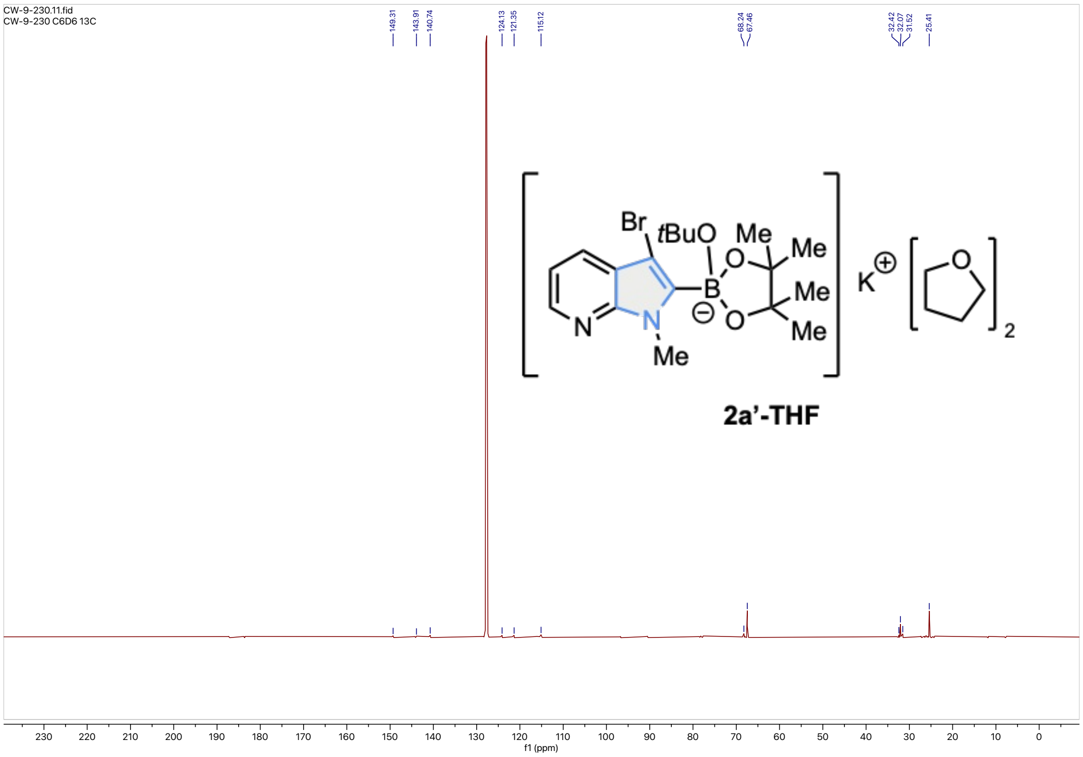

^{11}B NMR for **2a'-THF** (160 MHz, C_6D_6)

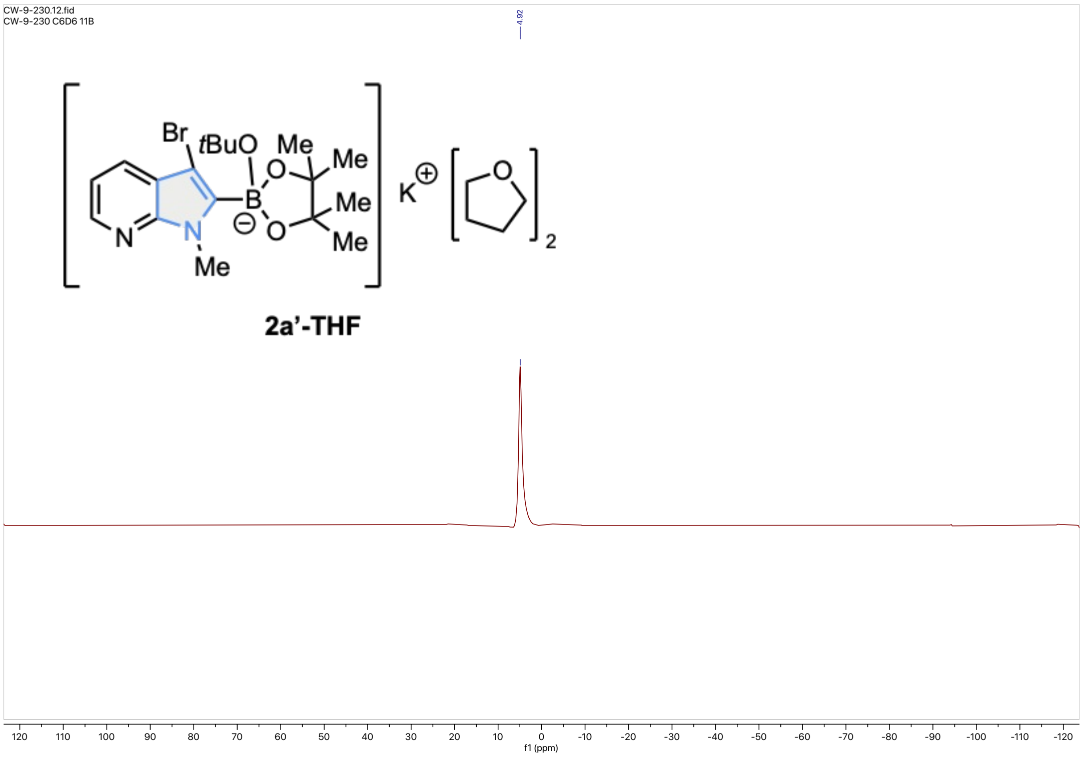

^{1}H NMR for **4** (500 MHz, $CDCl_3$)

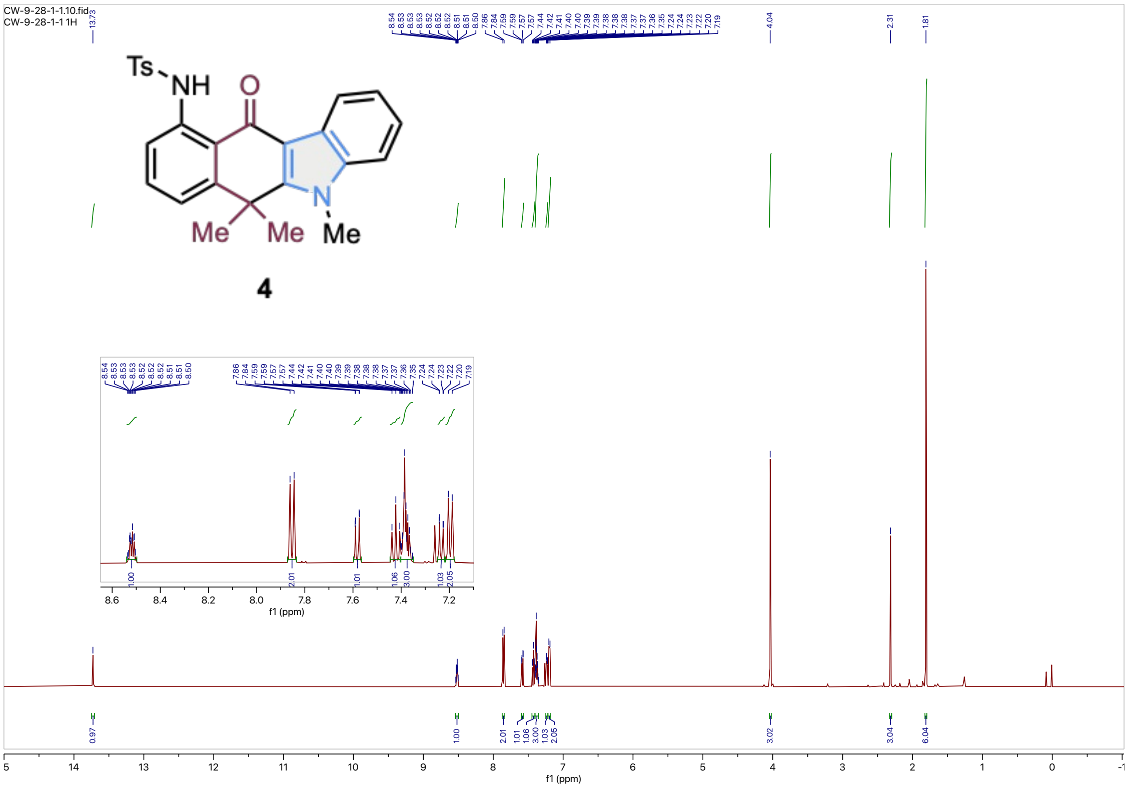

^{13}C NMR for **4** (126 MHz, $CDCl_3$)

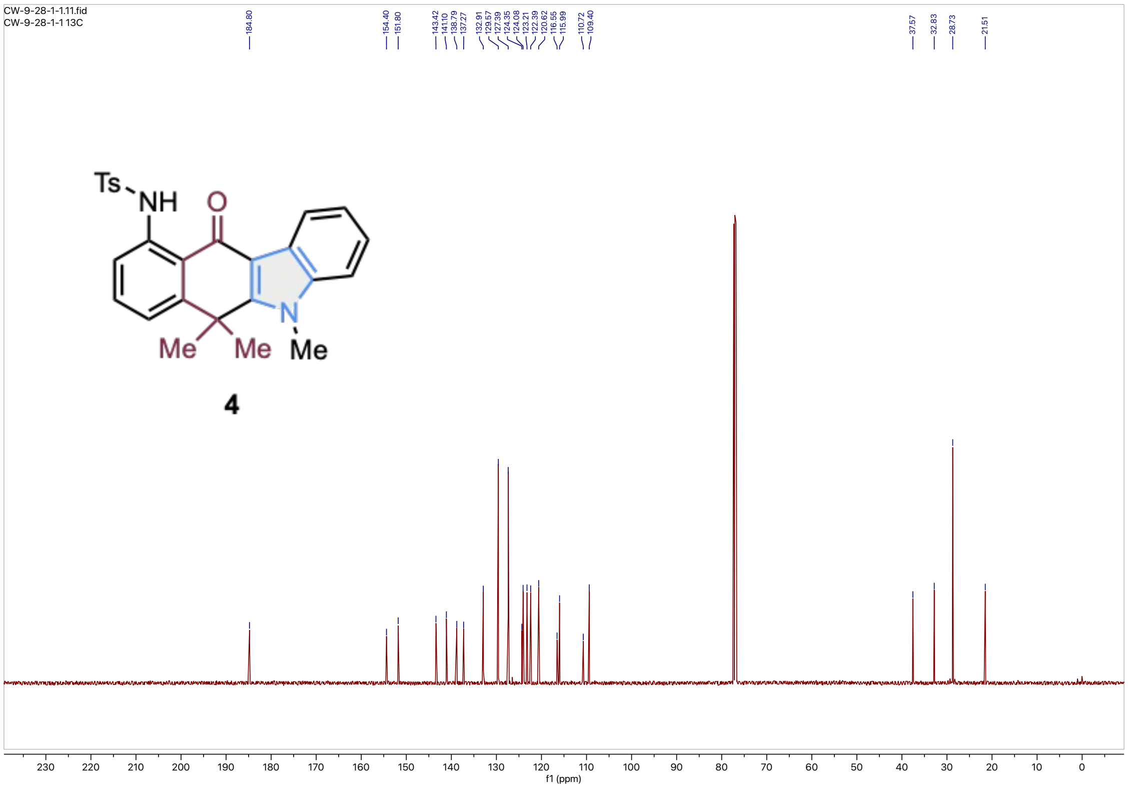

^{1}H NMR for **5**, 92% purity (500 MHz, $CDCl_3$)

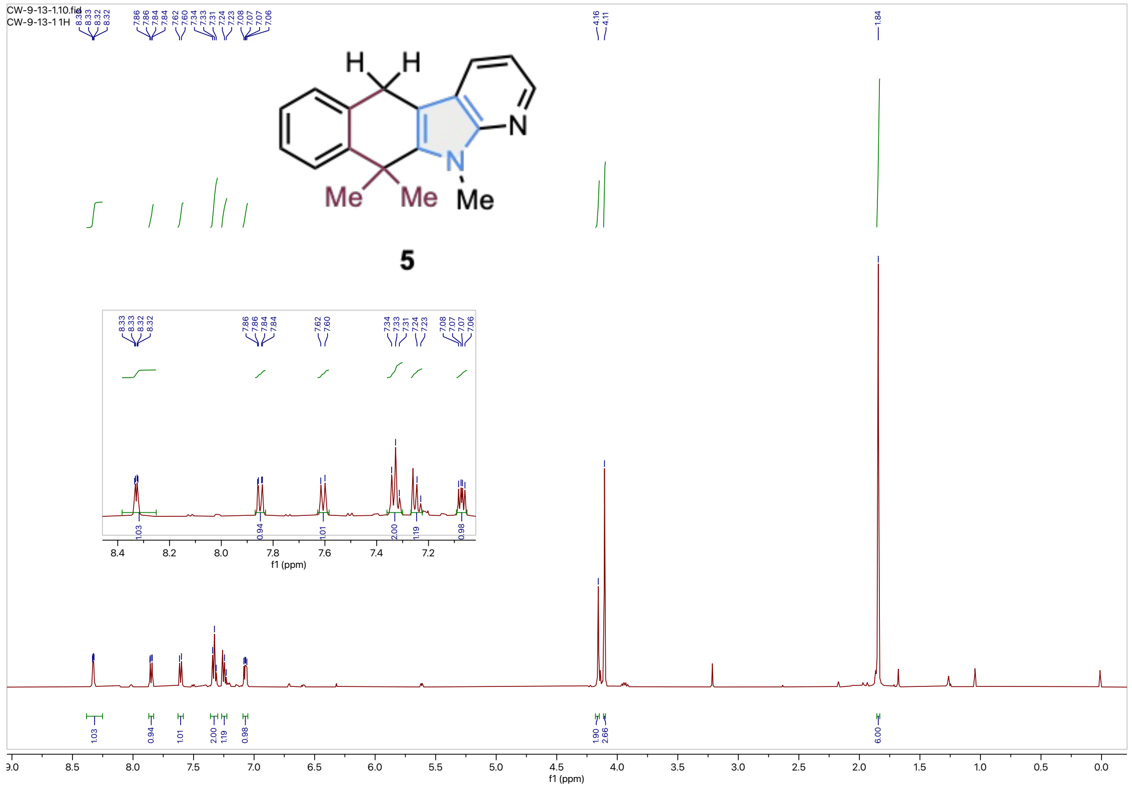

^{13}C NMR for **5**, 92% purity (126 MHz, $CDCl_3$)

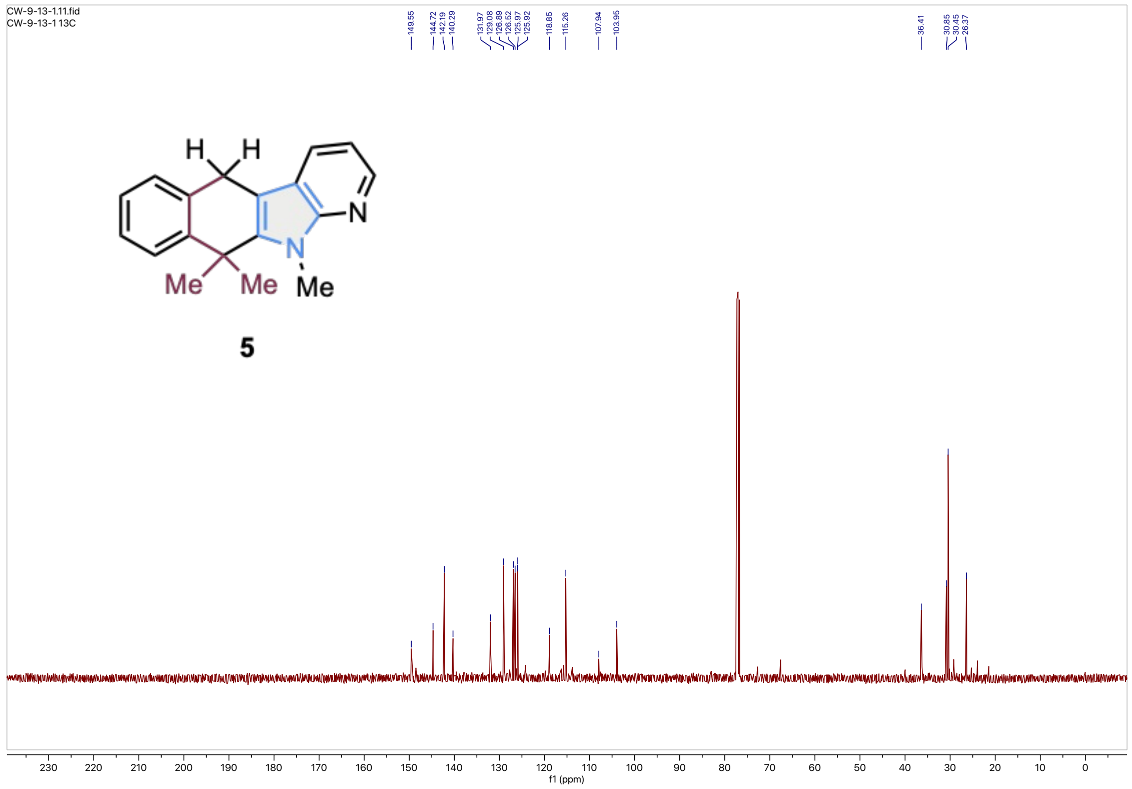

^{1}H NMR for **6** (500 MHz, $CDCl_3$)

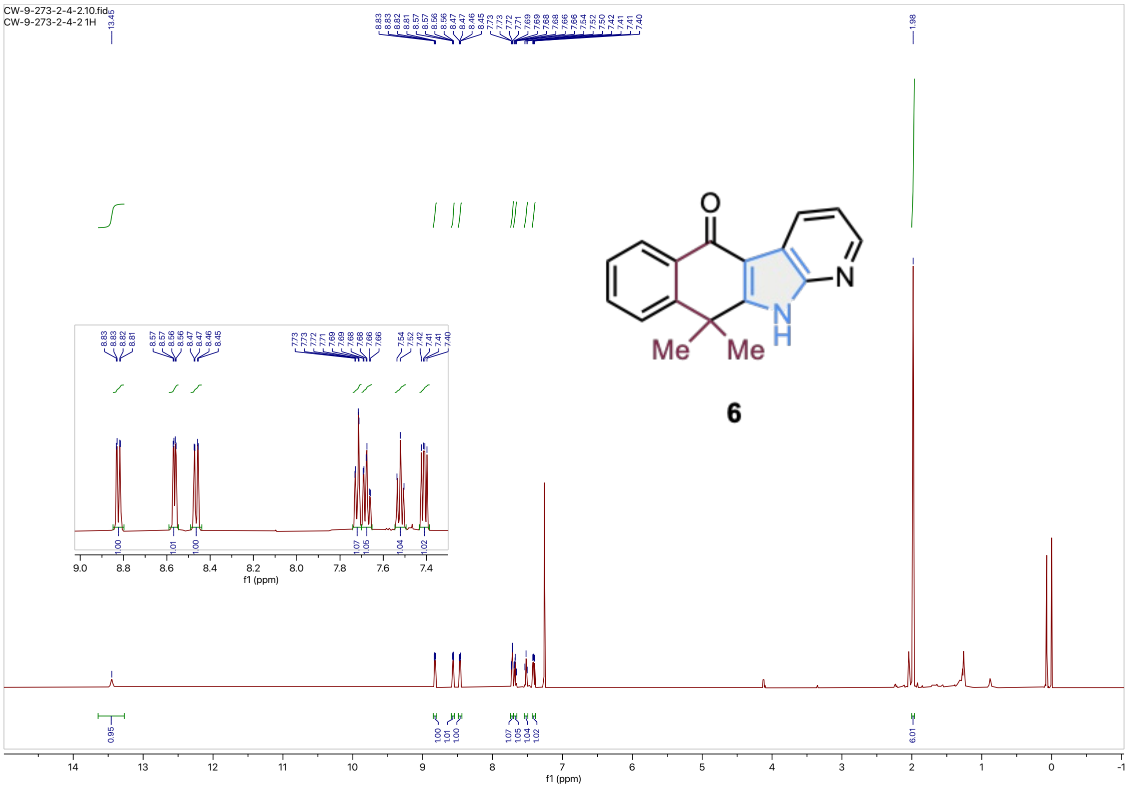

^{13}C NMR for **6** (126 MHz, $CDCl_3$)

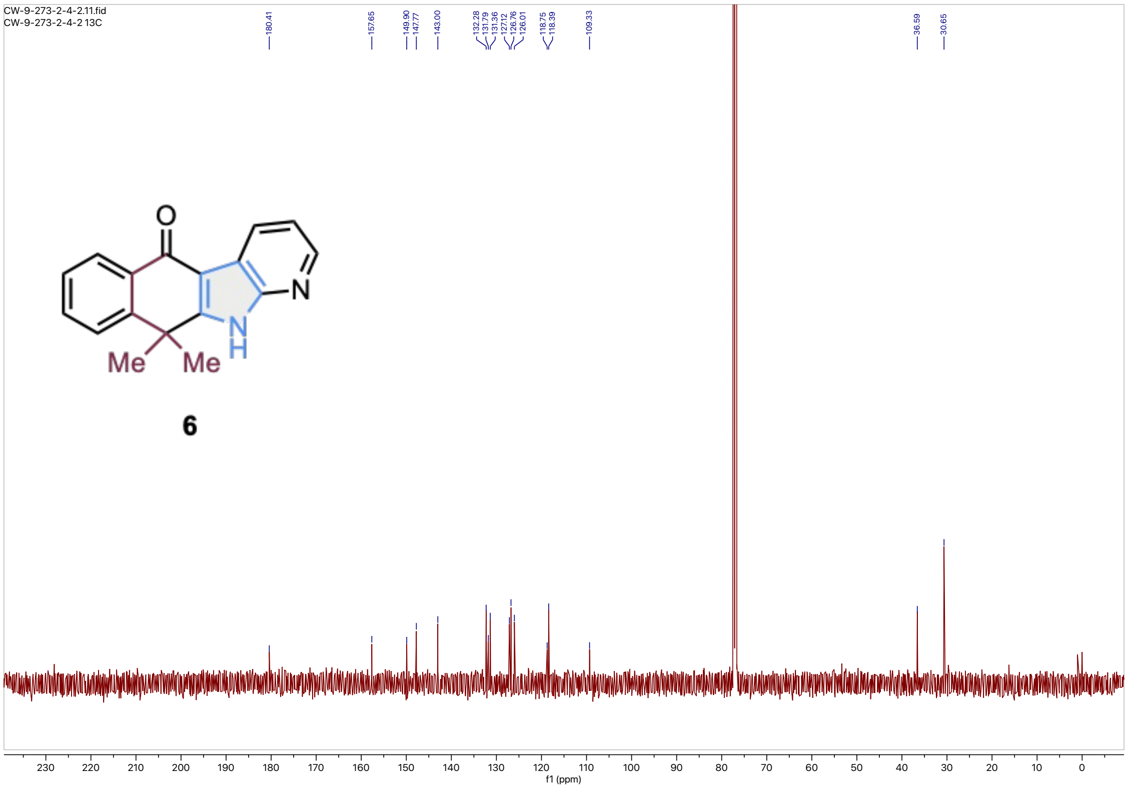

^{1}H NMR for **7** (500 MHz, $CDCl_3$)

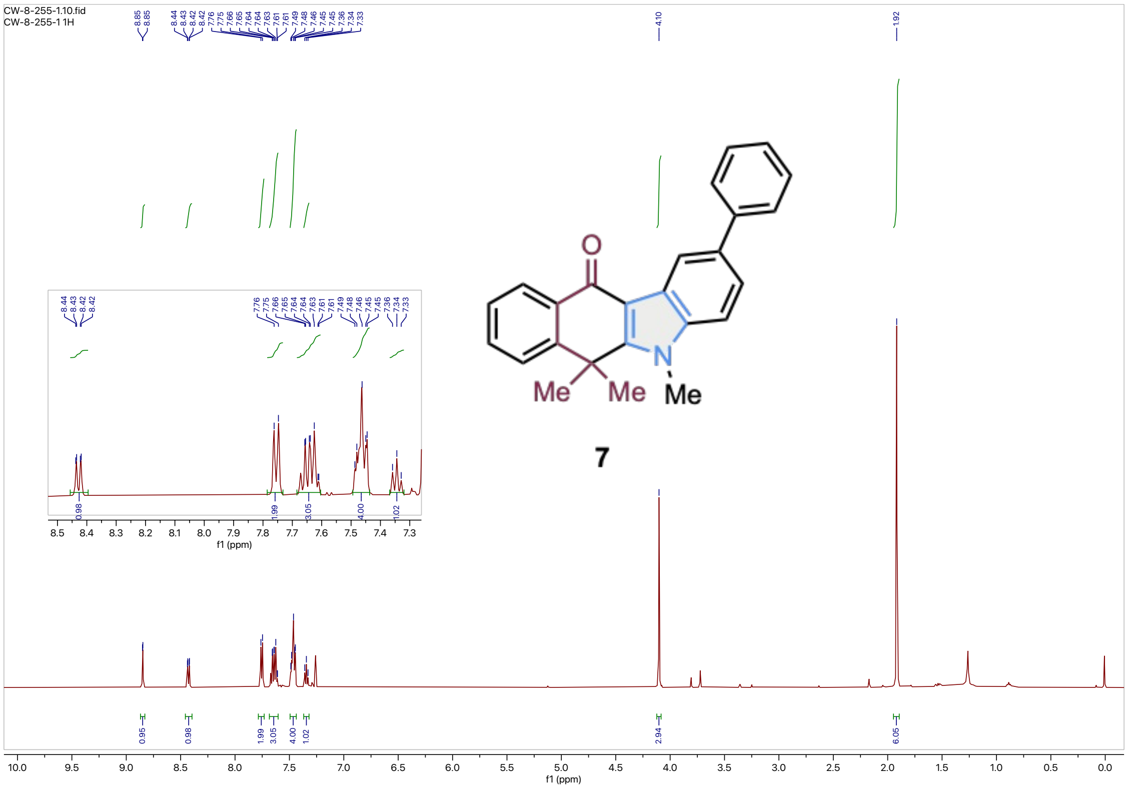

^{13}C NMR for **7** (126 MHz, $CDCl_3$)

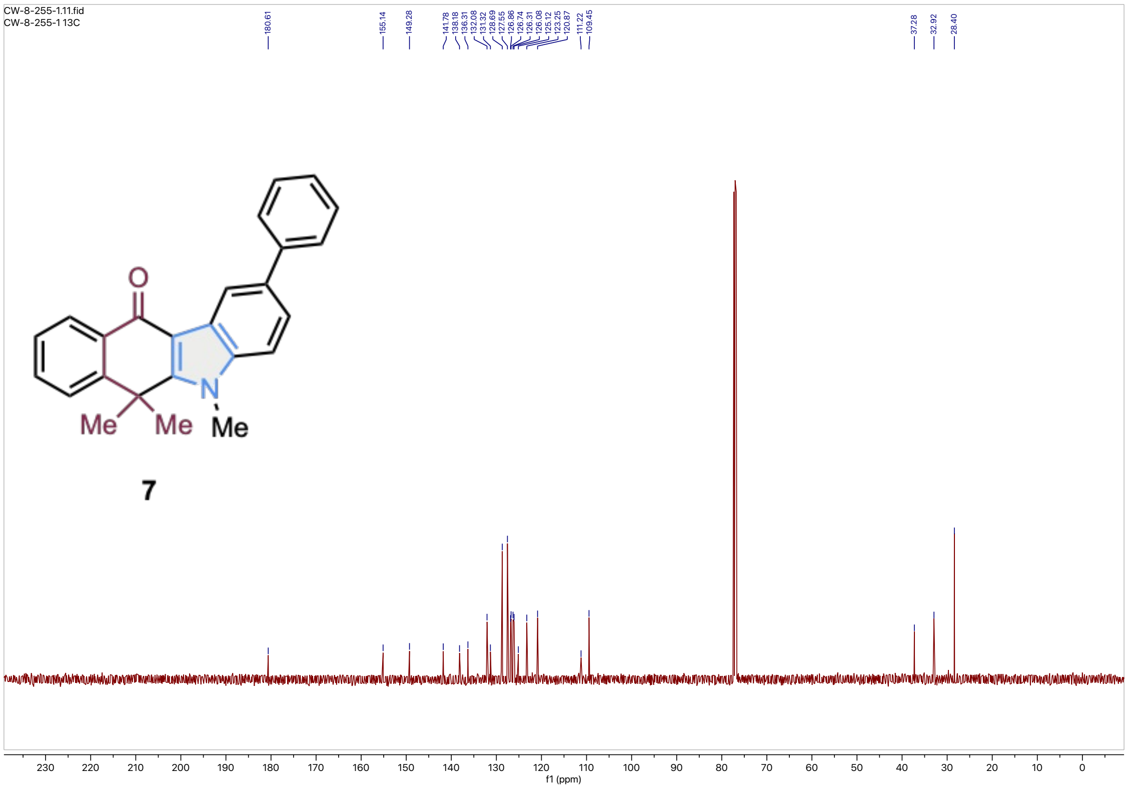

^{1}H NMR for **8** (500 MHz, $CDCl_3$)

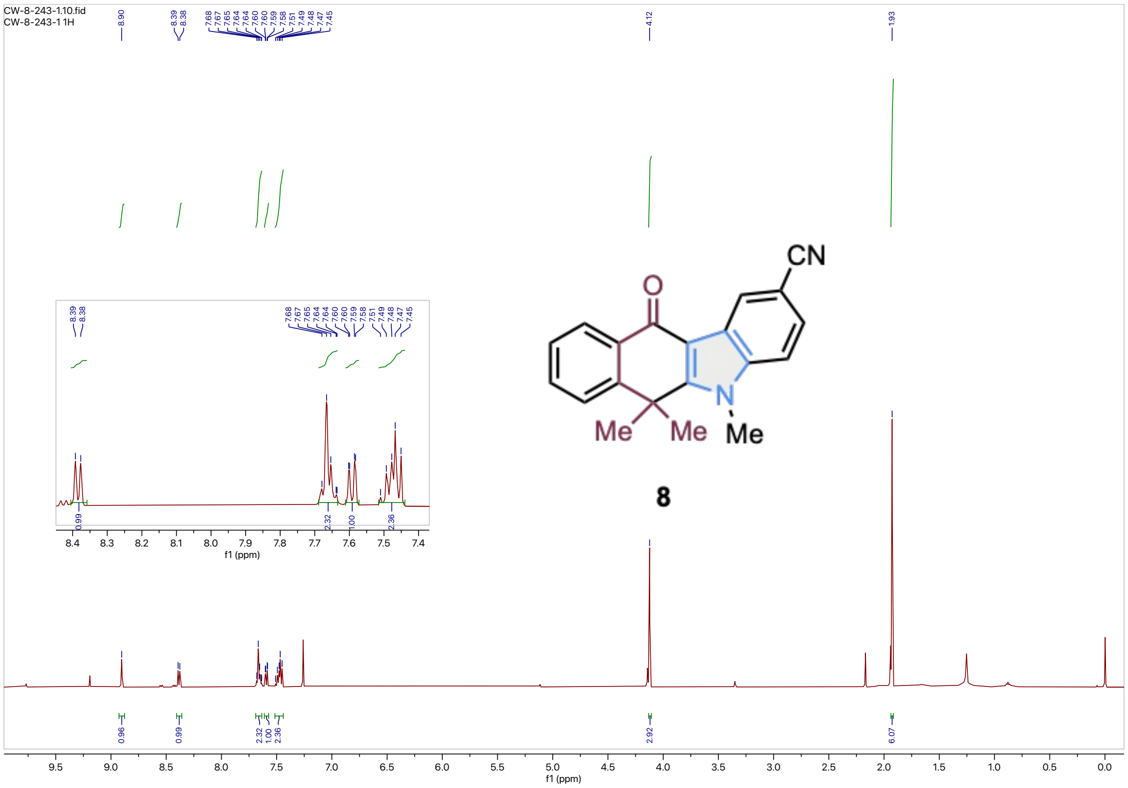

^{13}C NMR for **8** (126 MHz, $CDCl_3$)

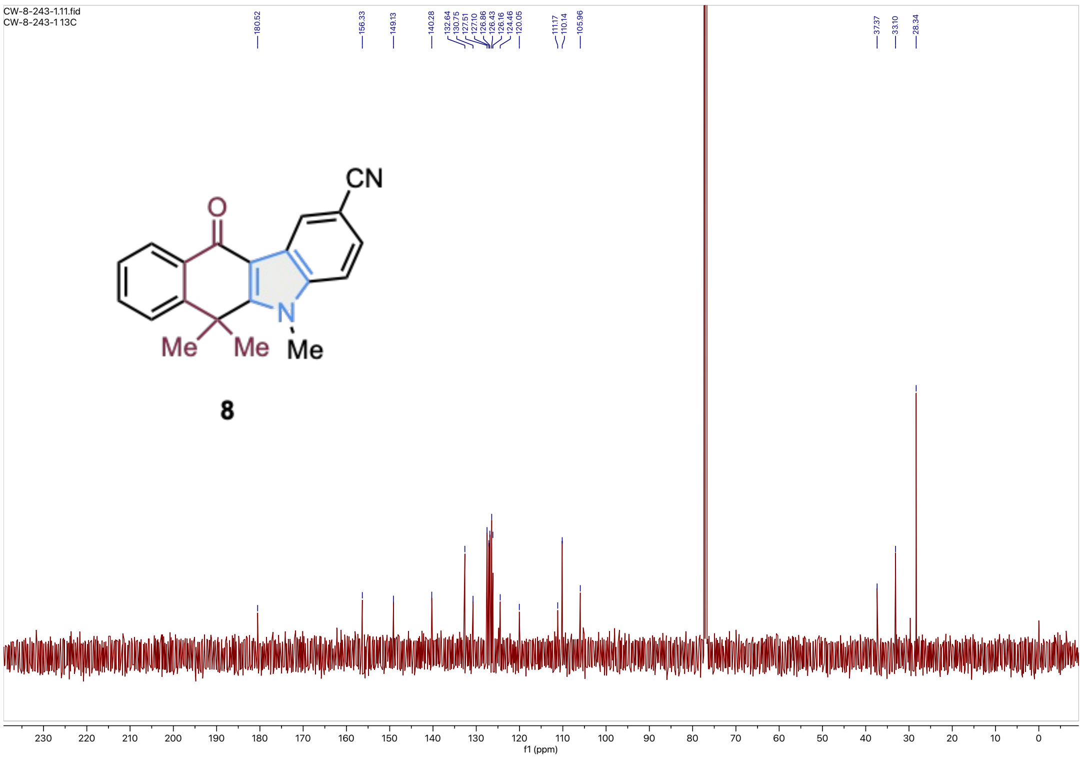

Supplement: Supplementary file 1 — Supporting File 1: The authors have cited additional references within the Supporting Information [47–66]. [file ANIE-65-e6371423-s002.pdf]
